# Tracking disease resistance deployment in potato breeding by enrichment sequencing

**DOI:** 10.1111/pbi.12997

**Published:** 2018-09-19

**Authors:** Miles R. Armstrong, Jack Vossen, Tze Yin Lim, Ronald C. B. Hutten, Jianfei Xu, Shona M. Strachan, Brian Harrower, Nicolas Champouret, Eleanor M. Gilroy, Ingo Hein

**Affiliations:** ^1^ CMS The James Hutton Institute Dundee UK; ^2^ Wageningen University Wageningen The Netherlands; ^3^ Chinese Academy of Agricultural Science (CAAS) Beijing China; ^4^ Simplot Plant Sciences Boise Idaho USA; ^5^ School of Life Sciences Division of Plant Sciences at the James Hutton Institute University of Dundee Dundee UK; ^6^ Present address: Department of Medicine Columbia University New York NY USA

**Keywords:** disease resistance genes, dRenSeq, crops, breeding, tracking of NLRs, potato

## Abstract

Following the molecular characterisation of functional disease resistance genes in recent years, methods to track and verify the integrity of multiple genes in varieties are needed for crop improvement through resistance stacking. Diagnostic resistance gene enrichment sequencing (dRenSeq) enables the high‐confidence identification and complete sequence validation of known functional resistance genes in crops. As demonstrated for tetraploid potato varieties, the methodology is more robust and cost‐effective in monitoring resistances than whole‐genome sequencing and can be used to appraise (trans) gene integrity efficiently. All currently known NB‐LRRs effective against viruses, nematodes and the late blight pathogen *Phytophthora infestans* can be tracked with dRenSeq in potato and hitherto unknown polymorphisms have been identified. The methodology provides a means to improve the speed and efficiency of future disease resistance breeding in crops by directing parental and progeny selection towards effective combinations of resistance genes.

## Introduction

To sustain a potential world population of 9.1 billion by 2050, food production is required to increase by up to 70% compared to 2005–2007 levels (FAO, 2009). Plant pathogens represent a continuous and serious threat towards this goal and significantly reduce crop yields. The arrival into Europe of the oomycete pathogen *Phytophthora infestans,* for example, led to the Irish potato famine in the mid 1840s. The famine resulted in the death of more than a million people due to starvation and forced the emigration of approximately two million people from Ireland following successive failures of potato crop production (Donnelly, 2001). Late blight remains the most devastating disease of potato and, alongside other pathogens such as cyst nematodes and viruses, continues to threaten global potato production (Birch *et al*., 2012).

The realisation that resistances against pathogens could be introduced into potato cultivars from wild species (Rudorf *et al*., 1949) led to the establishment of international germplasm collections. These collections have been used to introgress genes such as *R1‐R11* from *Solanum demissum* into varieties to control late blight (Black *et al*., 1953) and are now being systematically explored to identify additional novel resistances (Van Weymers *et al*., 2016; Vossen *et al*., 2014). Owing to the advances in genomics and genetics technologies, numerous functional plant nucleotide‐binding, leucine‐rich‐repeat resistance genes (NLRs) have been subsequently cloned that control diverse pathogens such as potato virus X (Bendahmane *et al*., 1999), potato cyst nematodes (van der Vossen *et al*., 2000) and the late blight pathogen *Phytophthora infestans* (Hein *et al*., 2009). Similar germplasm resources exist for major crops including wheat, rice, and maize (FAO, 2010) and are being explored for beneficial genes including NLRs (Kourelis and van der Hoorn, 2018).

It has been estimated that controlling major diseases through, for example, the informed deployment of functional resistance genes, could contribute over 30% towards crop yield whilst reducing the requirements for chemical applications (Gebhardt and Valkonen, 2001).

Such developments in agricultural crop production are dependent on the implementation of new breeding tools to select resistant genotypes and deploy them as varieties. This can be particularly challenging where multiple disease resistances are to be combined in order to take advantage of epistatic interactions (Haesaert *et al*., 2015). To this end we have advanced diagnostic Resistance gene enrichment Sequencing (dRenSeq), to expedite the process of identifying and validating the sequence integrity of known functional resistance genes in cultivars and breeding lines. The methodology takes advantage of the focused re‐sequencing of NLRs through RenSeq (Jupe *et al*., 2013). RenSeq has previously been used for improving genome annotations and genetic mapping of plant NLRs (Chen *et al*., 2018; Jupe *et al*., 2013), the prioritisation of novel NLRs in wild diploid species (Jiang *et al*., 2018; Van Weymers *et al*., 2016), and identification of candidate NLRs when combined with long‐read sequencing technology (Giolai *et al*., 2016; Witek *et al*., 2016).

Here we demonstrate the applicability of dRenSeq to illuminate the presence of functional NLR genes in tetraploid potatoes, the most important non‐cereal food crop (Birch *et al*., 2012). DRenSeq methodology is able to identify and validate all currently known NLRs effective against potato virus X, potato cyst nematode *Globodera pallida* and *P. infestans*. Used as a diagnostic tool in polyploid varieties, dRenSeq provides a robust application that can be utilised to study resistances in any crop where NLRs control diseases.

## Results

### dRenSeq identifies functional NLRs and appraises transgene integrity

**Figure 1:**
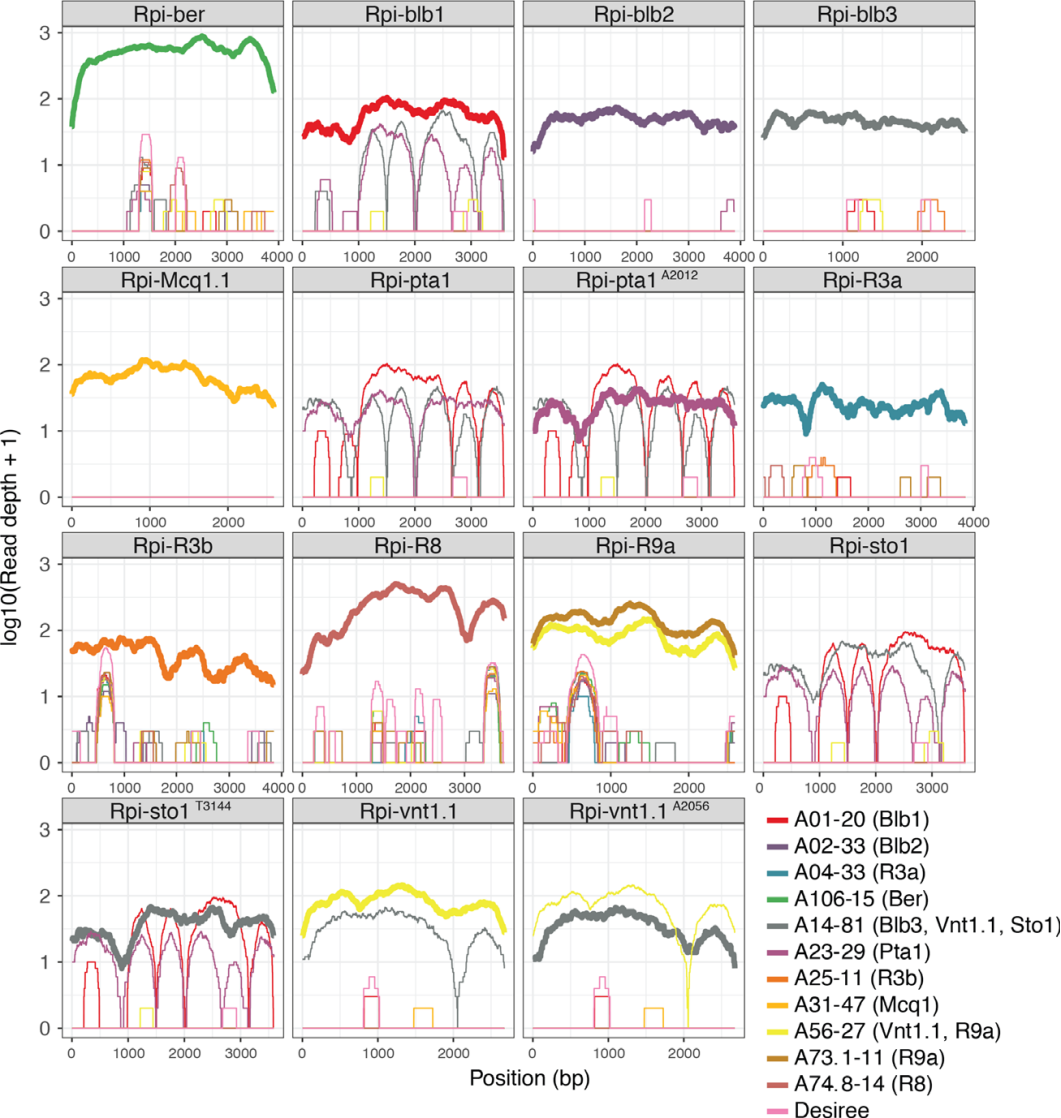
DRenSeq analysis in tetraploid potatoes. DRenSeq analysis of 11 transgenic potato lines derived from the variety Desiree. The sequence representation of known NLRs effective against late blight are shown in each box. The *x*‐axis depicts the coding DNA sequence (CDS) and the *y*‐axis the read‐coverage on a log scale. Thick horizontal lines indicate full sequence representation without any sequence polymorphisms between the reference and the NLR enriched reads.

The efficacy of dRenSeq in accurately identifying known functional NLRs in crops was initially assessed in 11 transgenic lines derived from the potato variety Desiree that is susceptible towards late blight (Zhu *et al*., 2012, 2014). The plants contained (combinations of) 14 known NLRs effective against *P. infestans* (*Rpi* genes) (Figure 1). Twelve genomic DNA samples from the transgenic and non‐transformed Desiree plants were indexed prior to NLR enrichment and sequenced on a single lane of Illumina MiSeq (Jupe *et al*., 2013). For dRenSeq, only NLR enriched paired‐end reads were mapped, without allowing for any high‐quality mismatches, to a reference set of functionally validated NLRs, including their 5′ and 3′ flanking regions (Material and Methods). The representation of individual, full‐length NLRs was calculated by extracting the sequence coverage of dRenSeq‐mapped reads to the reference coding DNA sequence (CDS) (Table 1). We define a resistance gene as “present” if 100% of the CDS is represented by dRenSeq‐mapped reads.

**Table 1:** NLR coverage in transgenic Desiree lines

	Transgenic Desiree lines											
ID	A01‐20	A02‐33	A04‐33	A106‐15	A14‐81	A23‐29	A25‐11	A31‐47	A56‐27	A73‐1‐11	A74‐8‐14	wt
*Rpi* ‐ transgene (s)	*Blb1*	*Blb2*	*R3a*	*Ber*	*Blb3; Sto1; Vnt1.1*	*Pta1*	*R3b*	*Mcq1.1*	*R9a; Vnt1.1*	*R9a*	*R8*	none
Gene name												
* Rpi‐mcq1.1*	0.00	0.00	0.00	0.00	0.00	0.00	0.00	100.00	0.00	0.00	0.00	0.00
* Rpi‐R3a*	6.52	0.00	100.00	0.00	0.00	0.00	13.98	0.00	0.00	19.04	8.29	13.48
* Rpi‐R3b*	9.27	37.12	15.81	19.24	29.13	15.45	100.00	9.22	26.40	32.06	15.13	23.65
* Rpi‐R8*	19.93	8.08	24.77	18.73	29.21	13.59	19.88	21.11	19.64	41.92	100.00	44.17
* Rpi‐R9a*	17.67	28.70	16.86	49.96	27.20	37.96	29.71	36.50	100.00	100.00	47.07	39.08
* Rpi‐ber*	13.15	12.94	6.40	100.00	17.06	12.69	11.90	25.74	25.35	16.09	22.51	11.97
* Rpi‐blb1*	100.00	0.00	0.00	0.00	77.92	82.54	0.00	0.00	15.90	0.00	0.00	6.99
* Rpi‐blb2*	0.00	100.00	0.00	0.00	0.00	6.92	0.00	0.00	0.00	0.00	0.00	4.29
* Rpi‐blb3*	13.52	0.00	0.00	0.00	100.00	0.00	13.21	0.00	11.05	0.00	0.00	10.53
* Rpi‐pta1*	88.64	0.00	0.00	0.00	97.19	98.52	0.00	0.00	6.35	0.00	0.00	6.99
* Rpi‐pta1* ^*A2012*^	87.86	0.00	0.00	0.00	97.19	100.00	0.00	0.00	6.35	0.00	0.00	6.99
* Rpi‐sto1*	78.82	0.00	0.00	0.00	99.22	94.99	0.00	0.00	15.89	0.00	0.00	6.99
* Rpi‐sto1* ^*T3144*^	78.65	0.00	0.00	0.00	100.00	94.99	0.00	0.00	6.35	0.00	0.00	6.99
* Rpi‐vnt1.1*	7.51	0.00	0.00	0.00	99.93	0.00	0.00	9.38	100.00	0.00	0.00	7.96
* Rpi‐vnt1.1* ^*A2056*^	7.51	0.00	0.00	0.00	100.00	0.00	0.00	9.38	99.85	0.00	0.00	7.96

DRenSeq was simultaneously conducted in 11 transgenic Desiree lines alongside a wild‐type (wt) Desiree control. The IDs of the transgenic lines and the Resistance to *Phytophthora infestans (Rpi)* nucleotide‐binding, leucine‐rich‐repeat resistances transgenes are shown. The representation of individual, full‐length *Rpi* genes was calculated by extracting the sequence coverage of dRenSeq‐mapped reads to the reference coding DNA sequence (CDS). Highlighted in green are *Rpi* genes that achieved 100% representation and are therefore classified as ‘present’.

The analysis confirmed the presence of expected single and multiple NLRs but also identified, with high‐confidence, previously unknown sequence variations within transgenes. Full sequence representation was initially observed for 11 of the 14 NLRs. Surprisingly, *Rpi‐sto1* and *Rpi‐vnt1.1* in transgenic line A14‐81 as well as *Rpi‐pta1* in line A23‐29 achieved ‘only’ between 98.5% and 99.9% CDS coverage. Remapping NLR‐enriched paired‐end reads from these lines under less stringent conditions, which allowed a single high‐quality SNP per read pair, revealed unexpected sequence variations. We refer to these variants as *Rpi‐sto1* ^*T3144*^ *, Rpi‐vnt1.1* ^*A2056*^ and *Rpi‐pta1* ^*A2012*^ whereby the affected nucleotide position after the CDS start is indicated alongside the nucleotide substitution (Table S1). All independent paired‐end reads supporting the sequence polymorphisms were monomorphic for the substitutions and no reads were identified that supported the reference nucleotide at these loci (Figure S1).a–c Crucially, all these predicted sequence polymorphisms were independently validated by Sanger‐based re‐sequencing of the plasmids used to generate the transgenic lines as well as PCR products derived from the transgenic plants themselves. This shows that the polymorphisms arose during gene cloning and that dRenSeq provides the high‐resolution required to identify single nucleotide polymorphisms, even amongst a background of related endogenous NLR sequences. Upon generation of new references that incorporate the confirmed polymorphisms, all 14 NLRs were fully represented thus demonstrating the efficacy of the dRenSeq method (Figure 1 and Table 1).

### dRenSeq validates *Rpi‐vnt1.1* deployment in deregulated GM potato varieties

Following the successful validation of dRenSeq in transgenic Desiree plants, the method was applied to the Innate® Generation 2 transgenic lines Glaciate, Acclimate and Hibernate. These lines have been successfully deregulated in the USA after the introduction of the late blight resistance gene *Rpi‐vnt1.1* (Foster *et al*., 2009). Also included in the study were the respective progenitor potato varieties Russet Burbank (for Glaciate), Ranger Russet (for Acclimate) and Atlantic (for Hibernate).

**Figure 2:**
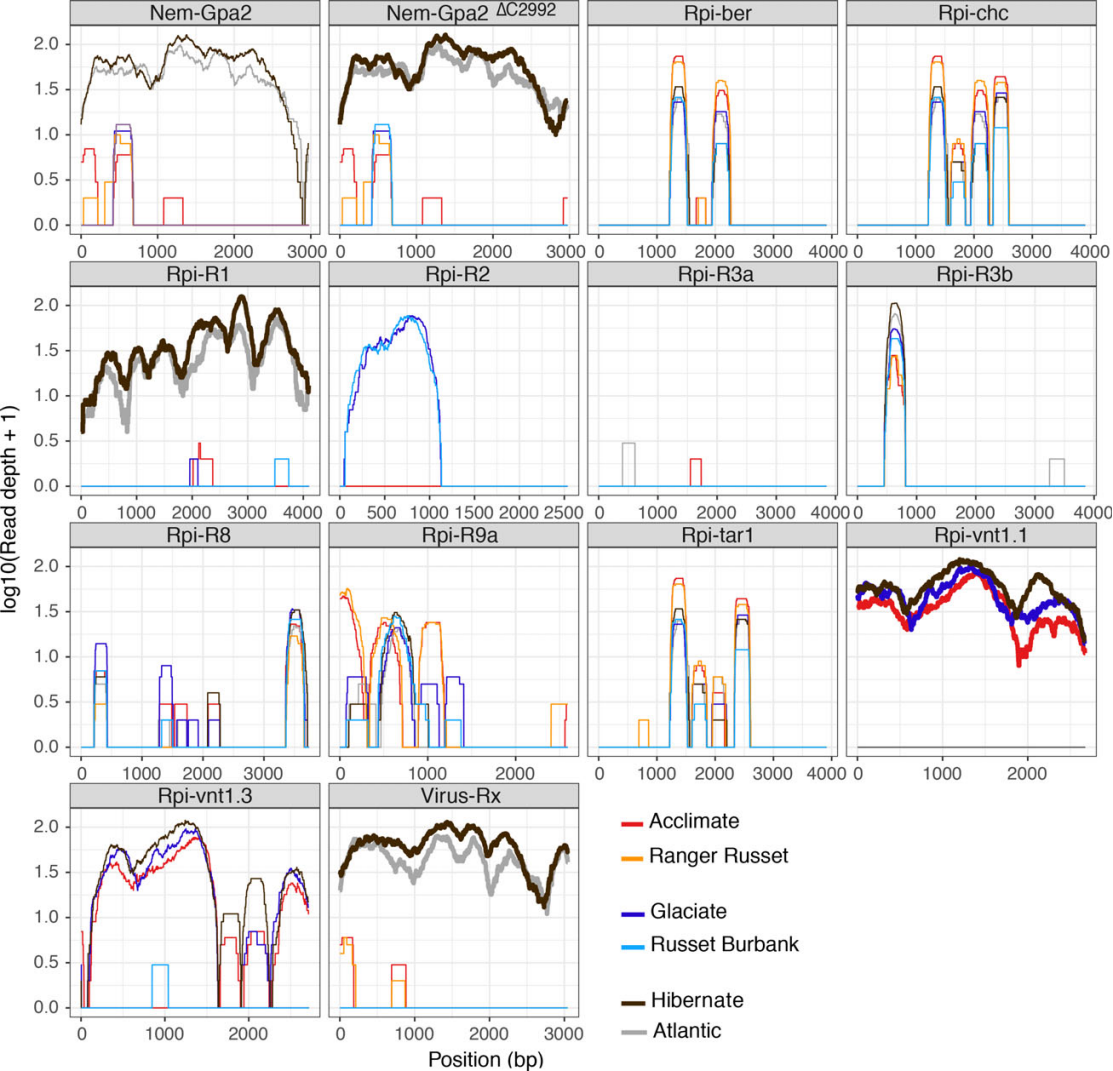
DRenSeq analysis in Innate® generation 2 transgenic lines Glaciate, Acclimate and Hibernate alongside the progenitor varieties Russet Burbank, Ranger Russet and Atlantic. The sequence representation of known NLRs effective against late blight (*Rpi*), nematodes (*Nem*), and viruses (*Virus*) are shown in each box. The *x*‐axis depicts the coding DNA sequence (CDS) and the *y*‐axis the read‐coverage on a log scale. Thick horizontal lines indicate full sequence representation without any sequence polymorphisms between the reference and the NLR enriched reads.

The application of dRenSeq confirmed the presence of functional *Rpi‐vnt1.1* in these Innate® transgenic lines and the absence of this gene in all respective progenitor plants (Figure 2). In addition, dRenSeq enabled the identification and sequence validation of NLRs effective against diverse pathogens such as nematodes (*Nem*) and viruses (*Virus*) (Table 2). For example, dRenSeq revealed that the variety Atlantic contains the late blight resistance gene *R1*, the virus resistance gene *Rx* and the nematode resistance gene *Gpa2*. Within *Gpa2*, a deletion in the intron sequence was identified which does not impact on the NLR protein sequences. We refer to this deletion as *Nem‐Gpa2* ^*ΔC2922*^ and subsequently observed the same polymorphism in Hibernate. As anticipated, dRenSeq also independently identified the presence of *Rpi‐R1* and *Rx* alongside *Rpi‐vnt1.1* in Hibernate*,* which provides evidence for the robustness of the approach (Figure 2 and Table 2). Where we observed significant partial coverage of referenced NLRs other than the transgene *Rpi‐vnt1.1* and the closely related gene *Rpi‐vnt1.3*, the nucleotide positions of the partial CDS coverage and the depth of the coverage were, overall, in very good agreement between the progenitor lines and the transgenic plants (Figure 2 and Table 2). For example, dRenSeq in Russet Burbank revealed partial coverage (42.3%) of *Rpi‐R2* that encompassed the 5′ end of the CDS. The equivalent *Rpi‐R2* coverage in Glaciate amounted to 42.6% of the CDS with a very similar read‐depth and distribution of reads (Figure 2).

**Table 2:** NLR coverage in Innate® generation 2 line

ID	Atlantic	Hibernate	Ranger Russet	Acclimate	Russet Burbank	Glaciate
Gene name						
*Nem‐Gpa2*	99.2	98.9	18.8	24.5	8.9	8.8
*Nem‐Gpa2* ^*ΔC2922*^	100.0	100.0	18.8	26.4	8.9	8.8
*Rpi‐R1*	100.0	100.0	0.0	8.6	6.1	3.5
*Rpi‐R1* ^*ΔT4109*^	100.0	100.0	0.0	8.6	6.1	3.5
*Rpi‐R2*	0.0	0.0	0.0	0.0	42.3	42.6
*Rpi‐R2‐like*	0.0	0.0	0.0	0.0	0.0	0.0
*Rpi‐R3a*	5.5	0.0	0.0	4.7	0.0	0.0
*Rpi‐R3b*	15.7	9.4	9.1	9.2	9.4	9.3
*Rpi‐R3b* ^*G3111*^	15.7	9.4	9.1	9.2	9.4	9.3
*Rpi‐R3b* ^*G1696/G3111*^	15.7	9.4	9.1	9.2	9.4	9.3
*Rpi‐R8*	24.1	20.7	13.7	30.3	18.5	35.0
*Rpi‐R9a*	27.0	30.1	46.1	39.7	39.1	42.9
*Rpi‐abpt*	0.0	0.0	0.0	0.0	0.0	0.0
*Rpi‐abpt* ^*T86*^	0.0	0.0	0.0	0.0	0.0	0.0
*Rpi‐blb1*	0.0	0.0	0.0	0.0	0.0	0.0
*Rpi‐blb2*	0.0	0.0	0.0	0.0	0.0	0.0
*Rpi‐blb3*	0.0	0.0	0.0	0.0	0.0	0.0
*Rpi‐pta1*	0.0	0.0	0.0	0.0	0.0	0.0
*Rpi‐pta1* ^*A2012*^	0.0	0.0	0.0	0.0	0.0	0.0
*Rpi‐sto1*	0.0	0.0	0.0	0.0	0.0	0.0
*Rpi‐sto1* ^*T3144*^	0.0	0.0	0.0	0.0	0.0	0.0
*Rpi‐vnt1.1*	0.0	100.0	0.0	100.0	7.2	100.0
*Rpi‐vnt1.1* ^*A2056*^	0.0	96.3	0.0	96.3	7.2	98.8
*Rpi‐vnt1.3*	0.0	95.5	0.0	94.2	7.1	86.9
*Virus‐Rx*	100.0	100.0	12.8	12.4	0.0	0.0

DRenSeq was conducted on Innate® generation 2 transgenic lines Glaciate, Acclimate and Hibernate alongside the progenitor varieties Russet Burbank, Ranger Russet and Atlantic. The name of the varieties and nucleotide‐binding, leucine‐rich‐repeat resistances (NLR) effective against diverse pathogens such as nematodes (*Nem*), late blight (*Rpi*) and viruses (*Virus*) are shown. The representation of individual resistance genes was calculated by extracting the sequence coverage of dRenSeq‐mapped reads to the reference coding DNA sequence (CDS). Highlighted in green are resistance genes that achieved 100% representation and are therefore classified as ‘present’.

### dRenSeq corroborates NLRs in characterised varieties and identifies sequence polymorphisms

Following the successful validation of dRenSeq in transgenic plants with different progenitors, the method was applied to 12 distinct tetraploid potatoes to further assess the performance of dRenSeq in individual varieties. Similar to the transgenic plants and variety Atlantic, sequence variations compared to reference sequences were identified within certain NLRs and independently detected in multiple varieties (Tables S1 and 3). In addition to *Nem‐Gpa2* ^*ΔC2922*^, polymorphic NLRs *Rpi‐abpt* ^*T86*^ and *Rpi‐R1* ^*ΔT4109*^ were identified which contain a synonymous substitution or a deletion in the flanking regions respectively and have no impact on the protein sequence (Table S1). However, the reference form of *Rpi‐R3b* was not observed in any of these varieties whereas *Rpi‐R3b* variants with single or double non‐synonymous nucleotide substitutions were identified. The single substitution, R3b^*G3111*^, was present in one variety and the double substitution, *Rpi‐R3b* ^*G1696/G3111*^, in 8 varieties including Innovator, Picasso and the late blight differential line 2573 (2). For the differential line 2573 (2), it has been shown previously that *Rpi‐R3b* function, such as the recognition of the corresponding effector from *P. infestans, Avr3b,* is maintained (Zhu *et al*., 2014). Similarly, *Avr3b* is recognised in Picasso and a specific cell death response is observed in *Nicotiana benthamiana* upon co‐infiltration of *Rpi‐R3b* ^*G1696/G3111*^ with *Avr3b* (Strachan *et al.,* submitted).

**Table 3:** NLR coverage in 12 potato varieties

ID	Alouette	Bionica	Cara	Craigs Snow White	Innovator	King Edward	Pentland Ace	Pentland Dell	Picasso	Spunta	Toluca	2573 (2)
Gene name												
*Nem‐Gpa2*	29.81	21.51	98.72	27.49	18.18	58.06	8.97	30.31	97.95	0.00	0.00	7.69
*Nem‐Gpa2* ^*ΔC2922*^	29.81	21.51	100.00	27.49	18.18	70.70	8.97	30.31	100.00	0.00	0.00	7.69
*Rpi‐R1*	9.34	19.21	100.00	100.00	99.20	45.73	0.00	100.00	100.00	100.00	0.00	100.00
*Rpi‐R1* ^*ΔT4109*^	9.34	19.21	100.00	99.90	100.00	45.73	0.00	99.66	99.24	99.88	0.00	100.00
*Rpi‐R2*	0.00	58.27	0.00	9.89	50.00	7.41	8.39	58.20	4.61	0.00	0.00	57.21
*Rpi‐R2‐like*	8.25	96.97	0.00	34.32	100.00	18.00	0.00	97.37	9.79	0.00	0.00	93.95
*Rpi‐R3a*	100.00	100.00	100.00	0.00	100.00	63.03	100.00	100.00	100.00	6.24	17.80	100.00
*Rpi‐R3b*	99.12	99.66	98.94	10.93	97.72	66.07	96.99	99.92	99.33	8.54	20.12	99.12
*Rpi‐R3b* ^*G3111*^	99.43	99.79	99.07	10.93	98.65	73.16	99.22	100.00	99.66	8.54	28.79	99.64
*Rpi‐R3b* ^*G1696/G3111*^	100.00	100.00	100.00	10.93	100.00	79.57	100.00	100.00	100.00	8.54	28.79	100.00
*Rpi‐R8*	41.92	46.04	19.42	46.12	25.84	29.00	31.19	38.44	20.41	28.28	27.23	100.00
*Rpi‐R9a*	32.41	50.35	27.89	47.49	5.13	50.23	47.07	40.82	36.65	39.58	51.58	100.00
*Rpi‐abpt*	8.27	96.14	0.00	24.86	93.77	18.05	8.39	95.82	9.81	0.00	0.00	96.30
*Rpi‐abpt* ^*T86*^	8.27	100.00	0.00	24.86	97.48	18.05	8.39	100.00	9.81	0.00	0.00	100.00
*Rpi‐blb1*	0.00	4.12	0.00	0.00	0.00	9.10	0.00	13.89	12.14	0.00	0.00	0.00
*Rpi‐blb2*	5.32	100.00	9.69	0.00	3.01	0.00	0.00	0.00	32.03	0.00	100.00	6.25
*Rpi‐blb3*	0.00	55.03	0.00	9.87	44.85	0.00	0.00	50.47	9.79	0.00	0.00	44.65
*Rpi‐pta1*	0.00	4.12	0.00	0.00	0.00	9.11	0.00	13.90	18.63	0.00	0.00	0.00
*Rpi‐pta1* ^*A2012*^	0.00	4.12	0.00	0.00	0.00	9.11	0.00	13.90	18.63	0.00	0.00	0.00
*Rpi‐sto1*	0.00	4.06	0.00	0.00	0.00	4.45	0.00	0.00	12.13	0.00	0.00	0.00
*Rpi‐sto1* ^*T3144*^	0.00	4.06	0.00	0.00	0.00	4.45	0.00	0.00	12.13	0.00	0.00	0.00
*Rpi‐vnt1.1*	95.48	21.71	0.11	8.78	9.30	0.00	7.47	8.82	7.14	6.95	0.00	0.00
*Rpi‐vnt1.1* ^*A2056*^	88.15	21.71	0.11	8.78	9.30	0.00	7.47	8.82	7.14	6.95	0.00	0.00
*Rpi‐vnt1.3*	100.00	21.38	0.11	8.65	9.16	0.00	7.36	8.68	7.03	6.84	0.00	0.00
*Virus‐Rx*	23.51	22.20	100.00	15.89	30.77	67.16	11.69	22.82	100.00	21.87	16.16	12.35

DRenSeq was conducted on 12 individual potato varieties. The name of the varieties and nucleotide‐binding, leucine‐rich‐repeat resistances (NLR) effective against diverse pathogens such as nematodes (*Nem*), late blight (*Rpi*) and viruses (*Virus*) are shown. The representation of individual resistance genes was calculated by extracting the sequence coverage of dRenSeq‐mapped reads to the reference coding DNA sequence (CDS). Highlighted in green are resistance genes that achieved 100% representation and are therefore classified as ‘present’.

Included in the 12 varieties as additional controls were seven varieties that have been assessed previously for the presence of known NLRs through pathogen assays, gene cloning and/or effector recognition studies (Table S2). In total, dRenSeq corroborated all 12 previously determined NLRs in the varieties Bionica, Cara, Craigs Snow White, Pentland Ace, Toluca and 2573 (2), which alone contained six known NLRs (Tables 3 and S2), which was in agreement with previous observations (Kim et al 2012). Interestingly, the variety Cara contains the nematode resistance gene *Gpa2* and the virus resistance *Rx*. This linkage of the 90% identical genes, *Rx* and *Gpa2*, in Cara has been described previously (van der Vossen *et al*., 2000) and the variety was used as a parent in the breeding of Picasso. Both Cara and Picasso share, in addition to *Gpa2* ^*ΔC2922*^ and *Rx,* the late blight resistance genes *Rpi‐R1, Rpi‐R3a, Rpi‐R3b* ^*G1696/G3111*^ (Table 3).

In Pentland Dell, which was released in 1960 as an early example of resistance gene stacking, the presence of late blight resistances *Rpi‐R1*,* Rpi‐R2* and *Rpi‐R3a* had been inferred through the use of differential pathogen isolates (Malcolmson 2009). DRenSeq confirmed the presence of *Rpi‐R1,* revealed that the *R2*‐specific response is based on the presence of the *R2‐*family member *Rpi‐abpt* ^*T86*^, and that both *R3a* and *R3b* ^*G1696/G3111*^ are contained in this variety.

Remarkably, dRenSeq identified the source of resistance in the variety Alouette, which featured on a national list as recently as 2015, as *Rpi‐vnt1.3* from *Solanum venturii* (Pel *et al*., 2009). As this is the first example for the deployment of this resistance gene in a commercial variety, the dRenSeq‐based inference of *Rpi‐vnt1.3* in Alouette was independently confirmed through (i) assessing *Avr‐vnt1* specific responses in a segregating F1 population and (ii) PCR‐based allele mining (Table S3; Figures S2 and S3).

To generate the segregating F1 population, Alouette was crossed with the susceptible variety Vitalia. In total, 75 progenies from this Al*Vi population were assessed in four replicates for late blight resistances and yielded a segregating population with 39 clear resistant and 36 clear susceptible genotypes. This 1 : 1 segregation ratio provides strong evidence that a single dominant resistant gene is responsible for the late blight resistance in the variety Alouette. From this segregating population 9 resistant and 8 susceptible clones where infiltrated with *Agrobacterium tumefaciens* transiently expressing five individual avirulence genes. Infiltrations were repeated in three plants using three leaves per plant. The scores for hypersensitive responses correlated with the observed disease resistance scores in that all resistant clones yielded an *Avr‐vnt1‐*specific response, while susceptible plants showed no response. None of the other *Avr* genes tested triggered an HR in any of the tested Al*Vi clones, showing that there was a very specific recognition of *Avr‐vnt1* in the resistant plants only (Table S3).

These data further demonstrate that dRenSeq can be applied to diverse genotypes and is not limited to specific varieties. Critically, where dRenSeq distinguishes different allelic variants, breeders have the opportunity to assess their performance prior to deployment. Functional analysis of allelic variants may distinguish stronger from weaker alleles, but alleles with altered recognition spectra may also be identified.

### dRenSeq‐based NLR identification is more cost effective than whole‐genome‐sequencing

**Figure 3:**
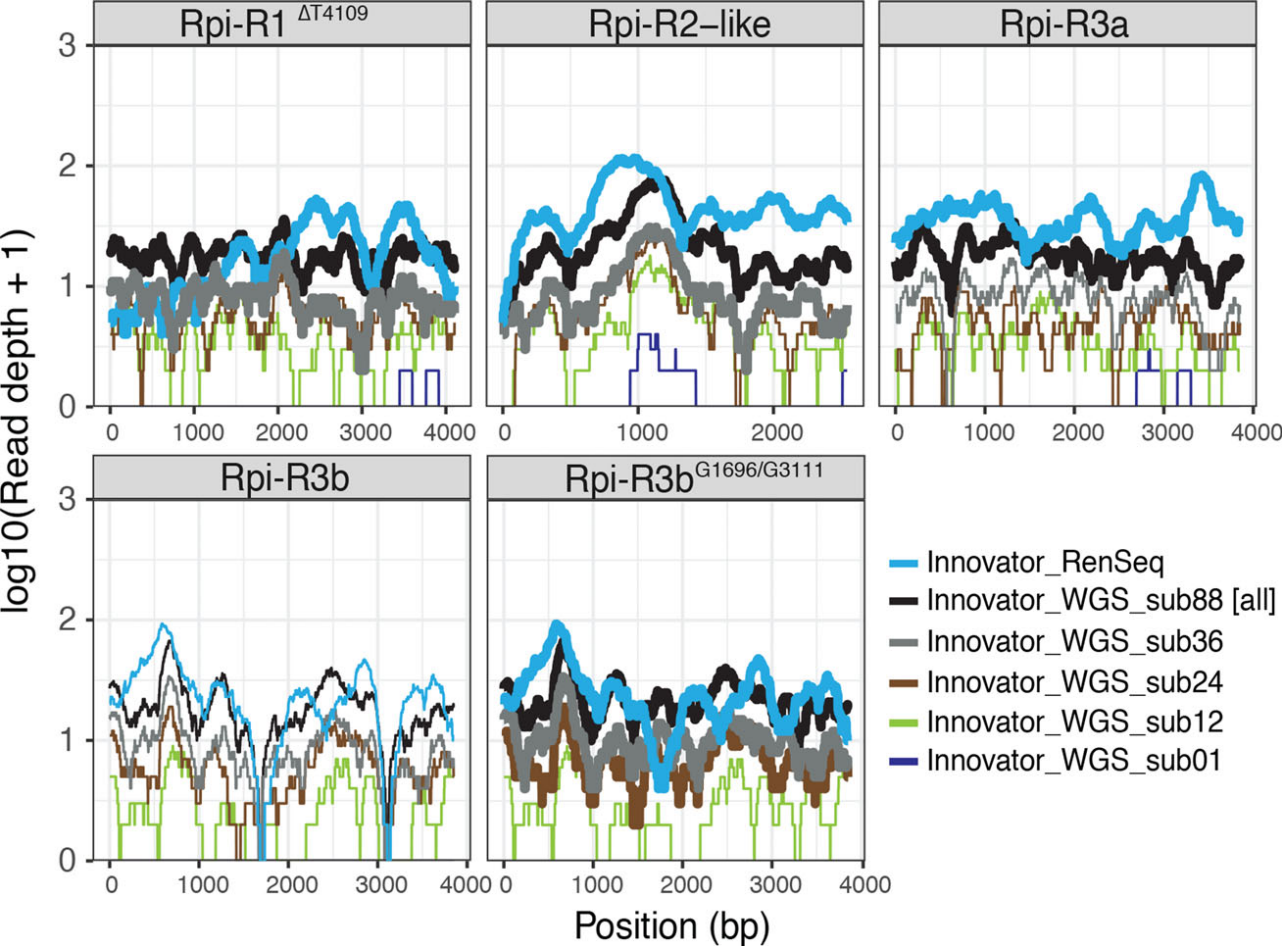
Comparison between dRenSeq and whole‐genome shotgun sequencing at different sampling depth for the potato variety Innovator. The sequence representation of NLRs identified in Innovator are shown in each box. The *x*‐axis depicts the coding sequence from start to stop and the *y*‐axis the read‐coverage on a log scale. Thick horizontal lines indicate full sequence representation without any sequence polymorphisms between the reference and the NLR enriched reads.

The efficiency of dRenSeq in identifying NLRs was tested in a direct comparison with whole‐genome sequencing (WGS) of the potato variety Innovator (Figure 3). RenSeq of Innovator yielded 1.7 million high‐quality MiSeq read pairs representing 778.6 Mbp of sequence data which was comparable among all enrichments conducted (Table S4). DRenSeq analysis revealed the presence of four NLRs; *Rpi‐R1* ^*ΔT4109*^, *Rpi‐R2‐like*,* Rpi‐R3a* and *Rpi‐R3b* ^*G1696/G3111*^ (Figure 3; Tables 3 and 4). It is interesting to note that a distant progenitor of Innovator, AM78‐3778, was the source of the molecular characterisation and cloning of *Rpi‐R2‐like* (Lokossou *et al*., 2009).

**Table 4:** NLR coverage in commercial potato variety Innovator following RenSeq and whole‐genome sequencing

	Innovator					
	RenSeq	Whole‐genome sequencing				
Total gigabase pairs	0.778	69.04	28.99	19.33	9.3	0.777
Gene name	RenSeq_all	sub88 [all]	sub36	sub24	sub12	sub01
*Rpi‐R1* ^*ΔT4109*^	100.00	100.00	100.00	99.34	89.76	7.36
*Rpi‐R2‐like*	100.00	100.00	100.00	95.13	93.12	20.48
*Rpi‐R3a*	100.00	100.00	98.29	97.04	91.22	11.33
*Rpi‐R3b*	97.72	99.22	97.30	96.96	71.63	0.00
*Rpi‐R3b* ^*G1696/G3111*^	100.00	100.00	100.00	100.00	83.15	0.00

DRenSeq was conducted on potato variety Innovator and compared to whole‐genome sequencing (WGS). For the comparison between RenSeq and WGS, subsamples of WGS reads were obtained. The sequence volume of WGS reads compared to RenSeq reads are shown in gigabases and x sequence volume [sub 01 =  equal amount to RenSeq; sub12 = 12× WGS compared to RenSeq; sub24 = 24× WGS compared to RenSeq; sub36 = 36× WGS compared to RenSeq; sub88 = 88× WGS compared to RenSeq (in this case all WGS data). The IDs of the Resistance to *Phytophthora infestans (Rpi)* nucleotide‐binding, leucine‐rich‐repeat resistances are shown. The representation of individual, full‐length *Rpi* genes was calculated by extracting the sequence coverage of dRenSeq‐mapped reads to the reference coding DNA sequence (CDS). WGS reads were mapped under the same stringent mapping condition used for dRenSeq. Highlighted in green are *Rpi* genes that achieved 100% representation and are therefore classified as ‘present’.

Whole‐genome sequencing (WGS) of Innovator yielded 228 million high‐quality Illumina NextSeq read pairs representing 69 000 Mbp of sequence data and thereby over 88× the sequence volume of dRenSeq (Table S4). After subsampling 778.6 Mbp WGS sequences to obtain equal sequence representation compared to RenSeq as well as 12× more WGS sequences, no known NLRs in Innovator were identified with 100% coverage (Table 4). Increasing WGS coverage to 24× compared to dRenSeq only identified *Rpi‐R3b* ^*G1696/G3111*^ *,* and 36× identified full length *Rpi‐R1* ^*ΔT4109*^ *, Rpi‐R2‐like and Rpi‐R3b* ^*G1696/G3111*^ but not *Rpi‐R3a*. The NLR *Rpi‐R3a* was detected only when all WGS reads were mapped against the references. Importantly, WGS independently confirmed the sequence polymorphisms in *Rpi‐R3b* ^*G1696/G3111*^ identified by dRenSeq (Figure 3; Figure S4a,b). Since we routinely combine 12 genomic DNA samples per dRenSeq analysis in a single Illumina MiSeq flow‐cell, this demonstrates that RenSeq‐based genome reduction is considerably more robust and cost‐effective in detecting NLRs than WGS.

## Discussion

Here we demonstrate that dRenSeq enables the parallel identification and sequence validation of multiple functional resistance genes effective against different pathogens. It is currently the only available tool to cost‐effectively analyse multiple genotypes in crop breeding programs, identify germ plasm with redundant NLRs, and to confirm transgene integrity in commercially available GM crops. The methodology can easily be adapted to include additional functional NLRs, as and when they become available, by ensuring sufficient representation of new genes within the bait library utilised.

A direct comparison between dRenSeq and non‐enriched whole‐genome sequencing highlights the advantages of dRenSeq. Compared to WGS, only a fraction of the reads is required after NB‐LRR gene enrichment to identify and confirm the presence of functional disease resistance genes.

As shown for transgenic Desiree and in other varieties, the sensitivity of dRenSeq, which is achieved through enrichment‐based deep‐sequencing, is sufficient to determine single sequence polymorphisms in NLRs. This is a prerequisite to certify deployment of functional genes rather than pseudogenised or less effective variants. PCR‐based tests are typically unable to identify such variations without sequencing multiple cloned products (Van Weymers *et al*., 2016). Indeed, as recently shown for *R8*, positive PCR amplification does not necessary indicate the presence of functional NLRs as pseudogenised genes can also yield products of similar size (Jiang *et al*., 2018). Therefore, it is often necessary to Sanger sequence PCR products of NLRs, which typically requires the cloning of PCR products and sequencing of recombinant clones. As the average coding sequence NLR size in potato is estimated to be around 2.7 kb (Jupe *et al*., 2012), at least four Sanger sequencing reactions are required for the analysis of each recombinant clone. Furthermore, as shown for *Rpi‐vnt1* in *S. okadae*, numerous recombinants need to be analysed to achieve a true representation of diverse haplotypes (Van Weymers *et al*., 2016). Presuming that the sequencing of five recombinant clones would be sufficient to determine presence/absence and the sequence identity of functional genes, more than 380 Sanger sequencing reactions would be required per single potato variety to assess the 19 genes (Table S5) that were tested in parallel through dRenSeq (5 clones × 4 sequencing reactions × 19 genes). Since dRenSeq enables the simultaneous analysis and sequence validations of all genes in 12 varieties, the equivalent number of Sanger sequencing reaction would be at least 4560. Considering the costs and time investments for the PCR analysis and uncertainties in amplifying functional genes from a background of endogenous homologues, dRenSeq represents a cost‐effective alternative. Furthermore, a complete dRenSeq analysis from DNA isolation to the computational analysis can be achieved in 7 days with little hands on time being required, which again contrasts with the PCR‐based alternative detailed above.

Similarly, effector recognition studies are dependent on the discovery of cognate avirulence genes which are not available for all NLRs, are limited by suitable expression systems that work in all host genotypes, and can suffer from a lack of specific plant responses (Vleeshouwers *et al*., 2011). For example, the presence of *Rx* in some varieties including Cara and Picasso precludes the use of the often preferred PVX Agroinfection assay (Du *et al*., 2014). Similarly, as shown for the cross between Alouette and Vitalia, some plants are rather recalcitrant to *A. tumefaciens*‐based effector delivery (Table S3). The observed responses to *Avr‐vnt1* and/or *Avr8* co‐infiltration with *Rpi‐R8* in the cross revealed a spectrum of responsiveness that was measured on a score from 0 to 2. Therefore, it is necessary to conduct multiple independent inoculations to obtain reproducible results. In addition, these assays typically need special glasshouses, licences and risk assessments.

The ability to track NLRs in crops through dRenSeq is a major advance for modern molecular crop breeding. The methodology can inform on: (i) germ plasm pedigrees; (ii) complementary sources for NLR stacking; (iii) the historic deployment of resistances; (iv) the geographical differences in NLR deployments. The latter is a prerequisite to gain an insight into the local adaption of pathogens. Indeed, dRenSeq enables the establishment of a reference for already deployed resistance in existing varieties and therefore identify suitable sources of complementary resistance in pre‐breeding sources. Thereby, dRenSeq can direct parental selection as well as progeny ranking in crop breeding programs that aim to combine multiple resistances where differential pathogen isolates may not be able to confirm the functionality of the resistances in combination. This could be established for any crop where NLRs control diseases. A prime example is the potential application of a bespoke dRenSeq approach to help control downy mildew of lettuce (*Bremia lactucae*), where a number of functional resistances have been identified already and which could be efficiently combined.

The insight into deployment of NLRs is also relevant for future resistance management strategies. Sensible deployment regulations are required to ensure the durability of resistance in the long run. The dRenSeq application provides a key diagnostic tool to unambiguously determine the NLR contents of new varieties, from which farmers, seed producers, and phytosanitary regulators can make decisions. In the future, similar methodologies could also be adapted for other gene families that control disease including receptor‐like kinases and receptor‐like proteins once more functional genes have been isolated.

## Material and methods

### Illumina library preparation

Genomic DNA was extracted from fresh leaf material using the Qiagen DNeasy Plant Mini Kit (Qiagen, Hilden, Germany). The Covaris M220 sonicator (Covaris, Woburn), was used for the fragmentation of DNA to approximately 500 bp in length, with the following settings: 50 W Peak Incident Power, 20% Duty Factor, 200 cycles per burst, 60 seconds treatment time for a 50 μL volume with 1 μg of starting DNA material. The fragments sizes were checked using a Bioanalyser (Agilent, Santa Clara) and no size selection was conducted. Indexed libraries were prepared using the NEBNext library preparation kit for Illumina (NEB, Ipswich). Multiple rounds of AMPure XP bead purification (Beckman Coulter, Brea) at a 1 : 1 ratio of beads to sample was used during the protocol to eliminate fragments smaller than 250 bp in length.

### Targeted enrichment

The genomic DNA libraries were quantified fluorometrically using Qubit (Thermofisher, Waltham) and 12 indexed libraries were typically pooled prior to enrichment at equimolar amounts so as to achieve 750 ng of starting material. The bespoke NB‐LRR specific enrichment probe set was purchased from MYcroarray‐MYbaits and was based on an improved probe set design described previously (Van Weymers *et al*., 2016). To capture the entire coding DNA sequence (CDS) of known NLRs in the dRenSeq method, the probe set was expanded to include more baits over the individual CDS entireties. The increased bait library [RenSeq library version 4] has been uploaded to http://solanum.hutton.ac.uk. The enrichment protocol was essentially as described for the SureSelect target enrichment system (Agilent, Santa Clara) except that human Cot‐1 and salmon sperm DNA were omitted from the blocking mix and were replaced by NimbleGen SeqCap EZ Developer Reagent (Roche, Basel, Switzerland). Additionally, the blocking mix was supplemented with 1 μL of 1000 mm universal blocking oligo, containing six inosines in place of the six nucleotide index sequence and a 3′ spacer C3 modification to prevent the oligo from participating in any subsequent PCR amplification. The post capture amplification was performed with the Herculase II polymerase (Agilent, Santa Clara). Sequencing was conducted on an Illumina MiSeq platform using the v.2 reagent kit and 2 × 250 bp conditions.

### Computational analysis

Illumina reads were trimmed with cutadapt (Martin, 2011) version 1.9.1 to a minimum length of 100 bp with a minimum phred quality score of 20 using settings anticipating 3′ anchored adapters. The trimmed reads were mapped to the reference NLRs including 5′ and 3′ flanking regions (Appendix S1 and S2, Table S5) using bowtie2 (Langmead and Salzberg, 2012) version 2.2.1 in very‐sensitive end‐to‐end mode with discordant read mappings disabled and a maximum insert size of 1000 bp. Typically, the score‐min parameter was set at L, −0.01,−0.01 which results in a miss‐match penalty of five for a 250 bp read pair. Consequently, only reads identical to the reference were mapped, except reads containing a variant nucleotide with a quality score <30. For variant discovery, the score‐min parameter was set at L, −0.03,−0.03 thus allowing a single high‐quality SNP per read pair or a miss‐match rate of ~0.5%. Due to the synthetic nature of the reference and the high nucleotide similarity of some of the sequences within it, up to 10 mapping positions per read pair were allowed (‐k 10). The resulting bam alignments were sorted and indexed using samtools (Li *et al*., 2009) v1.3.1.

Innovator WGS reads were generated by Illumina NextSeq sequencing and were subsampled using seqtk (https://github.com/lh3/seqtk). The read mapping was performed as described above. After mapping, the read coverage and depth of coverage for each reference NLR gene was calculated at each position between the first and last nucleotide of the start and stop codons (Appendix S2) using Bedtools (version 2.25.0) coverage (Quinlan and Hall, 2010). Read depth was log10 transformed and plotted against position using R studio (R Studio Team, 2015) (v1.0.143) and ggplot2. All reads have been submitted to the European Nucleotide Archive (https://www.ebi.ac.uk/ena) with the ENA accession number ERP105478.

### Segregation of late blight resistance in Alouette*Vitalia population

A cross was made between the late blight resistant potato variety Alouette and the susceptible variety Vitalia. Seeds from this Al*Vi population were sown *in vitro* and 75 germinated seedlings were maintained *in vitro*. Four copies of each genotype were propagated and planted in the late blight trial field in Wageningen. Approximately 1 month after planting, the field was spray inoculated with a spore suspension of complex *P. infestans* isolate IPO‐C. From the middle of July disease progress was scored at 4–10 days intervals (Table S3). An obvious distinction could be made between 39 resistant and 36 susceptible genotypes.

### Agroinfiltration in resistant and susceptible clones of Al*Vi population

Agroinfiltration with *Avr* genes was performed as previously described (Rietman *et al*., 2012). In total 17 randomly selected F1 progeny clones (8 susceptible and 9 resistant) from the Al*Vi population (Table S3) where infiltrated with *Agrobacterium tumefaciens* transiently expressing five individual avirulence genes. Infiltrations were repeated in three plants using three leaves per plant. The scores for hypersensitive responses were according to Rietman *et al*. (2012) ranging from 0% to 100% cell death, which was converted to a 0–2 scale representing no‐ to complete cell death in the agroinfiltrated area.

### PCR analysis in resistant and susceptible clones of Al*Vi population

To ascertain which *Rpi‐vnt1* gene was responsible for the late blight resistance in Alouette, we conducted PCR reactions with primers LK69 (5′AGCATTGGCCCAATTATCATTAAC3′) and LK70 (5′ATGAATTATTGTGTTTACAAGACTTG3′) on selected clones from the Al*Vi population. The amplification yielded a 1100 bp *Rpi‐vnt1*‐specific amplicon and which was subsequently Sanger sequenced.

## Competing interest

The authors declare competing financial interests: BBSRC Industrial Partnership Awards BB/L008025/1 and BB/K018299/1 awarded to I.H. involve US company JR Simplot.

## Author contributions

I.H., M.R.A., E.M.G, N.C and J.V. conceived the study and wrote the manuscript. M.R.A., S.M.S. and B.H. performed the research. J.V. and R.C.B.H. produced and provided plant material. J.X. performed experiments to show the presence of *Rpi‐vnt1.3* in potato variety Alouette. N.C. characterised Innate varieties. T.Y.L and M.R.A. conducted the data analysis. All authors read and approved the manuscript.

## Data availability

All NLR enriched sequence information has been deposited at the European Nucleotide Archive (https://www.ebi.ac.uk/ena) with the ENA accession number ERP105478. The data will be made publicly available upon publication of the manuscript.

## Supporting information


**Figure S1.** (a) Sequence polymorphisms are reliably identified with dRenSeq: Example *Rpi‐pta1* in transgenic Desiree line A23‐29. (b) Sequence polymorphisms are reliably identified with dRenSeq: Example *Rpi‐sto1* in transgenic Desiree line A14‐81. (c) Sequence polymorphisms are reliably identified with dRenSeq: Example *Rpi‐vnt1.1* in transgenic Desiree line A23‐29.
**Figure S2.** A F1 population derived from a cross between varieties Alouette × Vitalia segregates for recognition of *Avr‐vnt1*.
**Figure S3.** *Rpi‐vnt1* PCR analysis in the Al*Vi population.
**Figure S4.** (a) Sequence polymorphisms are reliably identified with dRenSeq: Example *Rpi‐R3b* in potato variety Innovator. (b) Sequence polymorphisms identified by dRenSeq are also found by whole‐genome sequencing (WGS): Example *Rpi‐R3b* in potato variety Innovator.
**Table S1.** Sequence variations identified in resistance genes.
**Table S2.** Previously characterised potato varieties/pre‐breeding clones confirmed by dRenSeq analysis.
**Table S3.** A F1 population derived from varieties Alouette × Vitalia segregates for *Rpi‐vnt1.3*.
**Table S4.** Illumina sequencing statistics. Shown are the total number of RenSeq enriched and Illumina MiSeq (2 × 250 bp) generated reads.
**Table S5.** NLR references. Shown are the gene names, the GenBank ID, and the reference detailing the molecular characterisation of the resistances.
**Appendix S1.** FASTA sequence of all reference NLRs used including their 5′ and 3′ flanking region.
**Appendix S2.** Coordinates of the reference NLR CDS (start–stop).
