# Supplementary material for: Tracking disease resistance deployment in potato breeding by enrichment sequencing
Source: Plant Biotechnol J. 2018 Sep 19;17(2):540–9. doi: 10.1111/pbi.12997 (PMC6335062; doi:10.1111/pbi.12997)
Supplement: Supplementary file 1 — Figure S1. (a) Sequence polymorphisms are reliably identified with dRenSeq: Example Rpi‐pta1 in transgenic Desiree line A23‐29. (b) Sequence polymorphisms are reliably identified with dRenSeq: Example Rpi‐sto1 in transgenic Desiree line A14‐81. (c) Sequence polymorphisms are reliably identified with dRenSeq: Example Rpi‐vnt1.1 in transgenic Desiree line A23‐29. Figure S2. A F1 population derived from a cross between varieties Alouette × Vitalia segregates for recognition of Avr‐vnt1. Figure S3. Rpi‐vnt1 PCR analysis in the Al*Vi population. Figure S4. (a) Sequence polymorphisms are reliably identified with dRenSeq: Example Rpi‐R3b in potato variety Innovator. (b) Sequence polymorphisms identified by dRenSeq are also found by whole‐genome sequencing (WGS): Example Rpi‐R3b in potato variety Innovator. Table S1. Sequence variations identified in resistance genes. Table S2. Previously characterised potato varieties/pre‐breeding clones confirmed by dRenSeq analysis. Table S3. A F1 population derived from varieties Alouette × Vitalia segregates for Rpi‐vnt1.3. Table S4. Illumina sequencing statistics. Shown are the total number of RenSeq enriched and Illumina MiSeq (2 × 250 bp) generated reads. Table S5. NLR references. Shown are the gene names, the GenBank ID, and the reference detailing the molecular characterisation of the resistances. Appendix S1. FASTA sequence of all reference NLRs used including their 5′ and 3′ flanking region. Appendix S2. Coordinates of the reference NLR CDS (start–stop). [file PBI-17-540-s001.docx]

**Supplementary Table 1: Sequence variations identified in resistance genes**

| **Gene** | **Pos.** | **ATG+** | **Ref.** | **Var.** | **Codon** | **Class** | **Amino acid** |
| --- | --- | --- | --- | --- | --- | --- | --- |
| ***Transgenic Desiree plants*** |  |  |  |  |  |  |  |
| *Rpi-pta1^A2012^* | 4280 | 2012 | G | A | GTG/GTA | S | Val/Val |
| *Rpi-sto1^T3144^* | 5394 | 3144 | C | T | CAC/CAT | S | His/His |
| *Rpi-vnt1.1^A2056^* | 2766 | 2056 | G | A | CGA/CAA | NS | Arg/Gln |
|  |  |  |  |  |  |  |  |
| ***Varieties*** |  |  |  |  |  |  |  |
| *Rpi-R1^ΔT4109^* | 5171 | 4109 | T | - del |  | 3' UTR | / |
| *Rpi-R3b^G1696/G3111^* | 1947 | 1696 | A | G | CAT/CGT | NS | His/Arg |
|  | 3362 | 3111 | A | G | AAG/GAG | NS | Lys/Glu |
| *Rpi-R3b^G3111^* | 3362 | 3111 | A | G | AAG/GAG | NS | Lys/Glu |
| *Rpi-abpt^T86^* | 337 | 86 | C | T | GAC/GAT | S | Asp/Asp |
| *Nem-Gpa2^ΔC2922^* | 7798 | 2922 | C | - del |  | intron | / |

Sequence variations in reference genes where identified following dRenSeq mapping of paired-end reads to NLR reference sequences (Appendix 1). Paired-end read mapping was conducted at a 0% mismatch rate, not allowing for any sequence polymorphisms and at a 0.5% mismatch rate, effectively allowing for one sequence polymorphism in 200 bp of sequence. The position of sequence polymorphisms relative to the reference sequence (including flanking sequences) is shown (Pos.). The same polymorphisms are shown in relation to the first nucleotide of the start codon (ATG+) for the coding DNA sequence (CDS) (Appendix 2). The published reference (Ref.) is compared to the variation identified (Var.) Variations include nucleotide substitutions and deletions (-del). If applicable, the nucleotide substitution is shown in the form of a codon change (Codon) and the nature of the change is indicated (NS: non-synonymous; S: synonymous). The resulting amino acid substitution, where applicable, is highlighted.

**Supplementary Table 2: Previously characterised potato varieties/pre-breeding clones confirmed by dRenSeq analysis.**

| **Variety** | **Pred. NB-LRR** | **Ref.** | **Confirmed NB-LRRs** |
| --- | --- | --- | --- |
| Bionica | *Rpi-blb2* | (Haverkort *et al.*, 2009) | *Rpi-R3a, Rpi-R3b^G1696/G3111^, Rpi-abpt^T86^, Rpi-blb2* |
| Cara | *Nem-Gpa2, Virus-Rx* | (van der Vossen *et al.*, 2000) | *Nem-Gpa2^ΔC2922^, Rpi-R1, Rpi-R1^ΔT4109^, R3a, Rpi-R3b^G1696/G3111^, Virus-Rx* |
| Craigs Snow White | *Rpi-R1* | (Stewart and Bradshaw, 2001) | *Rpi-R1* |
| Pentland Ace | *Rpi-R3a* | (Black *et al.*, 1953; Stewart and Bradshaw, 2001) | *Rpi-R3a, Rpi-R3b^G1696/G3111^* |
| Pentland Dell^*^ | *Rpi-R1, Rpi-R2, Rpi-R3* | (Malcolmson, 1969) | *Rpi-R1, Rpi-abpt, Rpi-R3a*, R*pi-R3b^G3111^ Rpi-R3b^G1696/G3111^* |
| Toluca | *Rpi-blb2* | (Haverkort *et al.*, 2009) | *Rpi-blb2* |
| 2573(2)^**^ | *Rpi-R1*  *Rpi-abpt*  *Rpi-R3a, Rpi-R3b, Rpi-R8, Rpi-R9a* | (Black *et al.*, 1953) | *Rpi-R1, Rpi-R3a, Rpi-R3b^G1696/G3111^, Rpi-R8*, *Rpi-R9a*, *Rpi-abpt^T86^* |

Detailed are the names of the potato varieties analysed in this study and used as controls. Previous studies in these varieties have identified NB-LRR (Pred. NB-LRR) which were confirmed by dRenSeq (Confirmed NB-LRRs). *Pentland Dell has been described to contain *Rpi-R1, Rpi-R2* and *Rpi-R3* (Malcolmson, 1969). It is now known that *Rpi-abpt* is an *R2* family member (Lokossou *et al.*, 2009) and the differentiation of *Rpi-R3* into *Rpi-R3a/R3b* occurred later than the resistance study of Pentland Dell. **This plant was never registered as a cultivar. Hybrid seedlings from crosses between *S. demissum* and *S. tuberosum* were selected from Pentland Field in 1937. An unknown number of backcrosses to *S. tuberosum* resulted in the clone 2573(2). This plant is used as a late blight differential and pre-breeding clone due to the combination of resistances.

**Supplementary Table 3: A F1 population derived from varieties Alouette x Vitalia segregates for *Rpi-vnt1.3***

| **Genotype** | **Average percentage of blighted foliage after inoculation with IPO-C*** | | | | | | **Average effector response **** | | |
| --- | --- | --- | --- | --- | --- | --- | --- | --- | --- |
|  | 20-jul | 24-jul | 31-jul | 10-aug | 18-aug | **conclusion** | *Avr8/*  *Rpi-R8* | *Avr-*  *vnt1* | **conclusion** |
| Alouette | 0 | 0 | 0 | 0 | 5 | R |  |  |  |
| Vitalia | 10 | 30 | 100 | 100 | 100 | S |  |  |  |
| Al*Vi-1 | 10 | 60 | 100 | 100 | 100 | S |  |  |  |
| Al*Vi-2 | 20 | 60 | 90 | 100 | 100 | S |  |  |  |
| Al*Vi-3 | 20 | 70 | 100 | 100 | 100 | S |  |  |  |
| Al*Vi-4 | 0 | 0 | 0 | 0 | 0 | R |  |  |  |
| Al*Vi-5 | 0 | 0 | 0 | 0 | 0 | R |  |  |  |
| Al*Vi-6 | 0 | 0 | 0 | 0 | 5 | R |  |  |  |
| Al*Vi-7 | 0 | 0 | 0 | 0 | 5 | R |  |  |  |
| Al*Vi-8 | 10 | 70 | 100 | 100 | 100 | S |  |  |  |
| Al*Vi-9 | 10 | 60 | 100 | 100 | 100 | S |  |  |  |
| Al*Vi-10 | 30 | 80 | 100 | 100 | 100 | S |  |  |  |
| Al*Vi-11 | 20 | 70 | 100 | 100 | 100 | S |  |  |  |
| Al*Vi-12 | 30 | 80 | 100 | 100 | 100 | S |  |  |  |
| Al*Vi-13 | 5 | 50 | 100 | 100 | 100 | S |  |  |  |
| Al*Vi-14 | 5 | 30 | 100 | 100 | 100 | S |  |  |  |
| Al*Vi-15 | 0 | 0 | 0 | 0 | 5 | R |  |  |  |
| Al*Vi-16 | 0 | 0 | 0 | 0 | 5 | R |  |  |  |
| Al*Vi-17 | 0 | 0 | 0 | 0 | 5 | R |  |  |  |
| Al*Vi-19 | 0 | 1 | 0 | 0 | 20 | R |  |  |  |
| Al*Vi-20 | 0 | 0 | 0 | 0 | 5 | R |  |  |  |
| Al*Vi-22 | 0 | 0 | 0 | 0 | 5 | R |  |  |  |
| Al*Vi-23 | 0 | 0 | 0 | 0 | 5 | R |  |  |  |
| Al*Vi-24 | 20 | 40 | 100 | 100 | 100 | S |  |  |  |
| Al*Vi-25 | 10 | 30 | 100 | 100 | 100 | S |  |  |  |
| Al*Vi-26 | 10 | 90 | 100 | 100 | 100 | S |  |  |  |
| Al*Vi-27 | 0 | 0 | 0 | 0 | 20 | R |  |  |  |
| Al*Vi-28 | 20 | 50 | 100 | 100 | 100 | S |  |  |  |
| Al*Vi-29 | 0 | 0 | 0 | 0 | 30 | R |  |  |  |
| Al*Vi-30 | 0 | 0 | 0 | 0 | 0 | R |  |  |  |
| Al*Vi-31 | 10 | 50 | 90 | 90 | 100 | S | 1,57 | 0 | n |
| Al*Vi-32 | 10 | 50 | 100 | 100 | 100 | S |  |  |  |
| Al*Vi-33 | 0 | 0 | 0 | 0 | 10 | R |  |  |  |
| Al*Vi-34 | 10 | 40 | 95 | 100 | 100 | S |  |  |  |
| Al*Vi-35 | 10 | 50 | 90 | 95 | 100 | S |  |  |  |
| Al*Vi-36 | 0 | 0 | 0 | 0 | 10 | R | 0,875 | 2 | r |
| Al*Vi-37 | 3 | 20 | 95 | 95 | 100 | S | 1,2 | 0 | n |
| Al*Vi-38 | 0 | 0 | 0 | 0 | 5 | R | 1,8 | 2 | r |
| Al*Vi-39 | 0 | 10 | 100 | 100 | 100 | S |  |  |  |
| Al*Vi-40 | 1 | 10 | 100 | 100 | 100 | S |  |  |  |
| Al*Vi-41 | 0 | 5 | 50 | 100 | 100 | S | 1,6 | 0 | n |
|  |  |  |  |  |  |  |  |  |  |
| Al*Vi-42 | 0 | 2 | 100 | 100 | 100 | S |  |  |  |
| Al*Vi-43 | 0 | 2 | 60 | 100 | 100 | S |  |  |  |
| Al*Vi-44 | 0 | 0 | 0 | 0 | 5 | R | 1,67 | 2 | r |
| Al*Vi-45 | 0 | 0 | 0 | 0 | 1 | R |  |  |  |
| Al*Vi-46 | 0 | 5 | 50 | 100 | 100 | S | 1 | 0 | n |
| Al*Vi-47 | 0 | 0 | 0 | 0 | 5 | R | 1,25 | 2 | r |
| Al*Vi-48 | 0 | 0 | 0 | 0 | 5 | R |  |  |  |
| Al*Vi-49 | 0 | 0 | 0 | 0 | 10 | R | 1 | 2 | r |
| Al*Vi-50 | 0 | 0 | 0 | 0 | 5 | R |  |  |  |
| Al*Vi-51 | 0 | 5 | 100 | 100 | 100 | S |  |  |  |
| Al*Vi-52 | 0 | 0 | 0 | 0 | 5 | R |  |  |  |
| Al*Vi-53 | 0 | 0 | 0 | 0 | 0 | R |  |  |  |
| Al*Vi-54 | 0 | 0 | 0 | 0 | 0 | R |  |  |  |
| Al*Vi-55 | 0 | 0 | 0 | 0 | 5 | R |  |  |  |
| Al*Vi-56 | 0 | 2 | 90 | 100 | 100 | S | 1 | 0,166666667 | n |
| Al*Vi-57 | 0 | 0 | 0 | 0 | 5 | R | 1,125 | 2 | r |
| Al*Vi-58 | 0 | 0 | 0 | 0 | 10 | R |  |  |  |
| Al*Vi-59 | 0 | 0 | 0 | 0 | 0 | R | 0,6 | 1,6 | r |
| Al*Vi-60 | 0 | 0 | 0 | 0 | 5 | R |  |  |  |
| Al*Vi-62 | 0 | 0 | 0 | 0 | 0 | R |  |  |  |
| Al*Vi-63 | 0 | 2 | 70 | 100 | 100 | S |  |  |  |
| Al*Vi-64 | 0 | 0 | 0 | 0 | 1 | R | 0,97 | 1,8 | r |
| Al*Vi-65 | 1 | 10 | 70 | 100 | 100 | S | 1,5 | 0 | n |
| Al*Vi-66 | 0 | 0 | 0 | 0 | 0 | R |  |  |  |
| Al*Vi-67 | 1 | 1 | 0 | 0 | 5 | R | 0,75 | 1 | r |
| Al*Vi-68 | 10 | 20 | 100 | 100 | 100 | S |  |  |  |
| Al*Vi-69 | 10 | 30 | 100 | 100 | 100 | S |  |  |  |
| Al*Vi-70 | 3 | 10 | 90 | 100 | 95 | S |  |  |  |
| Al*Vi-71 | 10 | 30 | 90 | 100 | 100 | S |  |  |  |
| Al*Vi-72 | 20 | 60 | 100 | 100 | 100 | S |  |  |  |
| Al*Vi-73 | 10 | 60 | 100 | 100 | 100 | S | 1,25 | 0 | n |
| Al*Vi-74 | 0 | 0 | 0 | 0 | 5 | R |  |  |  |
| Al*Vi-75 | 10 | 50 | 100 | 100 | 100 | S | 1,65 | 0 | n |
| Al*Vi-76 | 0 | 0 | 0 | 0 | 5 | R |  |  |  |
| Al*Vi-77 | 0 | 0 | 0 | 0 | 10 | R |  |  |  |
| Al*Vi-78 | 0 | 0 | 0 | 0 | 5 | R |  |  |  |

Summary of phenotypic and genotypic data for the segregating population derived from crossing resistant potato variety Alouette with susceptible Vitalia.

* Disease severity scores in field trial represents the average percentage of blighted foliage (n=4 repeats). S: genotype was found to be susceptible. R: plant was found to be resistant.

** The scores for hypersensitive response were on a scale from 0-2 representing no- to complete cell death in the agroinfiltrated area. r: plant was found to be responsive to Avr-vnt1. n: genotype was found to be non-responsive to Avr-vnt1. Green and yellow highlighted cells represent resistant and susceptible phenotypes, respectively, among plants that have been subjected to the late blight and effector response

**Supplementary Table 4: Illumina sequencing statistics**

| **RenSeq reads transgenic plants** | | | |  |  |
| --- | --- | --- | --- | --- | --- |
| **Sample** | **Reads** | **Read1 (bp)** | **Read2 (bp)** | **Total (bp)** |  |
| A01-20 | 1142492 | 276930502 | 276331929 | 553262431 |  |
| A02-33 | 712486 | 175264911 | 174432154 | 349697065 |  |
| A04-33 | 775450 | 189222801 | 188696777 | 377919578 |  |
| A106-15 | 1060340 | 257013973 | 256328625 | 513342598 |  |
| A14-81 | 1351401 | 330019972 | 329135978 | 659155950 |  |
| A23-29 | 864160 | 211575120 | 210962282 | 422537402 |  |
| A25-11 | 970722 | 237410573 | 236670684 | 474081257 |  |
| A31-47 | 819470 | 201233034 | 200549036 | 401782070 |  |
| A56-27 | 1072174 | 262054107 | 261269878 | 523323985 |  |
| A73-1-11 | 1309340 | 317002105 | 316283742 | 633285847 |  |
| A74-8-14 | 1110198 | 259039117 | 259011833 | 518050950 |  |
|  |  |  |  |  |  |
| **RenSeq reads in Innate® transgenics lines and progenitors** | | | | | |
| **Sample** | **Reads** | **Read1 (bp)** | **Read2 (bp)** | **Total (bp)** |  |
| Ranger Russet | 1253558 | 298357872 | 296652087 | 595009959 |  |
| Acclimate | 1633264 | 386741118 | 383709650 | 770450768 |  |
| Russet Burbank | 1464237 | 352176117 | 351839141 | 704015258 |  |
| Glaciate | 1349292 | 323096789 | 322121478 | 645218267 |  |
| Atlantic | 1753963 | 411214307 | 407923832 | 819138139 |  |
| Hibernate | 1857850 | 444709101 | 442644460 | 887353561 |  |
|  |  |  |  |  |  |
| **RenSeq reads in 12 varieties** | | | | | |
| **Sample** | **Reads** | **Read1 (bp)** | **Read2 (bp)** | **Total (bp)** |  |
| Alouette | 2043900 | 500664590 | 469511689 | 970176279 |  |
| Bionica | 2026261 | 469001053 | 444412356 | 913413409 |  |
| Cara | 1391101 | 331273048 | 331316786 | 662589834 |  |
| Craigs Snow White | 2640588 | 615238162 | 601817300 | 1217055462 |  |
| Innovator | 1702803 | 394379540 | 384196202 | 778575742 |  |
| King Edward | 1140995 | 260143532 | 258485807 | 518629339 |  |
| Pentland Ace | 1177182 | 279866932 | 279811797 | 559678729 |  |
| Pentland Dell | 1616407 | 367968151 | 366351473 | 734319624 |  |
| Picasso | 2606068 | 623216607 | 616110092 | 1239326699 |  |
| Spunta | 1398370 | 333511233 | 305225476 | 638736709 |  |
| Toluca | 1277527 | 305438752 | 305225476 | 610664228 |  |
| 2573(2) | 984069 | 234379871 | 234351184 | 468731055 |  |
|  |  |  |  |  |  |
| **RenSeq vs WGS in Innovator** | | | | | |
| **Sample** | **Reads** | **Read1 (bp)** | **Read2 (bp)** | **Total (bp)** | **x dRenSeq** |
| Innovator_WGS_sub88 [all] | 228615259 | 34520904109 | 34520904109 | 69041808218 | **88.67706055** |
| Innovator_WGS_sub36 | 96015121 | 14498283271 | 14498283271 | 28996566542 | **37.24309014** |
| Innovator_WGS_sub24 | 64009990 | 9665508490 | 9665508490 | 19331016980 | **24.82869159** |
| Innovator_WGS_sub12 | 31068775 | 4675444323 | 4626834988 | 9302279311 | **11.94781549** |
| Innovator_WGS_sub01 | 2595834 | 390635821 | 386576332 | 777212153 | **0.998248611** |

Shown are the total number of RenSeq enriched and Illumina MiSeq (2x250 bp) generated reads. The sequencing volume in base pairs (bp) is shown for paired-end reads 1 and 2 as well as for the combined sequencing volume. For the comparison between RenSeq and whole-genome sequencing (WGS), subsamples of WGS reads were attained. The relative sequence volume of WGS reads compared to RenSeq reads are shown (x dRenSeq).

**Supplementary Table 5: NLR references**

| **Gene** | **GenBank** | **Reference** |
| --- | --- | --- |
| *R1* | GenBank: AF447489.1 | (Ballvora *et al.*, 2002) |
| *R2* | GenBank: FJ536325.1 | (Lokossou *et al.*, 2009) |
| *R2-like* | GenBank: FJ536323.1 | (Lokossou *et al.*, 2009) |
| *R3a* | GenBank: AY849382.1 | (Huang *et al.*, 2005) |
| *R3b* | GenBank: JF900492.1 | (Li *et al.*, 2011) |
| *Rpi-sto1* | GenBank: EU884421.1 | (Vleeshouwers *et al.*, 2008) |
| *Rpi-pta1* | GenBank: EU884422.1 | (Vleeshouwers *et al.*, 2008) |
| *Rpi- blb1* | GenBank: AY426259.1 | (Van Der Vossen *et al.*, 2003) |
| *Rpi-blb2* | GenBank: DQ122125.1 | (Van Der Vossen *et al.*, 2005) |
| *Rpi-blb3* | GenBank: FJ536346.1 | (Lokossou *et al.*, 2009) |
| *Rpi-abpt* | GenBank: FJ536324.1 | (Lokossou *et al.*, 2009) |
| *Rpi-vnt1.1* | GenBank: FJ423044.1 | (Foster *et al.*, 2009) |
| *Rpi-vnt1.3* | GenBank:FJ423046 | (Foster *et al.*, 2009) |
| *Rpi-amr3* | GenBank:KT373889 | (Witek *et al.*, 2016) |
| *R8* | GenBank: KU530153 | (Vossen *et al.*, 2016) |
| *R9a/Rpi-edn2* | <https://www.google.com/patents/US20140041072> | (Jo *et al.*, 2015) |
| *Rpi-Ph-3* | KJ563933.1 | (Zhang *et al.*, 2014) |
| *Gpa2* | GenBank: AF195939.1 | (van der Vossen *et al.*, 2000) |
| *Rx* | AJ011801.1 | (Bendahmane *et al.,* 1999) |

Shown are the gene names, the GenBank ID, and the reference detailing the molecular characterisation of the resistances.

**Supplementary Figure 1a: Sequence polymorphisms are reliably identified with dRenSeq: Example *Rpi-pta1* in transgenic Desiree line A23-29.**

Graphical representation of *Rpi-pta1* in transgenic Desiree line A23-29 following dRenSeq-mapping of paired-end reads. The start and stop position of the coding DNA sequence (CDS), relative to the reference sequence that included 5’ and 3’ flanking sequences is shown alongside the position of an intron. Paired-end read mapping was conducted at a 0% mismatch rate, not allowing for any sequence polymorphisms (top), and at a 0.5% mismatch rate, effectively allowing for 1 sequence polymorphism in 200 bp of sequence (middle). The position of a sequence polymorphism relative to the published reference sequence is highlighted. No reads mapped to the polymorphism at a 0% mismatch rate, but numerous paired-end reads with an identical polymorphism (middle and bottom) closed the gap at a 0.5% mismatch rate. The sequence polymorphism was independently confirmed following Sanger-sequencing of plasmids and transgenic lines.

**Supplementary Figure 1b: Sequence polymorphisms are reliably identified with dRenSeq: Example *Rpi-sto1* in transgenic Desiree line A14-81.**

Graphical representation of *Rpi-sto1* in transgenic Desiree line A14-81 following dRenSeq mapping of paired-end reads. The start and stop position of the coding DNA sequence (CDS), relative to the reference sequence that included 5’ and 3’ flanking sequences is shown alongside the position of an intron. Paired-end read mapping was conducted at a 0% mismatch rate, not allowing for any sequence polymorphisms (top), and at a 0.5% mismatch rate, effectively allowing for 1 sequence polymorphism in 200 bp of sequence (middle). The position of a sequence polymorphism relative to the published reference sequence is highlighted. No reads mapped to the polymorphism at a 0% mismatch rate, but numerous paired-end reads with an identical polymorphism (middle and bottom) closed the gap at a 0.5% mismatch rate. The sequence polymorphism was independently confirmed following Sanger-sequencing of plasmids and transgenic lines.

**Supplementary Figure 1c: Sequence polymorphisms are reliably identified with dRenSeq: Example *Rpi-vnt1.1* in transgenic Desiree line A23-29.**

Graphical representation of *Rpi-vnt1.1* in transgenic Desiree line A14-81 following dRenSeq mapping of paired-end reads. The start and stop position of the coding DNA sequence (CDS), relative to the reference sequence that included 5’ and 3’ flanking sequences is shown. Paired-end read mapping was conducted at a 0% mismatch rate, not allowing for any sequence polymorphisms (top), and at a 0.5% mismatch rate, effectively allowing for 1 sequence polymorphism in 200 bp of sequence (middle). The position of a sequence polymorphism relative to the published reference sequence is highlighted. No reads mapped to the polymorphism at a 0% mismatch rate, but numerous paired-end reads with an identical polymorphism (middle and bottom) closed the gap at a 0.5% mismatch rate. The sequence polymorphism was independently confirmed following Sanger-sequencing of plasmids and transgenic lines.

**Supplementary Figure 2: A F1 population derived from a cross between varieties Alouette x Vitalia segregates for recognition of *Avr-vnt1.***

**a.**

abaxial view adaxial view

Avr8

Avrsto1

Avrvnt1

R8+Avr8

Avr2

RD28

Avr8

Avrsto1

Avrvnt1

R8+Avr8

Avr2

RD28

**b.**


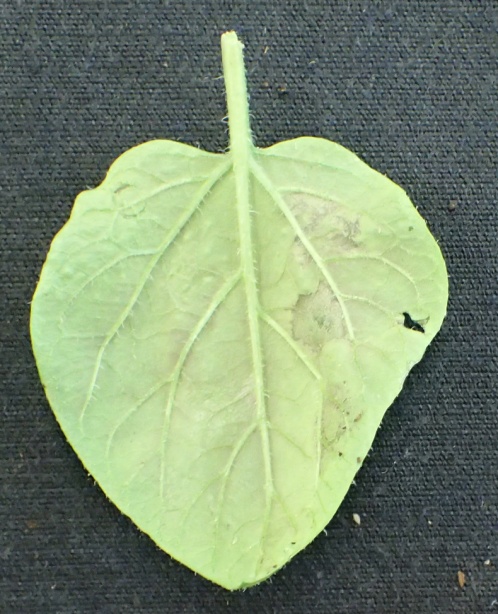

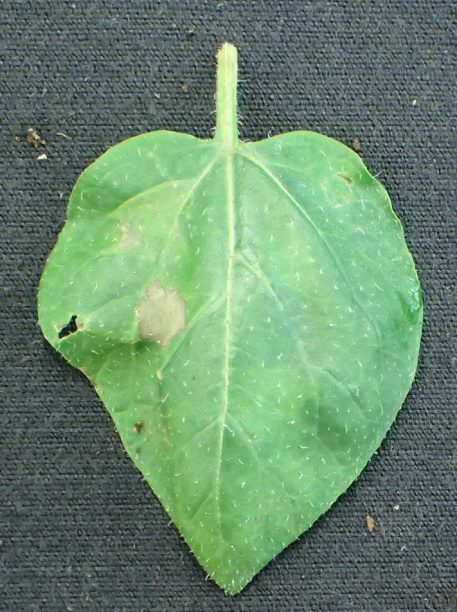


Avr-vnt1

R8+Avr8

Agroinfiltration of late blight A*vr* effectors in the Alouette*Vitalia population. **a**: agroinfiltration scheme; **b**: representative pictures of an infiltrated leaf from resistant clone (Al*Vi-59) three days post infiltration.

Agroinfiltration with five *Avr* genes was performed in 17 randomly selected clones (8 susceptible and 9 resistant) from the Al*Vi population (Supplementary Table 4). Infiltrations were repeated in three plants using three leaves per plant. In nearly all infiltrated leaves, the positive control (R8+Avr8) showed an obvious hypersensitive response (HR). The 9 resistant clones from the Al*Vi population responded to *Avr-vnt1* infiltration with an HR, while the 8 susceptible genotypes showed no response. None of the other *Avr* genes tested triggered an HR in any of the tested Al*Vi clones, showing that there was a very specific recognition of *Avr-vnt1* only in the resistant plants.

**Supplementary Figure 3**


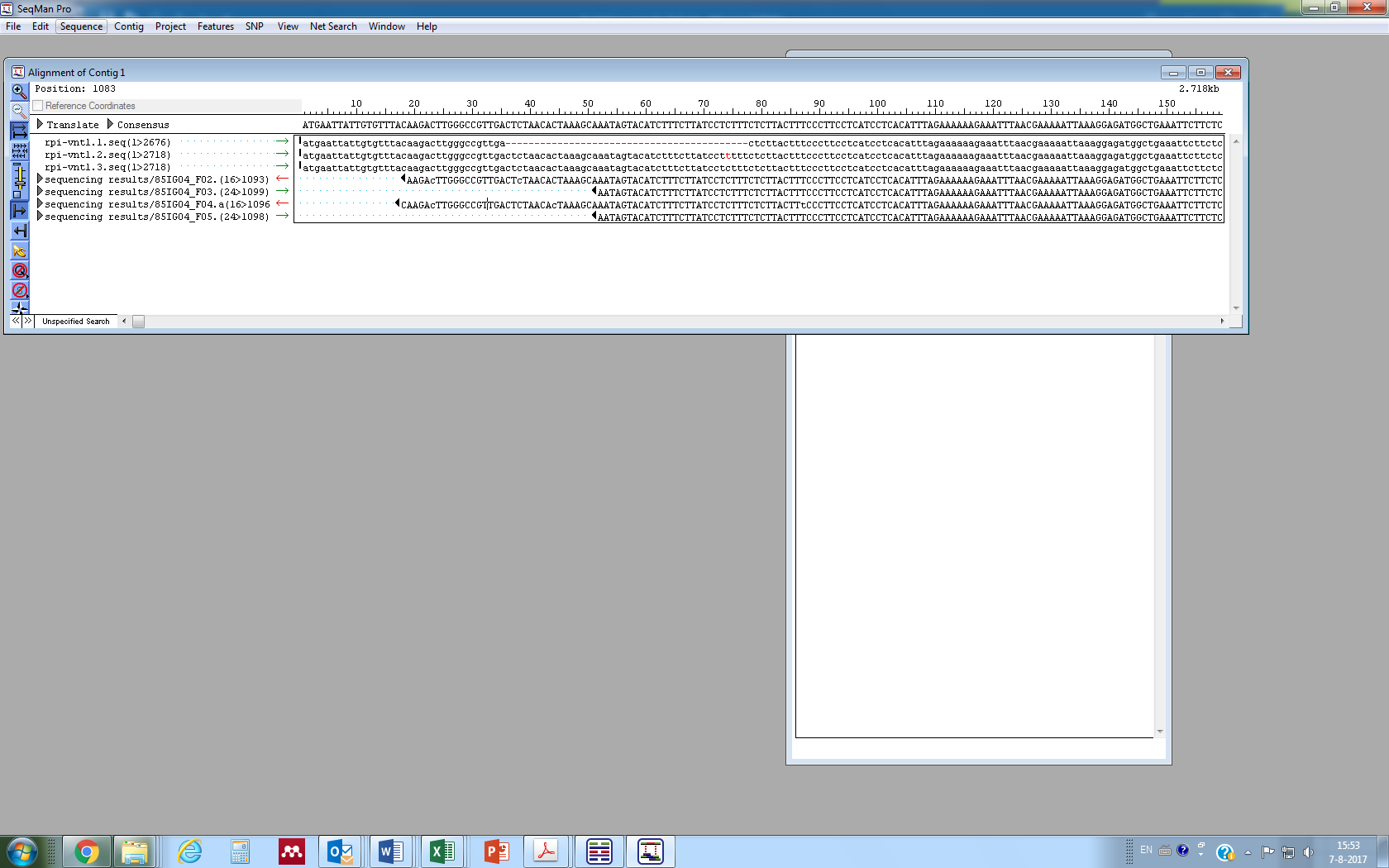


*Rpi-vnt1* PCR analysis in the Al*Vi population. To identify which allele of *Rpi-vnt1* was present in Alouette, we sequenced the PCR products F02 and F03 from clone Al*Vi-4 and F04 and F05 from clone Al*Vi-47 using Sanger sequencing. F02 and F04 were sequenced with primer LK69, and F03 and F05 were sequenced with primer LK70. Sequence alignments show that these amplicons contain a sequence that was identical to *Rpi-vnt1.3*. Only the sequences matching the 5’ end of *Rpi-vnt1* are shown. No polymorphisms were present in the remainder of the amplicon sequences.

**Supplementary Figure 4a: Sequence polymorphisms are reliably identified with dRenSeq: Example *Rpi-R3b* in potato variety Innovator**


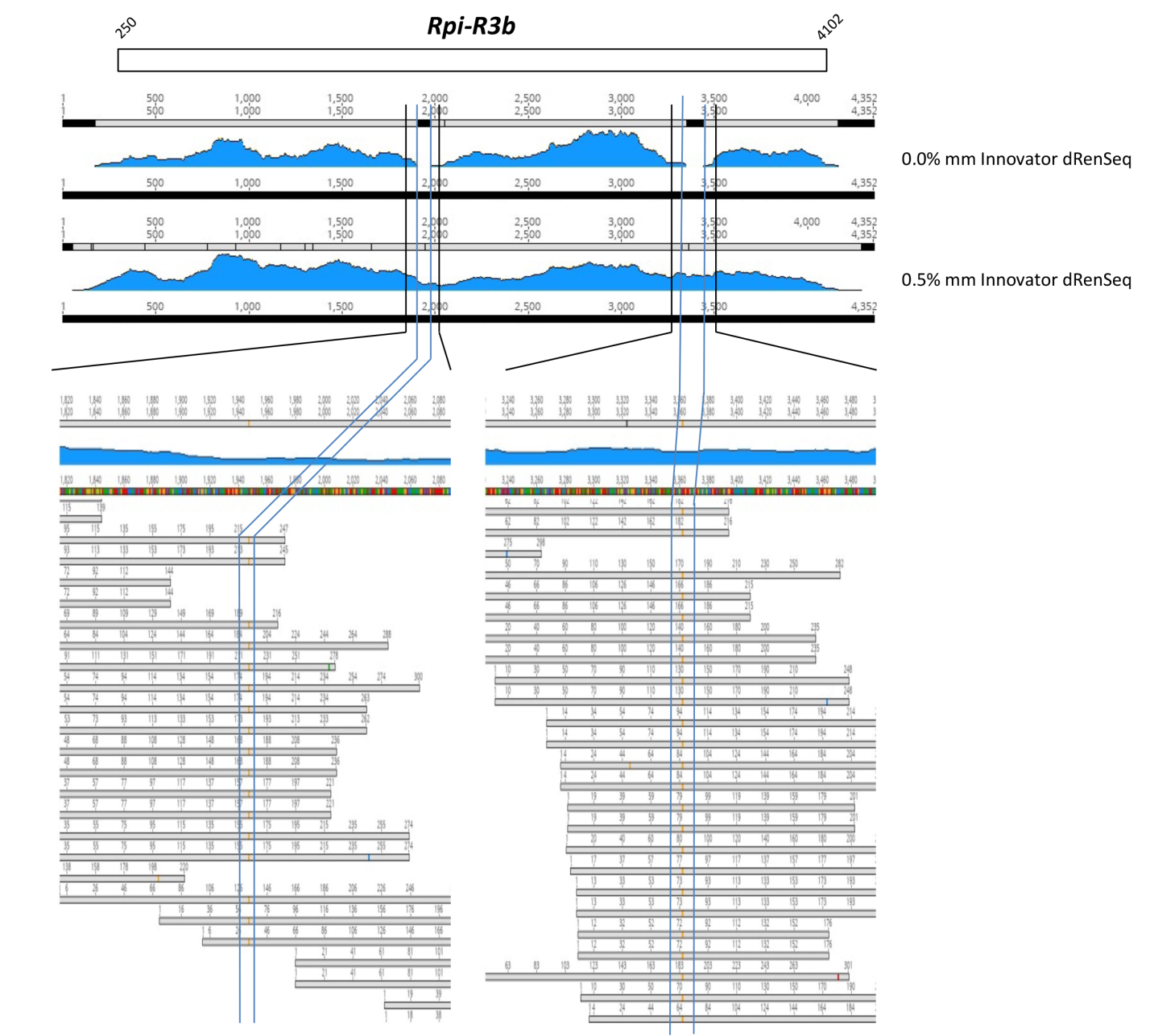


Graphical representation of *Rpi-R3b* in potato variety Innovator following dRenSeq mapping of paired-end reads. The start and stop position of the coding DNA sequence (CDS), relative to the reference sequence that included 5’ and 3’ flanking sequences is shown. Paired-end read mapping was conducted at a 0% mismatch rate, not allowing for any sequence polymorphisms (top), and at a 0.5% mismatch rate, effectively allowing for 1 sequence polymorphism in 200 bp of sequence (middle). The positions of two sequence polymorphism relative to the published reference sequence is highlighted. No reads mapped to the polymorphism at a 0% mismatch rate, but numerous paired-end reads with an identical polymorphism (middle and bottom) closed the gap at a 0.5% mismatch rate.

**Supplementary Figure 4b: Sequence polymorphisms identified by dRenSeq are also found by whole-genome sequencing (WGS): Example *Rpi-R3b* in potato variety Innovator**


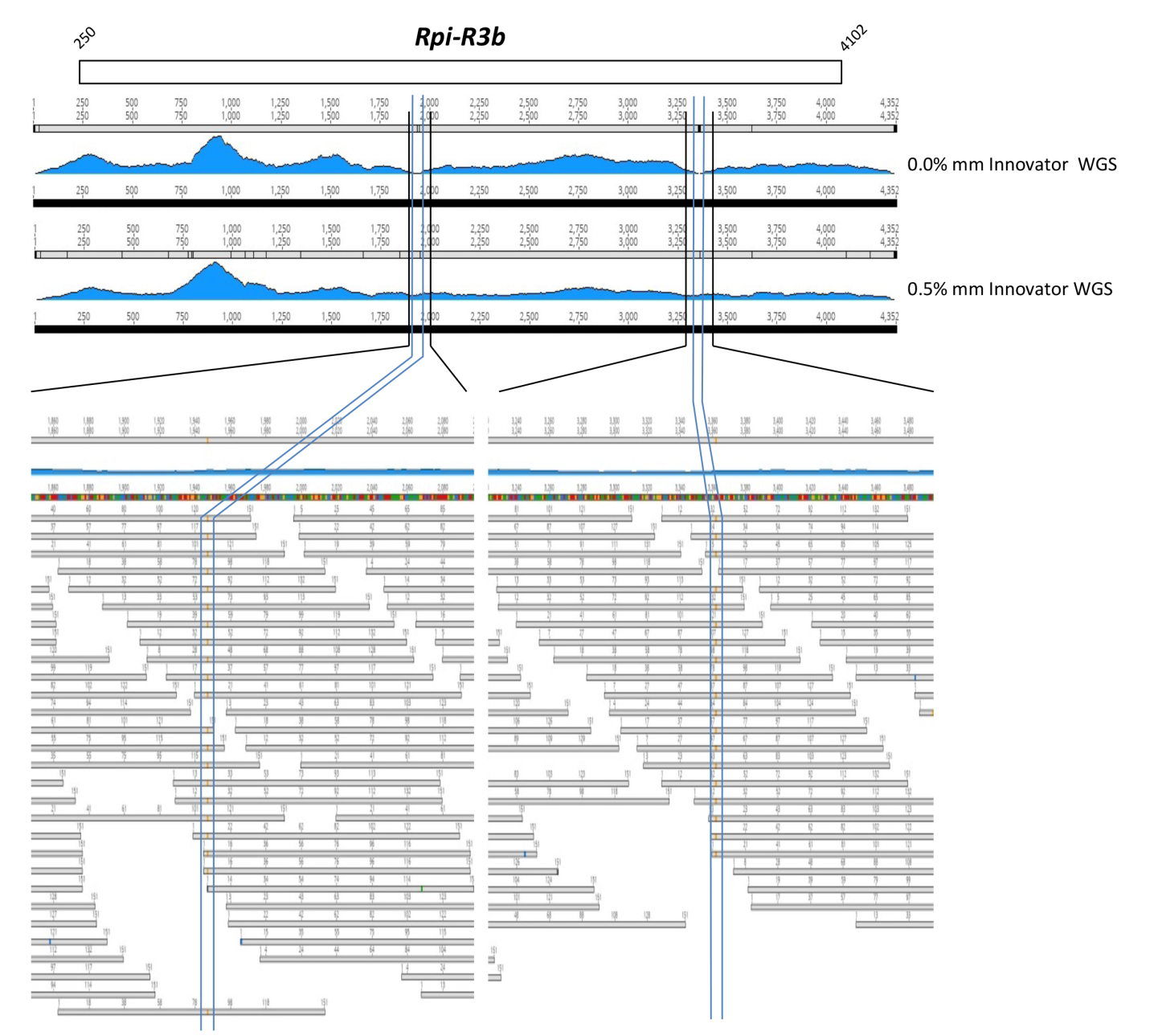


Graphical representation of *Rpi-R3b* in potato variety Innovator following mapping of whole-genome sequencing-derived paired-end reads. The start and stop position of the coding DNA sequence (CDS), relative to the reference sequence that included 5’ and 3’ flanking sequences is shown. Paired-end read mapping was conducted at a 0% mismatch rate, not allowing for any sequence polymorphisms (top), and at a 0.5% mismatch rate, effectively allowing for 1 sequence polymorphism in 200 bp of sequence (middle). The positions of two sequence polymorphism relative to the published reference sequence is highlighted. No reads mapped to the polymorphism at a 0% mismatch rate, but numerous paired-end reads with an identical polymorphism (middle and bottom) closed the gap at a 0.5% mismatch rate.

# References

Ballvora, A., Ercolano, M. R., Weiß, J., Meksem, K., Bormann, C. A., Oberhagemann, P., Salamini, F. and Gebhardt, C. (2002) ‘The R1 gene for potato resistance to late blight (Phytophthora infestans) belongs to the leucine zipper/NBS/LRR class of plant resistance genes’, *Plant Journal*, 30(3), pp. 361–371.

Bendahmane, A., Kanyuka, K. and Baulcombe, D. C. (1999) ‘The Rx Gene from Potato Controls Separate Virus Resistance and Cell Death Responses’, *The Plant Cell*, 11(5), pp. 781–792.

Black, W., Mastenbroek, C., Mills, W. R. and Peterson, L. C. (1953) ‘A proposal for an international nomenclature of races of Phytophthora infestans and of genes controlling immunity in Solanum demissum derivatives’, *Euphytica*, 2(3), pp. 173–179.

Foster, S. J., Park, T.-H., Pel, M., Brigneti, G., Śliwka, J., Jagger, L., van der Vossen, E. and Jones, J. D. G. (2009) ‘Rpi-vnt1.1, a Tm-22 Homolog from Solanum venturii, Confers Resistance to Potato Late Blight’, *Molecular Plant-Microbe Interactions*, 22(5), pp. 589–600.

Haverkort, A. J., Struik, P. C., Visser, R. G. F. and Jacobsen, E. (2009) ‘Applied biotechnology to combat late blight in potato caused by phytophthora infestans’, *Potato Research*, 52(3), pp. 249–264.

Huang, S., Van Der Vossen, E. A. G., Kuang, H., Vleeshouwers, V. G. A. A., Zhang, N., Borm, T. J. A., Van Eck, H. J., Baker, B., Jacobsen, E. and Visser, R. G. F. (2005) ‘Comparative genomics enabled the isolation of the R3a late blight resistance gene in potato’, *Plant Journal*, 42(2), pp. 251–261.

Jo, K.-R., Visser, R. G. F., Jacobsen, E. and Vossen, J. H. (2015) ‘Characterisation of the late blight resistance in potato differential MaR9 reveals a qualitative resistance gene, R9a, residing in a cluster of Tm-2 2 homologs on chromosome IX’, *Theoretical and Applied Genetics*. 128(5), pp 931–941.

Li, G., Huang, S., Guo, X., Li, Y., Guo, Z. and Kuang, H. (2011) ‘Cloning and Characterization of R3b;Members of the R3 Superfamily of Late Blight Resistance Genes Show Sequence and Functional Divergence’, *American Phytopathological Society*, 24(10), pp. 1132–1142.

Lokossou, A. a, Park, T., van Arkel, G., Arens, M., Ruyter-Spira, C., Morales, J., Whisson, S. C., Birch, P. R. J., Visser, R. G. F., Jacobsen, E. and van der Vossen, E. a G. (2009) ‘Exploiting knowledge of R/Avr genes to rapidly clone a new LZ-NBS-LRR family of late blight resistance genes from potato linkage group IV.’, *Molecular plant-microbe interactions : MPMI*, 22(6), pp. 630–641.

Malcolmson, J. F. (1969) ‘Races of Phytophthora infestans occurring in Great Britain’, *Transactions of the British Mycological Society*, 53(3), pp. 417

Stewart, H. E. and Bradshaw, J. E. (2001) ‘Assessment of the field resistance of potato genotypes with major gene resistance to late blight (Phytophthora infestans (Mont.) de Bary) using inoculum comprised of two complementary races of the fungus’, *Potato Research*, 44(1), pp. 41–52.

Vleeshouwers, V. G. A. A., Rietman, H., Krenek, P., Champouret, N., Young, C., Oh, S. K., Wang, M., Bouwmeester, K., Vosman, B., Visser, R. G. F., Jacobsen, E., Govers, F., Kamoun, S. and Van der Vossen, E. A. G. (2008) ‘Effector genomics accelerates discovery and functional profiling of potato disease resistance and Phytophthora Infestans avirulence genes’, *PLoS ONE*, 3(8). doi: 10.1371/journal.pone.0002875.

Van Der Vossen, E. A. G., Gros, J., Sikkema, A., Muskens, M., Wouters, D., Wolters, P., Pereira, A. and Allefs, S. (2005) ‘The Rpi-blb2 gene from Solanum bulbocastanum is an Mi-1 gene homolog conferring broad-spectrum late blight resistance in potato’, *Plant Journal*, 44(2), pp. 208–222.

van der Vossen, E. A., van der Voort, J. N., Kanyuka, K., Bendahmane, A., Sandbrink, H., Baulcombe, D. C., Bakker, J., Stiekema, W. J. and Klein-Lankhorst, R. M. (2000) ‘Homologues of a single resistance-gene cluster in potato confer resistance to distinct pathogens: a virus and a nematode.’, *The Plant journal*, 23(5), pp. 567–76.

Van Der Vossen, E., Sikkema, A., Te Lintel Hekkert, B., Gros, J., Stevens, P., Muskens, M., Wouters, D., Pereira, A., Stiekema, W. and Allefs, S. (2003) ‘An ancient R gene from the wild potato species Solanum bulbocastanum confers broad-spectrum resistance to Phytophthora infestans in cultivated potato and tomato’, *Plant Journal*, 36(6), pp. 867–882.

Vossen, J. H., van Arkel, G., Bergervoet, M., Jo, K. R., Jacobsen, E. and Visser, R. G. F. (2016) ‘The Solanum demissum R8 late blight resistance gene is an Sw-5 homologue that has been deployed worldwide in late blight resistant varieties’, *Theoretical and Applied Genetics*. Springer Berlin Heidelberg, 129(9), pp. 1785–1796.

Witek, K., Jupe, F., Witek, A. I., Baker, D., Clark, M. D. and Jones, J. D. G. (2016) ‘Accelerated cloning of a potato late blight–resistance gene using RenSeq and SMRT sequencing’, *Nature Biotechnology*. Nature Publishing Group, 34(6), pp. 656–660.

Zhang, C., Liu, L., Wang, X., Vossen, J., Li, G., Li, T., Zheng, Z., Gao, J., Guo, Y., Visser, R. G. F., Li, J., Bai, Y. and Du, Y. (2014) ‘The Ph-3 gene from Solanum pimpinellifolium encodes CC-NBS-LRR protein conferring resistance to Phytophthora infestans’, *Theoretical and Applied Genetics*, 127(6), pp. 1353–1364.

# Appendix 1 – FASTA sequence of all reference NLRs used including their 5’ and 3’ flanking region

>Nem-Gpa2

TCTAGAGATTGGAATGGAGTGATTCTTAGGGGTTTCTTTTTGAATTAATATGAGGGTTAGTATTCAATCTTCAATTCGACATTTTCTCATAATTTCTTTATCTGTTTATTTTTCCTATTCGTAAATCTCTTGGGAAAAATTGGGGTTTTATCGATTTGGACTCCTTTTTGATGAAAAAGGTATATTTACGATCTTTATGTTATGGGTAAACTGATTTTAACATAAAATTATTGATTCATCGATTATTTTTATCATATTAACCGCGTACAATTTGGACTTTCCCGGTAAAGTTAAAGTATGATAAATTGAGAATTTCAAGGTCGATCTTAGCTCCATTTTTGATGAAATTTCATATTTGAACTTATCTAAGCATGGGTAAGATGTTTTTCAAGAAATATTTCATTTTCGAGTCGGGGTTTTGGATTCGAATATTTTAGGCTTCTTCAAGAATGTAGATTTTTGTTTAAATTGAGTTTGTGAATTGATTTCAACTCCATTTTCAAATTGGTTTTCACCATTAGCTTCCAAATACTTTAAGGATCATTTTACATCAAAAAATTCCAGATTTGGGTATCGTTTTCCGGTATGAGACTTTTGGACCGTTTTGCCCCTTTTCCCTAAATTTCTTGATTTTGGTGTCATTGGACTCGAATTGTGATTGTGAATAATTGTTTGAATAGATTATCGTGATCCAGATTATACTTGGAAAGGAAAGGCTCAAGTCAAGTAACTTTTGGAGTTCGTTTTAAGGCAAGTGGCTTCCAAACTTTGTAAAACTCTTAGACTACGCATGACTACTTTCCTAATTATGTTGGGGAGTAATGGGGGATTGAGGATGGGTTTTATTTGTTGATTGAAATTGTTGTAAATGAAAGATGGGGAATAAAACGAGCTAAATGTGTTATGTGTGACTTGAATTTGTTTGAATAAGTCATGTGATAACTGATATTGAGGGATAGAAGAGCATGAGCAGGCTATGATTGATACAGACATTGATGTTGAGGCAGATGATGTGTAATACTATGATGTGGTCGTGATATGGTTGTGATTGAGACATGTGATGTGTAATACTATGATGTGGTCGTGATATGGTTGTGATTGAGACAGGTGATGTGTAATACTATGATGTGGTCGTGATATGGTTGTGATTGAGACAGGTGATGTGTAATACTATGATGTGGTCGTGATATGGTTGTGACTGAGACAGGTGATGTGTAATACTATGATGTGGTCGTGATATGGTTGTGATTGAGACAGATGATGTGTAATACGATGATGTGATCGTGATATGATTGTGATTGATTACATGTGCATATTCATTATTCATCCCATGTGTGAACTATCTGTTGCATGAGTTCTGAGACACTGATATGAGGATGGATGGATATGAGACACAGTTGAGACTAGCTCCGGCTAGAGATGTATGAGATGGACTAGCTCCGGCTAGCGATTTGGATGCCGATGGGATCTGGTTCCGGCGGTGATACATGGTCCATGTGTGGCCCCCATGGGTTCTGATTTGAGTATTCAACGCGGACTGATTACGTCAACAGATGTGTATCGTAGGACAGACATGTATCACGACTACATGACATCATTATTGCATTTTGCATCGCATTTGCCTTATCTTTGTCTGTGATGTGTGGATTGTATCGGTTTACCCTTTTTATGTGGAATTTGATCTACTTGCTCTTATTTGTTGATCTGAGGTTGATGAGGATATACTGTTGGTTCTGGCTGTTGAATATGATCTGTTTAGTATAGGTTGGTTGGTTTGCTGCTAGATTGAAGTTTCGGTGGTTCGGTTGGGATTGAAAGGAGTTGTTTGTAGCTGCTAGTTTTGCTTAGTTTAGAGTTACTTGCGAGTACCTGTGGTTTTCGGTACTCACCCTTGCTTCTACACAATTGTGTAGGTTGACAGCTCTCTCTCAGATATTTTCTTTAGCAGATTGAGCTTTGAGACATACTCGAGAGGTAGCGGTTCATTCCAGACGTGCCCTTGAGTTATCTTTACTTTCAGTTTTGTTCTATTCGAGAACTATACTCTGAGACTTGTATATTTTTATTCGAATTCTGTATTTAGAGGTTTGTACATGTGACAACCAAATTCTGGGTAGTGTTAAGTCTTAATTAAAGTTTTCTGCTTATTTATTATCTTTTATTCTCGTATTTCTACTTCTCTATCGTTGTGGTTGGGTTAGGCTGACGTGTCTGGTGGGAAACGGACATGTGCCATCACATCCGGATTTGGGGTGTGACAAATATTTTGTTAGTTATATACAAAATTGTATGTAGTATATGTATATTTTCTGCTTTCATCACAATTGTATATAGATATTTGTATATTTTGTTAGTTATATACAAAATTGCTTGAAGTATATGTATATTTTCTGCTTAAATCATAATTGTATATATATATATATATATATATATTTCTATATTTTGTAAGTTATATACAATAGTATGAATTAAACAATATACAAACCTTACATTATTATATATACAGTTAGGTTACACCAAAAATTATCAAATTAAAGCACAACTTTTTTATCGAATCATATACAATTCATATATATAATTGACTTAGTAATTTTATACAACTACTTACACTTCTACATGGTATAAGAATTTTGCACAATTACTTACATATATACAATATTATCAATTAAACAATATACAAATCGTATAACTTATATATACAGTAAAATTACAACAACAACAACAAAAATTATCAAATTAAAGCACACCGTTGTTGTCGAATCATATACACTCCATATATACAAATTGTGTCATTCAATTTTTCGAACAAAAAATTAGAATTGAATTGTTAATATAAAATTTATCTAATATTGTATAAACAAAATTAAATTATTGCAAACCATTAGAATGAAAAAAACAAAAATAAACCGTTTTCCAAAATTTCAATTATATACTATACAAATCAATTGTATACTTTCTTGCCGTTCAAAACATGAAGTTTCCTTGAAAGAAACGCTTACCTAGCGTTGAATATACAAGAATATTGATTAATCGTATGCTTCAGTCGTTTGAGGAACCCAGTTGTTATTGTGTTTCTATTGCTATAGAACTCCTTTTTGGAAAAATATTTGATTTTGGACGATTAGCTTGAATCATGGGATTATATAAAATTTTTATTACCGTATTTAGCACTCATGTATCCATTTATTAAAAAAAAATTGTATAAATTATATTTTTAAAAGAAAATATACAAAATTAATGCTTCATAGCAAACTAAACTATACCCATTGAATGTAATTACTAAACTATACCTATAGAGCGTTATTTCATTAAATACGTTTATCATATATGAAGTTTTCCCTCAAGAGATCCTACACCTTATATATAGCTTCTCAAATGTGGAAATTCAATCTCACACCCAACAATCTTTCCCTCAGACTAAGTTTCATGGCCCAATATCACAATGATCCACGAGTCAATTCATGAGATTCACTATGTGTGTCACCCACATCGTCTAAGTATTTTATGGCAATCAAGCCCTACAACTTGCTTCTTCTTTATATATATATATATATATATATATATATATATATATATATGTGTGTGTGTGTGTGTGTGTGTGCGCATCTCTAATTAATCTCGTAAAGGGATTAAGGGGCCAATTTCAAAGAATTAGGCGATTTTCTTAGTTTTTCGTGTGTGTTAACCCATAGGTATTTTGGTGATATGGTTTTCGGATGATTTATTTTGTGCAACTTATATGGAACCCTTCGTAGGGAGTTAGTCTCACACTTTTTAGAGTCCATTTTGGGCATTCAGGGGCTAATTTATAGGAAATAGGTGATCTTCTCAGTTTGTCTGTATTAGCCCATGAATATTTTGGTGATATGTCTTCCGAATAATTTCTTTGTAAAATCTTTACGGGACCCTCCATAGGGAGTTAGTGGAGCAGTACGTATAGTCTCACAATTTTAGAGTTCATTTTGGGCATTTAGGGGCCAATTTACAGGATTTAGGCGACTTTCTCAGTGTTTTGTGTGTGTTAGCCCATTAATAGTTGGTGATATGACTTTCAGACGATTTCTTTGCTACACATTTACGGAACCCTCTGTAGGAAGTCGGGGGAGCAATACGTACAATCTCACAATTTTAGAGTCCATTTTAGGCATTTAGGGGCCAATTTAAAGAAATTGGACAATTTTCTCAGTTTTTCGTGTCTGTTAGCCATTAATATATTGGTGAATATGACCTACAGATGATTTCTAATCGAAATCTTTACGAAACCCTCAGTAGGGAGTTGGGGGAGCAATACGTACCGTCTGACAATTTTTAGAGTCCATTTTGGGCATTTAAGGGCCAATTTACAGGAATTAGACGATTTTCTTAGTATTTTTTCATGTGTTAGCCCATAAATATTTTGTTGATTTGACTTTTAGAGTCTAAACTTCTCATGTATATTAAGAGATATTTATGCTTGGTTAATTGAATCGAACTAGGAATAGAGAAATTCCTACTTGGATCTTAATATTTCTCTCTCTTTGATTTGGAAAATTCTAGGAAGTTGCTTTCAATGGAATTAAAATCATCAATCTCTTGTATGTAAGAAACATACTTATATTCATGAATAGATATGTTTAGGGTCTAATAATGAATTATCACAATTTTTTCTACTTTTTCTTGTCAGAGTCCTGCCTTTTTCTTTTTCTTTTTTAACTTTGGTCTCTGCTTTTGTCTACATGATGATAAGGTTGGTGGACCTAGCTGGAAATGTGATGGAAATAGCTAGTAAANGAAAGAACTTTGCATTTTCTGTTTTCTTAAAAACTGATAAATTACATAACTTGTGGCAATTTGTCCATTTTCATACTGAGAGATATTTCTATTTTTTTTGGATATATGGCTTATGCTGCTGTTACTTCCCTTATGAGAACCATACATCAATCAATGGAACTTACTGGATGTGATTTGCAACCGTTTTATGAAAAGCTCAAATCTTTGAGAGCTATTCTGGAGAAATCCTGCAATATAATGGGCGATCATGAGGGGTTAACAATCTTGGAAGTTGAAATCATAGAGGTAGCATACACAACAGAAGATATGGTTGACTCGGAATCAAGAAATGTTTTTTTAGCACGGAATGTGGGGAAAAGAAGCAGGGCTATGTGGGGGATTTTTTTCGTCTTGGAACAAGCACTAGAATGCATTGATTCCACCGTGAAACAGTGGATGGCAACATCGGACAGCATGAAAGATCTAAAACCACAAACTAGCTCACTTGTCAGTTTACCTGAACATGATGTTGAGCAGCCCGAGAATATAATGGTTGGCCGTGAAAATGAATTTGAGATGATGCTGGATCAACTTGCTAGAGGAGGAAGGGAACTAGAAGTTGTCTCAATCGTAGGGATGGGAGGCATCGGGAAAACAACTTTGGCTGCAAAACTCTATAGTGATCCTTACATTATGTCTCGATTTGATATTCGTGCAAAAGCAACTGTTTCACAAGAGTATTGTGTGAGAAATGTACTCCTAGGCCTTCTTTCTTTGACAAGTGATGAACCTGATTATCAGCTAGCGGACCAACTGCAAAAGCATCTGAAAGGCAGGAGATACTTGGTAGTCATTGATGACATATGGACTACAGAAGCTTGGGATGATATAAAACTATGTTTCCCAGACTGCGATAATGGAAGCAGAATACTCCTGACTACTCGGAATGTGGAAGTGGCTGAATATGCTAGCTCAGGTAAGCCTCCTCATCACATGCGCCTCATGAATTTTGACGAAAGTTGGAATTTACTACACAAAAAGATCTTTGAAAAAGAAGGTTCTTATTCTCCTGAATTTGAAAATATTGGGAAACAAATTGCATTAAAATGTGGAGGGTTACCTCTAGCAATTACTTTGATTGCTGGACTTCTCTCCAAAATCAGTAAAACATTGGATGAGTGGCAAAATGTTGCGGAGAATGTACGTTCGGTGGTAAGCACAGATCTTGAAGCAAAATGCATGAGAGTGTTGGCTTTGAGTTACCATCACTTGCCTTCTCACCTAAAACCGTGTTTTCTGTATTTTGCAATTTTCGCAGAGGATGAACGGATTTATGTAAATAAACTTGTTGAGTTATGGGCCGTAGAGGGGTTTTTGAATGAAGAAGAGGGAAAAAGCATAGAAGAGGTGGCAGAAACATGTATAAACGAACTTGTAGATAGAAGTCTAATTTCTATCCACAATGTGAGTTTTGATGGGGAAACACAGAGATGTGGAATGCATGATGTGACCCGTGAACTCTGTTTGAGGGAAGCTCGAAACATGAATTTTGTGAATGTTATCAGAGGAAAGAGTGATCAAAATTCATGTGCACAATCCATGCAGTGTTCCTTTAAGAGTCGAAGTCGGATCAGTATCCATAATGAGGAAGAATTGGTTTGGTGTCGTAACAGCGAGGCTCATTCTATCATCACGTTGTGTATATTCAAATGCGTCACACTGGAATTGTCTTTCAAGCTAGTAAGAGTACTAGATCTTGGTTTGACTACATGCCCAATTTTTCCCAGTGGAGTACTTTCTCTAATTCATTTGAGATACCTATCTTTGCGTTTTAATCCTCGCTTACAGCAGTATCGAGGATCGAAAGAAGCTGTTCCCTCATCAATAATAGACATTCCTCTATCGATATCAAGCCTATGCTATCTGCAAACTTTTAAACTTTACCATCCATTTCCCAATTGTTATCCTTTCATATTACCATCGGAAATTTTGACAATGCCACAATTGAGGAAGCTGTGTATGGGCTGGAATTACTTGCGGAGTCATGAGCCTACAGAGAACAGATTGGTTTTGAAAAGTTTGCAATGCCTCAATGAATTGAATCCTCGGTATTGTACAGGGTCTTTTTTAAGACTATTTCCCAATTTAAAGAAGTTGGAAGTATTTGGCGTCAAAGAGGACTTTCGCAATCACAAGGACCTGTATGATTTTCGCTACTTATATCAGCTCGAGAAATTGGCATTTAGTACTTATTATTCATCTTCTGCTTGCTTTCTAAAAAACACTGCACCTTTAGGTTCTACTCCGCAAGATCCTCTGAGGTTTCAGATGGAAACATTGCACTTAGAGACTCATTCCAGGGCAACTGCACCTCCAACTGATGTTCCAACTTTCCTCTTACCTCCTCCGGATTGTTTTCCACAAAACCTTAAGAGTTTAACTTTTAGCGGAGATTTCTTTTTGGCATGGAAGGATTTGAGCATTGTTGGTAAATTACCCAAACTCGAGGTCCTTCAACTATCACACAATGCCTTCAAAGGCGAGGAGTGGGAAGTAGTTGAGGAAGGGTTTCCTCACTTGAAGTTCTTGTTTCTGGATAGCATATACATTCGGTACTGGAGAGCTAGTAGTGATCACTTTCCATACCTTGAACGACTTTTTCTTAGCGATTGCTTTTATTTGGATTCAATCCCTCGAGATTTTGCAGATATAACCACACTAGCTCTTATTGATATATTTCGCTGCCAACAATCTGTTGGGAATTCCGCCAAGCAAATTCAACAGGACATTCAAGACAACTATGGAAGCTCTATCGAGGTCCATACTCGTTATCTTTAGTAAGACATCTTCTTCCTTGATTTACAACAATATTTAACTCATCATCATAGTAAACTCGATAATAATCTGGATAATAGCTTTAGTAAGTCAAATTGCACCAATTCAACAAAAGTTCTTGATGCTGTCATTGTGATTGATTCGAATCCTTCCAATATTGTGTAACTTGTTATACTTGCATGTTCATTCTTGATTTTGGGAAGTGTAACATTTCCATTTTTCATCTTGATTTTGGGAAGTCGAAATGGAGCATTTTTGGTAGTGTGACAACAGATGAAGATGATGATGATAGTGTGACAACAGATGAAGATGAAGATGAAGACTTTGAGAAAGAAGTTGCTTCTTGCGGCAATAATGTGTAAGTTCTTATACCTGCATGCTCATTCTTGCTATAATGTTCTCTTGTTCCTTAATTATGGGACATCTAACATATTATTTTCCATTTTTTGCATCTTTTTTTTTTCCTGCAGCGTGTAGTTAAGGTGTTCTGAGGACTAGCCAGTTCTCTGAAATAAATGTCAAATCAGAAGCCAAATGTGTGAGTGTTTGTTTTGTTCGTTTTCATTTTTTCTGCATAAGGTGGCAGGATGATTGCAAATGGCTTGTAATTTAATTGTATATGATATTTCGTATAGCCATTTGCCAGTGGTTTTTTAGATACTCCAAATTTTATGTACATACATAATGGTATAGGCCAGAACAGGCTCCATATATAACGTGTGTTTCCTTTCTTGGGAGTCCTCAATCTACCTCGCAAAGGAAGACAGACGGCTAAATCAAGAAAGAAATTTTTTTGAAAATCATGTGGCTAGTTGTTCAACTTTATACAAGTTTATGTGCATACTTGTGCATACCCAAAGTTGAATAACATAAACATAAAATGAAGTCAAGTTAAATGGCACATTTATGTATTATGCCTTTTGAATTTCATTAATAGTGAAAATCCTGAATCATATTCAGATTCCATCACTAATCGTTGAACCATGTTAATTTACTATGTATTATCTAATGGATTTTTTTGCTATCTTATTTATAATTGTTCAAAGTTTTGTTAATTATCTTTAGCATAATATCTGATTATATTATTTTGATATACTTTCTCTATCCCTAATTACTTGTCCATTTTTGAATTGGCACACCTATTAAGAAAATAATTATTGAAATAGTGAGTTTACCATTTTACCCATATTAATTATGAAGTGGATGAATTAAAAACTCAAGATTTTCAAAAAGTTCTATTTTTTTCAAAGTAATAAACTGACGGTATAATAGGTAAAAAAAATTATTCTTTCTTGATTTGTCAAAATAAACAAATAATTAGGAATAATTAAAAAAATGGATAAATAATTAAAAACGGAGGGAGCAATATGTTATCTTTAGCCTAATAATATCTGATTAATGGCCACCCTAATTGATTGGATAGGAGAGGATAGACTTGCTTCCAAGTAACCCAAAATATAAAAAGTTGACAAAAGGGTGCTAAATTCGAGACACATGTAGTACTTATATAATTCATGTGCGGACTCGTTCTTTTGTAGTACTCCCTCCGTTCTATTTTATACGTCACATTTTTACTTTATACTTTTATTAAGAAATGATGTAGTTTTATCTTTCTATTCTTATTTAATGTTTTCTTAAGTCAATTTTATAATAAATAATGAATATATTTTCAAGATTAATTAACTACTCTATCAAGGGTATAATAGGTAAAATATGATAATTTATACATAAATTTTATAAAATGACAAGTATTGTGGTCCAACTATTTATAGAAAGAAATGATATATAAAATGGGACGGAGGGCGTTATAAAGTTGACTTAAGAAAACATTAAATAAGGGTAGAAGGGTAAAATTACATTATTTCTTAATGTAAATGTAAAGTAAAAAGGTAACATATAAAATGGAAAGGAGGGAGTAGTATTTTCTTGTTTTATTTTACGTGGCACTCTATTCTCATAATCCGTCTTTAAAAATGTCATTTTATTGTAATTGAAAATAATTTAACTTAAAATTCTCCATCTACCCTTAATTAATGAAATGATTTACAATTATATAAATATATAAAAATTGTTTTAGCCTATAATTTTCTAAAATCTTTTTTTTTCTCTTATACATCGTATTAAGTCAAACATAAATGGAATGGACGGAGTATTTCTTTTATTTTTTTGTCACACCGCCCATATGTTTTCTCCCATCCCCCAGACCCCCACTATGTATATTCACTCCTTAGTTGGATCTGAATTTAGAGTTTAGAAGCTTCTATAATAATTTTAGATTAATATATAATAATAATAATAATAATTGAACTTACAGTATTAAATTTATGTGAATCTATATATATTGTATTGTAATTTTTTTAATTATAATTTTAACCAAATCAATAAAGCTATTCAGATGTAAAAGTATATATTATGATTTAACAACAAATTTCTATACGTCTTCCTAAGTTTTGATGCATAATTTCCTAAAACTCATAAATTTCCAAGTGACTACTTCCAGTATTACAATGAGAACTTATGTTTCGTTATGGATTTTCTTAGTGAATTAGTTTAATAAAATCAAAATGAAAAAAAATCATGTTTTATAACATAAAATTTTCATTGATTCATGCGAAAAAAAAACATCTAGTTCTTATAGTGTGAAAACTATTGAACTTATGGGATGTAGCTGTATGGAAGTTCATCAAGTGGTAGCTCCTTGTACGCAACTAGTGCTACTTTTTATTGACTAAAAGTTATTTTCTAGA

> *Nem-Gpa2^ΔC2922^*

TCTAGAGATTGGAATGGAGTGATTCTTAGGGGTTTCTTTTTGAATTAATATGAGGGTTAGTATTCAATCTTCAATTCGACATTTTCTCATAATTTCTTTATCTGTTTATTTTTCCTATTCGTAAATCTCTTGGGAAAAATTGGGGTTTTATCGATTTGGACTCCTTTTTGATGAAAAAGGTATATTTACGATCTTTATGTTATGGGTAAACTGATTTTAACATAAAATTATTGATTCATCGATTATTTTTATCATATTAACCGCGTACAATTTGGACTTTCCCGGTAAAGTTAAAGTATGATAAATTGAGAATTTCAAGGTCGATCTTAGCTCCATTTTTGATGAAATTTCATATTTGAACTTATCTAAGCATGGGTAAGATGTTTTTCAAGAAATATTTCATTTTCGAGTCGGGGTTTTGGATTCGAATATTTTAGGCTTCTTCAAGAATGTAGATTTTTGTTTAAATTGAGTTTGTGAATTGATTTCAACTCCATTTTCAAATTGGTTTTCACCATTAGCTTCCAAATACTTTAAGGATCATTTTACATCAAAAAATTCCAGATTTGGGTATCGTTTTCCGGTATGAGACTTTTGGACCGTTTTGCCCCTTTTCCCTAAATTTCTTGATTTTGGTGTCATTGGACTCGAATTGTGATTGTGAATAATTGTTTGAATAGATTATCGTGATCCAGATTATACTTGGAAAGGAAAGGCTCAAGTCAAGTAACTTTTGGAGTTCGTTTTAAGGCAAGTGGCTTCCAAACTTTGTAAAACTCTTAGACTACGCATGACTACTTTCCTAATTATGTTGGGGAGTAATGGGGGATTGAGGATGGGTTTTATTTGTTGATTGAAATTGTTGTAAATGAAAGATGGGGAATAAAACGAGCTAAATGTGTTATGTGTGACTTGAATTTGTTTGAATAAGTCATGTGATAACTGATATTGAGGGATAGAAGAGCATGAGCAGGCTATGATTGATACAGACATTGATGTTGAGGCAGATGATGTGTAATACTATGATGTGGTCGTGATATGGTTGTGATTGAGACATGTGATGTGTAATACTATGATGTGGTCGTGATATGGTTGTGATTGAGACAGGTGATGTGTAATACTATGATGTGGTCGTGATATGGTTGTGATTGAGACAGGTGATGTGTAATACTATGATGTGGTCGTGATATGGTTGTGACTGAGACAGGTGATGTGTAATACTATGATGTGGTCGTGATATGGTTGTGATTGAGACAGATGATGTGTAATACGATGATGTGATCGTGATATGATTGTGATTGATTACATGTGCATATTCATTATTCATCCCATGTGTGAACTATCTGTTGCATGAGTTCTGAGACACTGATATGAGGATGGATGGATATGAGACACAGTTGAGACTAGCTCCGGCTAGAGATGTATGAGATGGACTAGCTCCGGCTAGCGATTTGGATGCCGATGGGATCTGGTTCCGGCGGTGATACATGGTCCATGTGTGGCCCCCATGGGTTCTGATTTGAGTATTCAACGCGGACTGATTACGTCAACAGATGTGTATCGTAGGACAGACATGTATCACGACTACATGACATCATTATTGCATTTTGCATCGCATTTGCCTTATCTTTGTCTGTGATGTGTGGATTGTATCGGTTTACCCTTTTTATGTGGAATTTGATCTACTTGCTCTTATTTGTTGATCTGAGGTTGATGAGGATATACTGTTGGTTCTGGCTGTTGAATATGATCTGTTTAGTATAGGTTGGTTGGTTTGCTGCTAGATTGAAGTTTCGGTGGTTCGGTTGGGATTGAAAGGAGTTGTTTGTAGCTGCTAGTTTTGCTTAGTTTAGAGTTACTTGCGAGTACCTGTGGTTTTCGGTACTCACCCTTGCTTCTACACAATTGTGTAGGTTGACAGCTCTCTCTCAGATATTTTCTTTAGCAGATTGAGCTTTGAGACATACTCGAGAGGTAGCGGTTCATTCCAGACGTGCCCTTGAGTTATCTTTACTTTCAGTTTTGTTCTATTCGAGAACTATACTCTGAGACTTGTATATTTTTATTCGAATTCTGTATTTAGAGGTTTGTACATGTGACAACCAAATTCTGGGTAGTGTTAAGTCTTAATTAAAGTTTTCTGCTTATTTATTATCTTTTATTCTCGTATTTCTACTTCTCTATCGTTGTGGTTGGGTTAGGCTGACGTGTCTGGTGGGAAACGGACATGTGCCATCACATCCGGATTTGGGGTGTGACAAATATTTTGTTAGTTATATACAAAATTGTATGTAGTATATGTATATTTTCTGCTTTCATCACAATTGTATATAGATATTTGTATATTTTGTTAGTTATATACAAAATTGCTTGAAGTATATGTATATTTTCTGCTTAAATCATAATTGTATATATATATATATATATATATATTTCTATATTTTGTAAGTTATATACAATAGTATGAATTAAACAATATACAAACCTTACATTATTATATATACAGTTAGGTTACACCAAAAATTATCAAATTAAAGCACAACTTTTTTATCGAATCATATACAATTCATATATATAATTGACTTAGTAATTTTATACAACTACTTACACTTCTACATGGTATAAGAATTTTGCACAATTACTTACATATATACAATATTATCAATTAAACAATATACAAATCGTATAACTTATATATACAGTAAAATTACAACAACAACAACAAAAATTATCAAATTAAAGCACACCGTTGTTGTCGAATCATATACACTCCATATATACAAATTGTGTCATTCAATTTTTCGAACAAAAAATTAGAATTGAATTGTTAATATAAAATTTATCTAATATTGTATAAACAAAATTAAATTATTGCAAACCATTAGAATGAAAAAAACAAAAATAAACCGTTTTCCAAAATTTCAATTATATACTATACAAATCAATTGTATACTTTCTTGCCGTTCAAAACATGAAGTTTCCTTGAAAGAAACGCTTACCTAGCGTTGAATATACAAGAATATTGATTAATCGTATGCTTCAGTCGTTTGAGGAACCCAGTTGTTATTGTGTTTCTATTGCTATAGAACTCCTTTTTGGAAAAATATTTGATTTTGGACGATTAGCTTGAATCATGGGATTATATAAAATTTTTATTACCGTATTTAGCACTCATGTATCCATTTATTAAAAAAAAATTGTATAAATTATATTTTTAAAAGAAAATATACAAAATTAATGCTTCATAGCAAACTAAACTATACCCATTGAATGTAATTACTAAACTATACCTATAGAGCGTTATTTCATTAAATACGTTTATCATATATGAAGTTTTCCCTCAAGAGATCCTACACCTTATATATAGCTTCTCAAATGTGGAAATTCAATCTCACACCCAACAATCTTTCCCTCAGACTAAGTTTCATGGCCCAATATCACAATGATCCACGAGTCAATTCATGAGATTCACTATGTGTGTCACCCACATCGTCTAAGTATTTTATGGCAATCAAGCCCTACAACTTGCTTCTTCTTTATATATATATATATATATATATATATATATATATATATATGTGTGTGTGTGTGTGTGTGTGTGCGCATCTCTAATTAATCTCGTAAAGGGATTAAGGGGCCAATTTCAAAGAATTAGGCGATTTTCTTAGTTTTTCGTGTGTGTTAACCCATAGGTATTTTGGTGATATGGTTTTCGGATGATTTATTTTGTGCAACTTATATGGAACCCTTCGTAGGGAGTTAGTCTCACACTTTTTAGAGTCCATTTTGGGCATTCAGGGGCTAATTTATAGGAAATAGGTGATCTTCTCAGTTTGTCTGTATTAGCCCATGAATATTTTGGTGATATGTCTTCCGAATAATTTCTTTGTAAAATCTTTACGGGACCCTCCATAGGGAGTTAGTGGAGCAGTACGTATAGTCTCACAATTTTAGAGTTCATTTTGGGCATTTAGGGGCCAATTTACAGGATTTAGGCGACTTTCTCAGTGTTTTGTGTGTGTTAGCCCATTAATAGTTGGTGATATGACTTTCAGACGATTTCTTTGCTACACATTTACGGAACCCTCTGTAGGAAGTCGGGGGAGCAATACGTACAATCTCACAATTTTAGAGTCCATTTTAGGCATTTAGGGGCCAATTTAAAGAAATTGGACAATTTTCTCAGTTTTTCGTGTCTGTTAGCCATTAATATATTGGTGAATATGACCTACAGATGATTTCTAATCGAAATCTTTACGAAACCCTCAGTAGGGAGTTGGGGGAGCAATACGTACCGTCTGACAATTTTTAGAGTCCATTTTGGGCATTTAAGGGCCAATTTACAGGAATTAGACGATTTTCTTAGTATTTTTTCATGTGTTAGCCCATAAATATTTTGTTGATTTGACTTTTAGAGTCTAAACTTCTCATGTATATTAAGAGATATTTATGCTTGGTTAATTGAATCGAACTAGGAATAGAGAAATTCCTACTTGGATCTTAATATTTCTCTCTCTTTGATTTGGAAAATTCTAGGAAGTTGCTTTCAATGGAATTAAAATCATCAATCTCTTGTATGTAAGAAACATACTTATATTCATGAATAGATATGTTTAGGGTCTAATAATGAATTATCACAATTTTTTCTACTTTTTCTTGTCAGAGTCCTGCCTTTTTCTTTTTCTTTTTTAACTTTGGTCTCTGCTTTTGTCTACATGATGATAAGGTTGGTGGACCTAGCTGGAAATGTGATGGAAATAGCTAGTAAANGAAAGAACTTTGCATTTTCTGTTTTCTTAAAAACTGATAAATTACATAACTTGTGGCAATTTGTCCATTTTCATACTGAGAGATATTTCTATTTTTTTTGGATATATGGCTTATGCTGCTGTTACTTCCCTTATGAGAACCATACATCAATCAATGGAACTTACTGGATGTGATTTGCAACCGTTTTATGAAAAGCTCAAATCTTTGAGAGCTATTCTGGAGAAATCCTGCAATATAATGGGCGATCATGAGGGGTTAACAATCTTGGAAGTTGAAATCATAGAGGTAGCATACACAACAGAAGATATGGTTGACTCGGAATCAAGAAATGTTTTTTTAGCACGGAATGTGGGGAAAAGAAGCAGGGCTATGTGGGGGATTTTTTTCGTCTTGGAACAAGCACTAGAATGCATTGATTCCACCGTGAAACAGTGGATGGCAACATCGGACAGCATGAAAGATCTAAAACCACAAACTAGCTCACTTGTCAGTTTACCTGAACATGATGTTGAGCAGCCCGAGAATATAATGGTTGGCCGTGAAAATGAATTTGAGATGATGCTGGATCAACTTGCTAGAGGAGGAAGGGAACTAGAAGTTGTCTCAATCGTAGGGATGGGAGGCATCGGGAAAACAACTTTGGCTGCAAAACTCTATAGTGATCCTTACATTATGTCTCGATTTGATATTCGTGCAAAAGCAACTGTTTCACAAGAGTATTGTGTGAGAAATGTACTCCTAGGCCTTCTTTCTTTGACAAGTGATGAACCTGATTATCAGCTAGCGGACCAACTGCAAAAGCATCTGAAAGGCAGGAGATACTTGGTAGTCATTGATGACATATGGACTACAGAAGCTTGGGATGATATAAAACTATGTTTCCCAGACTGCGATAATGGAAGCAGAATACTCCTGACTACTCGGAATGTGGAAGTGGCTGAATATGCTAGCTCAGGTAAGCCTCCTCATCACATGCGCCTCATGAATTTTGACGAAAGTTGGAATTTACTACACAAAAAGATCTTTGAAAAAGAAGGTTCTTATTCTCCTGAATTTGAAAATATTGGGAAACAAATTGCATTAAAATGTGGAGGGTTACCTCTAGCAATTACTTTGATTGCTGGACTTCTCTCCAAAATCAGTAAAACATTGGATGAGTGGCAAAATGTTGCGGAGAATGTACGTTCGGTGGTAAGCACAGATCTTGAAGCAAAATGCATGAGAGTGTTGGCTTTGAGTTACCATCACTTGCCTTCTCACCTAAAACCGTGTTTTCTGTATTTTGCAATTTTCGCAGAGGATGAACGGATTTATGTAAATAAACTTGTTGAGTTATGGGCCGTAGAGGGGTTTTTGAATGAAGAAGAGGGAAAAAGCATAGAAGAGGTGGCAGAAACATGTATAAACGAACTTGTAGATAGAAGTCTAATTTCTATCCACAATGTGAGTTTTGATGGGGAAACACAGAGATGTGGAATGCATGATGTGACCCGTGAACTCTGTTTGAGGGAAGCTCGAAACATGAATTTTGTGAATGTTATCAGAGGAAAGAGTGATCAAAATTCATGTGCACAATCCATGCAGTGTTCCTTTAAGAGTCGAAGTCGGATCAGTATCCATAATGAGGAAGAATTGGTTTGGTGTCGTAACAGCGAGGCTCATTCTATCATCACGTTGTGTATATTCAAATGCGTCACACTGGAATTGTCTTTCAAGCTAGTAAGAGTACTAGATCTTGGTTTGACTACATGCCCAATTTTTCCCAGTGGAGTACTTTCTCTAATTCATTTGAGATACCTATCTTTGCGTTTTAATCCTCGCTTACAGCAGTATCGAGGATCGAAAGAAGCTGTTCCCTCATCAATAATAGACATTCCTCTATCGATATCAAGCCTATGCTATCTGCAAACTTTTAAACTTTACCATCCATTTCCCAATTGTTATCCTTTCATATTACCATCGGAAATTTTGACAATGCCACAATTGAGGAAGCTGTGTATGGGCTGGAATTACTTGCGGAGTCATGAGCCTACAGAGAACAGATTGGTTTTGAAAAGTTTGCAATGCCTCAATGAATTGAATCCTCGGTATTGTACAGGGTCTTTTTTAAGACTATTTCCCAATTTAAAGAAGTTGGAAGTATTTGGCGTCAAAGAGGACTTTCGCAATCACAAGGACCTGTATGATTTTCGCTACTTATATCAGCTCGAGAAATTGGCATTTAGTACTTATTATTCATCTTCTGCTTGCTTTCTAAAAAACACTGCACCTTTAGGTTCTACTCCGCAAGATCCTCTGAGGTTTCAGATGGAAACATTGCACTTAGAGACTCATTCCAGGGCAACTGCACCTCCAACTGATGTTCCAACTTTCCTCTTACCTCCTCCGGATTGTTTTCCACAAAACCTTAAGAGTTTAACTTTTAGCGGAGATTTCTTTTTGGCATGGAAGGATTTGAGCATTGTTGGTAAATTACCCAAACTCGAGGTCCTTCAACTATCACACAATGCCTTCAAAGGCGAGGAGTGGGAAGTAGTTGAGGAAGGGTTTCCTCACTTGAAGTTCTTGTTTCTGGATAGCATATACATTCGGTACTGGAGAGCTAGTAGTGATCACTTTCCATACCTTGAACGACTTTTTCTTAGCGATTGCTTTTATTTGGATTCAATCCCTCGAGATTTTGCAGATATAACCACACTAGCTCTTATTGATATATTTCGCTGCCAACAATCTGTTGGGAATTCCGCCAAGCAAATTCAACAGGACATTCAAGACAACTATGGAAGCTCTATCGAGGTCCATACTCGTTATCTTTAGTAAGACATCTTCTTCCTTGATTTACAACAATATTTAACTCATCATCATAGTAAACTCGATAATAATCTGGATAATAGCTTTAGTAAGTCAAATTGCACCAATTCAACAAAAGTTCTTGATGCTGTCATTGTGATTGATTCGAATCCTTCCAATATTGTGTAACTTGTTATACTTGCATGTTCATTCTTGATTTTGGGAAGTGTAACATTTCATTTTTCATCTTGATTTTGGGAAGTCGAAATGGAGCATTTTTGGTAGTGTGACAACAGATGAAGATGATGATGATAGTGTGACAACAGATGAAGATGAAGATGAAGACTTTGAGAAAGAAGTTGCTTCTTGCGGCAATAATGTGTAAGTTCTTATACCTGCATGCTCATTCTTGCTATAATGTTCTCTTGTTCCTTAATTATGGGACATCTAACATATTATTTTCCATTTTTTGCATCTTTTTTTTTTCCTGCAGCGTGTAGTTAAGGTGTTCTGAGGACTAGCCAGTTCTCTGAAATAAATGTCAAATCAGAAGCCAAATGTGTGAGTGTTTGTTTTGTTCGTTTTCATTTTTTCTGCATAAGGTGGCAGGATGATTGCAAATGGCTTGTAATTTAATTGTATATGATATTTCGTATAGCCATTTGCCAGTGGTTTTTTAGATACTCCAAATTTTATGTACATACATAATGGTATAGGCCAGAACAGGCTCCATATATAACGTGTGTTTCCTTTCTTGGGAGTCCTCAATCTACCTCGCAAAGGAAGACAGACGGCTAAATCAAGAAAGAAATTTTTTTGAAAATCATGTGGCTAGTTGTTCAACTTTATACAAGTTTATGTGCATACTTGTGCATACCCAAAGTTGAATAACATAAACATAAAATGAAGTCAAGTTAAATGGCACATTTATGTATTATGCCTTTTGAATTTCATTAATAGTGAAAATCCTGAATCATATTCAGATTCCATCACTAATCGTTGAACCATGTTAATTTACTATGTATTATCTAATGGATTTTTTTGCTATCTTATTTATAATTGTTCAAAGTTTTGTTAATTATCTTTAGCATAATATCTGATTATATTATTTTGATATACTTTCTCTATCCCTAATTACTTGTCCATTTTTGAATTGGCACACCTATTAAGAAAATAATTATTGAAATAGTGAGTTTACCATTTTACCCATATTAATTATGAAGTGGATGAATTAAAAACTCAAGATTTTCAAAAAGTTCTATTTTTTTCAAAGTAATAAACTGACGGTATAATAGGTAAAAAAAATTATTCTTTCTTGATTTGTCAAAATAAACAAATAATTAGGAATAATTAAAAAAATGGATAAATAATTAAAAACGGAGGGAGCAATATGTTATCTTTAGCCTAATAATATCTGATTAATGGCCACCCTAATTGATTGGATAGGAGAGGATAGACTTGCTTCCAAGTAACCCAAAATATAAAAAGTTGACAAAAGGGTGCTAAATTCGAGACACATGTAGTACTTATATAATTCATGTGCGGACTCGTTCTTTTGTAGTACTCCCTCCGTTCTATTTTATACGTCACATTTTTACTTTATACTTTTATTAAGAAATGATGTAGTTTTATCTTTCTATTCTTATTTAATGTTTTCTTAAGTCAATTTTATAATAAATAATGAATATATTTTCAAGATTAATTAACTACTCTATCAAGGGTATAATAGGTAAAATATGATAATTTATACATAAATTTTATAAAATGACAAGTATTGTGGTCCAACTATTTATAGAAAGAAATGATATATAAAATGGGACGGAGGGCGTTATAAAGTTGACTTAAGAAAACATTAAATAAGGGTAGAAGGGTAAAATTACATTATTTCTTAATGTAAATGTAAAGTAAAAAGGTAACATATAAAATGGAAAGGAGGGAGTAGTATTTTCTTGTTTTATTTTACGTGGCACTCTATTCTCATAATCCGTCTTTAAAAATGTCATTTTATTGTAATTGAAAATAATTTAACTTAAAATTCTCCATCTACCCTTAATTAATGAAATGATTTACAATTATATAAATATATAAAAATTGTTTTAGCCTATAATTTTCTAAAATCTTTTTTTTTCTCTTATACATCGTATTAAGTCAAACATAAATGGAATGGACGGAGTATTTCTTTTATTTTTTTGTCACACCGCCCATATGTTTTCTCCCATCCCCCAGACCCCCACTATGTATATTCACTCCTTAGTTGGATCTGAATTTAGAGTTTAGAAGCTTCTATAATAATTTTAGATTAATATATAATAATAATAATAATAATTGAACTTACAGTATTAAATTTATGTGAATCTATATATATTGTATTGTAATTTTTTTAATTATAATTTTAACCAAATCAATAAAGCTATTCAGATGTAAAAGTATATATTATGATTTAACAACAAATTTCTATACGTCTTCCTAAGTTTTGATGCATAATTTCCTAAAACTCATAAATTTCCAAGTGACTACTTCCAGTATTACAATGAGAACTTATGTTTCGTTATGGATTTTCTTAGTGAATTAGTTTAATAAAATCAAAATGAAAAAAAATCATGTTTTATAACATAAAATTTTCATTGATTCATGCGAAAAAAAAACATCTAGTTCTTATAGTGTGAAAACTATTGAACTTATGGGATGTAGCTGTATGGAAGTTCATCAAGTGGTAGCTCCTTGTACGCAACTAGTGCTACTTTTTATTGACTAAAAGTTATTTTCTAGA

>Rpi-abpt

TAAGACTTTTCTCTATATGTGTTTTTCCCCAAGTTGTATAATGGTTGTTGAAGATGCTTTAATTAAAAAAAAAAACCTTTTGTTTAGTGGAAAATTTCAAAAAGCTTTAGTACATCTTTGTCGTTTTATCCAATCGTAATTCTTTATTCAGAAACCACATGTTTTTTTTCTAATCTTACTTTTATGTCTATCACCCATTTTCCAATATACAGCCTACTCTTTTTTTCAATCAAAACTAGTATTCCTAAAGATGGCTGATGCCTTTCTATCATTTGCAGTTCAAAAATTGGGTGATTTCCTAATACAGAAAGTTTCCCTGCGTAAAAGTCTCAGAGACGAAATTAGATGGCTGATCAATGAGCTACTCTTCATACGGTCTTTCCTCAGAGATGCAGAACAAAAGCAGTGCGGAGATCAAAGAGTTCAACAATGGGTGTTTGAGATCAACTCTATTGCTAATGATGCTGTTGCTATACTCGAGACTTATAGCTTTGAGGCTGGTAAAGGTGCTAGTCGTCTCAAGGCTTGCACTTGCATATGTAGGAAGGAGAAGAAATTCTACAATGTTGCCGAGGAGATTCAATCACTCAAGCAACGAATCATGGATATCTCTCGCAAACGAGAGACTTATGGTATTACAAATATCAATAATAATGCAGGAGAAGGGCCAAGTAATCAGGTTACAAAATTGAGGAGAACTACCTCATATGTAGATGAACAGGATTACATTTTTGTTGGCTTTCAGGATGTTGTACAAACATTTCTAGCTCAACTTCTGAAAGCAGAGCCTCGTCGAAGCGTCCTCTCCATTTATGGAATGGGGGGTTTAGGCAAGACCACTCTTGCCAGAAAACTTTACACCAGTCCTGATATACTCAATAGCTTCCGTACACGCGCTTGGATATGTGTCTCTCAAGAGTACAACACAATGGATCTTCTTAGGAATATCATAAAATCCATCCAAGGTCGCACCAAGGAAACTCTAGATTTGTTGGAAAGGATGACAGAAGGAGATCTTGAAATTTATCTTCGTGATTTATTGAAAGAACGCAAATACCTTGTGGTGGTTGATGATGTATGGCAGAGAGAAGCATGGGAGAGTTTGAAAAGATCATTCCCGGATGGCAAGAATGGCAGCAGAGTCATTATTACCACGCGCAAAGAGGATGTCGCTGAAAGAGCAGACGACAGAGGTTTTGTTCATAAACTTCGTTTCCTAAGCCAAGAAGAAAGTTGGGATCTCTTTCGTAGGAAACTACTTGATGTTCGAGCAATGGTTCCAGAAATGGAAAGTCTAGCTAAGGATATGGTGGAAAAGTGTAGAGGCTTACCTCTTGCAATTGTTGTATTGAGCGGACTACTTTCGCATAAAAAGGGGCTAAACCAATGGCAAAAGGTGAAAGATCACCTTTGGAAGAACATTAAAGAAGATAAATCTATTGAAATCTCTAACATACTATCCTTAAGCTACAATGATTTGTCAACTGCGCTCAAGCAGTGTTTTCTCTACTTTGGTATTTTTCCAGAAGATCAAGTGGTAAAGGCTGATGACATAATACGGTTGTGGATGGCGGAGGGTTTCATACCCAGAGGAGAAGAAAGAATGGAGGATGTGGCTGACGGCTTCTTGAATGAACTGATAAGACGAAGCTTGGTTCAAGTAGCTAAAACATTTTGGGAAAAAGTTACTGACTGTAGGGTTCATGATTTACTTCGTGATCTTGCGATACAAAAGGCATTGGAGGTAAACTTCTTTGACATTTATGATCCAAGAAGCCACTCCATATCCTCTTTATGTATCAGACATGGCATTCATAGTGAAGGAGAAAGGTACCTCTCATCACTTGATCTTTCTAACTTGAAGTTGAGGTCAATTATGTTCTTCGATCCATATATTTGTAATGTGTTCCAACATATAGATGTGTTTCGACATCTATATGTGTTGTACTTGGATACGAATTTTGGGTATGTGTCTATGGTACCTGATGCCATAGGAAGTTTGTACCACCTCAAGTTGTTAAGATTGAGAGGTATCCATGATATTCCGTCTTCCATTGGCAACCTCAAGAATTTACAAACACTTGTCGTTGTAAATGGTTACACATTTTTTTGCGAACTACCCTGCAAGACAGCTGACCTAATAAATCTAAGACATTTAGTTGTTCAATATACAGAGCCTTTAAAATGTATAAACAAACTCACTAGTCTTCAAGTTCTTGATGGTGTTGCTTGTGATCAGTGGAAAGATGTTGACCCTGTTGATTTAGTCAATCTTCGAGAATTAAGCATGGATCGTATCAGGAGCTCTTACTCCCTAAACAACATTAGCAGCTTGAAAAACCTTAGCACTCTCAAATTGATTTGTGGAGAACGTCAATCATTTGCATCCCTTGAATTTGTTAATTGTTGTGAAAAGCTCCAGAAATTGTGGTTACAAGGGAGAATAGAGGAACTGCCTCATCTGTTTTCAAACTCCATCACAATGATGGTTCTGAGTTTCTCAGAACTGACAGAAGATCCGATGCCTATTTTGGGAAGGTTTCCAAACCTAAGGAATCTCAAATTAGATGGAGCTTACGAAGGAAAAGAAATAATGTGCAGTGATAACAGCTTCAGTCAACTAGAGTTCCTTCATCTTCGTGATCTTTGGAAGCTAGAAAGATGGGATTTAGGCACAAGTGCCATGCCTCTGATTAAAGGTCTTGGTATCCATAACTGTCCAAATTTAAAGGAGATTCCTGAGAGAATGAAAGACGTGGAGCTGTTGAAGCGGAATTATATGTTGTGAAGCTTTTCTGCCAAGCACATTGGTTATTAATTGAGTGGTTTTAGTGTTGATTTCTTATTATTGTTTTAAGCTTTTTGAGTGTGTAATTGGTTTGAACATTATTGTTTTAATTAATTGGTCTACTGTATGTTCTCATGCTTATCCACATTTAAGACAATGCTTTATATGTTAAAATGAAATTAAAAATACTAGTATATGGTACTCTCTCTTGTCCACAATTTCGTATATTTTTTGTTCCTCTTCATAAAAA

> *Rpi-abpt^T86^*

TAAGACTTTTCTCTATATGTGTTTTTCCCCAAGTTGTATAATGGTTGTTGAAGATGCTTTAATTAAAAAAAAAAACCTTTTGTTTAGTGGAAAATTTCAAAAAGCTTTAGTACATCTTTGTCGTTTTATCCAATCGTAATTCTTTATTCAGAAACCACATGTTTTTTTTCTAATCTTACTTTTATGTCTATCACCCAATTTCCAATATACAGCCTACTCTTTTTTTCAATCAAAACTAGTATTCCTAAAGATGGCTGATGCCTTTCTATCATTTGCAGTTCAAAAATTGGGTGATTTCCTAATACAGAAAGTTTCCCTGCGTAAAAGTCTCAGAGATGAAATTAGATGGCTGATCAATGAGCTACTCTTCATACGGTCTTTCCTCAGAGATGCAGAACAAAAGCAGTGCGGAGATCAAAGAGTTCAACAATGGGTGTTTGAGATCAACTCTATTGCTAATGATGCTGTTGCTATACTCGAGACTTATAGCTTTGAGGCTGGTAAAGGTGCTAGTCGTCTCAAGGCTTGCACTTGCATATGTAGGAAGGAGAAGAAATTCTACAATGTTGCCGAGGAGATTCAATCACTCAAGCAACGAATCATGGATATCTCTCGCAAACGAGAGACTTATGGTATTACAAATATCAATAATAATGCAGGAGAAGGGCCAAGTAATCAGGTTACAAAATTGAGGAGAACTACCTCATATGTAGATGAACAGGATTACATTTTTGTTGGCTTTCAGGATGTTGTACAAACATTTCTAGCTCAACTTCTGAAAGCAGAGCCTCGTCGAAGCGTCCTCTCCATTTATGGAATGGGGGGTTTAGGCAAGACCACTCTTGCCAGAAAACTTTACACCAGTCCTGATATACTCAATAGCTTCCGTACACGCGCTTGGATATGTGTCTCTCAAGAGTACAACACAATGGATCTTCTTAGGAATATCATAAAATCCATCCAAGGTCGCACCAAGGAAACTCTAGATTTGTTGGAAAGGATGACAGAAGGAGATCTTGAAATTTATCTTCGTGATTTATTGAAAGAACGCAAATACCTTGTGGTGGTTGATGATGTATGGCAGAGAGAAGCATGGGAGAGTTTGAAAAGATCATTCCCGGATGGCAAGAATGGCAGCAGAGTCATTATTACCACGCGCAAAGAGGATGTCGCTGAAAGAGCAGACGACAGAGGTTTTGTTCATAAACTTCGTTTCCTAAGCCAAGAAGAAAGTTGGGATCTCTTTCGTAGGAAACTACTTGATGTTCGAGCAATGGTTCCAGAAATGGAAAGTCTAGCTAAGGATATGGTGGAAAAGTGTAGAGGCTTACCTCTTGCAATTGTTGTATTGAGCGGACTACTTTCGCATAAAAAGGGGCTAAACCAATGGCAAAAGGTGAAAGATCACCTTTGGAAGAACATTAAAGAAGATAAATCTATTGAAATCTCTAACATACTATCCTTAAGCTACAATGATTTGTCAACTGCGCTCAAGCAGTGTTTTCTCTACTTTGGTATTTTTCCAGAAGATCAAGTGGTAAAGGCTGATGACATAATACGGTTGTGGATGGCGGAGGGTTTCATACCCAGAGGAGAAGAAAGAATGGAGGATGTGGCTGACGGCTTCTTGAATGAACTGATAAGACGAAGCTTGGTTCAAGTAGCTAAAACATTTTGGGAAAAAGTTACTGACTGTAGGGTTCATGATTTACTTCGTGATCTTGCGATACAAAAGGCATTGGAGGTAAACTTCTTTGACATTTATGATCCAAGAAGCCACTCCATATCCTCTTTATGTATCAGACATGGCATTCATAGTGAAGGAGAAAGGTACCTCTCATCACTTGATCTTTCTAACTTGAAGTTGAGGTCAATTATGTTCTTCGATCCATATATTTGTAATGTGTTCCAACATATAGATGTGTTTCGACATCTATATGTGTTGTACTTGGATACGAATTTTGGGTATGTGTCTATGGTACCTGATGCCATAGGAAGTTTGTACCACCTCAAGTTGTTAAGATTGAGAGGTATCCATGATATTCCGTCTTCCATTGGCAACCTCAAGAATTTACAAACACTTGTCGTTGTAAATGGTTACACATTTTTTTGCGAACTACCCTGCAAGACAGCTGACCTAATAAATCTAAGACATTTAGTTGTTCAATATACAGAGCCTTTAAAATGTATAAACAAACTCACTAGTCTTCAAGTTCTTGATGGTGTTGCTTGTGATCAGTGGAAAGATGTTGACCCTGTTGATTTAGTCAATCTTCGAGAATTAAGCATGGATCGTATCAGGAGCTCTTACTCCCTAAACAACATTAGCAGCTTGAAAAACCTTAGCACTCTCAAATTGATTTGTGGAGAACGTCAATCATTTGCATCCCTTGAATTTGTTAATTGTTGTGAAAAGCTCCAGAAATTGTGGTTACAAGGGAGAATAGAGGAACTGCCTCATCTGTTTTCAAACTCCATCACAATGATGGTTCTGAGTTTCTCAGAACTGACAGAAGATCCGATGCCTATTTTGGGAAGGTTTCCAAACCTAAGGAATCTCAAATTAGATGGAGCTTACGAAGGAAAAGAAATAATGTGCAGTGATAACAGCTTCAGTCAACTAGAGTTCCTTCATCTTCGTGATCTTTGGAAGCTAGAAAGATGGGATTTAGGCACAAGTGCCATGCCTCTGATTAAAGGTCTTGGTATCCATAACTGTCCAAATTTAAAGGAGATTCCTGAGAGAATGAAAGACGTGGAGCTGTTGAAGCGGAATTATATGTTGTGAAGCTTTTCTGCCAAGCACATTGGTTATTAATTGAGTGGTTTTAGTGTTGATTTCTTATTATTGTTTTAAGCTTTTTGAGTGTGTAATTGGTTTGAACATTATTGTTTTAATTAATTGGTCTACTGTATGTTCTCATGCTTATCCACATTTAAGACAATGCTTTATATGTTAAAATGAAATTAAAAATACTAGTATATGGTACTCTCTCTTGTCCACAATTTCGTATATTTTTTGTTCCTCTTCATAAAAA

>Virus-Rx

TGCGCAATTCTTACTGTTTTATACCTTTTCTCCCTCTCTTCCTCTCCATCATTTTCAGCCTTATGGATATTATTGTTATGTTCCTCATGAGTATCAACATGCATATTCTTTTCTCCAAATTGTTGTTCATCTCTTACAATATTATTATCATAAATTTCACCAACATTCGTATGATTTCGGCCATTTGCTTCTAGACCTTTTATCGATTTCTCCCTTTGTATTTTTTCTCTCAATACAAGTTGCCATAAGTATTTCACAATTTCCTTGTTGAATGGATTTTCCAAGCAAAAATAAGCTCCATTTTCCAAAGCGTTCTTGGCTAAGAGTCTATTGAGTTTATCACCTACAACTGCATAATTATATTGAGAAGTTAAAAATTAAAATTAAATTATATCATACATTATGAGAATAGTTAATTTCACACATATATTTACTGAATTTTATCTGTTATGACAACAACTTTGTTATGAAACAATCATTTATCATATAACTATAACTAAAAACAAGATTAACAATGGATAGATGTCGTCTCTTGACAATAGATTAACGATAAATTTTATAGCTAATTATATTTTTGTGGTGTATTAATTGAAACAACTGACTTTACCCAATATTTATAACAAGAAGTGAAAAATAGAATTATAAATACTTACCAAGAGAGATTATATTTAAGGTTACAGCTTGTGTCAAGAGTTCAAAACAATGTAAATCTGATGAATTGACATCGATTATCATCATGTCAATTTTTTTCTTTTCTTTGGAGAGCATTGACATTGCCGCAGGAGTCGTAACTGTCGTAACTGTATATATCCATACATTTTAAAGTTAAAGTTAGTGAAGAACATGAAAAAAATATTATACACAAAGTTATCATTTGGATATGTTTATTTAATTTCTCATTCAAATTCAATTATGAAATTTCATATTATGTTGCTATACTTTAATTTATGAAATAACACATATTAATCAACTTATATCGTAAACGGTGTGTGTAATGACCCGAAAGGTCATTTTGGTAATTTTTCGTGGAAATGACTATTTTGCCCTTTCGGTAGTTGCCCCGAGTCCCATGTTTTATGGTTGTAAAGTTAGTTTGAGGAAAAGTTGGTAAAAAGTTGAGTTTTTGAGAAGTTGAGTTTTTATGAGTTAAAGTTGGTTAAAAAGTGCTTTTTCGAGTTATTTGGAGTTTCGGGACTCGGATTGAATTTCCGTCGATTCCGGCAATTTTAGAATGTCGAAACGGGTCTGTGTGAAGTTACGGAGTTGAATTTGGAGTTGAAACGAAGAATTGAGGTCCTAAGTTGCTAAAGTTGTGAAATTGTGATAAAGAGTTGACTTTGGTCAACATATGAGGTTCCGAGGCTCGGATTGAAATTCCGAAAGTACCGTTGGTTTCGGGAGGCGATTCTAAGTCTAGAATGAGTCTTGGTTGAATTTTTAGACGCTCTGTTTGAGTTTCGAATTTTTTTTGCGTTAAACTAGTTTCTTGGCATCTTATTGAATTTCGGGTCAAGGAGTCCTCGAATTCAAATTCCGACGGTTCCATTGAGTCCGGAACGTCGAATTTAGTGGGGGTACATATTTAGTTTGTGTGCACGAGATTCCGAACGAATCCCGAGGGTCCAACGGGGACTTAAACTTTGGTGTTATTGTTGTGTTTCAGCTGCAATAGCAGTCCTTTTTAAGTCAAAAAACAGTAGCCATTTTCCCCCAACTTGAAAAATATCCATTTCTACATTTTTAGACTTTGAGAGCTCGAAAATAGGTGATTTCAAGTGGGTTTTCACAAGGAATTCATTGGGTAAGTTATTTCCAACTTATATCTTTGATTTCCATCGATTATTTATGGATTTTAACATCAAAATTTGAGATCCCAACTGAAAAATTTGGGGGTTTTTGCCTAGTCCGAATTTAATAGAAAATTTGAGTTTGTGAACTCATTTCAACTCCTTTTTCGAAACGGTTTTCACTATTGGATTCTAAATTTCTTGGGGAACATTTTTCACTAAAAATTTCCAGATTTCGAGAATATTTTTTCGGAACGAGTTTTTTTTGACCATTTTACCCTTTTCTCCAAATTTCACGATATTGGTGTTGTTAGATTCGTATTGATTGTGAGATTCCATTTATGAATAGATTGTGGTGATTTGGAGCGTGCTCGAAAGGGTAAAACTCAAATTTGAGTTACTAGTTGAAGTGTTTTGAGGCAAGTGACTTCTAAACTTTGTTAAACTCTTAGACTACACATGACTACTTTCCTAATTGTGTTGGGGAGTAATGGGGATTGAGGATGGGTTTTATTTGTTGATTGAAATTGTTGTAAATGAAAGATGGGGAATAAAACGAGCTAAGTGTGTTATATGTGACTTGAATTTGTTGAATAAGTCATGTGATAACCGATATTGAGGGGATAGAAGAGCATGAGTAAGCTATGATTGATACAGACATTGATGTTGAGACAGATGATGTGTAATACTATGATATGGTTGTGATTGAGACAGACGATGTGTAATACTATGATGTGGTCGCGATATGGTTGTGATTGAGACAGATAATCTGTAATACTATGATGTGATCGTGATATGATTGTGATTGATGACATGTGCATATTCATTATTCATCTCATGTGTGAACTATCTGTTGCATGAGTTCTGAGACACTGATATGAGGATGGATGGCTATGAGACACAGTTGAGACTAGCTCCGGCTAGAGATATATGAGATGGACTAGCTCCGGCTAGCGATTTGGATGCCGATGGGATCTGGTTCCGGCGGTGATACATGGTCCATGTGTGGCCCCCATGGGTTCTGATTTGAGTATTCAGCGCGGACTGATTACGTCAACAGATATGTATCGTAGGACAGACATGCATCATGACTATATGACATTATTATTGCATTTGCATCGCATTTGACTTCATCTTTGTCTGTGGTGTGTGGAGTTTACCTATTTGCCCCTCTTCATGTGATATATGATCTATTTGCTCTTACATGAGGATATGAGGTTGATGTTGAGACACTGTTGGATATATCTGTTGATTATGAATTGTGTAGTATAGGTTGTTGGTTCGCTGTTAGAATGAAGTCTCGGTGGTTCGGTTGGGATTGAAAGGAGTTGTTTGTAGCTGCTAGTTTAGCTTAGTTTAGTGTTACTTGCGAGTACCTGTTGCTTTTGGTACTCACCCTTGCTTCTACACAGTGGTGTAGGTTGACAGCTCTATCAGATTCGACTTATTACTCATCTTCAAATTAGAGCTTCCAGACTTACTTGAGAGGTAGCGGTTCATTCCAGACGTGCCCTCAAGTTATCTATATTTATTGTTTTGTTCTATTATAGAACTTTACTATGAGACTTGTATATTTTATTCAGATTCCGTACTAGAGGTTTGTACATGTGACAACCAATTCTGGGGTTTCATTGAGTTTATTTAAAGACTTCCGCTTATCTTTCTATCTTATTCTTGTTTTATTAATTCCGTATCGTCGGGTTTTTGGGTGTTAGGCATCACATCCGGTTTTGGAGTGTGACAGTGTGTGCAATCTTACTAATATATAATATTACTTTAGAGTAACTAACTGTAGGAATATATAATAGAGAAGATTCTATAGGAATAATTCTAAAGAATACATTCTAATTTATTCTTTGACTTGTTGAAAACAAATAAACACTGAATATATATTAGATTAAATACTATTAAAGCAAGATATAATAGGATAAAAAGTTTTAAGATAAATTTATGTAAAAAGTTTTAAGGTTATTTTATTTCATAAATAAGTAACGAGAGTTTAGGAGATTTAACAAATTATTAGAAAGGAGTTACCAAAATTATGTTGAAAAGGTTTAACATAGCTTGATTAAGGAACAATCGATATTAAATACTAACAAAGTAATTGAAAATGAAACAAAAATAAATGAGGAAAAAATCACATGAAAAGATTTTTCTTTTCTTGTCTACATCTAATAAAAGACAAAAAAGAAATTGAAGAAAAAAATCATGAAAATCATATGTCCCTATTTATTTGTTTGTCTACATCGAATAAAAGACAAAAAAGTCATCTCAGATAATAATGATAGTCAGATCATCTCTTATCATGTCTTTATCTCGCTCCTCTCTGTTGGGTTCTAAAGGGTGATTAGGGCTTGCAAATAATATGATTGATAAATTAGTTGACAAAAACTTATACTAAAAACTAATATTATTATATGAAAACTAATATAAAGAAAAATATTAAAACTGTTGAGAGATTAAATCTCCCCATAAACAAAACTCACTAAACGACTACATTGTGGATTCTATTATGTTGTGCTATGAGAATAGGGATCTTCAATTTATAGATCTCCAAATTTTTCCTCTAAGGAAAGAATAAACCAAATATGGAAGAAAATTATATTTTTCTTTTAGAAAAAGTTCAAGCATTTATGGTAATACTTTGACTTTTCTTTAAAGAAAAAATAAACTTCAAATAAGATAAGAAAATCAGGGCAAAACCCTAACACTCTCTTCGATCCCTTACTCTTATTTTGTGCCTCAATCATCCAAGTGAATTTGTTTTCTTTCTAAAACGATATATAGATTTGCAAAAATCAAAGATCTGTAATTTCAAAATCATTTATGAGGGACCTAATTATTTTCTCCTCCAACACGATGTCACTCTAGGCCGATTGACAAAGAATAAGTTTCCTTTTTATTTTTTGTACAAAAAAGTCAAGTGTGAAATAATTAATATAAACTTAAACTACCTCCTTTATGGAAAAATATCCGATAAAATTAGAACAACACATACTTTAACATAAAATTATTGAACCTCTTAAAAATTACACAATCGTTATGGAGAAAGCTAATCGAGTATGTGACATGGTTATAACAAAAACAAAAATGGAGAATCTAATTTAATTCCTTATAATCATACAAAATTCATTTATTAACTTGTAAAACAACATACACAAGCATAGAAATAATGTTATGATAACAAGGAAAGGAAAAGTTCTCAATAAACATCATATCAATTAATTCTCTCTATATATAATGAAAGTCAGACAGAAATTAGTTCTAGATATATCACCTTTGTATCCATTGAACTCTAGTAAATCAACCATTGGATTAGAAACCAATTCCTCATCTTGATATACCAGCAACACAGAAACCTCTTCAGTCAAATCATAAAAAGGTTCTGTCATTAGATTAATTTGAATTTGTAAGAAAAACACTTACAACTGAAGCTATTTATAAAGTATCATTTACTGGCTTATGAGCCTGTTTAGAGATAATTATATATAAATAATTTTATCTAAATATCTAGATAATCCCTCAAATTTGACACAAATTGTAATTTAGATACTTGAATTTATCTAAAAGAGACCAAATAAATACTTTTACTATTGTCATATACGCACTCATGAACACCTTATGCTGACATGGCAAATTTATGTGTATTGTAAATGCTTGTGTTATTACATTATTGAATCACAATAAAAAGATTATTTCTTAAAAAAATTAAATTTTTGATTGACTTTTATTCCAACAATCAAATTATACAACTTGGGGTTCATTAACTATTTCTATTAATATCAAAATTTAACATTGATTATTGGATATTATTTGAAGTTATTGAAGATGACATTATATTTATTAAGTATTAATTACACTTCTTGAATAGTTGGAAGATTCTTTGATTTGTGTCCATAATTCAATAGTTCAAATGGTGAATGTCTTGTCTTCAAGTAACGGCTAATGGTCTTAGAATTAGGCCTTATTTATTTTTGTTCGGATAAATTAGCCCAGAGATATTCTAAATAGAGTATGATATTGTGCACTTTGACCAAGTTAGCATAATTTTTTTTTTTTCAAAAAGTCTCACACATTATGGAAAAATTACGCGAGTTGGCAAATACTACTAGGTTATTAGTCATTGAGGGTAGGGTATAGTTTAATTTATTTACTGTCAGTAAATACAGTTTCAGTTTTTTTGCCATTAATAATGAGACCCACCACCTCTTTGTATACCTGTCACACCCCAAAATCGGATGTGATGGCACGTGCCCAAACCCCACCGGACACGTCAGCCTAACACCCAAAAACCCGACGATACGAAATTAATAAAACAAGAATAAGATAGAAAGATAAGCGGAAGTCTTTAAATAAACTCAACATAACCCCAGAATTGGTTGTCACATGTACAAACCTCTAATACGGAATCTGAATAAAATATACGAGTCTCATAGTAAAGTTCTAGAATAGAACAGAACAATAAATATAGATAACTTGAGGGCACGTCTGGAATGAACCGCTACCTCTCAAGTAAGTCTGGAAGCTCTAATCTGAAGATGGGTAATAAGCCGGATATGACAGAGCTGTCAACCTACACCACTGTGTAGAAGCAAGGGTGAGTACCAAAAGCAACAGGTACTCGCAAGTAACACTAAACTAAGCTAAACTAGCAGCTACAAACAACTCCTTTCAATCCCAACCGAACCACCGAAACTTCATTCTAACAGCGAACCAACAACCAATACTACACAATTCATAATCAACAGATATATCCAACAGTGTCTCAACATCAACCTCATATCCTCATGTAAGAGCAAATAGATCATATATCACATGAAGAGGGGCAAATAGGTAAAGTCCACACACCACAGACAAAGATGAAGTCAAATGCGATGCAAATGCAATAATGATGTCATGTAGTCGTGATGCATGTCTGTCCTAAGATACACATCTGTTGACGTAATCAGACCGCGCTGAATACTCAAATCAGAACCCATGGGGGCCACACATGGACCATGTATCACCGCCAGAACCAGATCCCATCGGCATCCAAATCGCTAGCCGGAGCTAGTCCATCTCATATATCTCTAGCCGGAGCTAGTCTCAACTGTGTCTCATATCCATCCATCCTCATATCAGTGTCTCAGAACTCATGCAACAGATAGTTCACACATGGGATGAATAATGAATATGCACGTCATCAATCACAATCATATCACGATCACATCATAGTATTACACATTATCTGTCTCAATCACAACCATATCACGACCACATCATAGTATTACACATCATCTGTCTCAATCACAACCATATCACGACCACATCATAGTATTACACATCATCTGTATCAACATCAATGTCTGTATCAATCATAGCCTACTCATGCTCTTCTATCCCCTCAATATCAGTTATCACATGACTTATTCAACAAATTCAAGTCACATATAACACATTTAGCTCGTTTTATTCCCCATCTTTCAATTACAACAATTTCAATCAACAAATAAAACTCATCCTCAATCCCCATTACTCCCCAACACAATTAGGAAAGTAGTCATATGTAGTCTAAGAATTTAACAAAGTTTAGAAGTCACTTGCCTCAAAACACTTCAACTAGTAACTCAAATCTGAGTTTTACTCTTCCGAGCACGCTCCAAATCACCACAATCTATTCATAAATGGAATCCCACAATCAATACGAATCTAACAACACCAATATCGTGAAATTTGGAGAAAAGGGTAAAATGGTCAAAAAAAATCTCGTTCCGAAAAATATTTTCGAAATCTGGAAATTTTTAGTGAAAAATGTTCCCCAAGAAATTTAGAATCCAATAGTGAAAACCGTTTCGAAAAAGGAGTTGAAATGAGTTCACAAACTCAAATTTTCTATTAAATTCGGATTAGGCAAAAAACCCCAAATTTTACAGTTGAGATCTCAAATTTTGATGTTAAAATCCATAAATAATCGATGGAAATCAAAGATCTAAGTTGGAAATAACTTACCCAATGAATTCCTTGCGAAAATCCACTTGAAATCACCTATTTTCGAGCTCTCAAAGTCTAAAAATGGAGAAATGGGTATTTTTCAAGTTGGGGAAAATGGCTACTGTTTTTTTTGACTTAAAAGGGCTGCTATTGCAGCTGAAACACAACCATAACACCAAAGTTTAAGTCCCCGTTGGACCCTCGGGATTCGTTCGAAATCTCGTGCACACAAACTAAATATGTACCCCCACTAAATTCGACGTTCCGGACTCAATGGAACCGTCGGAATTTAAATTCGAGGACTCCTTGACCCGAAATTCAATAAGATGCCAAGAAACTAGTTTAACGCCAAAAAAATTCGAAACTCAATCGGAGCGTCTAAAAATTCAACCAAGACTCATTCTAGACTTAGAATCGCCCCCCGAAACCAACGGTACTCTTGGAATTTCAATCTGAGCCTCGGAACCTTATATGTTGACCAAAGTCAACTCTTTATCAAAATTTCACAACTTTAGCAACTTAGGACCTCAATTCTTCGTTTCAACTCCAAATTCAACTCCGTAACTTCACACAGACCCGTTTTGACATTCTAAAACTGCCGGAATCGACGGAATTCCGATCCGAGTCCCGAAACTCCAAATAACTCGAAAAAGCACTTTTTAACCAACTTTAACTCATAAAAACTAAACTTCTCAAAAACTCAACTTTTTACCAACTTTTCCTCAAACTAACTTTACAACCACAAAACATGGGACTCGAGGTAACTACCGAAAGGGCAAAATAGTCACTTCCACGAAAAATTACCAAAATGACCTTTCGGGTCATTACAATACCGAATCAACAAAGCCTTCATCGGCCTTTATTTATGATTTTATACAAAATCAAATTTATCTCTCATCTCCTCTCCTCTCCTCATTTCCTATTTTTCTCCTAAAATAACTCTTTCACTTCCAATTTGAAATTCGAATTTCACTTTCTCCCTCCAGTCGTGTTTTACTTTTTGCAGGTAACCGCCTAAATCATAGGTGAACCTCTTTTTTTTTTTTTTTTCATTTTTCAATTGTATATGTATTGTGCTTGTTAGTGTTTGGATAATTTTTTCTCAAAATTCCCTCATTACTTATTAGATAATCATTTTTATAGAAAATAAAATGGATGATTCGACTAGAGGATTATGAGAGGTACGTCATTGCATGTCAAAGTTTTTAATAATCAACCTTCATTTTCACTCTGTTTAACTCAAGATTTTGGAGTCAATGCAGGGTCTATGGAAAAATCTAAACAAATTCTAGAGTAAAAATCTATTGAAGAATTGAGATCCAAGAAAAAAATGACCCAATTGCACTTCCAGAAGTCATCAACAATGTCAAGGCAAGCGACATTAAAATTGTTGAAGGAGAAAGTAAGAGGAAAGCCAAAGATAGTGATTCTGAGGAGGTTGTGTCTCCTTCATTAGATCAAGACAAATACGAAGAACATCAGGTATTTGCTTAAAACACAATTGTATGTAGATATTTGTATATTTTGTTAGTGATATACAAAATTGTATGTAGTATATGTATATTTTCTGCTTACATCACAATTGTATATAGATATTTGTATATTTTGTTAGTTATATACAAAATTGCTTGAAGTATATGTATATTTTTTGCTTAAATCATAATTGTATATATATATTTGTATATCTTGTAAGTTATATACAATAGTATGAATTAAACAATATACAAACCTTACATTATTATATATACAGTTAGGTTACACCAAAAATTATCAAATTAAAGCACAACTTTTTTATCGAATCATATACAATTCATATATATAATTGACTTAGTAATTTTATACAACTACTTACACTTATACATGGTATAAGAATTTTGCACAATTACTTACATATATACAATATTATCAATTAAACAATATACAAATCGTATAACTTATATATACAGTAAAATTACAACAACAACAACAAAAATTATCAAATTAAAGCACACCGTTGTTGTCGAATCATATACACTTCATATATAAAAATTGTGTCATTCAATTTTTCGAACAAAAAATTAGAATTGAATTGGTAATAAAAAATTTATCTAATCTTGTATAAACAAAATTAAATTATTGCAAACCATTAGAATGAAAAAAACAAAAATAATCCGTTTTCCAAAATTTCAATTATATACTATACAAATCAATTGTATACTTTCTTGCTGTTCAAAACATGAAGTTTCCTTGAAAGAAACGCTTACCTAGCGTTGAATATACAAGAATATTGATTAATCTTATGCTTCAGTCGTTTGAGGAACCCAGTTGTTATGGTGTTTCTATTGCTATAGAACTCCTTTTTGGAAAAATATTTGATTTTGGACGATTAGCTTGAACATTGGGACTATATAAATTTTTTATTACCGTATTTAGCACTCATGTATCCATTTATTAAAAAAAAATGTATAAATTATATTTTTAAAAGAAAATATACAAAATTAATGCTTCATAGCAAACTAAACTATGCCCATTGAATGTAATTACTAAACTATACCTATAGAGCGTTATTTCATTAAATACGTTTATCATATATGAAATTTTCCCTTAAGAGATCCTACACCTTATATATAGCTTCTCAAATGTGGAAATTCAATCTCACACCCAACAATCTTTCCCTCAGACTAAGTTTCATGGCCCAATATCACAATGATCCACGAGTCAATTCATGAGATTCACTATGTGCATCACCCACATCGTCTAAGTATTTTATGGCAATCAAGCCCTACAACTAGCTTCTTCTTTATATATATATGTGTGTGTATATGTGCGCGCGCGCGCATCTCTAATTAATCTCGTAAAGGGATTAAGGGGCCAATTTCAAAGAATTAGGCGATTTTCTTAGTTTTTCGTGTGTGTTAACCCATAAATATTTTGGTGATATGGTTTTCGGACGATTTCTTTTGTGCAACTTATATGGAACCCTTCGTAGGGAGTTAGTCTCACACTTTTTAGAGTCCATTTTGGGCACTCAGGGGCTAATTTATAGGAAATAGGTGATCTTCTCAGTCCGTCTGTATTAGCCCATGAATATTTTGGTGATATGTCTTCCGAATAATTTCTTTGTAAAATCTTTACGGGACCCTCCATAGGGAGTTAATGGAGCAGTACGTATAGTCTCACAATTTTAGAGTTCATTTTGGGCATTTAGGGGCCAATTTACAGGAATTAGGTGACTTTCTCAGTGTTTTGTGTGTGTTAGCCCATTAATATTTGGTGATATGACTTTCAGACGATTTCTTTGCTACACATTTACGAAACCCTCTGTAGGGAGTCGGGGGAGCAGTACGTACAATCTCACAATTTTAGAGTCCATTTTAGACATTTAGGGGCCAATTTAAAGAAATTGGACAATTTTCTCAGTTTTTCGTGTCTGTTAGCCATTAATATATTGGTGAATATGACCTACGGATGATTTCTAATAGAAATCTTTACGAAACCTTCAATAGGGAGTTGGGGGAGCAATACGTACCGTCTGACAATTTTTAGAGTCCATTTTGGGCATTTAAGGGCCAATTTACAGGAATTAGACAATTTTCTCAGTATTTTTCCATGTGTTAGCCCATAAATATTTTGTTGCTTTGACTTTTAGAGTCTAAACTTCTCATGTATATTAAGAGATATTTATGCTTGGTTAATTGAATCGAACTAGGAATAGAGAAATTCCTACTTGGATCTTAATATTTCTCTCTCTTTGATTTGGAAAATTCTACGAAGTTGCTTTCAATGGAATTAAAATCATAAATCTATTGTATGTAAGAAACATACTTATATTCATGAATAGATATGTGTAGGGTCTAATAATGAATTATCACAATTTTTTCTACTTTTTCCTGTCAGAGTCCTGCTTTTTCTTTTTCTTTTTCTTTTTTAACTTTGGTCTCTGCTTTTGTCTACATGATGATAAGGTTGGTGGACCTAGCTGGAAATGTGATGGAAATAGCTAGTAAAAGAAAGAACTTTGCATTTTCTGTTTTCTTAAAAACTGAAAAATTACATAACTTGTGGCAATTTGTTCATTTTCATACTGAGAGATATTTCTATTTTTTGGATATATGGCTTATGCTGCTGTTACTTCCCTTATGAGAACCATACATCAATCAATGGAACTTACTGGATGTGATTTGCAACCGTTTTATGAAAAGCTCAAATCTTTGAGAGCTATTCTGGAGAAATCCTGCAATATAATGGGCGATCATGAGGGGTTAACAATCTTGGAAGTTGAAATCGTAGAGGTAGCATACACAACAGAAGATATGGTTGACTCGGAATCAAGAAATGTTTTTTTAGCACAGAATTTGGAGGAAAGAAGCAGGGCTATGTGGGAGATTTTTTTCGTCCTGGAACAAGCACTAGAATGCATTGATTCCACCGTGAAACAGTGGATGGCAACATCGGACAGCATGAAAGATCTAAAACCACAAACTAGCTCGCTTGTCAGTTTACCTGAACATGATGTTGAGCAGCCCGAGAATATAATGGTTGGCCGTGAAAATGAATTTGAGATGATGCTGGATCAACTTGCTAGAGGAGGAAGGGAACTAGAAGTTGTCTCAATCGTAGGGATGGGAGGCATCGGGAAAACAACTTTGGCTACAAAACTCTATAGTGATCCGTGCATTATGTCTCGATTTGATATTCGTGCAAAAGCAACTGTTTCACAAGAGTATTGTGTGAGAAATGTACTCCTAGGCCTTCTTTCTTTGACAAGTGATGAACCTGATGATCAGCTAGCGGACCGACTGCAAAAGCATCTGAAAGGCAGGAGATACTTGGTAGTCATTGATGACATATGGACTACAGAAGCTTGGGATGATATAAAACTATGTTTCCCAGACTGTTATAATGGAAGCAGAATACTCCTGACTACTCGGAATGTGGAAGTGGCTGAATATGCTAGTTCAGGTAAGCCTCCTCATCACATGCGCCTCATGAATTTTGACGAAAGTTGGAATTTACTACACAAAAAGATCTTTGAAAAAGAAGGTTCTTATTCTCCTGAATTTGAAAATATTGGGAAACAAATTGCATTAAAATGTGGAGGATTACCTCTAGCAATTACTGTGATTGCTGGACTTCTCTCCAAAATGGGTCAAAGATTAGATGAGTGGCAAAGAATTGGGGAAAATGTAAGTTCGGTCGTTAGCACAGATCCTGAAGCACAATGCATGAGAGTGTTGGCTTTGAGTTACCATCACTTGCCTTCTCACCTAAAACCGTGTTTTCTGTATTTTGCAATTTTCACAGAGGATGAACAGATTTCTGTAAATGAACTTGTTGAGTTATGGCCTGTAGAGGGATTTTTGAATGAAGAAGAGGGAAAAAGCATAGAAGAGGTGGCAACAACATGTATAAACGAACTTATAGATAGAAGCTTAATTTTCATCCACAATTTTAGTTTTCGTGGAACAATAGAAAGTTGTGGAATGCATGATGTGACCCGTGAACTCTGTTTGAGGGAAGCTCGAAACATGAATTTTGTGAATGTTATCAGAGGAAAGAGTGATCAAAATTCATGTGCACAATCCATGCAGCGTTCCTTTAAGAGTCGAAGTCGGATCAGAATCCATAAGGTGGAAGAATTGGCTTGGTGTCGTAACAGTGAGGCTCATTCTATTATCATGTTGGGTGGATTCGAATGCGTCACACTGGAATTGTCTTTCAAGCTAGTAAGAGTACTAGATCTTGGTTTGAATACATGGCCAATTTTTCCCAGTGGAGTACTTTCTCTAATTCATTTGAGATACCTATCTTTGCGTTTTAATCCTTGCTTACAGCAGTATCAAGGATCGAAAGAAGCTGTTCCCTCATCAATAATAGACATTCCTCTATCGATATCAAGCCTATGCTATCTGCAAACTTTTAAACTTAACCTTCCATTTCCCAGTTATTATCCTTTCATATTACCATCGGAAATTTTGACGATGCCACAATTGAGGACGCTGTGTATGGGCTGGAATTACTTGCGGAGTCATGAGCCTACAGAGAACAGATTGGTTTTGAAAAATTTGCAATGCCTCAATCAATTGAACCCTCGGTATTGTACAGGGTCTTTTTTTAGACTATTTCCCAATTTAAAGAAGTTGCAAGTATTTGGCGTCCCAGAAGACTTTCGCAATAGCCAGGACCTGTATGATTTTCGCTACTTATATCAGCTCGAAGAATTGACATTTCGTTTATATTATCCATATGCTGCTTGCTTTCTAAAAAACACTGCACCTTCAGGTTCTACGCAAGATCCTCTGAGGTTTCAGACGGAAATATTGCACAAAGAGATTGATTTCGGGGGAACTGCACCTCCAACTTTACTCTTACCTCCTCCGGATGCTTTTCCACAAAACCTTAAGAGTTTAACTTTTAGGGGAGAATTCTCTGTGGCATGGAAGGATTTGAGCATTGTTGGTAAATTACCCAAACTCGAGGTCCTTATACTATCATGGAATGCCTTCATAGGCAAGGAGTGGGAAGTAGTTGAGGAAGGGTTTCCTCACTTGAAGTTCTTGTTTCTGGATGATGTATACATTCGATACTGGAGAGCTAGTAGTGATCACTTTCCGTACCTTGAACGAGTTATTCTTAGAGATTGCCGTAATTTGGATTCAATCCCTCGAGATTTTGCAGATATAACCACACTAGCTCTTATTGATATAGATTACTGTCAACAATCTGTTGTGAATTCCGCCAAGCAAATTCAACAGGACATTCAAGACAACTATGGAAGCTCTATCGAGGTCCATACTCGTCATCTTTTGTAAGACATCTTCTTCCTTGCTTTACAACAATAATTAACTCATCATCATAGTAAACTCGATAATAATCTGGATAATAGCCTTAGTAAGTCAAATTGCACCAATTCAACAAAAGTTCTTGATGCTGTCATTGTGTTTGATTTGAATCCTTCCAATATTGTGTAACTTGTTATACTTGCATGTTCATTCTTGATTTTGGGAAGTGTAACATTTCATTTTTCATCTTTTGTGGCTAGCATTCCCAAGAGTGTGACAACAGTTGAAGATGATGATGATAGTGTGACAACAGATGAAGATGATGATGATGATGACTTTGAGAAAGAAGTTGCTTCTTGCCGCAATAATGTGTAAGTTCTTATACCTGCATGCTCATTCTTGCTATAATGTTCTCTTGTTCCTTAATTATGGGACATCTGACATATTATTTTCCATGTTTTGCGTCTTTTATTTTTCTGCAGCGAGTAGTTAAGGTGTTCTGAGGACTAGCCAGTTCTCTGAAATAAATGTCAAATCAGAAGCCAAATGTGTGAGTGTTTGTTTTGTTCGTTTTCATTTTTTCTGCATAAGGTGGCAGGATGATTGCAAATGGCTTGTAATTTAATTGTATATGATCTTTCGTATAGCCATTTGTCAGTGGTTCTTAAGATACTCCAAATTTTATGCACATACATACATACTGTACAGGCCAGAACAGACTCCAGTAACGTGTGTTTCCTTTCTTGGGAGTCCTCAATCTACCTCGCAAAGGCTAAATCCAGTGGCACCAGCTTTATTACTAAAACATTCACACGGGAACAGTTGAGAAAAACTAGGCCTCCATACCAAACACACCCTTAAACTTGAGCTGGTTGATAGAGACTAGAGAGTAGAGAGCACTACATGGATTATGGACCTAGCTAAAAGAAAGACCTTTTGTTTGAAGATAAGCTTAAGAAATTCGCACACTCTCTGTCACCTTGACACAAGTGTTCTCCTCGACCCCAACTAATAATGTATGATCACAAATTTCCTGTCTTTTTTTTAAACTTTGGTCTCTTCTTTTGTCTACATGATGATAAGGTTAGTGACCTCGCATCTTTCTACTTTATCCTGTTAGTGGACCTAGCTGGAAATGTGATGGAAATAGTTAGTAAAAGTAAGATCTTTGCATTTTCTGTTTTCTTAAAAACTGAAATATTACATAACTTGTGGCAATTAATTTGTCCATTTTCATACTGAGAGTGAGAGAGTCCTATTTTTTGGATTTATGGCTTATGCTGCTGTTACTTCCCTTATGAGAACCATACACCAATCAATGGAAATTATTCAATGTGATTTGCAACCGTTTTATGAAATATGTTTTTTGACTTACAAACATTGAATTAAAATCTTTTGAACTTTTATAAATTAAGATTTTTTTCTTTTAAAGAAATCGATACTGTAATCCTCTATATCAACGAGCTTACTCATGTGAAGGTCAAGTGATAAAACCAGGGGCCAGAGCCAGTCTAGACCTAGGGGGTTCGGAGCTTTTGTAATTGGATCATTGTGATTACGGGTTGAATGTCTAATTTTCTAGGCGATTATGATAGTATTTTGTATCTGAATAATAGCTCACTTTTTCTAAGTAATGGAAAAGAAGGTCAAAACGTGCCACTAAAAGACTTGACTAAGACAAAAATGGGTTGTCAAGAACGTAGAGGAGGGTAGGATGGACAGTTGGTCAGATCTAGTATGGATAATACATGGAGGTAGTTGGAGTGGCGGCTCTCCCAAAATTCTGTCATCTTAGATCTTTGGATTCGGTCTTATACATATGTGAGGAAGGTAATGAAAAATATTGGGGTAGAGGATTACAACATGGGGAATCAAGATTTCAGATGGTCAGTCAACTAACTGAGCTACTAAGATTTCAAAAAAAAGAAAAAGAAGGGTTCCATGTGACATGAAAGTCTCATATTGCATAGTTTTTGAATGAGGCAAAGAAGTGAGGAATCAGATGAACCACCTTCGATGGAAAATTATACTATTTATACATATAGTTAAAATTGTTTTCTATCTTTATATTATTGTAGTCAGCTAATTTGCATGTTTACTTATTCATATTTTGAATAGACATAATACATAAATACATCCTTTAACTTGGCTTCAAATCAAATTTATGCCCTTCAACTTTGGATGTGCACAAATAGACATTTAAATTTGTATAAAGTTGAAGAAATAGACACACATGTCCTACATGACATCCTACATGTCATTTTTTATCCTACGTGGTGTCTTACGTGTATTGTGTCATGTAGGACTCGTGTGGCTAGTCGCTCAACTTTATACAAATTTAAGTGCATACTTGTGCATACCCAAAGTCGGAGAACATAAACGTCAAATGAGGTCAAGTTAAAGGGCACATTTATGTATTATGCCTTCTTGATTTCATTAATAATGAAAATCCTGAATCATATTCAGATTCCGTCACTAATCGTTGAATTAATATACTATGTATTATTTATTGGATTTTTTTGCTATCTTATTTTTAATTGTTCAAAGTTTTGTTTATTATCTTTAGCATAATATCTGATTATATTATTTTGATATACTTTTTCTAGCCCTAATTACTTGTCCATTTTTGAATTGACACACCTATTAAGAAAACAATTATTGAAATAGTGAGTTTACTATTTTACCCATATTAATTATGAAGTGGGTGATTTAAAAACTCAAGATTTTCAAAAAGTTCTACTTTTTTCAAAGTAATAAATTAAGGGTATAATATGTAAAAAAAATTATTCTTTCTTGATTTGTCAAAATAGAAAAGTAATTAGGAATAACTAAAAAAATGGATAAATAATTAAAAACGGAGGGAGCAATATGTTATCTTTAGCCTAATAATATCTGATTAATGGCCACCCTAATTGATTGGATAGGAGAGGATAGACTTGCTTCCAAGTAAGCCAAAATATAAAAAGTTGACAAAAGGGTGCTAAATTCGAGACACATGTAGTACTTATATAATTCATGTGCGGACTCGTTCTTTTGTAGTACTCCTTCCGTTCTATTTTATATGTCATATTTTTATTTTATACTTTCATTAAGAAATGATGTAATTTTATCTTTCTATTCTTATTTAATGTTTTTTTAAGTCAATTTTATAATAAATGATGAATATATTTTCAAGATTAATTAACTACTCTATCAAGGGTATAATAGGTAAAATATGGTAATTTATACATAAATTTTATAAAATGACAAGTATTGTGGTCCAACTATTTATAGAAAGAGATGACATATAAAATGGGACGGAGGGAGTTATAAAGTTGACTTAAGAAAACATTTAATAAGGGTAGAAGGGTAAAGTTACATTATTTCTTAATGTAAATGTAAAGTAAAAATGTAACATATAAAATGGGAAGGAGGGAGTAGTATTTTCTCGTTTTATTTTACGTGGCACTCTATTCTCATAATCTATCTTTAAAAATGTCATTTTATTGTAATTGAAAATAATTTAACTTAAAATTCTCCATCTACCCTTAATTAATGAAATGATTTACAATTATATAAATATATAAAAATTGTTTTAGCTTATAATTTTCTAAAATCTTTTTTTCTCTTATACATCGTATTAAGTCAAACAATACTAAGTCAAATGGAATGGCGGAGTATTTCTTTTATTTTTTTTCACACCGTCCATATGTTTTCTCCCATCCCCCAGACCCCCACTATGTATATTCACTCCTTAGTTGAATCTGAATTTAGAGTTTAGAAGCTT

>Rpi-vnt1.3

AACGGTGTAATTTCTTTTGACTATTCTACTAGTATCTATCCACAGCACGTGTTGTTCCTTTCTTCTTTCGTTTTTCATTTACTTGACATTATTAGGAGACTTGGCCCTGAACTCCAACTATTCTAAGCTGACCTTTCTTTTCCTTTACCAATTATCTTCTTCTTTCTAATTTCGTTTTACGCGTAGTACTGCCTGAATTTTCTGACTTTCAACGTTTGTTATTCATGCTTGAAAACGAAATACCAGCTAACAAAAGATGAATTATTGTGTTTACAAGACTTGGGCCGTTGACTCTAACACTAAAGCAAATAGTACATCTTTCTTATCCTCTTTCTCTTACTTTCCCTTCCTCATCCTCACATTTAGAAAAAAGAAATTTAACGAAAAATTAAAGGAGATGGCTGAAATTCTTCTCACAGCAGTCATCAATAAATCAATAGAAATAGCTGGAAATGTACTCTTTCAAGAAGGTACGCGTTTATATTGGTTGAAAGAGGACATCGATTGGCTCCAGAGAGAAATGAGACACATTCGATCATATGTAGACAATGCAAAGGCAAAGGAAGTTGGAGGCGATTCAAGGGTGAAAAACTTATTAAAAGATATTCAACAACTGGCAGGTGATGTGGAGGATCTATTAGATGAGTTTCTTCCAAAAATTCAACAATCCAATAAGTTCATTTGTTGCCTTAAGACGGTTTCTTTTGCCGATGAGTTTGCTATGGAGATTGAGAAGATAAAAAGAAGAGTTGCTGATATTGACCGTGTAAGGACAACTTACAGCATCACAGATACAAGTAACAATAATGATGATTGCATTCCATTGGACCGGAGAAGATTGTTCCTTCATGCTGATGAAACAGAGGTCATCGGTCTGGAAGATGACTTCAATACACTACAAGCCAAATTACTTGATCATGATTTGCCTTATGGAGTTGTTTCAATAGTTGGCATGCCCGGTTTGGGAAAAACAACTCTTGCCAAGAAACTTTATAGGCATGTCTGTCATCAATTTGAGTGTTCGGGACTGGTCTATGTTTCACAACAGCCAAGGGCGGGAGAAATCTTACATGACATAGCCAAACAAGTTGGACTGACGGAAGAGGAAAGGAAAGAAAACTTGGAGAACAACCTACGATCACTCTTGAAAATAAAAAGGTATGTTATTCTCTTAGATGACATTTGGGATGTTGAAATTTGGGATGATCTAAAACTTGTCCTTCCTGAATGTGATTCAAAAATTGGCAGTAGGATAATTATAACCTCTCGAAATAGTAATGTAGGCAGATACATAGGAGGGGATTTCTCAATCCACGTGTTGCAACCCCTAGATTCAGAGAAAAGCTTTGAACTCTTTACCAAGAAAATCTTTAATTTTGTTAATGATAATTGGGCCAATGCTTCACCAGACTTGGTAAATATTGGTAGATGTATAGTTGAGAGATGTGGAGGTATACCGCTAGCAATTGTGGTGACTGCAGGCATGTTAAGGGCAAGAGGAAGAACAGAACATGCATGGAACAGAGTACTTGAGAGTATGGCTCATAAAATTCAAGATGGATGTGGTAAGGTATTGGCTCTGAGTTACAATGATTTGCCCATTGCATTAAGGCCATGTTTCTTGTACTTTGGTCTTTACCCCGAGGACCATGAAATTCGTGCTTTTGATTTGACAAATATGTGGATTGCTGAGAAGCTGATAGTTGTAAATACTGGCAATGGGCGAGAGGCTGAAAGTTTGGCGGATGATGTCCTAAATGATTTGGTTTCAAGAAACTTGATTCAAGTTGCCAAAAGGACATATGATGGAAGAATTTCAAGTTGTCGCATACATGACTTGTTACATAGTTTGTGTGTGGACTTGGCTAAGGAAAGTAACTTCTTTCACACGGAGCACTATGCATTTGGTGATCCTAGCAATGTTGCTAGGGTGCGAAGGATTACATTCTACTCTGATGATAATGCCATGAATGAGTTCTTCCATTTAAATCCTAAGCCTATGAAGCTTCGTTCACTTTTCTGTTTCACAAAAGACCGTTGCATATTTTCTCAAATGGCTCATCTTAACTTCAAATTATTGCAAGTGTTGGTTGTAGTCATGTCTCAAAAGGGTTATCAGCATGTTACTTTCCCCAAAAAAATTGGGAACATGAGTTGCCTACGCTATGTGCGATTGGAGGGGGCAATTAGAGTAAAATTGCCAAATAGTATTGTCAAGCTCAAATGTCTAGAGACCCTGGATATATTTCATAGCTCTAGTAAACTTCCTTTTGGTGTTTGGGAGTCTAAAATATTGAGACATCTTTGTTACACAGAAGAATGTTACTGTGTCTCTTTTGCAAGTCCATTTTGCCGAATCATGCCTCCTAATAATCTACAAACTTTGATGTGGGTGGATGATAAATTTTGTGAACCAAGATTGTTGCACCGATTGATAAATTTAAGAACATTGTGTATAATGGATGTATCCGGTTCTACCATTAAGATATTATCAGCATTGAGCCCTGTGCCTAAAGCGTTGGAGGTTCTGAAGCTCAGATTTTTCAAGAACACGAGTGAGCAAATAAACTTGTCGTCCCATCCAAATATTGTCGAGTTGGGTTTGGTTGGTTTCTCAGCAATGCTCTTGAACATTGAAGCATTCCCTCCAAATCTTGTCAAGCTTAATCTTGTCGGCTTGATGGTAGACGGTCATCTATTGGCAGTGCTTAAGAAATTGCCCAAATTAAGGATACTTATATTGCTTTGGTGCAGACATGATGCAGAAAAAATGGATCTCTCTGGTGATAGCTTTCCGCAACTTGAAGTTTTGTATATTGAGGATGCACAAGGGTTGTCTGAAGTAACGTGCATGGATGATATGAGTATGCCTAAATTGAAAAAGCTATTTCTTGTACAAGGCCCAAACATTTCCCCAATTAGTCTCAGGGTCTCGGAACGGCTTGCAAAGTTGAGAATATCACAGGTACTATAAATAATTATTTACGTTTAATATCCATGATTTTTTTAAATTTGTATTTAGTTCATCAACTAAATATTCCATGTCTAATAAATTGCAGGGATGCCTTTGAAAATGATTCTGTGTTGGAGAGAATCTTCTGATGCCTGTTGGTATTATAATACTAATAATAAGAGAAAAAGTTTGATTACTGTTTCAAGTTAATTGCTTGTGATTTGTAAAAACAAATTACTTTTATATTTCTCTTTGTTTTATTTTATGTTTA

>Rpi-vnt1.1

AGTTATACACCCTACATTCTACTCGAGTCATTATGATGATGTCTCACGACCAAATCAAATCAAAGTTAAATAAATATCGAACCGAACGCCCACTCTGTATGAGTATGGCAAAAGATTTTGAGAGAATCAAGTTGCATAAAAGCCTAATTTTCATGGAACATACAAATTGAGTCTCATAATAGCCCAAACTCACAGCCATGAACCCAAATTGGGTAAAGTTTTGCAAGACGTTCATCAAACAGTTAGGAAACATAAAATGGCGCTAGATATATAATAAATTTTTTTAACATATGGTGTGATTGATAGTTATATACTAAAGATGTTTGCTTAGTTACGTAATTTTTTCAAAAAAAAAAGGTACATTATCAATCATCAGTCACAAAATATTAAAAGTTACTGTTTGTTTTTTAAATTCCATGTCGAATTTAATTGAATGACACTTAAATTGGGACGAACGGTGTAATTTCTTTTGACTATTCTACTAGTATCTATCCACAGCACGTGTTGTTCCTTTCTTCTTTCGTTTTTCATTTACTTGACATTATTAGGAGACTTGGCCCTGAACTCCAACTATTCTAAGCTGACCTTTCTTTTCCTTTACCAATTATCTTCTTCTTTCTAATTTCGTTTTACGCGTAGTACTGCCTGAATTTTCTGACTTTCAACGTTTGTTATTCATGCTTGAAAACGAAATACCAGCTAACAAAAGATGAATTATTGTGTTTACAAGACTTGGGCCGTTGACTCTTACTTTCCCTTCCTCATCCTCACATTTAGAAAAAAGAAATTTAACGAAAAATTAAAGGAGATGGCTGAAATTCTTCTCACAGCAGTCATCAATAAATCAATAGAAATAGCTGGAAATGTACTCTTTCAAGAAGGTACGCGTTTATATTGGTTGAAAGAGGACATCGATTGGCTCCAGAGAGAAATGAGACACATTCGATCATATGTAGACAATGCAAAGGCAAAGGAAGTTGGAGGCGATTCAAGGGTGAAAAACTTATTAAAAGATATTCAACAACTGGCAGGTGATGTGGAGGATCTATTAGATGAGTTTCTTCCAAAAATTCAACAATCCAATAAGTTCATTTGTTGCCTTAAGACGGTTTCTTTTGCCGATGAGTTTGCTATGGAGATTGAGAAGATAAAAAGAAGAGTTGCTGATATTGACCGTGTAAGGACAACTTACAGCATCACAGATACAAGTAACAATAATGATGATTGCATTCCATTGGACCGGAGAAGATTGTTCCTTCATGCTGATGAAACAGAGGTCATCGGTCTGGAAGATGACTTCAATACACTACAAGCCAAATTACTTGATCATGATTTGCCTTATGGAGTTGTTTCAATAGTTGGCATGCCCGGTTTGGGAAAAACAACTCTTGCCAAGAAACTTTATAGGCATGTCTGTCATCAATTTGAGTGTTCGGGACTGGTCTATGTTTCACAACAGCCAAGGGCGGGAGAAATCTTACATGACATAGCCAAACAAGTTGGACTGACGGAAGAGGAAAGGAAAGAAAACTTGGAGAACAACCTACGATCACTCTTGAAAATAAAAAGGTATGTTATTCTCTTAGATGACATTTGGGATGTTGAAATTTGGGATGATCTAAAACTTGTCCTTCCTGAATGTGATTCAAAAATTGGCAGTAGGATAATTATAACCTCTCGAAATAGTAATGTAGGCAGATACATAGGAGGGGATTTCTCAATCCACGTGTTGCAACCCCTAGATTCAGAGAAAAGCTTTGAACTCTTTACCAAGAAAATCTTTAATTTTGTTAATGATAATTGGGCCAATGCTTCACCAGACTTGGTAAATATTGGTAGATGTATAGTTGAGAGATGTGGAGGTATACCGCTAGCAATTGTGGTGACTGCAGGCATGTTAAGGGCAAGAGGAAGAACAGAACATGCATGGAACAGAGTACTTGAGAGTATGGCTCATAAAATTCAAGATGGATGTGGTAAGGTATTGGCTCTGAGTTACAATGATTTGCCCATTGCATTAAGGCCATGTTTCTTGTACTTTGGTCTTTACCCCGAGGACCATGAAATTCGTGCTTTTGATTTGACAAATATGTGGATTGCTGAGAAGCTGATAGTTGTAAATACTGGCAATGGGCGAGAGGCTGAAAGTTTGGCGGATGATGTCCTAAATGATTTGGTTTCAAGAAACTTGATTCAAGTTGCCAAAAGGACATATGATGGAAGAATTTCAAGTTGTCGCATACATGACTTGTTACATAGTTTGTGTGTGGACTTGGCTAAGGAAAGTAACTTCTTTCACACGGAGCACAATGCATTTGGTGATCCTAGCAATGTTGCTAGGGTGCGAAGGATTACATTCTACTCTGATGATAATGCCATGAATGAGTTCTTCCATTTAAATCCTAAGCCTATGAAGCTTCGTTCACTTTTCTGTTTCACAAAAGACCGTTGCATATTTTCTCAAATGGCTCATCTTAACTTCAAATTATTGCAAGTGTTGGTTGTAGTCATGTCTCAAAAGGGTTATCAGCATGTTACTTTCCCCAAAAAAATTGGGAACATGAGTTGCCTACGTTATGTGCGATTGGAGGGGGCAATTAGAGTAAAATTGCCAAATAGTATTGTCAAGCTCAAATGTCTAGAGACCCTGGATATATTTCATAGCTCTAGTAAACTTCCTTTTGGTGTTTGGGAGTCTAAAATATTGAGACATCTTTGTTACACAGAAGAATGTTACTGTGTCTCTTTTGCAAGTCCATTTTGCCGAATCATGCCTCCTAATAATCTACAAACTTTGATGTGGGTGGATGATAAATTTTGTGAACCAAGATTGTTGCACCGATTGATAAATTTAAGAACATTGTGTATAATGGATGTATCCGGTTCTACCATTAAGATATTATCAGCATTGAGCCCTGTGCCTAGAGCGTTGGAGGTTCTGAAGCTCAGATTTTTCAAGAACACGAGTGAGCAAATAAACTTGTCGTCCCATCCAAATATTGTCGAGTTGGGTTTGGTTGGTTTCTCAGCAATGCTCTTGAACATTGAAGCATTCCCTCCAAATCTTGTCAAGCTTAATCTTGTCGGCTTGATGGTAGACGGTCATCTATTGGCAGTGCTTAAGAAATTGCCCAAATTAAGGATACTTATATTGCTTTGGTGCAGACATGATGCAGAAAAAATGGATCTCTCTGGTGATAGCTTTCCGCAACTTGAAGTTTTGTATATTGAGGATGCACAAGGGTTGTCTGAAGTAACGTGCATGGATGATATGAGTATGCCTAAATTGAAAAAGCTATTTCTTGTACAAGGCCCAAACATTTCCCCAATTAGTCTCAGGGTCTCGGAACGGCTTGCAAAGTTGAGAATATCACAGGTACTATAAATAATTATTTACGTTTAATATCCATGATTTTTTTAAATTTGTATTTAGTTCATCAACTAAATATTCCATGTCTAATAAATTGCAGGGATGCCTTTGAAAATGATTCTGTGTTGGAGAGAATCTTCTGATGCCTGTTGGTATTATAATACTAATAATAAGAGAAAAAGTTTGATTACTGTTTCAAGTTAATTGCTTGTGATTTGTAAAAACAAATTACTTTTATATTTCTCTTTGTTTTATTTTATGTTTATTTATCTTTAATTAATGGAGTAATAAAATAAAAATCTTATTTTCAATAGAAAAAAGTAGACCTTATTTGTGGTGCATGTATGGTATCTTTTTGAAATTTTTGATATATTTGCTCTTTGATTCGAATTTCTTGCTTATATGATGATTTGCATAAATATAAAATATTATACAAATACCTATGGGTTGGAAAATATAGAAATATGCCAATCAAATGTATACAAAAATCATTAATAGATAGAATCGTAAAAGATATACAAATGAGAAATGCTTGACTAAGAAGCTTCGTGCAACCTCTCACACTGAGCACAATGCATTTGGTGATCTCGGCACTATTGCTGTTACTTGTAAGACTACGTTCCCCAATAAGTCTTTCCAAACGGCTTGCAAAGCTGAGAATATGAAAATCTCATAGGTTAGTTTGCTGCGTTAATTATTTACATTTAATATGCTCGATAAGGTGATTTTAAAAAAATTTGTACTAGTTAATTCATGAACTAAATATTTCATTTAATACTCCATAATTCTGAATATGGAAAATAAATAATATTTAATAACAAGAATAAAATGATAAATTATTCATTGATTTTATAAATTGGATAAATATTATTAAATATTCTTAAATAATATAATGAACAAGTGAAGATGAACGGAGGGAGTATGAAGCCTCTTTTCAAAG

> Rpi-vnt1.1^A2056^

AGTTATACACCCTACATTCTACTCGAGTCATTATGATGATGTCTCACGACCAAATCAAATCAAAGTTAAATAAATATCGAACCGAACGCCCACTCTGTATGAGTATGGCAAAAGATTTTGAGAGAATCAAGTTGCATAAAAGCCTAATTTTCATGGAACATACAAATTGAGTCTCATAATAGCCCAAACTCACAGCCATGAACCCAAATTGGGTAAAGTTTTGCAAGACGTTCATCAAACAGTTAGGAAACATAAAATGGCGCTAGATATATAATAAATTTTTTTAACATATGGTGTGATTGATAGTTATATACTAAAGATGTTTGCTTAGTTACGTAATTTTTTCAAAAAAAAAAGGTACATTATCAATCATCAGTCACAAAATATTAAAAGTTACTGTTTGTTTTTTAAATTCCATGTCGAATTTAATTGAATGACACTTAAATTGGGACGAACGGTGTAATTTCTTTTGACTATTCTACTAGTATCTATCCACAGCACGTGTTGTTCCTTTCTTCTTTCGTTTTTCATTTACTTGACATTATTAGGAGACTTGGCCCTGAACTCCAACTATTCTAAGCTGACCTTTCTTTTCCTTTACCAATTATCTTCTTCTTTCTAATTTCGTTTTACGCGTAGTACTGCCTGAATTTTCTGACTTTCAACGTTTGTTATTCATGCTTGAAAACGAAATACCAGCTAACAAAAGATGAATTATTGTGTTTACAAGACTTGGGCCGTTGACTCTTACTTTCCCTTCCTCATCCTCACATTTAGAAAAAAGAAATTTAACGAAAAATTAAAGGAGATGGCTGAAATTCTTCTCACAGCAGTCATCAATAAATCAATAGAAATAGCTGGAAATGTACTCTTTCAAGAAGGTACGCGTTTATATTGGTTGAAAGAGGACATCGATTGGCTCCAGAGAGAAATGAGACACATTCGATCATATGTAGACAATGCAAAGGCAAAGGAAGTTGGAGGCGATTCAAGGGTGAAAAACTTATTAAAAGATATTCAACAACTGGCAGGTGATGTGGAGGATCTATTAGATGAGTTTCTTCCAAAAATTCAACAATCCAATAAGTTCATTTGTTGCCTTAAGACGGTTTCTTTTGCCGATGAGTTTGCTATGGAGATTGAGAAGATAAAAAGAAGAGTTGCTGATATTGACCGTGTAAGGACAACTTACAGCATCACAGATACAAGTAACAATAATGATGATTGCATTCCATTGGACCGGAGAAGATTGTTCCTTCATGCTGATGAAACAGAGGTCATCGGTCTGGAAGATGACTTCAATACACTACAAGCCAAATTACTTGATCATGATTTGCCTTATGGAGTTGTTTCAATAGTTGGCATGCCCGGTTTGGGAAAAACAACTCTTGCCAAGAAACTTTATAGGCATGTCTGTCATCAATTTGAGTGTTCGGGACTGGTCTATGTTTCACAACAGCCAAGGGCGGGAGAAATCTTACATGACATAGCCAAACAAGTTGGACTGACGGAAGAGGAAAGGAAAGAAAACTTGGAGAACAACCTACGATCACTCTTGAAAATAAAAAGGTATGTTATTCTCTTAGATGACATTTGGGATGTTGAAATTTGGGATGATCTAAAACTTGTCCTTCCTGAATGTGATTCAAAAATTGGCAGTAGGATAATTATAACCTCTCGAAATAGTAATGTAGGCAGATACATAGGAGGGGATTTCTCAATCCACGTGTTGCAACCCCTAGATTCAGAGAAAAGCTTTGAACTCTTTACCAAGAAAATCTTTAATTTTGTTAATGATAATTGGGCCAATGCTTCACCAGACTTGGTAAATATTGGTAGATGTATAGTTGAGAGATGTGGAGGTATACCGCTAGCAATTGTGGTGACTGCAGGCATGTTAAGGGCAAGAGGAAGAACAGAACATGCATGGAACAGAGTACTTGAGAGTATGGCTCATAAAATTCAAGATGGATGTGGTAAGGTATTGGCTCTGAGTTACAATGATTTGCCCATTGCATTAAGGCCATGTTTCTTGTACTTTGGTCTTTACCCCGAGGACCATGAAATTCGTGCTTTTGATTTGACAAATATGTGGATTGCTGAGAAGCTGATAGTTGTAAATACTGGCAATGGGCGAGAGGCTGAAAGTTTGGCGGATGATGTCCTAAATGATTTGGTTTCAAGAAACTTGATTCAAGTTGCCAAAAGGACATATGATGGAAGAATTTCAAGTTGTCGCATACATGACTTGTTACATAGTTTGTGTGTGGACTTGGCTAAGGAAAGTAACTTCTTTCACACGGAGCACAATGCATTTGGTGATCCTAGCAATGTTGCTAGGGTGCGAAGGATTACATTCTACTCTGATGATAATGCCATGAATGAGTTCTTCCATTTAAATCCTAAGCCTATGAAGCTTCGTTCACTTTTCTGTTTCACAAAAGACCGTTGCATATTTTCTCAAATGGCTCATCTTAACTTCAAATTATTGCAAGTGTTGGTTGTAGTCATGTCTCAAAAGGGTTATCAGCATGTTACTTTCCCCAAAAAAATTGGGAACATGAGTTGCCTACGTTATGTGCGATTGGAGGGGGCAATTAGAGTAAAATTGCCAAATAGTATTGTCAAGCTCAAATGTCTAGAGACCCTGGATATATTTCATAGCTCTAGTAAACTTCCTTTTGGTGTTTGGGAGTCTAAAATATTGAGACATCTTTGTTACACAGAAGAATGTTACTGTGTCTCTTTTGCAAGTCCATTTTGCCAAATCATGCCTCCTAATAATCTACAAACTTTGATGTGGGTGGATGATAAATTTTGTGAACCAAGATTGTTGCACCGATTGATAAATTTAAGAACATTGTGTATAATGGATGTATCCGGTTCTACCATTAAGATATTATCAGCATTGAGCCCTGTGCCTAGAGCGTTGGAGGTTCTGAAGCTCAGATTTTTCAAGAACACGAGTGAGCAAATAAACTTGTCGTCCCATCCAAATATTGTCGAGTTGGGTTTGGTTGGTTTCTCAGCAATGCTCTTGAACATTGAAGCATTCCCTCCAAATCTTGTCAAGCTTAATCTTGTCGGCTTGATGGTAGACGGTCATCTATTGGCAGTGCTTAAGAAATTGCCCAAATTAAGGATACTTATATTGCTTTGGTGCAGACATGATGCAGAAAAAATGGATCTCTCTGGTGATAGCTTTCCGCAACTTGAAGTTTTGTATATTGAGGATGCACAAGGGTTGTCTGAAGTAACGTGCATGGATGATATGAGTATGCCTAAATTGAAAAAGCTATTTCTTGTACAAGGCCCAAACATTTCCCCAATTAGTCTCAGGGTCTCGGAACGGCTTGCAAAGTTGAGAATATCACAGGTACTATAAATAATTATTTACGTTTAATATCCATGATTTTTTTAAATTTGTATTTAGTTCATCAACTAAATATTCCATGTCTAATAAATTGCAGGGATGCCTTTGAAAATGATTCTGTGTTGGAGAGAATCTTCTGATGCCTGTTGGTATTATAATACTAATAATAAGAGAAAAAGTTTGATTACTGTTTCAAGTTAATTGCTTGTGATTTGTAAAAACAAATTACTTTTATATTTCTCTTTGTTTTATTTTATGTTTATTTATCTTTAATTAATGGAGTAATAAAATAAAAATCTTATTTTCAATAGAAAAAAGTAGACCTTATTTGTGGTGCATGTATGGTATCTTTTTGAAATTTTTGATATATTTGCTCTTTGATTCGAATTTCTTGCTTATATGATGATTTGCATAAATATAAAATATTATACAAATACCTATGGGTTGGAAAATATAGAAATATGCCAATCAAATGTATACAAAAATCATTAATAGATAGAATCGTAAAAGATATACAAATGAGAAATGCTTGACTAAGAAGCTTCGTGCAACCTCTCACACTGAGCACAATGCATTTGGTGATCTCGGCACTATTGCTGTTACTTGTAAGACTACGTTCCCCAATAAGTCTTTCCAAACGGCTTGCAAAGCTGAGAATATGAAAATCTCATAGGTTAGTTTGCTGCGTTAATTATTTACATTTAATATGCTCGATAAGGTGATTTTAAAAAAATTTGTACTAGTTAATTCATGAACTAAATATTTCATTTAATACTCCATAATTCTGAATATGGAAAATAAATAATATTTAATAACAAGAATAAAATGATAAATTATTCATTGATTTTATAAATTGGATAAATATTATTAAATATTCTTAAATAATATAATGAACAAGTGAAGATGAACGGAGGGAGTATGAAGCCTCTTTTCAAAG

>Rpi-sto1

CTTGCTAATTGAGTGTCTGTTATAATCAGTATTAATTACTCTCAAGGTAATAGTATATTCCAAACAAATTTTGTGTTACCAAATTAAATATATTTCTAAAACTATCTTGAAAGTAGTTAATATACTTTTGAGTGTTGTATCATGTTTTTAATATAAAATATTAAAATTTAGATGAAATTTACTTTCTAGTTAAATTGGTCAAAGTTGAAAGAATTTCAAGTGAAAAAGTTTTTAATAATTTTGCTTTTATGCTATATTTTTTAAAGTTGAACGACTTTTTAATAAAAAAGAATAATAAAATTATATGATAATTTTTATAATACAATGGCCTTTATATGATGAAAAAAAAGAAAGAAATTAGATGACAACAATGTCCAAAAATAATCTTAAAGAATTATGATTTATATAATAAAATTAAATTTAAAATTTGATGAAAAAATAGAGAAAAGAGGAAGATGATGAAGTGAAATGATTGGTGGTGGGTCCATGTGACATTAAAAAAACAATTCTCTTAAATAATCCTTTCATACTAATGATAATTTTTTTTTTTTTTTTTTTTACTAATTGCGTATTGAGAAAAGGAAAATGGGGCGGTAATTACAAAGTAGGGAATCGAACTTTATCAAGAAGTTGAGAGTTCAAGTAACCAACCAACTAAACTACTAAAATTTTTCTAATTAATGATAATTGTAATTCATTTAGCATAAAAAATTTCATTGCACTTACTTTTAGAGTTTTGAAAACAATACTTCATCTATTCTATATTAATTAAATTTTCTATATTAATTAAATTTGTGAGGCAATACAAACTTATTAAGAAAAATATTTAAGGACATAATTTAAATCATATTTTTCACTATTGTTTTTTGTGAAATCATAAATATAACTTTGTAAATAGTGCAATTTATCTCCTAGAAGCAAACTTCACTAAAGAAAAGGGCAAAGATGGAAAAGAAACTAAATATTCATCTTAAACTTTGAACAATTCAATTATTTTGAACAATGAAAAAAATCTCAAAAATTCAATTAATATGAATATTTTAGAGGCAAAAAATTAGTACTCCCTCCGTTCACTTTTATTTGTCATATTGCGCTTTTCGAAAGTCAATTTGACTAATTTTTAAAGATCAATTAGATTACACTAATTCAATATTTTAAATAGAAAAATTAGATATTCAAAAACTATACAAAAAATATTATACATTGCAATTTTTTGCATATCAATATGATAAAAAAATATATCGTAAAATATTAGTCAAAATTTTTATAGTTTGACTCTAATAATGAAAAGTATAATAATTAATAGTGGACGGAGGAAGTATTGTCTTTCCAGATTTGTTGCCATTTTTGGGCCAAGGACCATTAGCAGTTCTCTTCATTTTCTACTTCTGTCTCATATTAGCTGGGCATCTTACTAAAAATATTTGTCTCATATTACTTGATTATTTACTAAATCAAAATAGAATTAATTAATTTTTTCTCATTTTACCCCTCCAATTAATATAGTTTTGAAAGTTTTAAACAAATTTTGAAGAATCAAAATTTCTTTTTGCAAGAGACTTATTAATATAAACAAAGGATAAAATAATAAAATTTGTCAATTTATTGACGATCACTTAATAATCGTGTAAAATAGAAAATGTTTATCTAATATGAGACGGAGAAAATATATCCTAAAATATTTTTGGATGGATATGTGATATTCTAACCATTCACTAGACTATATTATGCATTTTAGCCGCCAATGACTTATTTCAGCTTTAATTAATTAGGAAAGAGGAAACTGCCAATGAGGAAGAGTAGGGGCGTAGTTGCTGTCGACGAAAAAAAGATAATACTCACTCTTTTCGATTTTTATTTTTATTTATCACTTTTAACCTATCATGTAAAAAGATAATTATTTTTTTCATGCTTTATCCTTAGTATTAAATAATTTAATAGGGATTATTTTGTAAAATATTTATATGAATAATTGTTTTCGTAATGAATTTGTCCAGTCAAACAATGATAAATAAAAATGAACGGAGAGAGTAGAAAACAAAACAAAAGAACAAGTTGCCAACTTGAGAGATTAAAAGGGACCAAAACGCCTTGGATTTTGAGATTCCATATGTGAAATTTCCATGAAATAATTGAATTTGTATTATTACAAATCAAACTTTCTATTTCATTCCAACTAGCCATCTTGGTTTCAAAATTACACATTCATTCATTCACAGATCTAATATTCTTAATAGTGATTTCCACATATGGCTGAAGCTTTCATTCAAGTTCTGTTAGACAATCTCACTTCTTTCCTCAAAGGGGAACTTACATTGCTTTTCGGTTTTCAAGATGAGTTCCAAAGGCTTTCAAGCATGTTTTCTACAATCCAAGCCGTCCTTGAAGATGCTCAGGAGAAGCAACTCAACAACAAGCCTCTAGAAAATTGGTTGCAAAAACTCAATGCTGCTACATACGAAGTCGATGACATCTTGGATGAATATAAAACCAAGGCCACAAGATTCTCCCAGTCTGAATATGGCCGTTATCATCCAAAGGTTATCCCTTTCCGTCACAAGGTCGGGAAAAGGATGGACCAAGTGATGAAAAAACTAAAGGCAATTGCTGAGGAAAGAAAGAATTTTCATTTGCACGAAAAAATTGTAGAGAGACAAGCTGTTAGACGGGAAACAGGTACTCATCTTAAATTAGTATTACAACAACTAAGTTTATATTCATTTTTTTGGCAATTATCAAATTCAGAAAAGGGTTAAATATACTCATGTCCTATCGTAAATAGTGTAAATATACCTCTCGTTGTACTTTCGATCTGAATATACTTGTCAAATCTGGCAAGCTCAGAATCAAATTATCCACCCCAACTTTTAAATACTCGACATCTTTAGAAATCCACCTGTCTAACTCATCCACTACCCATTCCCTTTGCTTTGAATTCTTTTCTTTACCTATAAACTTGGAACACTCGATCCGTTTTGCTTTTCTTAACAAAGCAGCTCAGAGAAAAGAGGTTTTCTTCTATTCTGTTTCTCTGTGTGCTGCACTTGGGTCCTTAATCCCATTAAAAACAGGGCATGTTAATCCCAACGACGGTAGCCTTTCCTGACAGCTGACTGTAAATTTAGTCTAACAAAGAAAAAAAAAGATTAGACATGTTTTTCCTTGTCATTGATTAGGCTGGATTTCTTTCAGAGTGGAACATAGGGGATATATTGGACCAAAAATAGAATGGGTATATATTTAAAGTATTTCTGATAGAACAGGAGTATATTGTGCGAAAATATCCTCTATTTTCTGTTGTCTCCTAATGAGTTTGAATGTAATAATATTCTCATGTGGACATTGCTTGCACCAGGTTCTGTATTAACCGAACCGCAGGTTTATGGAAGAGACAAAGAGAAAGATGAGATAGTGAAAATCCTAATAAACAATGTTAGTGATGCCCAACACCTTTCAGTCCTCCCAATACTTGGTATGGGGGGATTAGGAAAAACGACTCTTGCCCAAATGGTCTTCAATGACCAGAGAGTTACTGAGCATTTCCATTCCAAAATATGGATTTGTGTCTCGGAAGATTTTGATGAGAAGAGGTTAATAAAGGCAATTGTAGAATCTATTGAAGGAAGGCCACTACTTGGTGAGATGGACTTGGCTCCACTTCAAAAGAAGCTTCAGGAGTTGCTGAATGGAAAAAGATACTTGCTTGTCTTAGATGATGTTTGGAATGAAGATCAACAGAAGTGGGCAAATTTAAGAGCAGTCTTGAAGGTTGGAGCAAGTGGTGCTTCTGTTCTAACCACTACTCGTCTTGAAAAGGTTGGATCAATTATGGGAACATTGCAACCATATGAACTGTCAAATCTGTCTCAAGAAGATTGTTGGTTGTTGTTCATGCAACGTGCATTTGGACACCAAGAAGAAATAAATCCAAACCTTGTGGCAATCGGAAAGGAGATTGTGAAAAAAAGTGGTGGTGTGCCTCTAGCAGCCAAAACTCTTGGAGGTATTTTGTGCTTCAAGAGAGAAGAAAGAGCATGGGAACATGTGAGAGACAGTCCGATTTGGAATTTGCCTCAAGATGAAAGTTCTATTCTGCCTGCCCTGAGGCTTAGTTACCATCAACTTCCACTTGATTTGAAACAATGCTTTGCGTATTGTGCGGTGTTCCCAAAGGATGCCAAAATGGAAAAAGAAAAGCTAATCTCTCTCTGGATGGCGCATGGTTTTCTTTTATCAAAAGGAAACATGGAGCTAGAGGATGTGGGTGATGAAGTATGGAAAGAATTATACTTGAGGTCTTTTTTCCAAGAGATTGAAGTTAAAGATGGTAAAACTTATTTCAAGATGCATGATCTCATCCATGATTTGGCAACATCTCTGTTTTCAGCAAACACATCAAGCAGCAATATCCGTGAAATAAATAAACACAGTTACACACATATGATGTCCATTGGTTTCGCCGAAGTGGTGTTTTTTTACACTCTTCCCCCCTTGGAAAAGTTTATCTCGTTAAGAGTGCTTAATCTAGGTGATTCGACATTTAATAAGTTACCATCTTCCATTGGAGATCTAGTACATTTAAGATACTTGAACCTGTATGGCAGTGGCATGCGTAGTCTTCCAAAGCAGTTATGCAAGCTTCAAAATCTGCAAACTCTTGATCTACAATATTGCACCAAGCTTTGTTGTTTGCCAAAAGAAACAAGTAAACTTGGTAGTCTCCGAAATCTTTTACTTGATGGTAGCCAGTCATTGACTTGTATGCCACCAAGGATAGGATCATTGACATGCCTTAAGACTCTAGGTCAATTTGTTGTTGGAAGGAAGAAAGGTTATCAACTTGGTGAACTAGGAAACCTAAATCTCTATGGCTCAATTAAAATCTCGCATCTTGAGAGAGTGAAGAATGATAAGGACGCAAAAGAAGCCAATTTATCTGCAAAAGGGAATCTGCATTCTTTAAGCATGAGTTGGAATAACTTTGGACCACATATATATGAATCAGAAGAAGTTAAAGTGCTTGAAGCCCTCAAACCACACTCCAATCTGACTTCTTTAAAAATCTATGGCTTCAGAGGAATCCATCTCCCAGAGTGGATGAATCACTCAGTATTGAAAAATATTGTCTCTATTCTAATTAGCAACTTCAGAAACTGCTCATGCTTACCACCCTTTGGTGATCTGCCTTGTCTAGAAAGTCTAGAGTTACACTGGGGGTCTGCGGATGTGGAGTATGTTGAAGAAGTGGATATTGATGTTCATTCTGGATTCCCCACAAGAATAAGGTTTCCATCCTTGAGGAAACTTGATATATGGGACTTTGGTAGTCTGAAAGGATTGCTGAAAAAGGAAGGAGAAGAGCAATTCCCTGTGCTTGAAGAGATGATAATTCACGAGTGCCCTTTTCTGACCCTTTCTTCTAATCTTAGGGCTCTTACTTCCCTCAGAATTTGCTATAATAAAGTAGCTACTTCATTCCCAGAAGAGATGTTCAAAAACCTTGCAAATCTCAAATACTTGACAATCTCTCGGTGCAATAATCTCAAAGAGCTGCCTACCAGCTTGGCTAGTCTGAATGCTTTGAAAAGTCTAAAAATTCAATTGTGTTGCGCACTAGAGAGTCTCCCTGAGGAAGGGCTGGAAGGTTTATCTTCACTCACAGAGTTATTTGTTGAACACTGTAACATGCTAAAATGTTTACCAGAGGGATTGCAGCACCTAACAACCCTCACAAGTTTAAAAATTCGGGGATGTCCACAACTGATCAAGCGGTGTGAGAAGGGAATAGGAGAAGACTGGCACAAAATTTCTCACATTCCTAATGTGAATATATATAATTAAGTTATTTGCTATTGTTTCTTTGTTTGTGAGTCTTTTTGGTTCCTGCCATTGTGATTGCATGTAATTTTTTTCTAGGGTTGTTTGTTTGTGAGTCTCTCTCTCATTGGATGTAATTTTCTTTTGGTAACAAATTAACAATCTATTTGTATTATACGCTTTCAGAATCTATTACTTATTTGTAATTGTTTCTTTGTTTGTAAATTGTGAGTATCTTATTGTATGGAATTTTCTGATTTTATTTTGAAAACAAATCAATAAGATCCATCTGTATTATACTCCCTTCGTCTCATTTTATGTGACACTTTTTGGATTTCGAGATTCAAACAAATCTATTTTTGATCTTAAATTTTTCATAGATCTTTTAAACATTTTGAATTATCAATTATTGTGATTTTAGTACTTTTTATGTAGTTTACAAATATATAAAATTTATTTTTTTTAAAAAAAGAAGATTTCATGCGCATATTCCCGATCAAACTTAAATTACTAGACTCTCGAAAAATGAAAAGTGTCACATAAATTGAGACTGAGGGAGTACTTGTTAATGTTGTAATTATTGGCGAACAATAATGTTGGTGATTATCACTTTCTGAATAAATGTTGTGTCACGTGGAAAAAACACCAAATAGAAGTATTCATGCTTTTTTAGTATATATAAACACGATTTTTAACTTGGTTTCAGCGGATAGTCATGACCTTTTACTCTGAATGTGCACAAGTAGATACTTGTATAAAATTAAATAAATTTTATAAAATTATACAATATGACACTGAGAGTAATTGATACCAATTGCAGTCGTTGCTGCTTTTCGATTCTCTGTCATTCTCTAGG

>Rpi-sto1^T3144^

CTTGCTAATTGAGTGTCTGTTATAATCAGTATTAATTACTCTCAAGGTAATAGTATATTCCAAACAAATTTTGTGTTACCAAATTAAATATATTTCTAAAACTATCTTGAAAGTAGTTAATATACTTTTGAGTGTTGTATCATGTTTTTAATATAAAATATTAAAATTTAGATGAAATTTACTTTCTAGTTAAATTGGTCAAAGTTGAAAGAATTTCAAGTGAAAAAGTTTTTAATAATTTTGCTTTTATGCTATATTTTTTAAAGTTGAACGACTTTTTAATAAAAAAGAATAATAAAATTATATGATAATTTTTATAATACAATGGCCTTTATATGATGAAAAAAAAGAAAGAAATTAGATGACAACAATGTCCAAAAATAATCTTAAAGAATTATGATTTATATAATAAAATTAAATTTAAAATTTGATGAAAAAATAGAGAAAAGAGGAAGATGATGAAGTGAAATGATTGGTGGTGGGTCCATGTGACATTAAAAAAACAATTCTCTTAAATAATCCTTTCATACTAATGATAATTTTTTTTTTTTTTTTTTTTACTAATTGCGTATTGAGAAAAGGAAAATGGGGCGGTAATTACAAAGTAGGGAATCGAACTTTATCAAGAAGTTGAGAGTTCAAGTAACCAACCAACTAAACTACTAAAATTTTTCTAATTAATGATAATTGTAATTCATTTAGCATAAAAAATTTCATTGCACTTACTTTTAGAGTTTTGAAAACAATACTTCATCTATTCTATATTAATTAAATTTTCTATATTAATTAAATTTGTGAGGCAATACAAACTTATTAAGAAAAATATTTAAGGACATAATTTAAATCATATTTTTCACTATTGTTTTTTGTGAAATCATAAATATAACTTTGTAAATAGTGCAATTTATCTCCTAGAAGCAAACTTCACTAAAGAAAAGGGCAAAGATGGAAAAGAAACTAAATATTCATCTTAAACTTTGAACAATTCAATTATTTTGAACAATGAAAAAAATCTCAAAAATTCAATTAATATGAATATTTTAGAGGCAAAAAATTAGTACTCCCTCCGTTCACTTTTATTTGTCATATTGCGCTTTTCGAAAGTCAATTTGACTAATTTTTAAAGATCAATTAGATTACACTAATTCAATATTTTAAATAGAAAAATTAGATATTCAAAAACTATACAAAAAATATTATACATTGCAATTTTTTGCATATCAATATGATAAAAAAATATATCGTAAAATATTAGTCAAAATTTTTATAGTTTGACTCTAATAATGAAAAGTATAATAATTAATAGTGGACGGAGGAAGTATTGTCTTTCCAGATTTGTTGCCATTTTTGGGCCAAGGACCATTAGCAGTTCTCTTCATTTTCTACTTCTGTCTCATATTAGCTGGGCATCTTACTAAAAATATTTGTCTCATATTACTTGATTATTTACTAAATCAAAATAGAATTAATTAATTTTTTCTCATTTTACCCCTCCAATTAATATAGTTTTGAAAGTTTTAAACAAATTTTGAAGAATCAAAATTTCTTTTTGCAAGAGACTTATTAATATAAACAAAGGATAAAATAATAAAATTTGTCAATTTATTGACGATCACTTAATAATCGTGTAAAATAGAAAATGTTTATCTAATATGAGACGGAGAAAATATATCCTAAAATATTTTTGGATGGATATGTGATATTCTAACCATTCACTAGACTATATTATGCATTTTAGCCGCCAATGACTTATTTCAGCTTTAATTAATTAGGAAAGAGGAAACTGCCAATGAGGAAGAGTAGGGGCGTAGTTGCTGTCGACGAAAAAAAGATAATACTCACTCTTTTCGATTTTTATTTTTATTTATCACTTTTAACCTATCATGTAAAAAGATAATTATTTTTTTCATGCTTTATCCTTAGTATTAAATAATTTAATAGGGATTATTTTGTAAAATATTTATATGAATAATTGTTTTCGTAATGAATTTGTCCAGTCAAACAATGATAAATAAAAATGAACGGAGAGAGTAGAAAACAAAACAAAAGAACAAGTTGCCAACTTGAGAGATTAAAAGGGACCAAAACGCCTTGGATTTTGAGATTCCATATGTGAAATTTCCATGAAATAATTGAATTTGTATTATTACAAATCAAACTTTCTATTTCATTCCAACTAGCCATCTTGGTTTCAAAATTACACATTCATTCATTCACAGATCTAATATTCTTAATAGTGATTTCCACATATGGCTGAAGCTTTCATTCAAGTTCTGTTAGACAATCTCACTTCTTTCCTCAAAGGGGAACTTACATTGCTTTTCGGTTTTCAAGATGAGTTCCAAAGGCTTTCAAGCATGTTTTCTACAATCCAAGCCGTCCTTGAAGATGCTCAGGAGAAGCAACTCAACAACAAGCCTCTAGAAAATTGGTTGCAAAAACTCAATGCTGCTACATACGAAGTCGATGACATCTTGGATGAATATAAAACCAAGGCCACAAGATTCTCCCAGTCTGAATATGGCCGTTATCATCCAAAGGTTATCCCTTTCCGTCACAAGGTCGGGAAAAGGATGGACCAAGTGATGAAAAAACTAAAGGCAATTGCTGAGGAAAGAAAGAATTTTCATTTGCACGAAAAAATTGTAGAGAGACAAGCTGTTAGACGGGAAACAGGTACTCATCTTAAATTAGTATTACAACAACTAAGTTTATATTCATTTTTTTGGCAATTATCAAATTCAGAAAAGGGTTAAATATACTCATGTCCTATCGTAAATAGTGTAAATATACCTCTCGTTGTACTTTCGATCTGAATATACTTGTCAAATCTGGCAAGCTCAGAATCAAATTATCCACCCCAACTTTTAAATACTCGACATCTTTAGAAATCCACCTGTCTAACTCATCCACTACCCATTCCCTTTGCTTTGAATTCTTTTCTTTACCTATAAACTTGGAACACTCGATCCGTTTTGCTTTTCTTAACAAAGCAGCTCAGAGAAAAGAGGTTTTCTTCTATTCTGTTTCTCTGTGTGCTGCACTTGGGTCCTTAATCCCATTAAAAACAGGGCATGTTAATCCCAACGACGGTAGCCTTTCCTGACAGCTGACTGTAAATTTAGTCTAACAAAGAAAAAAAAAGATTAGACATGTTTTTCCTTGTCATTGATTAGGCTGGATTTCTTTCAGAGTGGAACATAGGGGATATATTGGACCAAAAATAGAATGGGTATATATTTAAAGTATTTCTGATAGAACAGGAGTATATTGTGCGAAAATATCCTCTATTTTCTGTTGTCTCCTAATGAGTTTGAATGTAATAATATTCTCATGTGGACATTGCTTGCACCAGGTTCTGTATTAACCGAACCGCAGGTTTATGGAAGAGACAAAGAGAAAGATGAGATAGTGAAAATCCTAATAAACAATGTTAGTGATGCCCAACACCTTTCAGTCCTCCCAATACTTGGTATGGGGGGATTAGGAAAAACGACTCTTGCCCAAATGGTCTTCAATGACCAGAGAGTTACTGAGCATTTCCATTCCAAAATATGGATTTGTGTCTCGGAAGATTTTGATGAGAAGAGGTTAATAAAGGCAATTGTAGAATCTATTGAAGGAAGGCCACTACTTGGTGAGATGGACTTGGCTCCACTTCAAAAGAAGCTTCAGGAGTTGCTGAATGGAAAAAGATACTTGCTTGTCTTAGATGATGTTTGGAATGAAGATCAACAGAAGTGGGCAAATTTAAGAGCAGTCTTGAAGGTTGGAGCAAGTGGTGCTTCTGTTCTAACCACTACTCGTCTTGAAAAGGTTGGATCAATTATGGGAACATTGCAACCATATGAACTGTCAAATCTGTCTCAAGAAGATTGTTGGTTGTTGTTCATGCAACGTGCATTTGGACACCAAGAAGAAATAAATCCAAACCTTGTGGCAATCGGAAAGGAGATTGTGAAAAAAAGTGGTGGTGTGCCTCTAGCAGCCAAAACTCTTGGAGGTATTTTGTGCTTCAAGAGAGAAGAAAGAGCATGGGAACATGTGAGAGACAGTCCGATTTGGAATTTGCCTCAAGATGAAAGTTCTATTCTGCCTGCCCTGAGGCTTAGTTACCATCAACTTCCACTTGATTTGAAACAATGCTTTGCGTATTGTGCGGTGTTCCCAAAGGATGCCAAAATGGAAAAAGAAAAGCTAATCTCTCTCTGGATGGCGCATGGTTTTCTTTTATCAAAAGGAAACATGGAGCTAGAGGATGTGGGTGATGAAGTATGGAAAGAATTATACTTGAGGTCTTTTTTCCAAGAGATTGAAGTTAAAGATGGTAAAACTTATTTCAAGATGCATGATCTCATCCATGATTTGGCAACATCTCTGTTTTCAGCAAACACATCAAGCAGCAATATCCGTGAAATAAATAAACACAGTTACACACATATGATGTCCATTGGTTTCGCCGAAGTGGTGTTTTTTTACACTCTTCCCCCCTTGGAAAAGTTTATCTCGTTAAGAGTGCTTAATCTAGGTGATTCGACATTTAATAAGTTACCATCTTCCATTGGAGATCTAGTACATTTAAGATACTTGAACCTGTATGGCAGTGGCATGCGTAGTCTTCCAAAGCAGTTATGCAAGCTTCAAAATCTGCAAACTCTTGATCTACAATATTGCACCAAGCTTTGTTGTTTGCCAAAAGAAACAAGTAAACTTGGTAGTCTCCGAAATCTTTTACTTGATGGTAGCCAGTCATTGACTTGTATGCCACCAAGGATAGGATCATTGACATGCCTTAAGACTCTAGGTCAATTTGTTGTTGGAAGGAAGAAAGGTTATCAACTTGGTGAACTAGGAAACCTAAATCTCTATGGCTCAATTAAAATCTCGCATCTTGAGAGAGTGAAGAATGATAAGGACGCAAAAGAAGCCAATTTATCTGCAAAAGGGAATCTGCATTCTTTAAGCATGAGTTGGAATAACTTTGGACCACATATATATGAATCAGAAGAAGTTAAAGTGCTTGAAGCCCTCAAACCACACTCCAATCTGACTTCTTTAAAAATCTATGGCTTCAGAGGAATCCATCTCCCAGAGTGGATGAATCACTCAGTATTGAAAAATATTGTCTCTATTCTAATTAGCAACTTCAGAAACTGCTCATGCTTACCACCCTTTGGTGATCTGCCTTGTCTAGAAAGTCTAGAGTTACACTGGGGGTCTGCGGATGTGGAGTATGTTGAAGAAGTGGATATTGATGTTCATTCTGGATTCCCCACAAGAATAAGGTTTCCATCCTTGAGGAAACTTGATATATGGGACTTTGGTAGTCTGAAAGGATTGCTGAAAAAGGAAGGAGAAGAGCAATTCCCTGTGCTTGAAGAGATGATAATTCATGAGTGCCCTTTTCTGACCCTTTCTTCTAATCTTAGGGCTCTTACTTCCCTCAGAATTTGCTATAATAAAGTAGCTACTTCATTCCCAGAAGAGATGTTCAAAAACCTTGCAAATCTCAAATACTTGACAATCTCTCGGTGCAATAATCTCAAAGAGCTGCCTACCAGCTTGGCTAGTCTGAATGCTTTGAAAAGTCTAAAAATTCAATTGTGTTGCGCACTAGAGAGTCTCCCTGAGGAAGGGCTGGAAGGTTTATCTTCACTCACAGAGTTATTTGTTGAACACTGTAACATGCTAAAATGTTTACCAGAGGGATTGCAGCACCTAACAACCCTCACAAGTTTAAAAATTCGGGGATGTCCACAACTGATCAAGCGGTGTGAGAAGGGAATAGGAGAAGACTGGCACAAAATTTCTCACATTCCTAATGTGAATATATATAATTAAGTTATTTGCTATTGTTTCTTTGTTTGTGAGTCTTTTTGGTTCCTGCCATTGTGATTGCATGTAATTTTTTTCTAGGGTTGTTTGTTTGTGAGTCTCTCTCTCATTGGATGTAATTTTCTTTTGGTAACAAATTAACAATCTATTTGTATTATACGCTTTCAGAATCTATTACTTATTTGTAATTGTTTCTTTGTTTGTAAATTGTGAGTATCTTATTGTATGGAATTTTCTGATTTTATTTTGAAAACAAATCAATAAGATCCATCTGTATTATACTCCCTTCGTCTCATTTTATGTGACACTTTTTGGATTTCGAGATTCAAACAAATCTATTTTTGATCTTAAATTTTTCATAGATCTTTTAAACATTTTGAATTATCAATTATTGTGATTTTAGTACTTTTTATGTAGTTTACAAATATATAAAATTTATTTTTTTTAAAAAAAGAAGATTTCATGCGCATATTCCCGATCAAACTTAAATTACTAGACTCTCGAAAAATGAAAAGTGTCACATAAATTGAGACTGAGGGAGTACTTGTTAATGTTGTAATTATTGGCGAACAATAATGTTGGTGATTATCACTTTCTGAATAAATGTTGTGTCACGTGGAAAAAACACCAAATAGAAGTATTCATGCTTTTTTAGTATATATAAACACGATTTTTAACTTGGTTTCAGCGGATAGTCATGACCTTTTACTCTGAATGTGCACAAGTAGATACTTGTATAAAATTAAATAAATTTTATAAAATTATACAATATGACACTGAGAGTAATTGATACCAATTGCAGTCGTTGCTGCTTTTCGATTCTCTGTCATTCTCTAGG

>Rpi-R9a

AGGTGAGCATGAAAGTGAAGACGAGCAGACAATTCTAAGGCCGAAGGCAATTTCTGAAGCTAGGACTAGGCTTCAAGCTAAAAAGATACAGATTCGACCAACTGGTACCAGGAGGATTGGCTTCAAAGGAGATGACAATGGTGTGAGCATTCCAACTAATCTGCCATACTCACCAAGAAAATTGGCATGGAAAGGAAAAGAAACTATGACTTCAAATCAGTTGACAGCTGAAAAGGAGATCAGAATTGGCAATTTGAAGGCAAAGAAAGGAAAAAAATCTACGACTTCAGATCAGTTGACTGTTGAAAAGGAGAAAAAAATTGGCAAATTGAAGGCAAAAAGGGGTGGGAAGAAGTAGTTGCATTATGAAGTTGGTTTTTTGTTTTCCGGTGTTATGTAATGGCATGATGAAACTTAATATTTTGACTTTTTTGATATTACTCTGTCTTGAACAAGTGTCTGTAAGTTTTTGTGGGTTCAAAATATATGACAATGCCATTACTACTTATCTATGTAGTTGTTATTTTTTGTTTTCCAGTTACATTATGTATGTTGCTTTATCTGGGAAGATGTCTTGATGGTAGGCTTAACAGATCTTGTGAGTTGTGAATCCCCAGTGATATTTCTTTTGAACTTGAGTGAGTTGATAATTTCTAGTGTTGGGAGTGTTTCTTTAATTTTAGTTGTCTACTAAGTTGTACAGATGACAAAATGGCTGAGATCATATTATATTGTTAACCTGCATTTGTGTAAAATTGAAAAGCAATCCATAATTTAGATGCTGTAAAAATTTGCAGATTGCTGTAAAAATGCGCAGATTGCTGTAAAAATACTGTAAAAATTTGCAGATTGCTGTAAAAATTTACAGATTGCTGTAAAAGTTTACAGATTGTAGATTGCTGCAAAAATTTGCAGATTACAGATTGCTGTAAAAATTTACAGATTACAGATTGCTGTAAAAATTTACAGATTACAGATTGCTGTAAAAATTTACAGATTGCTGTAAAAATGCAGATTTTGTAAATGGTAAAAATGCAGATTGCTAACATTAACAAGATTGCTAACTTTTCCATTACAATCAAGAGATCAACAAGATTGCTAACAACTTTCAACAAGCAAAACACAATGGCATTACATCAGTTGTAACCGGTATATTGCTAACATTACATCAAAATACTAACGACTACACCACACTACTACAACAGAGCTACACCAAACTGCTAACGACTACACACTACTAGAACAGAGCTACACCAAACTACTAACGACTACACACTACTAGAACAGAGCTACACCAAACTACTAACGACTACACACTACTAGAACAGAGCTACACCAAACTACTAACGACTACACACTACTAGAACAGAGCTACACCAAACTACTCACGACTACACATTACAAACGATCCAAGAAAAGAAGTTCAAACATAACTACAATACACTACATCAACTTGGCAGCTACAAAACCAACAATAATATCCCAAAAGATCAGAAGCAAGCTTATCAATTGAAACCCTTTCAACTTCCACTTCAGTTTATTATCATCCAAGTTCTTCAAATTAGAACATTCAATATGATGTTCCAACTCCAAGTTTGAAACTTTATCCTCCAAATTTGAATTTTCAAAAAAAGTCATATCATTCAAGTTAGAATCAATGTTTGGTTTATGAATTTCAATGCCACCTATTTCCTTTAGGATCTTCTTCAATCGGTTTCTTCCCTTCCGAATTGAATCTAATTCAATCTTTTGATTGTGAGTCATCAATGACGCATGACTTGGAAGTTTACTAACGATCCAATCCAATAACCACAAGAGAATGAGCAAGGACGACAACATTTGAAGAATCGACGTTCACCATTCTCACGGGTCATAGCAGTAAAATATTTTGCTATCAATCCACACTTACATGTATGAGACTCAACTTGTTGAGGAGAAGAAACAGAAGAAGATGTCAACATTGAAACAACACAAAATTTAACAAATCTAATGAAGAAGATGATTTTCAAAAGAGCTTCAAGAACTAACAGATGAAATTTGGGGGAAATGTTTAGGGTTTTGTTTAAAGAAATGGGTAATGGGTGTTTTATTTGGATGGGTTGGGTATTTTGAAGATGGGTCGGGTGGATCCGGGTGGATAGGGTGGTTTGGGTCAAACAAATTAGGCTTTAATTAATTAAATTTCCAATCAATTACAACATGCCACGTAATAAATGAAATAATTAAATATATTTTCTAAGTAGTCTGTTAGTCAAAAGGGCAAAATAGGTCCCTAAAGGTTAACCCCGAGGGTATTTTGAGGCCAAAAGGTAAACGAGGGGTATTTTTGTACCAATTCTGATACTATAAGGGTATTTTAGACCCTTCTCCCTTTTTTTTTTTTACTTTAATGCGACACTTTTTCATATTTTATATTTTCCTCAAATTATATCTTTTTTTTTCTTTTCCTTGTTGTAAGTCCTGGTGAAATAACCTCGTGATACCACAAGGATTTGAAGATTACACTCACAACAAAATAAAATATTTTTTTGATTCAAGCTCTAAAAATGAAAAAATACTGGTAGAAAGTATTTCTTTCTTAAACTAGCATTACGTGATATGAATTTGAATTAGTCTAATCAATAAATTTAGAACAAAACATTCCTTACCAGGAAAGTGAAGAGATTTTGACCATTCCACTAGAGTCATTATGGTGATGTCTCACCACCAAATCAAAGTTTAATAAAAATCGAACCGAATAACCGAACGTCCACTCGGACCAATTTTTTTTTTTTTTTGAGAGGATCAAATCGCATAAAAGCCTAATTTTCATGTAACATACAAATTGAGTCTCATAATATCCCAAACTCACAGCCATGAACCCAAATTGGGTAAAGTTTTGCAAGACATTTTAGGAAATTTAAAAATGGCGTCTGGATATTTAATTTTTTAAAAATATCGTGTGATTGATTAATTATACTAAAGATATTTGCTTAGTTACGTGACTTTTCAAAAAAAGAAAAAGAAAAGTACATTATCAATCATCAGCCACAAAATATTAAGTCACAGTTTGTTTCTTAAATTCCATATCGAATTAAATTGAATGACACTTAAATTGGAACGAATGGTGCAATTTCCTTCGACTATTCAACTAGTATCTTATCCACAGCATGTGTTGATCCTTTTTTCTTTCGTTTTTCATTTACTTGACATTATTAGGAGACTTGGCAGTGGACTCCAACTATTCTAAGCTGACCTTTCTTTTCCTTTACCAATTATCTTCTTCTTTCTAATTACTCATTCTGATCAGTTTTTTGTAGCTACTGAAAAAGAAATGGCTGAAATTCTTCTTACAGCAGTCATCAATAAATCTGTAGAAATAGCTGCAAATGTACTCTTTCAACAAGGGAGCCGCTTGAATTTTTTGAAAGAGGACATCGATTGGCTCCAGAGAGTACTGAGACACATTCGATCATATGTAGACGATGCAAAGGCCAAGGAAGTTGGAGGCGATTCAAGGGTCAAAAACTTATTAAAAGATATTCAAGAATTGGCAGGTGATGTGGAGGATCTCTTAGATGAGTTTCTTCCAAAAATCCAACAATCCAATAAGTTCAAAGGCGCAATTTGTTGCCTTAAGACGGTTTCTTTTGCCGATGAGTTTGCTGTGGAGATTGAGAAGATAAGAAGAAGGGTTGCTGACATTGATAGTTTAAGGACAACTTTCAACATCACAGATACAAGTAACAACAATAATGATTGCATTCCAATGGAACAGAGAAGAAAATTCCTTCATGCTGATGAAACAGAGGTCATCGGTTTGGATGATGACTTCAACAAGCTCCAAGACAAATTGCTTGTTCAAGATTTGTGTAATGGAGTTGTTTCAATAGTTGGCATGCCTGGTCTAGGAAAAACAACTCTTGCCAAGAAACTTTATAGGCATGTCCGTCATCAATTTGAGTGTTCTGCACTGGTCTACGTTTCACAACAGCCAAGAGCAGGAGAAATCTTACTTGACATAGCCAAGCAAGTTGGACTGACGGACGAGGGAAGGAAAGAACACTTGGAGGACAATCTAAGATCACTTTTGGAAACAAAAAGGTATGTTATTCTCTTAGATGACATTTGGGATACTAAAATCTGGGATGCTCTGAACCGTGTCCTTCGTCCTGAATGTGATTCAAAAATTGGCAGTAGGATAATTATCACTTCTCGATATCATCATGTAGGCAGATACATAGGAGAGGATTTCTCGCTCCACGAGTTGCAACCCTTAGATTCAGAGAAAAGTTTTGAACTCTTTACCAAGAAAATCTTTATTTTTGATAATAATAATAATTGGGCTAATGCTTCACCTGTCTTGGTAGATATTGGTAAAAGTATAGTTCGGAGATGTGGAGGTATTCCATTAGCCATTGTGGTGACGGCAGGCATGTTAAGGGCAAGAGAAAGAACGGAACATGCATGGAATAGAGTGCTTGAGCGTATAGGTCATAATATTCAGGATGGATGTGCTAAGGCATTGGCTCTGAGTTACAATGATTTGCCCATTGCATTAAGGCCATGTTTCTTGTACTTTGGTCTTTACCCCGAGGACCATGAAATTCGTGCTTTTGATTTGACAAATATGTGGATTGCTGAGAAGCTGATAGTTGTAAATAGTGGCAATGGGCGAGAGGCTGAAAGTTTGGCGGATGATGTCCTAAATGATTTGGTTTCAAGAAACTTGATTCAAGTTGCCAAGAGGACATATGATGGAAGAATTTCAAGTTGTCGCATACATGACTTGTTACATAGTTTGTGTGTTGACTTGGCTAAGGAAAGCAACTTCTTTCACACCGAGCACAATGCATTTGGTGATCCCGGCAATGTTTCTAGGCTGCGAAGGATTACATTCTACTCTGATAATAATGCCATGAATGAGTTCTTCCGTTCAAATCCTAAGCTTGAGAAGCTTCGTGCACTTTTCTGTTTCACAAAAGGAGACTCTTGCATATTTTCTCATTTGGCTCATCATGACTTCAAATTATTACAAGTGTTGGTTGTAGTCCAGCCTCGAAAAAATTATGATTTCAGCATTAGCCAAATCAAAATTGGGAACATGAGTTGCTTACGCTATCTGCGATTCGAGGGGGATATTTATGGGAAACTGCCAAATTGTATGGTGAAGCTCAAACACTTAGAGACCCTAGATATTAGTAAAAGCTTCATTATTAAACTTCCTACTGGTGTTTGGAAGACTACACAATTGAGACATCTTCGTTCTAATGGTTATAATCTAGCACCTTACAGTTACTTTTGTATAAGCCCATTTTTTCCAAACGTGCCTCCTAATAATGTACAAACTTTGATGTGGATGGATGGTGAATTTTTTGAACCGAGATGGTTGCACCGATTTATCAATTTAAGAAAACTGGGTTTACAGGAAGTATCCGATTCTACCATTAAGAAATTATCAACATTGAGCCCTGTGCCAACGACACTGGAGGTTCTAAAGCTCAGCTCATTTTTCAGTGAATTGAGAGAGCAAATAAACTTGTCGTCGTATCCAAATATTGTTAAGTTGCATTTGAACGGAAGAATTCCCTTGAACGTCTCTGAATCATTCCCTCCAAATCTTGTCAAGCTTACTCTTTGCAACTTGATGGTAGACGGTCATGTAGTGGCAGTGCTTAAGAAATTACCCAAATTAAAGATACTTACATTGCATAGGTGCAGACATGATGCAGAAAAAATGGATCTCTCTGGTGATGGTGATAGCTTTCCGCAACTTGAAGTTTTGCATATTAAAGATCCAGTCTGCTTGTCTGAAGTAACGTGCACGGATGATGTCGGTATGCCTAAATTGAAAAAGTTATTACTTATAGAAAGAACTGATTCCAACGTTAGGCTCTCGGAAAGACTTGCAAAGCTGAGAGTATGAAAATCCCAATGTGTCAACAGGTTAGTTATTTACTTTTAATATCTCAAAATAAGCTCATTTTTTATTAATTAATTCATGAACTAAATATTTTATGTCTAATAAATTGCAGATGCTTTTCAGAATGATTATGTCTTTGCTGGAGAGCATCTTTTGATGCCTGTTTGTATTTGTAATAAATAAATTAAATGTTTGATTGCTTCTTCAAGTTGATGTATTTGTGGCTTCTAATTTGTAAAATAAATATATTTATTCATCTATTTATGGTCTTATGTATATTTACCTTTGGAATTAGCAGTAGCTTAATTAGTTTCTTTTCTTCTTCAAGAATCAATGCTCACAATTCTAGTTTTAAACACGTTACTAAACTTACATTCTAAGTATTCTAATTAACTTAGTCCTTCAATTCTAAACTTGAAACTTTTAGACTCTAGAGCTTCTTTCCAAAATTTTTAATCTATGATCATTAAATTCATCGCACGTCTTGTCAATCAATATTATGTCATCTAACATCCGAAAATAACATACACTGGCACTTCACTTTGGATATATCGTGTCGATTGGTCCATCATTAAGACAAAAAGGAACATGCTAAGGGTTGATTCCTATGCAATTCCATCCTAATCGAGAAGTGTTCTGACTCACTCCCACAATTCTAATCCAAATTTTAATTCCATAGATAATGTGGTAAATCAAATTAAATTACAATTAATAGGCCTTTTTTTTCTTTTTAAGAAACGTCTCTCAATTTATACAAGAAATAGCTGTGTATAACATACTGACATTTTGGTTATACCAAAGTTCTCCTTACATTTTACTCCTTCCTTTCCGAAATATTCTGATGACTCGCTTTTTGAGAGTCAAATTATATGGACTTTAATCAATATCTTAACATGTATTGTTTTTAGGATCATATTATGTTATTAAATTTTAATTATATGAGGGGGGATTATCATATATAGTACTATTTTATAGTTTTTAAATATTTAATTTAACTAATCAAATTTAACTTCAAATATGAGTCAAATTATAAACACAACAACACAATTATCAGGGATGCAATCTACCACTGAGCTAATAGCCCGTTGTGCGAGCC

>Rpi-R8

AAAACTTTCACGCACCCATAGGAAGTGAAAAACACTTATTTTGTCATATCAGCATTTTGTATTTAATAGGTAATTTTTTAAACAATTTAAGTGTTCAATAGGTAATAGTCCTAGTTAAGATGTCTAACTGAAATATACAAATAACTTTTAAGGAATCGTGAATGACTTAAGCCTAATTTAATTGGTAAATATGTGTTATCCCGAAAGATGTATGGATGAATTATCACCACTACTTTTTTTAATCCCTGTTTGAGATCTCGATGACTATTCTTAGTCTAATTGTTCTGACATTATTTGGTCAATAAAGCATGTGAATACTAGTCTAATACATGAATTTCAATCTATTTTAAAGAAGGAGACAGCATTAATCTTACACCTACCACCTAAATCAACTTGCTGATACAACTTGCCTTTTTCTAAATATTAAATAATATCTGACTCATAAGTTAATAATTTATATATATAAAAAAAGGAGTCATCATTTTAAATTTATAGAAATACTCATATTCTATTTGAATGGATTGTCCGTGCCAAGTGAAAATATTAACTTAAAGAGTAATTATTTGTTACTATTATTATTGACTAGTGGATCTTTATAAATTTATGTGCATTTGAAAGTTTGTAATCGTGAATATTTATTTATAAAAGAAACATGAAAATATGTAATTCATCAATGCGATGCTTTAGAATATTTTGATATCTTATTTACAAATGATGATTTACTCATTCGGTAAGATTGCTTAAGAATTAGATAACTTTTGATAGTTTTCACAACTTGAGAATTTTCATATTTATGAGAAAAAATAACTTCATGTCCGACCCAATACCCCTTAATTATATGCTAAACAAATATCAAAATAGTTCATAGTTTAAGTATGAAATTAGCAAATCGGACATAATTTTGGGGGATTTTCCTAAGGAGTCGTTTGGCGTGAGGTATGAGGTATAAAAATTTTGGAATAAAATATAGGATTATTTTATTATGTATTTGATTGGAGGTACTAGATAGTCCTTTGATTATTTATCCCAATATTTATACTTTAGTGATGGAATAAGTTATTTCATATACATGTTAAATAACTTATTGTGAGATAATTACTCTCAAAATAACTTATTCTCGACCAAACGACCCTTAAAAAAAGGACTAAGTAATATAGTTGTATTATTGAGGCACTTTACAGCTACCACCTAAAGCAACTTGCTACTACAATTCATCTCTTGTTAGGTATAAGAATTATTAGAGAGACAGAAGAACTCTCTTCCTTCAAACCTTCAAACTTCCATTCATTTTATTTCAATGAATTTTTTTCTTATATCACCTATTGCGTAAACTTGGAAATATATCTTTGTTGGAATCCAAATCTGAGAAATTGAAGATTCTTGTCAACTAACTGTAAGTCATCTTCTTCATGAATTACTTTTTTCCCATAGATACAGAATTAGAGAGTTGATTTCTCAAATGGTTCACTCAACTATTAGTTAGTATCTCATAAAATTGAAAATGTAAGAAAGACGATTTTCTCAGATAATAATTAGTTAAGTGACATTTGACAATATGAAAATGGTACATAAGATGTATAACTTTTCACAAGCATTAACATAGCTTCAAACACTAGATATTGAAAGACATGAACCTAATTCCATGAGTTGGTGAACTTGCAGGAATTTTTCTACAAAAAATGAATGAAAATGAAATTGAGGAAATGTTAGATCACCTAAGAAGGATCAAAATTGAAGGTAACCTGGATTTCTTCAAGATTCGTCGTATTGGGGATCTTGATATTGTGCTAAGAGTTTTTAGAACCTTTATAAAGTATCATGTTCTTTTACCTGATTGTTTTGTCAAACTCACAATGAATGCCGAATGGACTGTGGAAATGCTTCACCGGGTATTTGATGGGATATCAGATGAATGTAAAACTAACCTTAATTTGGAAAGGCTAGAATCACATTTGTTGGAATTCTTTGAAGGTAACTCCAGTTTAAGTTACAATTATGAGTTGAATGATTTTGATCTGTCGAAATATATGGATTGTCTGGAAAAAATTCTAAATGATGTACTAATGATGTTCCTGGAAAAGGGTAGGTCCTGTTATCCCATAGAAAAACTTGCAATACAGCTATCTATAAAGAAACTGAAAATTGTTCAAAAGAAAATGATATTTTTGAGATACATATATACCACAGAGATAAATGGTAACGTCAACTATGAGAAGCTGGAATGTTTGGAGACTCGAATTCAGTTCATTGCTAACACTGTGGGACAATTTTGTTTGGCCGTATTAGATTATGTTGCTGATATTGAATTTAGTGATAATAATGATATCTTTAATATACCTCCGTATCTATTATCATTGATTGTGTTTGTGGAGCTGGAAATGAAGAAGATTTTTCATGGTGAACTAAAGGTGTCAAAGTTTACTCAATCAAAAACTTTCAAGGACAAGAAATTACCAAAAGAATTTTCAGATCTTCTCCAATATCTGTTGATGTATCTCAGAAACGAAAAACTCGAGAACTTTCCTAATAATATCTCTGCTCAAAATATTGATGTGGCAATAGAATTCTTGTTGGTTTTCCTTGATGCTGATGTGTCAAATCACGTTATTAATGGTAACTGGTTGAATGAGGTCTTGTTAAAGGTTGGAGCTATAGCGGGTGATATTCTATATGTAATTCAAAAGCTTCTTCCTAGATCTATAAACAAAGATGACACTAGCAAAATAAGTTTTTGCTCGATACAGATATTGGAGAAGACTAAAGATCTGAAGGCACAAGTTGAGACGTACTACAAATCCTTAAAATTTACTCCATCTCAGTTCCCCACCTTTGGTGGATTGAGCTTTCTGGATTCTCTTTTAAGGAAACTGAATGAGATGTCGAAATCTAAGTCTGGTTTAGATTTCCTGATGAAACCTCTTTTAGGGAATTTGGAGAAAGAGTTATCATCTCTTACATCCATTTTAGAGAAGGAGCTATCATCCATTTTCAGAGATGTCGTGCACCACGAACATAAAATTCCTAAAGATCTTCAGAGACGTACCATCAATTTGGCATATGAGGCTGAGGTTGCCATTGACTCTATTCTTGCTCAGTATAATGCTTTTTTGCATATTTTTTGCTCACTTCCTACAATTTTAAAAGAGATCAAGCAAATTAATGCAGAGGTGACTGAGATGTGGTCAGCAAACATTCCTCTTAATCCTCGCTACGTGGCTGCTCCATTTAAACATCTGCCAGCTCGACATAGCAATCTTGTGACTGATGAGGAGGTAGTGGGTTTTGAGAATAAAGCAGAAAAACTAATTGGTTATCTGATTAGAGGTACAAATGAGCTAGACGTCATCCCAATTGTAGGCATGGGGGGACAAGGGAAAACGACAATTGCTAGAAAGTTGTACAATAATGACATCATTGTTTCTCGCTTTAATGTTCGAGCATGGTGCATCATTTCTCAAACATATAGCCGAAGAGAGCTATTACAAGAGATTTTCAGTCAAGTTACGGGCTCCAAGGACAAGGAAGATGAGGTAGGCAAACTTGCTGACAGGTTGAGGAAAAGCCTAATGGGAAAGAGATATCTCATTGTATTGGATGATATGTGGGATTGTATGGTATGGGATGACTTAAGGCTTTCTTTTCCAGATGATGGAATCAGAAGTAGAATAGTCGTAACAACTCGACTTGAAGAAGTGGGTAAGCAAGTCAAGAACCATACTGATCCTTATTCTCTTCCATTCCTCACAACAAAAGAGAGTTGCCAATTGCTGCAGAAAAAAGTGTTTCAAAAGGAAGATTGCCCGCCTGAACTACAATATGTGAGTCAAGCAGTTGCAGAAAAATGCAAAGGACTGCCCCTAGTGGTTGTCTTGGTAGCTGGAATAATCAAAAAAAGGAAAATGGAAGAATCTTGGTGGAATGAGGTGAAAGATGCTTTATTTGACTATCTTGACAGTGAGTTCGAAGAATACAGTCTAGCGACTATGCAGTTGAGTTTTGATAACCTAGCTGATTGTTTAAAGCCTTGTCTTCTTTATATGGGGATGTTTCTGGAGGACGCAAGAATTCCAGTGTCTAAATTGATAAGCTTATGGATTGCTGAAGGATTCGTGGAGAACACTGAATCTGGGAGATTAATGGAAGAGGAAGCTGAAGGTTACTTGATGGATCTCATTAGCAGTAACGTGGTAATTGTTTCAAAGAAAGGTTATAATGGTAAAGTCAAATGCTGCCAGGTTCATGATGTTGTGCATCACTTTTGCTTGAAGAAGAGTAGAGAAGAAAAGTTTATGCTTGCAGTGAAGGGTCAATATATCCAGTTTCAACCGTTGGATTGGAAGGGAAGTCGAGTGAGCTTCAGTTTCAGTGAAGAGCTTTCCAAGTTTGCATCTCTGGTTTCCAAAACACAGAAGCCTTTCCACCAACACTTGAGGTCTCTGATAACGGCTAATGGTGGAGAATCTATTGATGTGATTCCCGTCTGTCAGATTAATGAATTGCGACTTCTTAAGGTCTTGGATTTGAGTTCTTATTATGTGGAGTCTTTGTGGTTAGCTAGATTAAACCCACTTAATCAGCTGAAGTACCTCGCAGTTTGGGCAGGTACTTTCTATTTTGATCCACAATCACATCTGCCCCATATAGAAACTTTAATTGTGACGAGTTGTTTTTATGGTGTACGGTTACCAGTGTCTTTTTGGGAAATGGAAAAATTAAGGCATGTTCATTTTGCTGGCGCTGGTTTTGCTATGCAGGGACTCTTTGAAGGATCCTCTAAATTGGAAAATTTGAGGATATTAAAGAAAATTGAGGAATTTCCAATTGATAGGCTGGATGTGTTATCAAGGAGGTGTCCTAATCTTCAACAACTTCAAATCACATTTGAGGATGATGTAGAGCCTTTTTGTCCCAAATTGGAGAGTCTTACCCAGCTTCAAGAACTTCAACTTTCCTTTGTGCATCCCCGCATTCTATCCGGGTTACAGTTGCCTTCAAATTTAAACAAATTGGTACTTAAAGGAATTCATATGGAAAGTGCTATTTCCTTCATTGCGGAACTACCAAGCCTGGAGTATCTCCAATTACTAGATGTGTGTTTTCCTCAATCAGAAGAGTGGTGCCTTGGAGATATCACGTTCCATAAACTTAAGTTGTTGAAACTGGTGCAGTTAAATATCTCAAAGTGGGATGCCTCGGAGGAATCATTTCCCTTGCTTGAAACACTTGTTATAAAAAAGTGTGATGACCTTGAGGAGATCCCACTTAGCTTTGCTGATATTCCATCATTGAAACAGATTAAGTTGATTGGGTCTTGGAAAGTATCTATGGAGGCTTCAGCTGTGAGAATTAAGGAAGAAGTCGAAGAGATTGAAGGATGTGACCGTATAGACCTCGTAAGAAGAAGTCGAAGAGATTGAAAGATGTGACCGTATAGACCTTGTCAAAGAATACTGAAGTCTATTTTGTTGCTAGCTCATTCTGTTATTGTAACACTAGTAGTGTTTGCTATGTTTGTTTTGATTGATCAACTCTTTTTTATGTATGATATGATTGGAGACAAATATACATCAGATCGTCTTTCACTAATTTCGTGTTTTTATGCAGCTTTATAATTTTGACCAATTTCTCTCCACTTACATCTTCATAGCTCAATACACATCAACCATGTTGTTGTTGTTGTTGTTGTTGAGTACTTCTTGATTTTCTAATTCGATGAACATCCGAAAAATTTTAAGGTTAAACTGCTTCTTGTTGCTGTAGGTGCAATGTTGAAGGATCTCTTTATTATGTGTTCAGGTGCCTCTTTTAGAAGTTGAAATTCATACAGAATGAAGCTAATAAATTTGTTCTTGTCATTCATCGATCATTTGAACTTTGCAACATATGAACTTAAGATATAGTAAAAATATAGTATGACATTCGAGTCCCCTCCGAATACAGAGTCGTCTATGTTAGGGAGCGTTTCACCCCCAATGTGGGACTTTATTACGTCGTGAATCCATTGTATCGGGCTCCAATGTGGGTACCGGACATTGGGTGGAAACAAAAAAATGTATATAGTACGACATGAAATGAATAAATAAGGGCATTTGACCCTTAAGAACCAAAAAAATCAATCTTTTGTATTATGATTGGCCCTGTTCTGAGAACAGACTCCATTCACAAGATCTATGGAAGAATTATCACCACTCCTTTTTTATCCTTGTTTGATTTATGACATATGATTTATCCTTTTTTGAATTATGTTTTTTTAAAAAAATATAAAAACTTATTCATAAGTTTATATTTTGTAAAAAAGAACAAATCATTATTTTCAGTTTTACAAAAATAAACTCATGTTCGACGTGAATAAATTGTCCGTCGTATCATGTGAAAGAATTACTAATTACTACTATTGTTGACTAGTGGATCTTTATAAGTTTATGTACATTTGAAAGTTAAAAGAAACATAAAGATATATAATTCATCGATGCGATGCTTTAGATATTATGATATCTTGTTAACGAAATGATTTACTCATTTGGTAAAATTGCATAAGAATTGAATTTCAAAGTTTTCACAACTTGTGCATTTTCATATTTATTAGAAAAAATATAACTTCATGTCCGACCCAATACCCAACCATAAAATTCCTTAATTATATGCTAAGCAAATATCATATAAGTGCATAATTTAACTATGAAACTGCCAAACCAAATAAAATTTTGGGGGATTTTCCTAAAGAGTCATTTGGTGTGAAGTATGAGATATAATAATTTTGGGATAAAATGCAGAATTATTTTATCTTGTGTTTGGTTGGAGCCATATTTGGTAGTCCTCCGATTATGTATCCCATATCATAGTGATGGAATATATAAATTATGATCACATATACATGATCAAGTAACTTATTTCGCGATAATTAATCCCGAAATAACTTGCTCCAACCAAACAACCCCTAAAGAAAAAGACAAATAATATGGGTGTATTATTGAGTCACTTTACAGCTACCGACTTAATTCATCTCTTGTT

>Rpi-R3b

TCTTTGTCATGTTGTTGGATGAAAAGTCATACTTTTGGAAAAGAAAAAAAAAGGACAATAGTTAATAGTAGCAGACATAGTTGTAAAACCACTAGCGAGAGAGAGAGAGAGAAATCAGTGATAATTCATAGTTGCAAATTACTCTTATCATTCAACAGGTAAAATTAAGCTCTTAGTAAAGAAACAGTAATCTTCTCCAAATTCTTATAATCATTTTGTTGTTTCCTGTTTTGCTTTGCAGATTTCAGAAATGGAAATTGGCTTAGCAGTTGGTGGTGCATTTCTCTCTTCAGCTTTGAATGTTCTCTTTGACAGGCTTGCTCCTAATAGTGATCTGTTGAAGATGTTTAAGAGGGACAAGCGTGATGTTCGGCTCTTAAAGAAGCTGAGGATGACTTTGCTTGGCCTTCAGGCTGTGTTAAGTGATGCGGAGAATAAGCAAGCATCAAATCCATACGTGAGCCAGTGGCTTAATGAGCTTCAAGATGCTGTGGACGGTGCTGAAAACTTAATTGAAGAAGTCAATTATGAAGTTTTGAGACTAAAGGTGGAAGGTCAGTGTCAAAATCTTGGAGAAACAAGCAATCAACAGGTAAGTGACTGCAACCTGTGCTTGAGTGATGATTTTTTTCTTAACATAAAGGAGAAGTTGGAAGAGACCATTGAAACATTGGAAGAGTTGGAAAAGCAAATTGGTCGCCTTGATCTAACAAAGTATCTTGATTCGGGTAAACAAGAAACAAGGGAATCTTCAACTTCTGTTGTTGATGAATCTGATATCTTAGGTAGGCAGAACGAAATAGAGGGATTGATTGACCGTTTGTTGTCTGAGGATGGAAAGAATCTGACTGTAGTTCCTGTTGTTGGAATGGGGGGCGTGGGCAAGACAACACTTGCTAAAGCTGTTTACAATGATGAGAAAGTAAAAAACCATTTTGGTTTCAAAGCTTGGATCTGTGTGTCTGAACCATATGATATTCTCAGAATAACAAAGGAGTTACTTCAAGAATTTGGCTTAATGGTTGATAACAATCTGAATCAACTTCAAGTCAAATTGAAGGAGAGCTTAAAGGGAAAAAAGTTTCTTATTGTCCTAGATGATGTATGGAATGAAAACTATAAAGAGTGGGATGACTTGAGAAATCTTTTTGTACAAGGAGATGTAGGAAGTAAGATCATTGTGACGACACGTAAGGAGAGTGTTGCCTTGATGATGGGTTGTGGGGCAATCAACGTGGGGACTCTATCTAGTGAAGTCTCTTGGGATCTTTTCAAGCGGCATTCATTTGAAAATAGGGATCCTAAGGAACATCCAGAACTTGAAGAGATTGGAATACAAATTGCATACAAGTGCAAAGGTTTGCCTTTAGCTCTAAAGGCACTTGCTGGTATTTTACGCTCCAAATCAGAGGTGGATGAGTGGAGACACATTTTAAGAAGTGAAATATGGGAGCTGCAAAGTCGTTCGAATGGAATCTTACCAGCGTTGATGTTGAGCTATAATGATCTTCCTCCACAATTGAAGCGGTGTTTTGCTTTTTGTGCAATATATCCGAAAGATTATCTATTTTGCAAAGAACAAGTTGTTCACCTGTGGATTGCTAATGGTCTTGTACAGCAGTTGCATTCAGCTAACCAATACTTTCTCGAGTTGAGATCGCGATCATTGTTTGAAAAGGTCCGAGAGTCTTCTAAATGGAATTCGGGGGAATTCTTAATGCATGACCTTGTCAACGATTTGGCCCAAATTGCATCTTCAAATCTGTGTATGAGGTTGGAAGAGAACCAAGGATCTCATATGTTGGAACGAACTCGACATTTGTCGTATTCAATGGGTGATGGTGATTTCGGTAAACTGAAAACCCTCAACAAATTGGAGCAATTGAGGACATTGCTTCCCATCAATATCCAGCGGCGTCCATGCCATCTTAAGAAGAGGATGCTTCATGACATATTTCCAAGACTAATATCCCTAAGGGCACTATCACTGTCTCCTTATGATATTGAGGAGTTGCCGAATGACTTGTTTATCAAATTGAAGCACCTAAAATTTTTGGACCTTTCTTGGACACAGATAAAAAAGTTGCCAGATTCAATTTGTGAACTGTACAGCTTAGAGATACTTATCTTGTCACATTGTAGTCATCTTAATGAGCCACCGCTGCAGATGGAGAAGTTGATCAACTTGCATCACCTCGACGTTAGCGACGCTTATTTCTTGAAGACGCCGCTACATGTGAGCAAGTTGAAAAATCTCCATGTGCTAGTGGGAGCTAAATTTTTTCTTACTGGTTCCAGTGGTTTGAGAATTGAAGATTTGGGTGAACTACATAACTTGTATGGATCTCTATCAATTCTAGAGTTGCAACATGTGGTAGATAGAAGGGAATCTCTGAAGGCAAATATGAGGGAAAAGAAACATGTTGAAAGGTTATCTTTGGAGTGGGGGGGAAGTTTTGCTGACAATTCACAAACTGAAAGAGACATACTTGATGAGCTACAACCAAATACAAACATAAAAGAACTCCGAATCACTGGCTATAGAGGAACAAAATTTCCAAATTGGCTAGCTGATCATTCATTTCATAAGCTAATAGAAATGTCTCTTAGCTACTGCAAGGACTGTGATTCCTTGCCAGCACTAGGACAGCTTCCTTGTTTAAAATCCCTTACCATTAGAGGGATGCATCAAATAACAGAGGTGAGTGAAGAGTTCTATGGTCGTTTTTCCTCCACAAAGCCATTTAACTCTCTTGAGAAACTTGAATTTGCAGAGATGCCGGAGTGGAAGCAGTGGCATGTACTGGGGAAGGGAGAGTTCCCTGTACTAGAGGAACTTTTGATTTATCGTTGCCCAAAGTTGATTGGGAAGTTGCCTGAAAATGTTTCTTCGCTGAGAAGATTGAGAATTTTAAAATGCCCTGAACTCAGTTTGGAGACACCTATCCAACTTTCAAATTTAAAAGAGTTTGAAGTTGCTGATGCTCAACTGTTTACATCTCAACTTGAAGGAATGAAGCAGATTGTTAAATTAGATATTACTGATTGTAAGTCTCTTACCTCCTTACCTATTAGCATTCTGCCGAGTACCTTGAAGAGAATAAGAATAGCTTTTTGTGGGGAGCTGAAATTGGAGGCGTCGATGAATGCTATGTTTCTCGAGAAATTGTCTCTAGTAAAATGTGATTCTCCTGAGTTGGTCCCAAGAGCACGCAATTTGAGTGTAAGAAGTTGCAACAACCTTACTAGGCTTTTGATTCCTACTGCCACTGAAAGACTCAGTATTAGAGATTATGATAATCTTGAAATACTTTCAGTGGCACGTGGGACTCAGATGACATCATTGAATATTTACGACTGCAAGAAGCTGAAGTCGCTGCCAGAACATATGCAGGAACTCCTTCCATCTCTTAAGAAACTGGTTGTGCAAGCTTGTCCAGAAATAGAGTCCTTTCCTGAAGGAGGATTGCCCTTCAATTTACAAGCCCTTTCAATCTGGAATTGCAAGAAACTGGTGAATGGCCGAAAAGAGTGGCATTTACAGAGACTCCCCTCTCTCATAGATTTAACCATCTACCATGATGGTAGCGATGAAGAGGTTCTTGCTGGTGAAAAATGGGAGTTGCCTTGCTCTATTCGAAGGCTTACTATATCCAATCTGAAAACATTAAGCAGCCAACTTCTCAAAAGCCTCACCTCCCTTGAGTACCTAGATGCTAGAGAGTTGCCTCAAATTCAGTCACTGCTGGAAGAAGGGCTTCCTTTCTCTCTTTCTGAGCTAATATTATTTAGTAATCATGATCTTCATTCACTACCGACAGAAGGTCTTCAGCATCTCACGTGGCTTCGACGTCTAGAGATTGTGGGTTGCCCTAGTCTCCAATCTCTTCCCGAATCGGGGTTGCCCTCCTCCCTCTCTGAGCTGGGCATTTGGAATTGCTCTAATCTTCAATCTCTTCCCGAATCAGGGATGCCCCCTTCCATCTCTAAACTACGCATTTCCGAATGCCCATTGCTCAAACCACTCCTAGAATTTAACAAGGGGGATTACTGGCCAAAAATTGCTCATATTCCCACCATATATATTGATAAGGAATACTAGTAATGATTAAAAAAAATGGTGCTCTGATAAATTGTAAGTTAAAATTCTTTATTTTTTTCATTTATTTTTTTGTTTACTTCCCTTTTCTTGCTTGTTAATTCTTTTCCTTTGTTAAATACTTGAGCAGCACAAAGCGTCTATTGCCACTGTGAAGCTGAGGAATCATCTTTGGATTGGGATGAATTTAATTTTAGATGACAGCAAAAAAGGTGATAAAACGAAAGCTTGTATAAGTAGAAATTGAAATTAT

> *Rpi-R3b^G1696/G3111^*

TCTTTGTCATGTTGTTGGATGAAAAGTCATACTTTTGGAAAAGAAAAAAAAAGGACAATAGTTAATAGTAGCAGACATAGTTGTAAAACCACTAGCGAGAGAGAGAGAGAGAAATCAGTGATAATTCATAGTTGCAAATTACTCTTATCATTCAACAGGTAAAATTAAGCTCTTAGTAAAGAAACAGTAATCTTCTCCAAATTCTTATAATCATTTTGTTGTTTCCTGTTTTGCTTTGCAGATTTCAGAAATGGAAATTGGCTTAGCAGTTGGTGGTGCATTTCTCTCTTCAGCTTTGAATGTTCTCTTTGACAGGCTTGCTCCTAATAGTGATCTGTTGAAGATGTTTAAGAGGGACAAGCGTGATGTTCGGCTCTTAAAGAAGCTGAGGATGACTTTGCTTGGCCTTCAGGCTGTGTTAAGTGATGCGGAGAATAAGCAAGCATCAAATCCATACGTGAGCCAGTGGCTTAATGAGCTTCAAGATGCTGTGGACGGTGCTGAAAACTTAATTGAAGAAGTCAATTATGAAGTTTTGAGACTAAAGGTGGAAGGTCAGTGTCAAAATCTTGGAGAAACAAGCAATCAACAGGTAAGTGACTGCAACCTGTGCTTGAGTGATGATTTTTTTCTTAACATAAAGGAGAAGTTGGAAGAGACCATTGAAACATTGGAAGAGTTGGAAAAGCAAATTGGTCGCCTTGATCTAACAAAGTATCTTGATTCGGGTAAACAAGAAACAAGGGAATCTTCAACTTCTGTTGTTGATGAATCTGATATCTTAGGTAGGCAGAACGAAATAGAGGGATTGATTGACCGTTTGTTGTCTGAGGATGGAAAGAATCTGACTGTAGTTCCTGTTGTTGGAATGGGGGGCGTGGGCAAGACAACACTTGCTAAAGCTGTTTACAATGATGAGAAAGTAAAAAACCATTTTGGTTTCAAAGCTTGGATCTGTGTGTCTGAACCATATGATATTCTCAGAATAACAAAGGAGTTACTTCAAGAATTTGGCTTAATGGTTGATAACAATCTGAATCAACTTCAAGTCAAATTGAAGGAGAGCTTAAAGGGAAAAAAGTTTCTTATTGTCCTAGATGATGTATGGAATGAAAACTATAAAGAGTGGGATGACTTGAGAAATCTTTTTGTACAAGGAGATGTAGGAAGTAAGATCATTGTGACGACACGTAAGGAGAGTGTTGCCTTGATGATGGGTTGTGGGGCAATCAACGTGGGGACTCTATCTAGTGAAGTCTCTTGGGATCTTTTCAAGCGGCATTCATTTGAAAATAGGGATCCTAAGGAACATCCAGAACTTGAAGAGATTGGAATACAAATTGCATACAAGTGCAAAGGTTTGCCTTTAGCTCTAAAGGCACTTGCTGGTATTTTACGCTCCAAATCAGAGGTGGATGAGTGGAGACACATTTTAAGAAGTGAAATATGGGAGCTGCAAAGTCGTTCGAATGGAATCTTACCAGCGTTGATGTTGAGCTATAATGATCTTCCTCCACAATTGAAGCGGTGTTTTGCTTTTTGTGCAATATATCCGAAAGATTATCTATTTTGCAAAGAACAAGTTGTTCACCTGTGGATTGCTAATGGTCTTGTACAGCAGTTGCATTCAGCTAACCAATACTTTCTCGAGTTGAGATCGCGATCATTGTTTGAAAAGGTCCGAGAGTCTTCTAAATGGAATTCGGGGGAATTCTTAATGCATGACCTTGTCAACGATTTGGCCCAAATTGCATCTTCAAATCTGTGTATGAGGTTGGAAGAGAACCAAGGATCTCATATGTTGGAACGAACTCGACATTTGTCGTATTCAATGGGTGATGGTGATTTCGGTAAACTGAAAACCCTCAACAAATTGGAGCAATTGAGGACATTGCTTCCCATCAATATCCAGCGGCGTCCATGCCATCTTAAGAAGAGGATGCTTCGTGACATATTTCCAAGACTAATATCCCTAAGGGCACTATCACTGTCTCCTTATGATATTGAGGAGTTGCCGAATGACTTGTTTATCAAATTGAAGCACCTAAAATTTTTGGACCTTTCTTGGACACAGATAAAAAAGTTGCCAGATTCAATTTGTGAACTGTACAGCTTAGAGATACTTATCTTGTCACATTGTAGTCATCTTAATGAGCCACCGCTGCAGATGGAGAAGTTGATCAACTTGCATCACCTCGACGTTAGCGACGCTTATTTCTTGAAGACGCCGCTACATGTGAGCAAGTTGAAAAATCTCCATGTGCTAGTGGGAGCTAAATTTTTTCTTACTGGTTCCAGTGGTTTGAGAATTGAAGATTTGGGTGAACTACATAACTTGTATGGATCTCTATCAATTCTAGAGTTGCAACATGTGGTAGATAGAAGGGAATCTCTGAAGGCAAATATGAGGGAAAAGAAACATGTTGAAAGGTTATCTTTGGAGTGGGGGGGAAGTTTTGCTGACAATTCACAAACTGAAAGAGACATACTTGATGAGCTACAACCAAATACAAACATAAAAGAACTCCGAATCACTGGCTATAGAGGAACAAAATTTCCAAATTGGCTAGCTGATCATTCATTTCATAAGCTAATAGAAATGTCTCTTAGCTACTGCAAGGACTGTGATTCCTTGCCAGCACTAGGACAGCTTCCTTGTTTAAAATCCCTTACCATTAGAGGGATGCATCAAATAACAGAGGTGAGTGAAGAGTTCTATGGTCGTTTTTCCTCCACAAAGCCATTTAACTCTCTTGAGAAACTTGAATTTGCAGAGATGCCGGAGTGGAAGCAGTGGCATGTACTGGGGAAGGGAGAGTTCCCTGTACTAGAGGAACTTTTGATTTATCGTTGCCCAAAGTTGATTGGGAAGTTGCCTGAAAATGTTTCTTCGCTGAGAAGATTGAGAATTTTAAAATGCCCTGAACTCAGTTTGGAGACACCTATCCAACTTTCAAATTTAAAAGAGTTTGAAGTTGCTGATGCTCAACTGTTTACATCTCAACTTGAAGGAATGAAGCAGATTGTTAAATTAGATATTACTGATTGTAAGTCTCTTACCTCCTTACCTATTAGCATTCTGCCGAGTACCTTGAAGAGAATAAGAATAGCTTTTTGTGGGGAGCTGAAATTGGAGGCGTCGATGAATGCTATGTTTCTCGAGAAATTGTCTCTAGTAAAATGTGATTCTCCTGAGTTGGTCCCAAGAGCACGCAATTTGAGTGTAAGAAGTTGCAACAACCTTACTAGGCTTTTGATTCCTACTGCCACTGAAAGACTCAGTATTAGAGATTATGATAATCTTGAAATACTTTCAGTGGCACGTGGGACTCAGATGACATCATTGAATATTTACGACTGCGAGAAGCTGAAGTCGCTGCCAGAACATATGCAGGAACTCCTTCCATCTCTTAAGAAACTGGTTGTGCAAGCTTGTCCAGAAATAGAGTCCTTTCCTGAAGGAGGATTGCCCTTCAATTTACAAGCCCTTTCAATCTGGAATTGCAAGAAACTGGTGAATGGCCGAAAAGAGTGGCATTTACAGAGACTCCCCTCTCTCATAGATTTAACCATCTACCATGATGGTAGCGATGAAGAGGTTCTTGCTGGTGAAAAATGGGAGTTGCCTTGCTCTATTCGAAGGCTTACTATATCCAATCTGAAAACATTAAGCAGCCAACTTCTCAAAAGCCTCACCTCCCTTGAGTACCTAGATGCTAGAGAGTTGCCTCAAATTCAGTCACTGCTGGAAGAAGGGCTTCCTTTCTCTCTTTCTGAGCTAATATTATTTAGTAATCATGATCTTCATTCACTACCGACAGAAGGTCTTCAGCATCTCACGTGGCTTCGACGTCTAGAGATTGTGGGTTGCCCTAGTCTCCAATCTCTTCCCGAATCGGGGTTGCCCTCCTCCCTCTCTGAGCTGGGCATTTGGAATTGCTCTAATCTTCAATCTCTTCCCGAATCAGGGATGCCCCCTTCCATCTCTAAACTACGCATTTCCGAATGCCCATTGCTCAAACCACTCCTAGAATTTAACAAGGGGGATTACTGGCCAAAAATTGCTCATATTCCCACCATATATATTGATAAGGAATACTAGTAATGATTAAAAAAAATGGTGCTCTGATAAATTGTAAGTTAAAATTCTTTATTTTTTTCATTTATTTTTTTGTTTACTTCCCTTTTCTTGCTTGTTAATTCTTTTCCTTTGTTAAATACTTGAGCAGCACAAAGCGTCTATTGCCACTGTGAAGCTGAGGAATCATCTTTGGATTGGGATGAATTTAATTTTAGATGACAGCAAAAAAGGTGATAAAACGAAAGCTTGTATAAGTAGAAATTGAAATTAT

>Rpi-R3b^G3111^

TCTTTGTCATGTTGTTGGATGAAAAGTCATACTTTTGGAAAAGAAAAAAAAAGGACAATAGTTAATAGTAGCAGACATAGTTGTAAAACCACTAGCGAGAGAGAGAGAGAGAAATCAGTGATAATTCATAGTTGCAAATTACTCTTATCATTCAACAGGTAAAATTAAGCTCTTAGTAAAGAAACAGTAATCTTCTCCAAATTCTTATAATCATTTTGTTGTTTCCTGTTTTGCTTTGCAGATTTCAGAAATGGAAATTGGCTTAGCAGTTGGTGGTGCATTTCTCTCTTCAGCTTTGAATGTTCTCTTTGACAGGCTTGCTCCTAATAGTGATCTGTTGAAGATGTTTAAGAGGGACAAGCGTGATGTTCGGCTCTTAAAGAAGCTGAGGATGACTTTGCTTGGCCTTCAGGCTGTGTTAAGTGATGCGGAGAATAAGCAAGCATCAAATCCATACGTGAGCCAGTGGCTTAATGAGCTTCAAGATGCTGTGGACGGTGCTGAAAACTTAATTGAAGAAGTCAATTATGAAGTTTTGAGACTAAAGGTGGAAGGTCAGTGTCAAAATCTTGGAGAAACAAGCAATCAACAGGTAAGTGACTGCAACCTGTGCTTGAGTGATGATTTTTTTCTTAACATAAAGGAGAAGTTGGAAGAGACCATTGAAACATTGGAAGAGTTGGAAAAGCAAATTGGTCGCCTTGATCTAACAAAGTATCTTGATTCGGGTAAACAAGAAACAAGGGAATCTTCAACTTCTGTTGTTGATGAATCTGATATCTTAGGTAGGCAGAACGAAATAGAGGGATTGATTGACCGTTTGTTGTCTGAGGATGGAAAGAATCTGACTGTAGTTCCTGTTGTTGGAATGGGGGGCGTGGGCAAGACAACACTTGCTAAAGCTGTTTACAATGATGAGAAAGTAAAAAACCATTTTGGTTTCAAAGCTTGGATCTGTGTGTCTGAACCATATGATATTCTCAGAATAACAAAGGAGTTACTTCAAGAATTTGGCTTAATGGTTGATAACAATCTGAATCAACTTCAAGTCAAATTGAAGGAGAGCTTAAAGGGAAAAAAGTTTCTTATTGTCCTAGATGATGTATGGAATGAAAACTATAAAGAGTGGGATGACTTGAGAAATCTTTTTGTACAAGGAGATGTAGGAAGTAAGATCATTGTGACGACACGTAAGGAGAGTGTTGCCTTGATGATGGGTTGTGGGGCAATCAACGTGGGGACTCTATCTAGTGAAGTCTCTTGGGATCTTTTCAAGCGGCATTCATTTGAAAATAGGGATCCTAAGGAACATCCAGAACTTGAAGAGATTGGAATACAAATTGCATACAAGTGCAAAGGTTTGCCTTTAGCTCTAAAGGCACTTGCTGGTATTTTACGCTCCAAATCAGAGGTGGATGAGTGGAGACACATTTTAAGAAGTGAAATATGGGAGCTGCAAAGTCGTTCGAATGGAATCTTACCAGCGTTGATGTTGAGCTATAATGATCTTCCTCCACAATTGAAGCGGTGTTTTGCTTTTTGTGCAATATATCCGAAAGATTATCTATTTTGCAAAGAACAAGTTGTTCACCTGTGGATTGCTAATGGTCTTGTACAGCAGTTGCATTCAGCTAACCAATACTTTCTCGAGTTGAGATCGCGATCATTGTTTGAAAAGGTCCGAGAGTCTTCTAAATGGAATTCGGGGGAATTCTTAATGCATGACCTTGTCAACGATTTGGCCCAAATTGCATCTTCAAATCTGTGTATGAGGTTGGAAGAGAACCAAGGATCTCATATGTTGGAACGAACTCGACATTTGTCGTATTCAATGGGTGATGGTGATTTCGGTAAACTGAAAACCCTCAACAAATTGGAGCAATTGAGGACATTGCTTCCCATCAATATCCAGCGGCGTCCATGCCATCTTAAGAAGAGGATGCTTCATGACATATTTCCAAGACTAATATCCCTAAGGGCACTATCACTGTCTCCTTATGATATTGAGGAGTTGCCGAATGACTTGTTTATCAAATTGAAGCACCTAAAATTTTTGGACCTTTCTTGGACACAGATAAAAAAGTTGCCAGATTCAATTTGTGAACTGTACAGCTTAGAGATACTTATCTTGTCACATTGTAGTCATCTTAATGAGCCACCGCTGCAGATGGAGAAGTTGATCAACTTGCATCACCTCGACGTTAGCGACGCTTATTTCTTGAAGACGCCGCTACATGTGAGCAAGTTGAAAAATCTCCATGTGCTAGTGGGAGCTAAATTTTTTCTTACTGGTTCCAGTGGTTTGAGAATTGAAGATTTGGGTGAACTACATAACTTGTATGGATCTCTATCAATTCTAGAGTTGCAACATGTGGTAGATAGAAGGGAATCTCTGAAGGCAAATATGAGGGAAAAGAAACATGTTGAAAGGTTATCTTTGGAGTGGGGGGGAAGTTTTGCTGACAATTCACAAACTGAAAGAGACATACTTGATGAGCTACAACCAAATACAAACATAAAAGAACTCCGAATCACTGGCTATAGAGGAACAAAATTTCCAAATTGGCTAGCTGATCATTCATTTCATAAGCTAATAGAAATGTCTCTTAGCTACTGCAAGGACTGTGATTCCTTGCCAGCACTAGGACAGCTTCCTTGTTTAAAATCCCTTACCATTAGAGGGATGCATCAAATAACAGAGGTGAGTGAAGAGTTCTATGGTCGTTTTTCCTCCACAAAGCCATTTAACTCTCTTGAGAAACTTGAATTTGCAGAGATGCCGGAGTGGAAGCAGTGGCATGTACTGGGGAAGGGAGAGTTCCCTGTACTAGAGGAACTTTTGATTTATCGTTGCCCAAAGTTGATTGGGAAGTTGCCTGAAAATGTTTCTTCGCTGAGAAGATTGAGAATTTTAAAATGCCCTGAACTCAGTTTGGAGACACCTATCCAACTTTCAAATTTAAAAGAGTTTGAAGTTGCTGATGCTCAACTGTTTACATCTCAACTTGAAGGAATGAAGCAGATTGTTAAATTAGATATTACTGATTGTAAGTCTCTTACCTCCTTACCTATTAGCATTCTGCCGAGTACCTTGAAGAGAATAAGAATAGCTTTTTGTGGGGAGCTGAAATTGGAGGCGTCGATGAATGCTATGTTTCTCGAGAAATTGTCTCTAGTAAAATGTGATTCTCCTGAGTTGGTCCCAAGAGCACGCAATTTGAGTGTAAGAAGTTGCAACAACCTTACTAGGCTTTTGATTCCTACTGCCACTGAAAGACTCAGTATTAGAGATTATGATAATCTTGAAATACTTTCAGTGGCACGTGGGACTCAGATGACATCATTGAATATTTACGACTGCGAGAAGCTGAAGTCGCTGCCAGAACATATGCAGGAACTCCTTCCATCTCTTAAGAAACTGGTTGTGCAAGCTTGTCCAGAAATAGAGTCCTTTCCTGAAGGAGGATTGCCCTTCAATTTACAAGCCCTTTCAATCTGGAATTGCAAGAAACTGGTGAATGGCCGAAAAGAGTGGCATTTACAGAGACTCCCCTCTCTCATAGATTTAACCATCTACCATGATGGTAGCGATGAAGAGGTTCTTGCTGGTGAAAAATGGGAGTTGCCTTGCTCTATTCGAAGGCTTACTATATCCAATCTGAAAACATTAAGCAGCCAACTTCTCAAAAGCCTCACCTCCCTTGAGTACCTAGATGCTAGAGAGTTGCCTCAAATTCAGTCACTGCTGGAAGAAGGGCTTCCTTTCTCTCTTTCTGAGCTAATATTATTTAGTAATCATGATCTTCATTCACTACCGACAGAAGGTCTTCAGCATCTCACGTGGCTTCGACGTCTAGAGATTGTGGGTTGCCCTAGTCTCCAATCTCTTCCCGAATCGGGGTTGCCCTCCTCCCTCTCTGAGCTGGGCATTTGGAATTGCTCTAATCTTCAATCTCTTCCCGAATCAGGGATGCCCCCTTCCATCTCTAAACTACGCATTTCCGAATGCCCATTGCTCAAACCACTCCTAGAATTTAACAAGGGGGATTACTGGCCAAAAATTGCTCATATTCCCACCATATATATTGATAAGGAATACTAGTAATGATTAAAAAAAATGGTGCTCTGATAAATTGTAAGTTAAAATTCTTTATTTTTTTCATTTATTTTTTTGTTTACTTCCCTTTTCTTGCTTGTTAATTCTTTTCCTTTGTTAAATACTTGAGCAGCACAAAGCGTCTATTGCCACTGTGAAGCTGAGGAATCATCTTTGGATTGGGATGAATTTAATTTTAGATGACAGCAAAAAAGGTGATAAAACGAAAGCTTGTATAAGTAGAAATTGAAATTAT

>Rpi-R3a

GATCCTTTTGGTGGTTGTTAATCTATGTGCTCATCGTTACCATTGTCATGACCCAATATTTTCTTTCTTCTATATAGCCCAATTGCCTGAAAGTATTATAGTATTTAGAAGTAGAATGTATATAAAGAAGGTATCACTGTCCATTCTGCATCGTGATTGTCTTTGATTTTTGATGATAATCCTTTCCAAGAGCAGGCTTCCTACCCAACTTCTGATTAAACTTACAGTGTCCAAGATTTCAATCCATTTATATTGCCTAACTATTATGCTGATAAAGCATTATCTTTCTTTTTTATCTGAACTTACCTGCAGTCTTACAGTATGCATAATTAAGACATAATGTTTCATCTGCTATATACTCACACTCACTGTAAAAAGTGTTTTCTGGAAGTATATTTTTTCTGTGATATTCTTATGCTGAATGTTGTCCATTTATGAGTATGGGAATTTTAGCTTAATGGGTGTCTTTGTTATAATTTTACCTTATTTGGTGCTTGGGATGATAGTGCCTTTGGGGAGAAGAAAAGCAGTGATTTAGGCAGAATCAAGTTTATGCATATGTTTGGTCTAATGTTGAAAAGGAAAAATGTCGAATGATGTGGGTATTTTCTTTTTTTAAGAATACAAAGTCACGATAAAGGGAGAAAATTTTGATGGTATTAATTTTGATATGGATAACCGTTGAGAATTTTGTCTGAGGTATTAGTTTCTATTTTGTACTTGGGATGATGATGTCTTTGGGAGTAGAAAAGTTGTGATCTATGCAGAATCATTTTCGCTGATTGTGATGGGGAAGCATATCTTATCCAACTTGATATAGTTAAATCTTGGTTTCTTGGTACCTTTCGTGAAACATTCATAGTTCATTTTGGTTCTGTAAATTTTGAGAAGTTGAACATCCCTCATAACAGGCTCTAATTCTTACTCGGATCCTTGGACGACGTTTAAACCTAGATAATATGAAGGTTAGGATACATGTCACCATGTCTCTTTGTTTTTTTACTAATTCAATTAACTTCAGAAACCTTGATGCATTTGCAATTCCCTTTCCAGATTGAAAGTTTGTATCCTGAAGAAATGGGACCAGATGTGATGCCTCTGGAAGACTTTCTAGTGAATGGCCTTCCCTTACTTGATCAGAAAATTGAAGATAGGATTAAACAAGCTTCTGCAAATGGGAATGTTCTGCGCTATGTTTGCTTGATTGATGATGCGAGGTAATTCTTTCATTTCATTTTTAGTTTGCTCAATGGATAATGGCAACTGGATCTTTTATTCTTATCACGTTCTGCAAGTCACCATACCTCTAGACGCTTAAAGCAAAGCTTGCTCACTGATGGGAGTGTTATGGCAAGTTAAGGTACTGATGAGTGATGAGCATAACAATTACTACTAATTAAGACAAGTTTATCTTAGAAGATAGTCTCAATTCCTCTTAACAAATGCTTTAGAAAATTATGACAGCAGTCACCCACTTGGACATATAAGTTTGAGAGCTATCTGCAAACCTGTTGTAAGTTCATCTGTTTTATGTTAGTCATCTGATCAGCTGTTCACCTCTTGTATCCGTAACCAATTAAAGAATGAGTCAGAAGTATAAATTTGTAATGGCATAGATGGGGAAAAATTGATTATTGAAAACACTTTTGTGTACATGACATTAACATCTCACATTTTCAGATGTGAAGTTGGCATCCAAGAAGTTCCAAAAGATTCTGCATTGGGAAGGTTAAGAGGAAGTGATAATGTGGTATGCATTGTTCACTTTCACCTCCCATTTTGCTATTTCTTTGGTCCCTCTTCTTCTTGTTCCATTACTTTGTTTTCCGTTGGGAATTTTCTTGTAACTGCTATGGAGGTTCAACCATATCATACGCGTCGCAATGTTTAAAGCCCTTTTGTGATGTCATTTAGTGTAGTGATGTATTACTTCATGCTGTGATTTAAAGAATTGCTCATCTTTTACCCATGCCTAGGCTGACGCCTTACTTTGTCTTTACTCTTTACGCATCATCTGGTGTCGCCCTGCAAAACTTTATGTAGTTAAGGTCTTTGATTCTACTTTAATCGTAAAATGTTTCCCTTGTACAGTTGTAATTATGAGGTCATAAAAACAGTAAGATTAATGATGATTGAGCGTCTGCAATTCGTTTGTCTGCAGGTGGAGATATACAGCCGTTGTTACGAGAAACAACCTTTGGTTATCCAAGGTGCTGGAGCAGGAAATGATACCACAGCAGCTGGTGTCCTAGCTGATATCCTTGACATTCAAGATTTATTCCCTTGACAGAGTATTATACACTGAGATACAAACATTCGATCCTCAAAGGACAAAAAATCGACTCTTCTTGTCCCTCTGCTTCCAGATTGGAGATTGAATTCAGGCTAAGAAAGCTCACGCATTTTTTGAAAAATTTCTCGATACACGAGATGCCTCGTCAGCTTACAGTAGATTATTACACAATTTCTCTCGTACATAACCGTCTCAAATGGAACAGTTCAAACTGAGTCATTTGCCTCAGTGCTGGACTAGTTTTTAGGATTTTTCTTCAGCTTATAATCAGTTTCCTTGTAATGACCATTTTCTTTATAATCTGAAAAACTGCTGATATTTCTTTATAATATTTCTTCAGAAAACCTTATGATTGAACACCACAATATTCACCTTTTCGGTATTTAATGTTCCTTTATGTTATGTGTTCTTCTATTTTTCCCTTTTTTTTTTAAAGTTAATCGTCAATTGGGACATGGACCCAGATAAAAACCATAATGACATTAATATCTCTCAATTGTTGAGATAAAATTATGAAAAGAGAAAAATCAATTATTAACCTATTCCTATGACTATTATTTGCATCTTATGAGAAGAGAAAAGACTTTGAAATATGACGTTGGCCTTTTCCTTTCTTCGACTGTCAACTATACAAAAACAAGGAATTCAGTTCTATTAAATTCAAGATTCAAAAATGATTTCTTTTTTATTTTGGAGCCCCGCCCCCCCCCCCCCCTCCCCCCTCTTGCTTCAATTATTGAATTGCAAAAGTAATGAATAGTCATGAAACCAGGCTGGCAGACTCCATAATGTTTTTGCTACTCTTCTTTTTTTTCTATGGCTGTGAGCCGTACACTGTAGATAAGACATAGTTGTACCACTTACAAGTAAGACCACTAGCAAAAGAAAGAGAGAAATCATTCTTGATCACATATCACTCTTAGCTCTTAGCATTCAACAGGTAAATAAAGTTGTTAGTAATTGAAGATTGAAACATTTATCTTCTTAAAATCATTTTGTGTTCTCCTTGTTTTGATTTGCAGATTTCAGTAATGGAGATTGGCTTAGCAGTTGGTGGTGCATTTCTCTCTTCAGCTTTGAATGTTCTCTTTGATAGGCTTGCTCCTCACGGTGATCTGCTCAACATGTTTCAGAAGCATAAGGATCATGTTAAGCTCTTAAAGAAGCTGGAGGACATTTTGCTCGGTCTTCAGATTGTGCTAAGTGATGCAGAGAATAAACAAGCATCAAATCGACATGTGAGCCAGTGGTTCAATAAGCTTCAGAATGCTGTGGACGGTGCTGAGAACTTGATAGAACAAGTCAATTATGAAGCTTTGAGGCTTAAGGTGGAAGGCCAGCATCAAAATCTTGCAGAAACAAGCAACCAGCAAGTAAGTGACCTTAACCTGTGCTTCAGTGATGATTTCTTTCTTAACATAAAGGATAAGTTGGAAGAAACCATTGAAACATTGGAGGTGTTGGAAAAGCAAATTGGTCGCCTTGGCTTAAAGGAACATTTTGGTTCGACTAAACAAGAAACTAGAACACCTTCAACTTCTTTAGTTGATGACTCTGATATCTTTGGAAGGCAGAATGATATAGAGGATTTGATTGACCGTTTATTATCTGAAGATGCAAGTGGAAAAAAGCGGACTGTAGTTCCTATTGTTGGAATGGGTGGTCTGGGTAAGACAACACTTGCTAAAGCGGTTTACAATGATGAGAGAGTGCAGATACATTTTGGTTTGAAAGCTTGGTTTTGTGTTTCTGAGGCATTTGATGCTTTCAGAATAACAAAAGGGTTACTTCAAGAAATTGGCTCATTTGACTTGAAGGCTGATGACAATCTTAATCAGCTACAAGTCAAATTGAAGGAAAGATTAAAGGGAAAGAAGTTTCTTATAGTTTTGGATGATGTGTGGAATGACAACTACAACAAGTGGGATGAATTGAGAAATGTTTTTGTACAAGGAGATATAGGAAGTAAGATCATTGTGACGACACGTAAAGAGAGTGTTGCCTTGATGATGGGAAATGAGCAAATTAGCATGGACAATTTGTCTACTGAATCCTCTTGGTCTTTATTTAAAACACATGCATTTGAAAACATGGGTCCTATGGGACATCCGGAACTTGAAGAGGTCGGGAAACAAATTGCAGCTAAGTGCAAAGGACTGCCCTTAGCTCTGAAGACGCTCGCTGGCATGTTACGCTCCAAATCAGAGGTTGAAGAGTGGAAACGTATTTTGAGAAGTGAAATATGGGAGCTGCCACACAATGACATATTACCAGCGTTGATGTTGAGCTACAATGATCTTCCCGCACATTTAAAGCGATGTTTTTCCTTTTGTGCAATATTTCCTAAAGATTATCCCTTTAGGAAAGAACAAGTTATTCATCTGTGGATTGCCAATGGTCTCGTACCACAGGAAGATGTAATAATTGAAGATTCAGGCAACCAATACTTTCTCGAGTTGAGGTCAAGATCATTATTCGAAAGGGTCCCAAATCCTTCTCAAGGGAACACAGAGAATTTATTCTTAATGCATGACCTTGTCAATGATTTAGCGCAAATTGCATCTTCAAAACTTTGTATCAGGTTGGAAGAGAGCCAAGGATCTCATATGTTGGAACAAAGTCAACACTTATCATATTCAATGGGATATGGTGGTGAGTTTGAGAAATTGACACCCCTCTACAAATTGGAGCAGCTGAGGACATTGCTTCCGACATGTATTGATCTCCCTGATTGTTGTCACCATCTAAGCAAGAGGGTGCTACATAACATACTGCCAAGACTAACATCCTTAAGGGCATTATCGTTGTCATGCTATGAGATTGTTGAGTTGCCAAATGACTTGTTTATCAAATTAAAGCTCCTCAGATTTTTGGATATTTCTCGGACAGAGATTAAAAGGTTGCCAGATTCCATTTGTGCATTGTATAACTTAGAGACACTTCTCCTGTCATCTTGTTATGATCTTGAGGAGCTACCGCTGCAGATGGAGAAGCTGATTAACTTGCGTCATCTTGACATAAGCAACACTCGTCTCTTGAAGATGCCGCTACATCTGAGCAAGTTGAAAAGCCTCCAAGTGTTAGTGGGAGCCAAATTTCTTATAGGTGGTTTGAGAATGGAAGATTTGGGTGAAGTACATAACTTGTACGGATCTCTATCAGTTGTAGAGTTGCAAAATGTGGTTGATAGAAGGGAAGCTGTGAAGGCAAAGATGAGGGAGAAGAATCACGTCGACAGGTTATATTTGGAGTGGAGTGGAAGTAGTAGTGCCGACAATTCACAAACAGAAAGAGACATACTTGATGAGCTACGCCCCCATAAAAACATAAAAGTAGTCAAAATCACTGGATATAGAGGGACAAACTTTCCCAATTGGCTAGCTGATCCTTTGTTTCTTAAGCTGGTAAAATTGTCTCTTAGAAACTGCAAGAACTGTTATTCATTGCCAGCACTAGGACAACTCCCTTTTCTGAAATTCCTTTCGATTAGAGAGATGCATGGAATAACAGAGGTGACGGAAGAATTCTATGGCAGTTGGTCCTCCAAAAAGCCTTTTAACTGTCTTGAGAAGCTTGAATTTAAAGATATGCCGGAGTGGAAGCAATGGGACCTACTAGGAAGTGGAGAGTTCCCTATACTTGAGAAGCTTTTGATTGAAAATTGCCCTGAACTCAGTTTGGAGACGGTACCCATCCAACTTTCAAGTTTAAAAAGTTTTGACGTAATTGGTTCTCCCTTGGTTATAAACTTTCCTTTAAGCATACTGCCCACTACCTTGAAGAGAATAAAGATATCTGATTGCCAGAAATTGAAATTGGAACAGCCAACTGGTGAGATTAGTATGTTTCTGGAGGAATTGACACTGATTAAATGTGATTGTATAGATGATATATCACCTGAGTTACTCCCAAGAGCACGCAAATTGTGGGTACAGGATTGGCACAACCTTACTAGGTTTTTGATTCCTACTGCCACTGAAACTCTCGATATTTGGAATTGTGAGAATGTTGAAATACTTTCGGTGGCATGTGGGGGGACCCAGATGACGTCACTGACTATTGCCTACTGTAAGAAGCTGAAGTGGCTGCCAGAACGTATGCAGGAACTCCTTCCATCTCTTAAGGAACTGCATCTGTCTAATTGTCCAGAAATAGAGTCCTTTCCTGAAGGTGGATTGCCCTTCAATTTACAACAACTTGCGATCAGATATTGCAAGAAACTGGTGAACGGCCGAAAGGAGTGGCATTTACAGAGACGACTCTGTCTCACAGCGTTAATCATCTACCATGATGGCAGTGACGAAGAGATTGTTGGTGGTGAGAATTGGGAGTTGCCTTCCTCTATTCAAAGGCTTACCATAGTGAATCTGAAAACATTAAGCAGCCAACATCTCAAAAACCTCACCTCTCTTCAATATCTATTTATTAGGGGTAATTTACCTCAGATTCAGCCAATGCTGGAACAAGGCCAGTGTTCGCACCTCACTTCGCTTCAAAGTCTACAAATCTCCTCCCTCCAATCACTTCCTGAATCGGCACTGCCCTCCTCCCTCTCTCACCTGGAGATCTCCCATTGCCCTAATCTCCAATCACTTCCTGAATCGGCACTGCCCTCCTCCCTCTCTCAGCTGACCATCAATAATTGCCCTAATCTCCAATCACTTTCCGAATCAACACTGCCCTCCTCCCTCTCTCAGCTGGAGATCTCCTTTTGCCCTAATCTCCAATATCTTCCACTAAAAGGGATGCCCTCTTCCCTCTCTGAACTATCTATTTACAAATGCCCATTGCTCAAACCACAACTAGAATTTGACAAGGGGGAATACTGGCCAAATATTGCTCAATTTCCCACCATAAAGATCGATAGGGAATGCATGTGATGATTAAAACGAATGGCTCTTCAACTTATGTAAGCTATTCTTTTCCCTTGGAAGCTTTTTATTTCCGTTTACTTCACTTTGCTTTTTTGTTAATTCTTTTCATTTTTAATCGCTAATTCTAAAAACATGCCTTGCACGTTTATCCCAAAATATTTCATATAATTTTTGTATAGCAAATGACATATTTTATCCCCTTCAAATCTATTGGTATTTACTAAAAAAATATCCATCATTTTTACGATTGAAAGTACATATATTTTCCTAATAAAAAGAATACACCCTTAATTATAAAATTGATTAAAAGTGCATAAAGTAAAAATAACTAATTGTTGTACAAATACAATATTCAAATGAGGATACTAAAATACCACATGTTAGGTTAATGTATACAAAACAACTAACCAATGTAAATTCTCAATATTAATAACCATAAAACAATAAAAAGTTATACAATAACAACAAACAAGAAACATAAACATTAAAAAAAATAAAAATAAAAAGAGTCAAGAAAAGAGAGCATAGAGATTCTCTTTTTTTTTCAATTTGTTTTAACTGTGTACAATATTGTGTCAAATGTCACTATTCATAGAATAAGATTGAAGAACACTTAAGAGTTGTTATGAATATAACATTAATCCATTTGATCATGGATGAGAGGTGGTAGTAAAAATCATAATGAATATAATTAGTGGGAGTTGCATATATTTACATAATGAAGAGTTAGTGGTAATAAATCTATGTAATATACTTAACATTAATACAAATATTTATATACAAAAAAGAATATAATATAGATCTATATGATTTTTTTCCCTTTTTGTTAAGGGCAAACATAACCATTCATATTTCCAATTGGAAGATGAACAAAACAAAAAATTCATGAATTTAACTCCAGCATGAAATTAANAAAAAGAATACATATTCAAGAAACTTTATGGTGGTTGATTCATGTACTAATATGATGATTTACTATTAAATATTTAATTTATTAAATAGTTTATAACATTTAAATTTAATGTAGAAATAGAATCATAATCTAACTTTTGCCTAACATTCTCCCCACTTTTAGTCAATTTTCCTTTTTGCATTATAATATGGATTGCACACAACAACTTCTATTTAAAAAAGGAAAGACAAAATTAGAAAAAATAGTATCAATAAATTGGAATGAAAAATGTAAAATTGTAATAAGAGGAAGAGAAGAAAAAATCAGTAGAATATATTTACCTAACCATGAATAAGGAAGTATGTTGAGTAAAAGAAGAGTAATGCAAAAGTGTGAGCTGATATATGGAGGAAATTGACTAATATAATAATCTGTATTGAATAATGAAAAGACCAATCTTTAATTTCTGCCAAATTTTTATTACGAGCTCAAGATAATATAAATTTATTAATTTAATAATGACATAAATAAATAACATACATTGTATGTATGTGTAGTTAATGGGGTAGTGAAAAAGACTCTTTCTAAAATTATTTTTCTCTTTAAAGTATTTTTAAACTTATAATATTAGTGCTATAGAAAATGCTATAGAAAATCATAAGCTACTGCAAGTTTTTATAGTAATTATTTTATTAAATTATATACCTATTATTTATAATCATTCCGGAAATATATAAACAATTTTGGAAAGCTTGTAAAATGAGAAATTATTATTTCTTATCGTTATTTTAGCTATTATTAACATTTAGTATGACAACATTTAAAAAGTAGCCTCCTTTAAATATTAACGGTTTCTAAATTAACGTAGCCAATAATTTGAAATGTTTTGACGTTTTGTTTATCTAAACACGCAGAATTTTGTTTTTGAACTTGAAACATGTAATTTTGCCTTGGGTGAATTATAATTTTATTTTTACTATTTAATTAATTAAATATTTTATAACATTTTAATATAATGAACTGGTAAAATGGTAATCCAACTTTAAAGGTTGGAGCTTCCCACTTTTAGTAATATATGATATATGATATATGATATATGATGATATATGATGTTGTTGTGCTCATCATCAAACACATAGCTTTATATTTTTGCTTCATAGAGAATCTAAATTTTTTAAAAGATAATCAGGGATAACGATCACAAGTTTTAGGAAATAAGTGCAACTTCCATTGTCAGAATAAGTAGACGTTATATAATTCTATATTTCTTGGATAGCGACCAGACAACTACACAGGGCAATGCTTGATGTAAGAGCTTCAGATATACATTTGTAGGGTGTTTATCCAACGAGTAATAAAATCACAGTCTTCTGATATCTTCTCTTGCATATACTTTTGCAATTTTAAACTTTATTTTGAATTGTGTTGTTGCTAACTTGAACATGTTCTGTGCTTAATCAGATGTGGATTTTGAAGGGCTAGTACGACAAGTCTGGTCCATCCATTGTCCACAAGAAGTGCTTATAAGGTGTTGCTGCTATTTTTTACATATGTTCATGAGTTCTTTTTTATTTTTTTGTTCTTTCCGCTAAAGTTATTATGTCATCCACAGTGAACTCAGGTCTGCTGTTATAGGCAAGTCTTCGAGATGGGGCAATACCGCTGATACTATTTCATGTTTCCAGTGCAAGCCTCTTTTGTAAGTAGACAAACTCGATTTGTAAATATGTTTGGGACTCAACTAGTGGTTAGAGTACTGATTTTGTAAGATTTGTGTACGAAAAATCAAATTAGAAAGATAACTTGCGATGATTGAATAAGCTCAAAGAAGTGGATGTGAATTTCAAATCGTGTCTTTTTTTTTTCATGTGTGTGTGTGAGAAATTTGAAGTGTTGTTTAGATTTGTTGGAATAGTCTAAATAGATTGTCTCTATAAAATTTGAAGACATTTGATGGAGATCTGATTAGATGATTTTGAACAACAAGTGCAACTGAAGATGGATATGTATATTATTTTCGTAGTTAGCAACGTGTTATATATTTTTATAGTGGATATATTGTTTTCTCATTCAGTGTTTGATATTCACATTGGAGTCCGACTAAATTTGAATTTGCGCATTTCAAGGGGCGGCGTTCTTGACATGATTTTTTCCATTCTACGGCTCGTACGTGCTCTTAAATTCTAATTAAGGATAGAAAAATCTCAACTATTTCACCACAACTCAAATTAGTTATATTGGGTATACAAGTGTAAACTAAGAAATATAACTATGTAGGTGCAAATATTTTTGAAAAACCCCAAATAATAAGCCAAAATAAAGAAAGAATACTAAATCGTACACTCATTACTACAACAACTAAAAAATATTTATAATCATTGAACTCCTATGCACATTCTACTGCAAAACATAAGTAGAAGAAGTTCAAATTTTGGCCATGAAATGACACCATTTTCTTACACTCAAAATGATCTTAAATTT

>Rpi-R2-like

TAAGACTTTTCTCTATATGTGTTTTTCCCCAAGTTGTATAATGGTTGTTGAAGATGCTTTAATTAAAAAAAAAAACCTTTTGTTTAGTGGAAAATTTCAAAAAGCTTTAGTACATCTTTGTCGTTTTATCCAATCGTAATTCTTTATTCAGAAACCACATGTTTTTTTTCTAATCTTACTTTTATGTCTATCACCCATTTTCCAATATACAGCCTACTCTTTTTTTCAATCAAAACTAGTATTCCTAAAGATGGCTGATGCCTTTCTATCATTTGCAGTTCAAAAATTGGGTGATTTCCTAATACAGAAAGTTTCCCTGCGTAAAAGTCTCAGAGATGAAATTAGATGGCTGATCAATGAGCTACTCTTCATACGGTCTTTCCTCAGAGATGCAGAACAAAAGCAGTGCGGAGATCAAAGAGTTCAACAATGGGTGTTTGAGATCAACTCTATTGCTAATGATGCTGTTGCTATACTCGAGACTTATAGCTTTGAGGCTGGTAAAGGTGCTAGTCGTCTCAAGGCTTGCACTTGCATATGTAGGAAGGAGAAGAAATTCTACAATGTTGCCGAGGAGATTCAATCACTCAAGCAACGAATCATGGATATCTCTCGCAAACGAGAGACTTATGGTATTACAAATATCAATAATAATGCAGGAGAAGGGCCAAGTAATCAGGTTACAAAATTGAGGAGAACTACCTCATATGTAGATGAACAGGATTACATTTTTGTTGGCTTTCAGGATGTTGTACAAACATTTCTAGCTCAACTTCTGAAAGCAGAGCCTCGTCGAAGCGTCCTCTCCATTTATGGAATGGGGGGTTTAGGCAAGACCACTCTTGCCAGAAAACTTTACACCAGTCCTGATATACTCAATAGCTTCCGTACACGCGCTTGGATATGTGTCTCTCAAGAGTACAACACAATGGATCTTCTTAGGAATATCATAAAATCCATCCAAGGTCGCACCAAGGAAACTCTAGATTTGTTGGAAAGGATGACAGAAGGAGATCTTGAAATTTATCTTCGTGATTTATTGAAAGAACGCAAATACCTTGTGGTGGTTGATGATGTATGGCAGAGAGAAGCATGGGAGAGTTTGAAAAGATCATTCCCGGATGGCAAGAATGGCAGCAGAGTCATTATTACCACGCGCAAAGAGGATGTCGCTGAAAGAGCAGACGACAGAGGTTTTGTTCATAAACTTCGTTTCCTAAGCCAAGAAGAAAGTTGGGATCTCTTTCGTAGGAAACTACTTGATGTTCGAGCAATGGTTCCAGAAATGGAAAGTCTAGCTAAGGATATGGTGGAAAAGTGTAGAGGCTTACCTCTTGCAATTGTTGTATTGAGCGGACTACTTTCGCATAAAAAGGGGCTAAACCAATGGCAAAAGGTGAAAGATCACCTTTGGAAGAACATTAAAGAAGATAAATCTATTGAAATCTCTAACATACTATCCTTAAGCTACAATGATTTGTCAACTGCGCTCAAGCAGTGTTTTCTCTACTTTGGTATTTTTCCAGAAGATCAAGTGGTAAAGGCTGATGACATAATACGGTTGTGGATGGCGGAGGGTTTCATACCCAGAGGAGAAGAAAGAATGGAGGATGTGGCTGACGGCTTCTTGAATGAACTGATAAGACGAAGCTTGGTTCAAGTAGCTAAAACATTTTGGGAAAAAGTTACTGACTGTAGGGTTCATGATTTACTTCGTGATCTTGCGATACAAAAGGCATTGGAGGTAAACTTCTTTGACATTTATGATCCAAGAAGCCACTCCATATCCTCTTTATGTATCAGACATGGCATTCATAGTGAAGGAGAAAGGTACCTCTCATCACTTGATCTTTCTAACTTGAAGTTGAGGTCAATTATGTTCTTCGATCCAGATTTTCGTAAGATGAGTCATATAAACCTCAGGAGTGAGTTCCAACATCTATATGTGTTGTACTTGGATACGAATTTTGGGTATGTGTCTATGGTACCTGATGCCATAGGAAGTTTGTACCACCTCAAGTTGTTAAGATTGAGAGGTATCCATGATATTCCGTCTTCCATTGGCAACCTCAAGAATTTACAAACACTTGTCGTTGTAAATGGTTACACATTTTTTTGCGAACTACCCTGCAAGACAGCTGACCTAATAAATCTAAGACATTTAGTTGTTCAATATACAGAGCCTTTAAAATGTATAAACAAACTCACTAGTCTTCAAGTTCTTGATGGTGTTGCTTGTGATCAGTGGAAAGATGTTGACCCTGTTGATTTAGTCAATCTTCGAGAATTAAGCATGGATCGTATCAGGAGCTCTTACTCCCTAAACAACATTAGCAGCTTGAAAAACCTTAGCACTCTCAAATTGATTTGTGGAGAACGTCAATCATTTGCATCCCTTGAATTTGTTAATTGTTGTGAAAAGCTCCAGAAATTGTGGTTACAAGGGAGAATAGAGGAACTGCCTCATCTGTTTTCAAACTCCATCACAATGATGGTTCTGAGTTTCTCAGAACTGACAGAAGATCCGATGCCTATTTTGGGAAGGTTTCCAAACCTAAGGAATCTCAAATTAGATGGAGCTTACGAAGGAAAAGAAATAATGTGCAGTGATAACAGCTTCAGTCAACTAGAGTTCCTTCATCTTCGTGATCTTTGGAAGCTAGAAAGATGGGATTTAGGCACAAGTGCGATGCCTCTGATTAAAGGTCTTGGTATCCATAACTGTCCAAATTTAAAGGAGATTCCTGAGAGAATGAAAGACGTGGAGCTGTTGAAGCGGAATTATATGTTGTGAAGCTTTTCTGCCAAGCACATTGGTTATTAATTGAGTGGTTTTAGTGTTGATTTCTTATTATTGTTTTAAGCTTTTTGAGTGTGTAATTGGTTTGAACATTATTGTTTTAATTAATTGGTCTACTGTATGTTCTCATGCTTATCCACATTTAAGACAATGCTTTATATGTTAAAATGAAATTAAAAATACTAGTATATGGTACTCTCTCTTGTCCACAATTTCGTATATTTTTTGTTCCTCTTCATAAAAA

>Rpi-R1

TGTCCTGATTAGGAGACTTATTATTTCATTTCATATATAGATAGGTCCCTTGAGAACTAGAAAGATTAAATTAAAGATTGAGATCCAATAATGCATATGAACACAGAACATTTGTCTTTTTTCCAAAGGGGACCATATATATATAAGATGTCATTGTGCTTTATGTATGGAAGAGAGAATAACGATCTCATATATATATCTCATATATATATATATCATCTAAAATAAGATGTTTTAAACCATCTGGTATTCGGTATACAATTTACACTAAAAAGACCAAACAGGTGGGAAGGACACAAACATTAGATCAAAAATTAAGGTTAAGTGATTCAGATATCAAGAGGAACAATATACTAATTGGAACAAATTAAAGTATCCTCACTTACAATGGTCATATATAGAAGCTACTTAGGTAATACTCTCACTATCCCTAATTATTTGTCCACTTTTAAATTAGCACACCTATTAATAAAACAATTATTGGCATAGTGAGTTTACCATTTTACCTTTTTAATTATGAAGCGAATGAATTAAAAACTTAAGATATTAAAAAAATTCTGCCTTTAACAAAGTAATTATTTGAGGGTATAATAGGTAAAAAGAAATTGTCCTTTTTTTATTTGTCAAAATGAACAAGTAGTTAGGGACAACTAAAAAAGGAAAAATGGATGAGTAATTAGGAACGGAGGGAGTATAAAACACTGTCATCACTCAAAAAATATGAGTATCTTGACTTGCACAACATAGGTACTTAATCAAAGACTCAATATACAAATCTCTAAAGTAAATTTGTATTTGTATATACAGTCTCTTTGAAAGCCCAATTTGTATAAAATATTTAAATGCAGCTAGATATACAAACGGAAATTAGCATAGCAACTGAAACTATAGATATAGAACATAATTAGGCAATGACTTTGTTTTTTGTTTGTCTGCCTCACACTTTATTTGACTGCCTTCCTTGAATACTTTGAATATTCTAAGTACGCCAGCTATAAGGTGAAGAAAGAATTAAACTATAATACTCTGTATTGCTCTTCTTCCATAATAGTGTAACAAGGATGAATTTCAACAATGAATTGTCTGATCTGAAAAATCGCTTCCTATTTAGGACGCTGAGAGCCCAGAAATGCTCGGATGTTGCAAGAGATCGAATAGATTTCTTTATATGGGAGTTAAAATTCCTTAATTGTTTTCTCCATTTGCAGAGCTTCGCTTTTGCAAGTGAATGTGGTATGCTAGATATCTCACAGAAAATGATAGAAATTTGCAAGAGGTTTAATACACCACCTCCACATAATTCATTTGCATACTGGAAGGAGGTAATTTGCAAGAGGCTGTGCGCTATTAGCATCCAGCCGGATGCTAGTTCAGATGATGGATTTGCATGCTGGAAGAAAGTAATTTGGAAGACTAAGCAAGAATTCAGAGCTAAATACTCCTTTCCAAAAACACTACTTGCAGACAACAAGGTATATGATGATGATGATACTAATCCCAAATTTGTGATGGAATTCATCGATGCTGTTGTGGGGAATCTCAATGTTCTAGTCAAGATCAATGATCCATCTTCATTGCTTTTTGTTCCAGGACCCAAGGAACAAATAGAACAAGTGTTAAAGGAGTTGAAGTTATTGAGATTTTTTGTCTGCTTTGTTTCAAACAAATGTATAGAGCCTCAATACCAACATACTACTTTTTATACTCACGCTTTAATTGAGGCTAGCCACATCGCAATGGTTGTGTGGTTGAATTTGCCAATCTATGGAAACAGAAATCAAGACTTGGCTTCAAGTGAAGTTAGTTGTTTGCTTTCTGATTTCATGGAAATGAAGATTAAGTCCATTCAGCCAGACATCAGCCGCAACAATATTTATATTGATGTCTTGAGGGCGTTGAAGTCAACCATACCACAAGCTCAAGATAAGCATGCTGCTGAGAGTGGCATTGTGGAGACTCCAACACACAATCTGATGGTTGGTTTGAGTGATCAAATGGCCAACCTTCAGGAGATGCTCTGCCTTCTAAGAGACAATCTCATTCATCTGCCAATACTAGATCTGGAATTTCATCTTCAAGATATGGATTCTGTTATTGTTGATGCCGGACTTCTTATTTACTCATTATATGATATCAAGGGGCAGAAGGAAGACACAACATTGGAGGATATCAACCAGGCACTTGGTTTTGATCTTCCCAGAAACATTGAGCCTATCAAGGCAATGATCAACCTTGTCATGCAAAAGGCATTTCAATGTAACTTGCCAAGGATTCATGGACTAGGTTATGTCGATTTTCTATTGAAAAACCTGAAGGATTTCCAAGGCCGTTATTCAGATTCACTCGATTTCCTCAAGAATCAACTTCAAGTTATTCAAACTGAATTTGAGAGCTTGCAACCTTTCTTGAAGGTTGTCGTAGAAGAGCCACACAATAAGCTCAAGACACTGAATGAAGATTGTGCTACACAGATAATTAGGAAAGCATATGAGGTGGAATATGTAGTTGATGCTTGTATAAACAAAGAGGTTCCTCAGTGGTGCATCGAGCGTTGGCTCCTGGATATCATAGAGGAGATTACTTGTATCAAAGCAAAGATTCAGGAAAAGAACACGGTTGAGGATACAATGAAGACTGTCATTGCTCGTACATCATCAAAACTGGCAAGGACTCCAAGGATGAATGAAGAGATTGTTGGGTTTGAGGATGTCATAGAAAATTTAAGAAAAAAACTACTGAATGGAACCAAAGGGCAAGATGTCATTTCAATTCACGGCATGCCAGGTTTAGGTAAGACGACTTTAGCCAACAGTCTCTATTCTGACAGGTCAGTTTTTTCTCAATTTGATATTTGTGCACAATGTTGTGTGTCTCAAGTATATTCTTATAAGGACTTAATATTGGCCTTGCTACGTGATGCTATTGGTGAGGGTTCTGTGCGTAGAGAACTTCATGCCAATGAATTAGCTGATATGCTTCGCAAAACTCTATTGCCCCGAAGGTACCTTATCCTTGTTGATGACGTGTGGGAAAATAGTGTTTGGGATGATTTAAGAGGTTGTTTTCCAGATGTCAATAACAGAAGCAGAATCATTCTAACAACAAGACATCATGAAGTTGCCAAATATGCTAGTGTTCATAGTGATCCCCTTCATCTTCGTATGTTTGACGAAGTTGAAAGTTGGAAGTTGCTTGAAAAGAAAGTGTTTGGTGAAGAAAGCTGTTCCCCTCTCCTAAAAAATGTTGGGCTAAGAATAGCAAAAATGTGTGGACAACTACCTCTTTCAATTGTTCTGGTGGCTGGTATTCTGTCAGAGATGGAAAAGGAAGTAGAATGTTGGGAACAAGTGGCCAACAATTTGGGTTCCTACATTCACAATGACTCAAGAGCCATTGTAGACAAAAGTTATCATGTTTTACCTTGTCATCTTAAGTCTTGCTTCCTTTATTTTGGAGCATTTTTAGAAGATAGAGTGATTGACATTTCAAGGTTAATAAGGCTATGGATATCAGAAGCATTTATAAAAAGTAGTGAAGGCAGGAGGTTGGAGGATATAGCAGAAGGTTACTTGGAGAATCTTATTGGAAGAAATCTAGTAATGGTTACTCAGAGGTCCATTTCAGATGGTAAGGCGAAAGAATGTCGCCTTCATGATGTATTACTCGACTTCTGCAAGGAAAGAGCAGCTGAGGAGAATTTTCTACTATGGATAAATAGGTAATATGATAAGTAACTGTACTTTCAATCAATCAAGTATTTCAAGTTATATCTGAAAATTAATGATATGATTTTGCTAATTGATATATTCAGGGATCAGATTACCAAACCTTCTTCCTGTGTTTACTCTCACAAGCAGCATGCTCACTTGGCCTTCACTGAAATGCATAATCTTGTAGAATGGAGTGCGTCTTGCTCATTTGTTGGCTCGGTAGTACTTTCCAATAAATATGACTCATACTTTTCCACTCGTGACATATCCTCACTACATGATTTTTCAATTTCACGCATTTTACCAAATTTCAAGTTTCTAAAAGTGTTAGATTTGGAACACCGGGTTTTTATTGATTTTATTCCAACTGAGCTTGTTTACTTGAAGTATTTTTCTGCACACATTGAACAGAATTCAATTCCTTCAAGCATATCCAATCTTTGGAACCTTGAAACTCTTATATTAAAAAGTCCAATATATGCGTTACGTTGCACGCTACTACTACCTAGTACAGTTTGGGATATGGTTAAATTGAGACATCTGTATATTCCTGACTTCAGCACAAGGATTGAAGCAGCATTACTTGAGAACTCTGCAAAACTTTATAATTTGGAAACCCTTTCCACTCTATATTTCTCTCGTGTTGAGGATGCAGAATTGATGCTGAGAAAAACACCTAATCTTCGAAAACTGATATGTGAAGTTGAATGTTTAGAATACCCCCCTCAGTACCATGTGTTGAATTTTCCAATACGGCTTGAAATACTAAAGCTTTATCGATCAAAATTTAAAACCATCCCCTTTTGCATCTCTGCACCAAATCTCAAATACTTGAAACTCTGTGGCTTTTCCCTGGATTCTCAGTACTTATCAGAAACTGCTGATCATCTCAAGCACCTTGAGGTACTCATACTGTACAAGGTTGAATTTGGTGATCATAGGGAATGGAAAGTGAGCAATGGCAAGTTCCCTCAACTCAAAATCTTGAAACTAGAATATTTGTCCTTGGTGAAATGGATTGTAGCTGATGATGCCTTTCCTAACCTTGAACAATTGGTTTTGCGTGGATGTCAAGATCTTATGGAGATCCCTTCTTGTTTCATGGACATCCTTTCTCTCAAGTACATCGGGGTAGAATACTGCAATGAGTCGGTTGTCAAGTCAGCCTTGAATATACAAGAAACACAAGTCGAAGATTATCAAAATACTAATTTCAAGCTCGTTCTCATCGAGGTACACTACTGAAAAAAGCTTTATTCTGCATGATTTTGATGAATCAGAAATCGCCTAAATTTTACAAACTGTTTTCTCAGTTATCTTTACCTCGTGGCCTCGTTTTACATTTGGGTTCTTCTCTTAGTTTTCTTTGCAGAAAAAGGCGTGGAAATTAAATTTAACTGATGCGGAAGATATGCACAATGCAGTAAAAAATATTCTTGCAGAAATAAGATAGGTACTACTTTTTTTTTTTTCTTTCCTTTTTTTAAATACACCAAATAGATAGATTCATCTTTTTTGTCTTTTCGATATGAAAGGGATAGAATCAGTTTCATCTGATGAGAAAGAGAAGAAACTTACTGTGACCGGAGATGTGGATGCTGATGAAGTTCAATTAGTTGTGGAGAAACTGAGAAAGCGTGGCATGCCAGGGTTGTAGTCCCAACTTGTCAACACAAATGTGCTATACTCATTTTGCTTACTGTAATACCATTTCATGACACACACACACAAACATTAACTGTAGTAAAGTTTTGATGGATCAGTAAATCTGAGTTCAACCCATTGTAATCCGTTCAAATTCAACTCAAAAAATTCCCATTGAGTTATTCTTTAACAGGGTATCCAGAGTTTGTAGCTGGAGCAATTTGGAATATCACATGTAATTTCTTTATGAGTTAATTCGTTTAATAAAAGATTCTGTAAAACGTCCAACGGCTGTTGCATTCATTGTAAACTAAATATATCTCAGTATGTAACTATTGAACAAATTTTTCATTTTAGTCCCTGAGGTTTGATGTAAGTCATTAGATTTTACGGATCCTGAAGTGAATGGTTTTAGCCTTTTCTATTTTCTTATGAGTTCACCAAAATGTTGTGATGCCACTCTGCTACATGTTAGAGAAATGAGAATGTTAGCACCCGAGAGTATGGCCTAGCGGTCAATCAATGAAGCAGGTGAAAACAACAAAAGCAAAAAATACTAAGAGATTTCTTCACATCTATCTAAGTACCGCTAAGCAAAGATACTGTAATGACCCTCCTGGTCATTTATGTGTCTTGCCTTCTGTGTGTCGTTTAGAGTGTTCCTATAGCGACCCCAAGTCATTTATGACTTGCTGGGACTAACGGTTCGGTCACATGGTCGTTCGTTTGGTTTTGGTGCGAGTTTTTGTGTTTTGGAGCTTATGAATCTTGAACGATGATTTTCGATCAAAAATTCAAGAAGATGACATCGGAATCCATTTCTAACGATTCCATCAGCTCCGGAAGGGTCATTTTAGGCTAGTAGCTTGGTCGGCATGACTCCCGGTGCGATTAGGCCTTTTAACTTTAAGTTTAAGCCTAAGTTTGACTTTGGTCAACATTCTGAGTAAACGCGCTCGGATGAGAATTCCGTCAGTGCGGTTAGCTCCGGAATGTCAAGTTTGGT

> *Rpi-R1^ΔT4109^*

TGTCCTGATTAGGAGACTTATTATTTCATTTCATATATAGATAGGTCCCTTGAGAACTAGAAAGATTAAATTAAAGATTGAGATCCAATAATGCATATGAACACAGAACATTTGTCTTTTTTCCAAAGGGGACCATATATATATAAGATGTCATTGTGCTTTATGTATGGAAGAGAGAATAACGATCTCATATATATATCTCATATATATATATATCATCTAAAATAAGATGTTTTAAACCATCTGGTATTCGGTATACAATTTACACTAAAAAGACCAAACAGGTGGGAAGGACACAAACATTAGATCAAAAATTAAGGTTAAGTGATTCAGATATCAAGAGGAACAATATACTAATTGGAACAAATTAAAGTATCCTCACTTACAATGGTCATATATAGAAGCTACTTAGGTAATACTCTCACTATCCCTAATTATTTGTCCACTTTTAAATTAGCACACCTATTAATAAAACAATTATTGGCATAGTGAGTTTACCATTTTACCTTTTTAATTATGAAGCGAATGAATTAAAAACTTAAGATATTAAAAAAATTCTGCCTTTAACAAAGTAATTATTTGAGGGTATAATAGGTAAAAAGAAATTGTCCTTTTTTTATTTGTCAAAATGAACAAGTAGTTAGGGACAACTAAAAAAGGAAAAATGGATGAGTAATTAGGAACGGAGGGAGTATAAAACACTGTCATCACTCAAAAAATATGAGTATCTTGACTTGCACAACATAGGTACTTAATCAAAGACTCAATATACAAATCTCTAAAGTAAATTTGTATTTGTATATACAGTCTCTTTGAAAGCCCAATTTGTATAAAATATTTAAATGCAGCTAGATATACAAACGGAAATTAGCATAGCAACTGAAACTATAGATATAGAACATAATTAGGCAATGACTTTGTTTTTTGTTTGTCTGCCTCACACTTTATTTGACTGCCTTCCTTGAATACTTTGAATATTCTAAGTACGCCAGCTATAAGGTGAAGAAAGAATTAAACTATAATACTCTGTATTGCTCTTCTTCCATAATAGTGTAACAAGGATGAATTTCAACAATGAATTGTCTGATCTGAAAAATCGCTTCCTATTTAGGACGCTGAGAGCCCAGAAATGCTCGGATGTTGCAAGAGATCGAATAGATTTCTTTATATGGGAGTTAAAATTCCTTAATTGTTTTCTCCATTTGCAGAGCTTCGCTTTTGCAAGTGAATGTGGTATGCTAGATATCTCACAGAAAATGATAGAAATTTGCAAGAGGTTTAATACACCACCTCCACATAATTCATTTGCATACTGGAAGGAGGTAATTTGCAAGAGGCTGTGCGCTATTAGCATCCAGCCGGATGCTAGTTCAGATGATGGATTTGCATGCTGGAAGAAAGTAATTTGGAAGACTAAGCAAGAATTCAGAGCTAAATACTCCTTTCCAAAAACACTACTTGCAGACAACAAGGTATATGATGATGATGATACTAATCCCAAATTTGTGATGGAATTCATCGATGCTGTTGTGGGGAATCTCAATGTTCTAGTCAAGATCAATGATCCATCTTCATTGCTTTTTGTTCCAGGACCCAAGGAACAAATAGAACAAGTGTTAAAGGAGTTGAAGTTATTGAGATTTTTTGTCTGCTTTGTTTCAAACAAATGTATAGAGCCTCAATACCAACATACTACTTTTTATACTCACGCTTTAATTGAGGCTAGCCACATCGCAATGGTTGTGTGGTTGAATTTGCCAATCTATGGAAACAGAAATCAAGACTTGGCTTCAAGTGAAGTTAGTTGTTTGCTTTCTGATTTCATGGAAATGAAGATTAAGTCCATTCAGCCAGACATCAGCCGCAACAATATTTATATTGATGTCTTGAGGGCGTTGAAGTCAACCATACCACAAGCTCAAGATAAGCATGCTGCTGAGAGTGGCATTGTGGAGACTCCAACACACAATCTGATGGTTGGTTTGAGTGATCAAATGGCCAACCTTCAGGAGATGCTCTGCCTTCTAAGAGACAATCTCATTCATCTGCCAATACTAGATCTGGAATTTCATCTTCAAGATATGGATTCTGTTATTGTTGATGCCGGACTTCTTATTTACTCATTATATGATATCAAGGGGCAGAAGGAAGACACAACATTGGAGGATATCAACCAGGCACTTGGTTTTGATCTTCCCAGAAACATTGAGCCTATCAAGGCAATGATCAACCTTGTCATGCAAAAGGCATTTCAATGTAACTTGCCAAGGATTCATGGACTAGGTTATGTCGATTTTCTATTGAAAAACCTGAAGGATTTCCAAGGCCGTTATTCAGATTCACTCGATTTCCTCAAGAATCAACTTCAAGTTATTCAAACTGAATTTGAGAGCTTGCAACCTTTCTTGAAGGTTGTCGTAGAAGAGCCACACAATAAGCTCAAGACACTGAATGAAGATTGTGCTACACAGATAATTAGGAAAGCATATGAGGTGGAATATGTAGTTGATGCTTGTATAAACAAAGAGGTTCCTCAGTGGTGCATCGAGCGTTGGCTCCTGGATATCATAGAGGAGATTACTTGTATCAAAGCAAAGATTCAGGAAAAGAACACGGTTGAGGATACAATGAAGACTGTCATTGCTCGTACATCATCAAAACTGGCAAGGACTCCAAGGATGAATGAAGAGATTGTTGGGTTTGAGGATGTCATAGAAAATTTAAGAAAAAAACTACTGAATGGAACCAAAGGGCAAGATGTCATTTCAATTCACGGCATGCCAGGTTTAGGTAAGACGACTTTAGCCAACAGTCTCTATTCTGACAGGTCAGTTTTTTCTCAATTTGATATTTGTGCACAATGTTGTGTGTCTCAAGTATATTCTTATAAGGACTTAATATTGGCCTTGCTACGTGATGCTATTGGTGAGGGTTCTGTGCGTAGAGAACTTCATGCCAATGAATTAGCTGATATGCTTCGCAAAACTCTATTGCCCCGAAGGTACCTTATCCTTGTTGATGACGTGTGGGAAAATAGTGTTTGGGATGATTTAAGAGGTTGTTTTCCAGATGTCAATAACAGAAGCAGAATCATTCTAACAACAAGACATCATGAAGTTGCCAAATATGCTAGTGTTCATAGTGATCCCCTTCATCTTCGTATGTTTGACGAAGTTGAAAGTTGGAAGTTGCTTGAAAAGAAAGTGTTTGGTGAAGAAAGCTGTTCCCCTCTCCTAAAAAATGTTGGGCTAAGAATAGCAAAAATGTGTGGACAACTACCTCTTTCAATTGTTCTGGTGGCTGGTATTCTGTCAGAGATGGAAAAGGAAGTAGAATGTTGGGAACAAGTGGCCAACAATTTGGGTTCCTACATTCACAATGACTCAAGAGCCATTGTAGACAAAAGTTATCATGTTTTACCTTGTCATCTTAAGTCTTGCTTCCTTTATTTTGGAGCATTTTTAGAAGATAGAGTGATTGACATTTCAAGGTTAATAAGGCTATGGATATCAGAAGCATTTATAAAAAGTAGTGAAGGCAGGAGGTTGGAGGATATAGCAGAAGGTTACTTGGAGAATCTTATTGGAAGAAATCTAGTAATGGTTACTCAGAGGTCCATTTCAGATGGTAAGGCGAAAGAATGTCGCCTTCATGATGTATTACTCGACTTCTGCAAGGAAAGAGCAGCTGAGGAGAATTTTCTACTATGGATAAATAGGTAATATGATAAGTAACTGTACTTTCAATCAATCAAGTATTTCAAGTTATATCTGAAAATTAATGATATGATTTTGCTAATTGATATATTCAGGGATCAGATTACCAAACCTTCTTCCTGTGTTTACTCTCACAAGCAGCATGCTCACTTGGCCTTCACTGAAATGCATAATCTTGTAGAATGGAGTGCGTCTTGCTCATTTGTTGGCTCGGTAGTACTTTCCAATAAATATGACTCATACTTTTCCACTCGTGACATATCCTCACTACATGATTTTTCAATTTCACGCATTTTACCAAATTTCAAGTTTCTAAAAGTGTTAGATTTGGAACACCGGGTTTTTATTGATTTTATTCCAACTGAGCTTGTTTACTTGAAGTATTTTTCTGCACACATTGAACAGAATTCAATTCCTTCAAGCATATCCAATCTTTGGAACCTTGAAACTCTTATATTAAAAAGTCCAATATATGCGTTACGTTGCACGCTACTACTACCTAGTACAGTTTGGGATATGGTTAAATTGAGACATCTGTATATTCCTGACTTCAGCACAAGGATTGAAGCAGCATTACTTGAGAACTCTGCAAAACTTTATAATTTGGAAACCCTTTCCACTCTATATTTCTCTCGTGTTGAGGATGCAGAATTGATGCTGAGAAAAACACCTAATCTTCGAAAACTGATATGTGAAGTTGAATGTTTAGAATACCCCCCTCAGTACCATGTGTTGAATTTTCCAATACGGCTTGAAATACTAAAGCTTTATCGATCAAAATTTAAAACCATCCCCTTTTGCATCTCTGCACCAAATCTCAAATACTTGAAACTCTGTGGCTTTTCCCTGGATTCTCAGTACTTATCAGAAACTGCTGATCATCTCAAGCACCTTGAGGTACTCATACTGTACAAGGTTGAATTTGGTGATCATAGGGAATGGAAAGTGAGCAATGGCAAGTTCCCTCAACTCAAAATCTTGAAACTAGAATATTTGTCCTTGGTGAAATGGATTGTAGCTGATGATGCCTTTCCTAACCTTGAACAATTGGTTTTGCGTGGATGTCAAGATCTTATGGAGATCCCTTCTTGTTTCATGGACATCCTTTCTCTCAAGTACATCGGGGTAGAATACTGCAATGAGTCGGTTGTCAAGTCAGCCTTGAATATACAAGAAACACAAGTCGAAGATTATCAAAATACTAATTTCAAGCTCGTTCTCATCGAGGTACACTACTGAAAAAAGCTTTATTCTGCATGATTTTGATGAATCAGAAATCGCCTAAATTTTACAAACTGTTTTCTCAGTTATCTTTACCTCGTGGCCTCGTTTTACATTTGGGTTCTTCTCTTAGTTTTCTTTGCAGAAAAAGGCGTGGAAATTAAATTTAACTGATGCGGAAGATATGCACAATGCAGTAAAAAATATTCTTGCAGAAATAAGATAGGTACTACTTTTTTTTTTTCTTTCCTTTTTTTAAATACACCAAATAGATAGATTCATCTTTTTTGTCTTTTCGATATGAAAGGGATAGAATCAGTTTCATCTGATGAGAAAGAGAAGAAACTTACTGTGACCGGAGATGTGGATGCTGATGAAGTTCAATTAGTTGTGGAGAAACTGAGAAAGCGTGGCATGCCAGGGTTGTAGTCCCAACTTGTCAACACAAATGTGCTATACTCATTTTGCTTACTGTAATACCATTTCATGACACACACACACAAACATTAACTGTAGTAAAGTTTTGATGGATCAGTAAATCTGAGTTCAACCCATTGTAATCCGTTCAAATTCAACTCAAAAAATTCCCATTGAGTTATTCTTTAACAGGGTATCCAGAGTTTGTAGCTGGAGCAATTTGGAATATCACATGTAATTTCTTTATGAGTTAATTCGTTTAATAAAAGATTCTGTAAAACGTCCAACGGCTGTTGCATTCATTGTAAACTAAATATATCTCAGTATGTAACTATTGAACAAATTTTTCATTTTAGTCCCTGAGGTTTGATGTAAGTCATTAGATTTTACGGATCCTGAAGTGAATGGTTTTAGCCTTTTCTATTTTCTTATGAGTTCACCAAAATGTTGTGATGCCACTCTGCTACATGTTAGAGAAATGAGAATGTTAGCACCCGAGAGTATGGCCTAGCGGTCAATCAATGAAGCAGGTGAAAACAACAAAAGCAAAAAATACTAAGAGATTTCTTCACATCTATCTAAGTACCGCTAAGCAAAGATACTGTAATGACCCTCCTGGTCATTTATGTGTCTTGCCTTCTGTGTGTCGTTTAGAGTGTTCCTATAGCGACCCCAAGTCATTTATGACTTGCTGGGACTAACGGTTCGGTCACATGGTCGTTCGTTTGGTTTTGGTGCGAGTTTTTGTGTTTTGGAGCTTATGAATCTTGAACGATGATTTTCGATCAAAAATTCAAGAAGATGACATCGGAATCCATTTCTAACGATTCCATCAGCTCCGGAAGGGTCATTTTAGGCTAGTAGCTTGGTCGGCATGACTCCCGGTGCGATTAGGCCTTTTAACTTTAAGTTTAAGCCTAAGTTTGACTTTGGTCAACATTCTGAGTAAACGCGCTCGGATGAGAATTCCGTCAGTGCGGTTAGCTCCGGAATGTCAAGTTTGGT

>Rpi-pta1

CCTGCAGGCTTGCTAATTGAGTGTCTGTTATAATCAGTATTAATTACTCTCAAGGTAATAGTATATTCCAAACAAATTTTGTGTTACCAAATTAAATATATTTCTAAAACTATCCTGAAAGTAGTTAATATACTTTTGAGTGTTGTATCATGTTTTTAATATAAAATATTAAAATTTAGATGAAATTTACTTTCTAGTTAAATTGGTCAAAGTTGAAAGAATTTCAAGTGAAAAAGTTTTTAATAATTTTGCTTTTATGCTATATTTTTTAAAGTTGAACGACTTTTTAATAAAAAAGAATAATAAAATTATATGATAATTTTTATAATACAATGGCCTTTATATGATGAAAAAAAAGAAAGAAATTAGATGACAACAATGTCCAAAAATAATCTTAAAGAATTATGATTTATATATAATAAAATTAAATTTAAAATTTGATGAAAAAATAGAGAAAAGAGGAAGATGATGAAGTGAAATGATTGGTGGTGGGTCCATGTGACATTAAAAAAAACAATTCTCTTAAATAATCCTTTCATACTAATGATAATTTTTTTTTTTTTTTTTTTTTTTTTAACAAATTGCGTATTGAAAAAAGGAAAATGGGGCGGTAATTACAAAGTAGGGAATCGAACTTTATCAAGAAGTTGAGAGTTCAAGTAACCAACCAACTAAACTACTAAAAATTTTCTAATTAATGATAATTGTAATTCATTTAGCATAAAAAATTTCATTGCACTTACTTTTAGAGTTTTGAAAACAATACTTCATCTATTCTATATTAATTAAATTTTCTATATTAATTAAATTTGTGAGGCAATACAAACTTATTAAGAAAAATATTTAAGGACATAATTTAAATCATATTTTTCACTATTGTTTTTTGTGAAATCATAAATATAACTTTATAAATAGTGCAATTTATCTCCTAGAAGCAAACTTCACTAAAGAAAAGGGCAAAGATGGAAAAGAAACTAAATATTCATCTTAAACTTTGAACAATTCAATTATTTTGAACAATGAAAAAAATCTCAAAAATTCAATTAATATGAATATTTTAGAGGCAAAAAATTAGTACTCCCTCCGTTCACTTTTATTTGTCATATTGCGCTTTTCGAAAGTCAATTTGACTAATTTTTAAAGATCAATTAGATTACACTAATTCAATATTTTAAATAGAAAAATTAGATATTCAAAAACTATACAAAAAATATTATACATTGCAATTTTTTGCATATCAATATGATAAAAAAATATATCGTAAAATATTAGTCAAAATTTTTATAATTTGACTCTAATAATGAAAAGTATAATAATTAATAGTGGACGGAGGAAGTATTGTCTTTCCAGATTTGTTGCCATTTTTGGGCCAAGGGCCATTAGCAGTTCTCTTCATTTTCTACTTCTGTCTCATATTAGCTGGGCATCTTACTAAAAATATTTGTCTCATATTACTTGATTATTTACTAAATCAAAATAGAATTAATTAATTTTTTCTCATTTTACCCCTCCAATTAATATAGTTTTGAAAGTTTTAAACAAATTTTGAAGAATCAAAATTTCTTTTTGCAAGAGACTTATTAATATAAACAAAGGATAAAATAATAAAATTTGTCAATTTATTGACGATCACTTAATAATCGTGTAAAATAGAAAATGTTTATCTAATATGAGACGGAGAAAATATATCCTAAAATATTTTTGGATGGATATGTGATATTCTAACCATTCACTAGACTATATTATGCATTTTAGCCGCCAATGACTTATTTCAGCTTTAATTAATTAGGAAAGAGGAAACTGCCAATGAGGAAGAGTAGGGGCGTAGTTGCTGTCGACGAAAAAAAGATAATACTCACTCTTTTCGATTTTTATTTTTATTTATCACTTTTAACCTATCATGTAAAAAGATAATTATTTTTTTCATGCTTTATCCTTAGTATTAAATAATTTAATAGGGATTATTTTGTAAAATATTTATATGAATAATTGTTTTCGTAATGAATTTGTCTAGTCAAACAATGATAAATAAAAATGAACGGAGAGAGTAGAAAACAAAACAAAAGAACAAGTTGCCAACTTGAGAGATTAAAAGGGACCAAAACGCCTTGGATTTTGAGATTCCATATGTGAAATTTCCATGAAATAATTGAATTTGTATTATTACAAATCAAACTTTCTATTTCATTCCAACTAGCCATCTTGGTTTCAAAATTACACATTCATTCATTCACAGATCTAATATTCTTAATAGTGATTTCCACATATGGCTGAAGCTTTCATTCAAGTTCTGTTAGACAATCTCACTTCTTTCCTCAAAGGGGAACTTACATTGCTTTTCGGTTTTCAAGATGAGTTCCAAAGGCTTTCAAGCATGTTTTCTACAATCCAAGCCGTCCTTGAAGATGCTCAGGAGAAGCAACTCAACAACAAGCCTCTAGAAAATTGGTTGCAAAAACTCAATGCTGCTACATACGAAGTCGATGACATCTTGGATGAATATAAAACCAAGGCCACAAGATTCTCCCAGTCTGAATATGGCCGTTATCATCCAAAGGTTATCCCTTTCCGTCACAAGGTCGGGAAAAGGATGGACCAAGTGATGAAAAAACTAAAGGCAATTGCTGAGGAAAGAAAGAATTTTCATTTGCACGAAAAAATTGTAGAGAGACAAGCTGTTAGACGGGAAACAGGTACTCATCTTAAATTAGTATTACAACAACTAAGTTTATATTCATTTTTTTGGCAATTATCAAATTCAGAAAAGGGTTAAATATACTCATGTCCTATCGTAAATAGTGTAAATATACCTCTCGTTGTACTTTCGATCTGAATATACTTGTCAAATCTGGCAAGCTCAGAATCAAATTATCCACCCCAACTTTTAAATACTCGACATCTTTAGAAATCCACCTGTCTAACTCATCCACTACCCATTCCCTTTGCTTTGAATTCTTTTCTTTACCTATAAACTTGGAACACTCGATCCGTTTTGCTTTTCTTAACAAAGCAGCTCAGAGAAAAGAGGTTTTCTTCTATTCTGTTTCTCTGTGTGCTGCACTTGGGTCCTTAATCCCATTAAAAACAGGGCATGTTAATCCCAACGACGGTAGCCTTTCCTGACAGCTGACTGTAAATTTTGTCTAACAAAGAAAAAAAAAGATTAGACATGTTTTTCCTTGTCATTGATTAGGCTGGATTTCTTTCAGAGTGGAACATAGGGGATATATTGGACCAAAAATAGAATGGGTATATATTTAAAGTATTTCTGATAGAACAGGAGTATATTGTGCGAAAATATCCTCTATTTTCTGTTGTCTCCTAATGAGTTTGAATGTAATAATATTCTCATGTGGACATTGCTTGCACCAGGTTCTGTATTAACCGAACCGCAGGTTTATGGAAGAGACAAAGAGAAAGATGAGATAGTGAAAATCCTAATAAACAATGTTAGTGATGCCCAACACCTTTCAGTCCTCCCAATACTTGGTATGGGGGGATTAGGAAAAACGACTCTTGCCCAAATGGTCTTCAATGACCAGAGAGTTACTGAGCATTTCCATTCCAAAATATGGATTTGTGTCTCGGAAGATTTTGATGAGAAGAGGTTAATAAAGGCAATTGTAGAATCTATTGAAGGAAGGCCACTACTTGGTGAGATGGACTTGGCTCCACTTCAAAAGAAGCTTCAGGAGTTGCTGAATGGAAAAAGATACTTGCTTGTCTTAGATGATGTTTGGAATGAAGATCAACAGAAGTGGGCTAATTTAAGAGCAGTCTTGAAGGTTGGAGCAAGTGGTGCTTCTGTTCTAACCACTACTCGTCTTGAAAAGGTTGGATCAATTATGGGAACATTGCAACCATATGAACTGTCAAATCTGTCTCAAGAAGATTGTTGGTTGTTGTTCATGCAACGTGCATTTGGACACCAAGAAGAAATAAATCCAAACCTTGTGGCAATCGGAAAGGAGATTGTGAAAAAAAGTGGTGGTGTGCCTCTAGCAGCCAAAACTCTTGGAGGTATTTTGTGCTTCAAGAGAGAAGAAAGAGCATGGGAACATGTGAGAGACAGTCCGATTTGGAATTTGCCTCAAGATGAAAGTTCTATTCTGCCTGCCCTGAGGCTTAGTTACCATCAACTTCCACTTGATTTGAAACAATGCTTTGCGTATTGTGCGGTGTTCCCAAAGGATGCCAAAATGGAAAAAGAAAAGCTAATCTCTCTCTGGATGGCGCATGGTTTTCTTTTATCAAAAGGAAACATGGAGCTAGAGGATGTGGGCGATGAAGTATGGAAAGAATTATACTTGAGGTCTTTTTTCCAAGAGATTGAAGTTAAAGATGGTAAAACTTATTTCAAGATGCATGATCTCATCCATGATTTGGCAACATCTCTGTTTTCAGCAAACACATCAAGCAGCAATATCCGTGAAATAAATAAACACAGTTACACACATATGATGTCCATTGGTTTCGCCGAAGTGGTGTTTTTTTACACTCTTCCCCCCTTGGAAAAGTTTATCTCGTTAAGAGTGCTTAATCTAGGTGATTCGACATTTAATAAGTTACCATCTTCCATTGGAGATCTAGTACATTTAAGATACTTGAACCTGTATGGCAGTGGCATGCGTAGTCTTCCAAAGCAGTTATGCAAGCTTCAAAATCTGCAAACTCTTGATCTACAATATTGCACCAAGCTTTGTTGTTTGCCAAAAGAAACAAGTAAACTTGGTAGTCTCCGAAATCTTTTACTTGATGGTAGCCAGTCATTGACTTGTATGCCACCAAGGATAGGATCATTGACATGCCTTAAGACTCTAGGTCAATTTGTTGTTGGAAGGAAGAAAGGTTATCAACTTGGTGAACTAGGAAACCTAAATCTCTATGGCTCAATTAAAATCTCGCATCTTGAGAGAGTGAAGAATGATAGGGACGCAAAAGAAGCCAATTTATCTGCAAAAGGGAATCTGCATTCTTTAAGCATGAGTTGGAATAACTTTGGACCACATATATATGAATCAGAAGAAGTTAAAGTGCTTGAAGCCCTCAAACCACACTCCAATCTGACTTCTTTAAAAATCTATGGCTTCAGAGGAATCCATCTCCCAGAGTGGATGAATCACTCAGTATTGAAAAATATTGTCTCTATTCTAATTAGCAACTTCAGAAACTGCTCATGCTTACCACCCTTTGGTGATCTGCCTTGTCTAGAAAGTCTAGAGTTACACTGGGGGTCTGCGGATGTGGAGTATGTTGAAGAAGTGGATATTGATGTTCATTCTGGATTCCCCACAAGAATAAGGTTTCCATCCTTGAGGAAACTTGATATATGGGACTTTGGTAGTCTGAAAGGATTGCTGAAAAAGGAAGGAGAAGAGCAATTCCCTGTGCTTGAAGAGCTGATAATTCACGAGTGCCCTTTTCTGACCCTTTCTTCTAATCTTAGGGCTCTTACTTCCCTCAGAATTTGCTATAATAAAGTAGCTACTTCATTCCCAGAAGAGATGTTCAAAAACCTTGCAAATCTCAAATACTTGACAATCTCTCGGTGCAATAATCTCAAAGAGCTGCCTACCAGCTTGGCTAGTCTGAATGCTTTGAAAAGTCTAAAAATTCAATTGTGTTGCGCACTAGAGAGTCTCCCTGAGGAAGGGCTGGAAGGTTTATCTTCACTCACAGAGTTATTTGTTGAACACTGTAACATGCTAAAATGTTTACCAGAGGGATTGCAGCACCTAACAACCCTCACAAGTTTAAAAATTCGGGGATGTCCACAACTGATCAAGCGGTGTGAGAAGGGAATAGGAGAAGACTGGCACAAAATTTCTCACATTCCTAATGTGAATATATATAATTAAGTTATTTGCTATTGTTTCTTTGTTTGTGAGTCTTTTTGGTTCCTGCCATTGTGATTGCATGTAATTTTTTTCTAGGGTTGTTTGTTTGTGAGTCTCTCTCTCATTGGATGTAATTTTCTTTTGGTAACAAATTAACAATCTATTTGTATTATACGCTTTCAGAATCTATTACTTATTTGTAATTGTTTCTTTGTTTGTAAATTGTGAGTATCTTATTGTATGGAATTTTCTGATTTTATTTTGAAAACAAATCAATAAGATCCATCTGTATTATACTCCCTTCGTCTCATTTTATGTGACACTTTTTGGATTTCGAGATTCAAACAAATCTATTTTTGATCTTAAATTTTTCATAGATCTTTTAAACATTTTGAATTATCAATTATTGTGATTTTAGTACTTTTTATGTAGTTTACAAATATATAAAATTTATTTTTTTTAAAAAAAGAAGATTTCATGCGCATATTCCCGATCAAACTTAAATTACTAGACTCTCGAAAAATGAAAAGTGTCACATAAATTGAGACAGAGGGAGTACTTGTTAATGTTGTAATTATTGGCGAACAATAATGTTGGTGATTATCACTTTTTGAATAAATGTTGTGTCACGTGGAAAAAACACCAAATAGAAGTATTCATGCTTTTTTAGTATATATAAACACGATTTTTAACTTGGTTTCAGCGGATAGTCATGACCTTTTACTTTGAATGTGCACAAGTAGATACTTGTATAAAATTAAATAAATTTTATAAAATTATACAATATGACACTGAGAGTAATTGATACCAATTGCAGTCGTTGCTGCTTTTCGATTCTCTGTCATTCTCTAGGCGCGCC

> *Rpi-pta1^A2012^*

CCTGCAGGCTTGCTAATTGAGTGTCTGTTATAATCAGTATTAATTACTCTCAAGGTAATAGTATATTCCAAACAAATTTTGTGTTACCAAATTAAATATATTTCTAAAACTATCCTGAAAGTAGTTAATATACTTTTGAGTGTTGTATCATGTTTTTAATATAAAATATTAAAATTTAGATGAAATTTACTTTCTAGTTAAATTGGTCAAAGTTGAAAGAATTTCAAGTGAAAAAGTTTTTAATAATTTTGCTTTTATGCTATATTTTTTAAAGTTGAACGACTTTTTAATAAAAAAGAATAATAAAATTATATGATAATTTTTATAATACAATGGCCTTTATATGATGAAAAAAAAGAAAGAAATTAGATGACAACAATGTCCAAAAATAATCTTAAAGAATTATGATTTATATATAATAAAATTAAATTTAAAATTTGATGAAAAAATAGAGAAAAGAGGAAGATGATGAAGTGAAATGATTGGTGGTGGGTCCATGTGACATTAAAAAAAACAATTCTCTTAAATAATCCTTTCATACTAATGATAATTTTTTTTTTTTTTTTTTTTTTTTTAACAAATTGCGTATTGAAAAAAGGAAAATGGGGCGGTAATTACAAAGTAGGGAATCGAACTTTATCAAGAAGTTGAGAGTTCAAGTAACCAACCAACTAAACTACTAAAAATTTTCTAATTAATGATAATTGTAATTCATTTAGCATAAAAAATTTCATTGCACTTACTTTTAGAGTTTTGAAAACAATACTTCATCTATTCTATATTAATTAAATTTTCTATATTAATTAAATTTGTGAGGCAATACAAACTTATTAAGAAAAATATTTAAGGACATAATTTAAATCATATTTTTCACTATTGTTTTTTGTGAAATCATAAATATAACTTTATAAATAGTGCAATTTATCTCCTAGAAGCAAACTTCACTAAAGAAAAGGGCAAAGATGGAAAAGAAACTAAATATTCATCTTAAACTTTGAACAATTCAATTATTTTGAACAATGAAAAAAATCTCAAAAATTCAATTAATATGAATATTTTAGAGGCAAAAAATTAGTACTCCCTCCGTTCACTTTTATTTGTCATATTGCGCTTTTCGAAAGTCAATTTGACTAATTTTTAAAGATCAATTAGATTACACTAATTCAATATTTTAAATAGAAAAATTAGATATTCAAAAACTATACAAAAAATATTATACATTGCAATTTTTTGCATATCAATATGATAAAAAAATATATCGTAAAATATTAGTCAAAATTTTTATAATTTGACTCTAATAATGAAAAGTATAATAATTAATAGTGGACGGAGGAAGTATTGTCTTTCCAGATTTGTTGCCATTTTTGGGCCAAGGGCCATTAGCAGTTCTCTTCATTTTCTACTTCTGTCTCATATTAGCTGGGCATCTTACTAAAAATATTTGTCTCATATTACTTGATTATTTACTAAATCAAAATAGAATTAATTAATTTTTTCTCATTTTACCCCTCCAATTAATATAGTTTTGAAAGTTTTAAACAAATTTTGAAGAATCAAAATTTCTTTTTGCAAGAGACTTATTAATATAAACAAAGGATAAAATAATAAAATTTGTCAATTTATTGACGATCACTTAATAATCGTGTAAAATAGAAAATGTTTATCTAATATGAGACGGAGAAAATATATCCTAAAATATTTTTGGATGGATATGTGATATTCTAACCATTCACTAGACTATATTATGCATTTTAGCCGCCAATGACTTATTTCAGCTTTAATTAATTAGGAAAGAGGAAACTGCCAATGAGGAAGAGTAGGGGCGTAGTTGCTGTCGACGAAAAAAAGATAATACTCACTCTTTTCGATTTTTATTTTTATTTATCACTTTTAACCTATCATGTAAAAAGATAATTATTTTTTTCATGCTTTATCCTTAGTATTAAATAATTTAATAGGGATTATTTTGTAAAATATTTATATGAATAATTGTTTTCGTAATGAATTTGTCTAGTCAAACAATGATAAATAAAAATGAACGGAGAGAGTAGAAAACAAAACAAAAGAACAAGTTGCCAACTTGAGAGATTAAAAGGGACCAAAACGCCTTGGATTTTGAGATTCCATATGTGAAATTTCCATGAAATAATTGAATTTGTATTATTACAAATCAAACTTTCTATTTCATTCCAACTAGCCATCTTGGTTTCAAAATTACACATTCATTCATTCACAGATCTAATATTCTTAATAGTGATTTCCACATATGGCTGAAGCTTTCATTCAAGTTCTGTTAGACAATCTCACTTCTTTCCTCAAAGGGGAACTTACATTGCTTTTCGGTTTTCAAGATGAGTTCCAAAGGCTTTCAAGCATGTTTTCTACAATCCAAGCCGTCCTTGAAGATGCTCAGGAGAAGCAACTCAACAACAAGCCTCTAGAAAATTGGTTGCAAAAACTCAATGCTGCTACATACGAAGTCGATGACATCTTGGATGAATATAAAACCAAGGCCACAAGATTCTCCCAGTCTGAATATGGCCGTTATCATCCAAAGGTTATCCCTTTCCGTCACAAGGTCGGGAAAAGGATGGACCAAGTGATGAAAAAACTAAAGGCAATTGCTGAGGAAAGAAAGAATTTTCATTTGCACGAAAAAATTGTAGAGAGACAAGCTGTTAGACGGGAAACAGGTACTCATCTTAAATTAGTATTACAACAACTAAGTTTATATTCATTTTTTTGGCAATTATCAAATTCAGAAAAGGGTTAAATATACTCATGTCCTATCGTAAATAGTGTAAATATACCTCTCGTTGTACTTTCGATCTGAATATACTTGTCAAATCTGGCAAGCTCAGAATCAAATTATCCACCCCAACTTTTAAATACTCGACATCTTTAGAAATCCACCTGTCTAACTCATCCACTACCCATTCCCTTTGCTTTGAATTCTTTTCTTTACCTATAAACTTGGAACACTCGATCCGTTTTGCTTTTCTTAACAAAGCAGCTCAGAGAAAAGAGGTTTTCTTCTATTCTGTTTCTCTGTGTGCTGCACTTGGGTCCTTAATCCCATTAAAAACAGGGCATGTTAATCCCAACGACGGTAGCCTTTCCTGACAGCTGACTGTAAATTTTGTCTAACAAAGAAAAAAAAAGATTAGACATGTTTTTCCTTGTCATTGATTAGGCTGGATTTCTTTCAGAGTGGAACATAGGGGATATATTGGACCAAAAATAGAATGGGTATATATTTAAAGTATTTCTGATAGAACAGGAGTATATTGTGCGAAAATATCCTCTATTTTCTGTTGTCTCCTAATGAGTTTGAATGTAATAATATTCTCATGTGGACATTGCTTGCACCAGGTTCTGTATTAACCGAACCGCAGGTTTATGGAAGAGACAAAGAGAAAGATGAGATAGTGAAAATCCTAATAAACAATGTTAGTGATGCCCAACACCTTTCAGTCCTCCCAATACTTGGTATGGGGGGATTAGGAAAAACGACTCTTGCCCAAATGGTCTTCAATGACCAGAGAGTTACTGAGCATTTCCATTCCAAAATATGGATTTGTGTCTCGGAAGATTTTGATGAGAAGAGGTTAATAAAGGCAATTGTAGAATCTATTGAAGGAAGGCCACTACTTGGTGAGATGGACTTGGCTCCACTTCAAAAGAAGCTTCAGGAGTTGCTGAATGGAAAAAGATACTTGCTTGTCTTAGATGATGTTTGGAATGAAGATCAACAGAAGTGGGCTAATTTAAGAGCAGTCTTGAAGGTTGGAGCAAGTGGTGCTTCTGTTCTAACCACTACTCGTCTTGAAAAGGTTGGATCAATTATGGGAACATTGCAACCATATGAACTGTCAAATCTGTCTCAAGAAGATTGTTGGTTGTTGTTCATGCAACGTGCATTTGGACACCAAGAAGAAATAAATCCAAACCTTGTGGCAATCGGAAAGGAGATTGTGAAAAAAAGTGGTGGTGTGCCTCTAGCAGCCAAAACTCTTGGAGGTATTTTGTGCTTCAAGAGAGAAGAAAGAGCATGGGAACATGTGAGAGACAGTCCGATTTGGAATTTGCCTCAAGATGAAAGTTCTATTCTGCCTGCCCTGAGGCTTAGTTACCATCAACTTCCACTTGATTTGAAACAATGCTTTGCGTATTGTGCGGTGTTCCCAAAGGATGCCAAAATGGAAAAAGAAAAGCTAATCTCTCTCTGGATGGCGCATGGTTTTCTTTTATCAAAAGGAAACATGGAGCTAGAGGATGTAGGCGATGAAGTATGGAAAGAATTATACTTGAGGTCTTTTTTCCAAGAGATTGAAGTTAAAGATGGTAAAACTTATTTCAAGATGCATGATCTCATCCATGATTTGGCAACATCTCTGTTTTCAGCAAACACATCAAGCAGCAATATCCGTGAAATAAATAAACACAGTTACACACATATGATGTCCATTGGTTTCGCCGAAGTGGTGTTTTTTTACACTCTTCCCCCCTTGGAAAAGTTTATCTCGTTAAGAGTGCTTAATCTAGGTGATTCGACATTTAATAAGTTACCATCTTCCATTGGAGATCTAGTACATTTAAGATACTTGAACCTGTATGGCAGTGGCATGCGTAGTCTTCCAAAGCAGTTATGCAAGCTTCAAAATCTGCAAACTCTTGATCTACAATATTGCACCAAGCTTTGTTGTTTGCCAAAAGAAACAAGTAAACTTGGTAGTCTCCGAAATCTTTTACTTGATGGTAGCCAGTCATTGACTTGTATGCCACCAAGGATAGGATCATTGACATGCCTTAAGACTCTAGGTCAATTTGTTGTTGGAAGGAAGAAAGGTTATCAACTTGGTGAACTAGGAAACCTAAATCTCTATGGCTCAATTAAAATCTCGCATCTTGAGAGAGTGAAGAATGATAGGGACGCAAAAGAAGCCAATTTATCTGCAAAAGGGAATCTGCATTCTTTAAGCATGAGTTGGAATAACTTTGGACCACATATATATGAATCAGAAGAAGTTAAAGTGCTTGAAGCCCTCAAACCACACTCCAATCTGACTTCTTTAAAAATCTATGGCTTCAGAGGAATCCATCTCCCAGAGTGGATGAATCACTCAGTATTGAAAAATATTGTCTCTATTCTAATTAGCAACTTCAGAAACTGCTCATGCTTACCACCCTTTGGTGATCTGCCTTGTCTAGAAAGTCTAGAGTTACACTGGGGGTCTGCGGATGTGGAGTATGTTGAAGAAGTGGATATTGATGTTCATTCTGGATTCCCCACAAGAATAAGGTTTCCATCCTTGAGGAAACTTGATATATGGGACTTTGGTAGTCTGAAAGGATTGCTGAAAAAGGAAGGAGAAGAGCAATTCCCTGTGCTTGAAGAGCTGATAATTCACGAGTGCCCTTTTCTGACCCTTTCTTCTAATCTTAGGGCTCTTACTTCCCTCAGAATTTGCTATAATAAAGTAGCTACTTCATTCCCAGAAGAGATGTTCAAAAACCTTGCAAATCTCAAATACTTGACAATCTCTCGGTGCAATAATCTCAAAGAGCTGCCTACCAGCTTGGCTAGTCTGAATGCTTTGAAAAGTCTAAAAATTCAATTGTGTTGCGCACTAGAGAGTCTCCCTGAGGAAGGGCTGGAAGGTTTATCTTCACTCACAGAGTTATTTGTTGAACACTGTAACATGCTAAAATGTTTACCAGAGGGATTGCAGCACCTAACAACCCTCACAAGTTTAAAAATTCGGGGATGTCCACAACTGATCAAGCGGTGTGAGAAGGGAATAGGAGAAGACTGGCACAAAATTTCTCACATTCCTAATGTGAATATATATAATTAAGTTATTTGCTATTGTTTCTTTGTTTGTGAGTCTTTTTGGTTCCTGCCATTGTGATTGCATGTAATTTTTTTCTAGGGTTGTTTGTTTGTGAGTCTCTCTCTCATTGGATGTAATTTTCTTTTGGTAACAAATTAACAATCTATTTGTATTATACGCTTTCAGAATCTATTACTTATTTGTAATTGTTTCTTTGTTTGTAAATTGTGAGTATCTTATTGTATGGAATTTTCTGATTTTATTTTGAAAACAAATCAATAAGATCCATCTGTATTATACTCCCTTCGTCTCATTTTATGTGACACTTTTTGGATTTCGAGATTCAAACAAATCTATTTTTGATCTTAAATTTTTCATAGATCTTTTAAACATTTTGAATTATCAATTATTGTGATTTTAGTACTTTTTATGTAGTTTACAAATATATAAAATTTATTTTTTTTAAAAAAAGAAGATTTCATGCGCATATTCCCGATCAAACTTAAATTACTAGACTCTCGAAAAATGAAAAGTGTCACATAAATTGAGACAGAGGGAGTACTTGTTAATGTTGTAATTATTGGCGAACAATAATGTTGGTGATTATCACTTTTTGAATAAATGTTGTGTCACGTGGAAAAAACACCAAATAGAAGTATTCATGCTTTTTTAGTATATATAAACACGATTTTTAACTTGGTTTCAGCGGATAGTCATGACCTTTTACTTTGAATGTGCACAAGTAGATACTTGTATAAAATTAAATAAATTTTATAAAATTATACAATATGACACTGAGAGTAATTGATACCAATTGCAGTCGTTGCTGCTTTTCGATTCTCTGTCATTCTCTAGGCGCGCC

>Rpi-Mcq1.1

TTAAATTGAATGACAGTTAAATTGGAATGAATGGTGTAATTTCCTTTGACTATTGTACTAGTATCTTATCCACAGCATGTGTTGTTCCTTCCTTCTTTCGTTTTTCATTTACTTGACATTAGTAGGAGACTTGGCAGTGGACTCCAACTATTCTAAGCTGACCTTTCTTTTCCTTTACCAATTATCTTCTCTTTTCTAATTTCTCATTCTGATCGGTTTTTGTAGCTACTGAAAAAGAAAGAGTGAAGAAATGGCTGAAATTCTTCTTACAGCAGTCATCAATAAATCTGTAGAAATAGCTGGAAATGTACTCTTTCAAGAAGGTACGCGTTTATATTGGTTGAAGGAGGATATAGATTGGCTCCAAAGAGAAATGAGACACATTCGATCATATGTAGACAATGCAAAGGCCAAGGAAGTTGGAGGTGATTCAAGGGTGAAAAACTTATTAAAAGATATTCAACAACTCGCAGGTGATGTGGAGGATCTCCTAGATGAGTTTCTTCCAAAAATTCAACAATCCAGTAAGTTCAAAGGCGCAATTTGTTGCCTTAAGACCGTTTCTTTTGCGGATGAGTTTGCTATGGAGATTGAGAAGATAAAAAGAAGGGTTGTGGACATTGATCGTGTAAGGACAACTTACAACATCATGGATACAAATAACAACAATGATTGCATTCCATTGGACCAGAGAAGATTGTTCCTTCATGTTGATGAAACAGAGGTCATCGGTTTGGATGATGACTTCAATACACTACAAGCCAAATTACTTGACCAAGATTTGCCTTATGGAGTTGTTTCAATAGTTGGCATGCCCGGTCTAGGAAAAACAACTCTTGCCAAGAAACTTTATAGGCATGTCCGTCATAAATTTGAGTGTTCGGGACTGGTCTATGTTTCACAACAGCCAAGGGCGGGAGAAATCTTAATCGACATAGCCAAACAAGTTGGACTGACGGAAGACGAAAGGAAAGAAAACTTGGAGAACAACCTACGGTCACTCTTGAAAAGAAAAAGGTATGTTATTCTCTTAGATGACATTTGGGATGTTGAAATTTGGGATGATCTAAAACTTGTCCTTCCTGAATGTGATTCAAAAATTGGCAGTAGGATAATTATAACCTCTCGAAATAGTAATGTAGGCAGATACATAGGAGGGGATTTCTCAATTCACGTGTTGCAACCTCTAAATTCGGAGAACAGTTTTGAACTCTTTACCAAGAAAATCTTTATTTTTGATAACAATAATAATTGGACCAATGCTTCACCAAACTTGGTAGATATTGGTAGAAGTATAGTTGGTAGATGTGGTGGTATACCACTAGCCATTGTGGTGACTGCAGGCATGTTAAGGGCAAGAGAAAGAACAGAACGTGCATGGAACAGGTTACTTGAGAGTATGAGCCATAAAGTTCAAGATGGATGTGCTAAGGTATTGGCTCTGAGTTACAATGATTTGCCAATTGCATTAAGGCCATGTTTCTTGTATTTTGGCCTTTACCCCGAGGATCATGAAATTCGTGCTTTTGATTTGACAAATATGTGGATTGCTGAGAAGTTGATAGTTGTAAATAGTGGCAATGGGCGAGAGGCTGAAAGTTTGGCGGATGATGTCCTAAATGATTTGGTTTCAAGAAACATGATTCAAGTTGCCAAAAGGACATATGATGGAAGAATTTCAAGTTGTCGCATACATGACTTGTTACATAGTTTGTGTGTTGACTTGGCTAAGGAAAGCAACTTCTTTCACACCGAGCACAATGCATTGGGTGATCCCGGAAATGTTGCTAGGCTGCGAAGGATTACATTCTACTCTGATAATAATGCCATGAATGAGTTCTTCCGTTCAAATCCTAAGCTTGAGAAGCTTCGTGCACTTTTCTGTTTTACAGAAGACCCTTGCATATTTTCTCAACTGGCTCATCTTGATTTCAAATTATTGCAAGTGTTGGTTGTAGTCATCTTTGTTGATGATATTTGTGGTGTCAGTATCCCAAACACATTTGGGAACATGAGGTGCTTACGTTATCTGCGATTCCAGGGGCATTTTTATGGGAAACTGCCAAATTGTATGGTGAAGCTCAAACGTCTAGAGACCCTCGATATTGGTTATAGCTTAATTAAATTTCCTACTGGTGTTTGGAAGTCTACACAATTGAAACATCTTCGTTATGGAGGTTTTAATCAAGCATCTAACAGTTGCTTTTCTATAAGCCCATTTTTCCCAAACTTGTACTCATTGCCTCATAATAATGTACAAACTTTGATGTGGCTGGATGATAAATTTTTTGAGGCGGGATTGTTGCACCGATTGATCAATTTAAGAAAACTGGGTATAGCAGGAGTATCTGATTCTACAGTTAAGATATTATCAGCATTGAGCCCTGTGCCAACGGCGCTGGAGGTTCTGAAGCTCAAAATTTACAGGGACATGAGTGAGCAAATAAACTTGTCGTCCTATCCAAATATTGTTAAGTTGCGTTTGAATGTTTGCGGAAGAATGCGCTTGAACTGTGAAGCATTTCCTCCAAATCTTGTCAAGCTTACTCTTGTCGGCGATGAGGTAGACGGTCATGTAGTGGCAGAGCTTAAGAAATTGCCCAAATTAAGGATACTTAAAATGTTTGGGTGCAGTCATAATGAAGAAAAGATGGATCTCTCTGGTGATGGTGATAGCTTTCCGCAACTTGAAGTTCTGCATATTGATGAACCAGATGGGTTGTCTGAAGTAACGTGTAGGGATGATGTCAGTATGCCTAAATTGAAAAAGTTGTTACTTGTACAACGCCGCCCTTCTCCAATTAGTCTCTCAGAACGTCTTGCAAAGCTCAGAATATGAAATTCACAATGTGTCAATATATAGGTTAGTTTGCTACGTTAATCTCCCATTATGTCTAATGAATTGCGCGCAGATGCATTTGAGAATGATTGATTGTAAATTGTAATTGTAATAAATAAATAAATGTTTGATTGCTTTCTGAAGTTGATGTATTTGTGGCTTGTGATTTGTAAAACATATTTATTTATTGTCTTATCACTTATGTTTATTTACCTTTGGAATTAGCAGTAGCTTTCGTTTCTTCTCTTCT

>Rpi-blb3

TAAGACTTTTCTCTATATGTGTTTTTCCCCAAGTTGTATAATGGTTGTTGAAGATGCTTTAATTAAAAAAAAAAACCTTTTGTTTAGTGGAAAATTTCAAAAAGCTTTAGTACATCTTTGTCGTTTTATCCAATCGTAATTCTTTATTCAGAAACCACATGTTTTTTTTCTAATCTTACTTTTATGTCTATCACCCATTTTCCAATATACAGCCTACTCTTTTTTTCAATCAAAACTAGTATTCCTAAAGATGGCTGATGCCTTTCTATCATTTGCAGTTCAAAAATTGGGTGATTTCCTAATACAGAAAGTTTCCCTGCGTAAAAGTCTCAGAGATGAAATTAGATGGCTGATAAATGAGCTACTCTTCATACGGTCTTTCCTCAGAGATGCAGAACAAAAGCAGTGCGGAGATCAAAGAGTTCAACAATGGGTGTTTGAGATCAACTCTATTGCTAATGATGCTGTTGCTATACTCGAGACTTATAGCTTTGAGGCTGGTAAAGGTGCTAGTCGTCTCAAGGCTTGCACTTGCATATGTAGGAAGGAGAAGAAATTCTACAATGTTGCCGAGGAGATTCAATCACTCAAGCAACGAATCATGGATATCTCTCGCAAACGAGAGACTTATGGTATTACAAATATCAATTATAATTCAGGAGAAAGGCCAAGTAATCAGGTTACAACATTGAGGAGAACTACCTCATATGTAGATGAACAGGATTACATTTTTGTTGGCTTTCAGGATGTTGTACAAACATTGCTAGCTCAACTTCTGAAAGCAGAGCCTCGTCGAAGCGTCCTCTCCATTTATGGAATGGGGGGTTTAGGCAAGACCACTCTTGCCAGAAAACTTTACACCAGTCCTGATATACTCAATAGCTTTCCTACACGCGCTTGGATATGTGTCTCTCAAGAGTACAACACAATGGATCTTCTTAGGACTATCATAAAATCCATCCAAGGCTGCGCCAAGGAAACTCTAGATTTGTTGGAAAAGATGGCAGAAATAGATCTAGAAAATCACCTTCGTGATCTATTGAAAGAATGCAAATACCTTGTGGTGGTTGATGATGTATGGCAGAGAGAAGCATGGGAGAGTTTGAAAAGAGCATTCCCGGATGGCAAGAATGGAAGCAGAGTCATTATTACCACGCGCAAAGAGGATGTCGCTGAAAGAGTAGACCACAGAGGTTTTGTTCATAAACTTCGTTTCCTAAGTCAAGAAGAAAGTTGGGATCTCTTTCGTAGGAAACTACTTGATGTTCGAGCAATGGTTCCAGAAATGGAAAGTTTAGCTAAGGATATGGTGGAAAAGTGTAGAGGCTTACCTCTTGCAATTGTTGTATTGAGCGGACTACTTTCGCATAAAAAGGGGCTAAACCAATGGCAAAAGGTGAAAGATCACCTTTGGAAGAACATTAAAGAAGATAAATCTATTGAAATCTCTAACATACTATCCTTAAGCTACAATGATTTGTCAACTGCGCTCAAGCAGTGTTTTCTCTACTTTGGTATTTTTCCAGAAGATCAAGTGGTAAAGGCTGATGACATAATACGGTTGTGGATGGCGGAGGGTTTCATACCCAGAGGAGAAGAAAGAATGGAGGATGTGGCTGACGGCTTCTTGAATGAACTGATAAGACGAAGCTTGGTTCAAGTAGCTAAAACATTTTGGGAAAAAGTTACTGACTGTAGGGTTCATGATTTACTTCGTGATCTTGCGATACAAAAGGCATTGGAGGTAAACTTCTTTGACGTTTATGGTCCAAGAAGCCACTCCATATCCTCTTTATGTATCAGACATGGCATTCATAGTGAAGGAGAAAGGTACCTCTCATCACTTGATCTTTCTAACTTGAAGTTGAGGTCAATTATGTTCTTCGATCCAGATTTTCGTAAGATGAGTCATATAAACCTCAGGAGTGAGTTCCAACATCTGTATGTGTTGTACTTGGATACGAATTTTGGGTATGTGTCTATGGTACCTGATGCCATAGGAAGTTTGTACCACCTCAAGTTGTTAAGATTGAGAGGTATCCATGATATTCCGTCTTCCATTGGCAACCTCAAGAATTTACAAACACTTGTCGTTGTAAATGGTTACACATTTTTTTGCCAACTACCCTGCAAGACAGCTGACCTAATAAATCTAAGACATTTAGTTGTTCAATATTCAGAGCCTTTAAAATGTATAAACAAACTCACTAGTCTTCAAGTTCTTGATGGTGTTGCTTGTGATCAGTGGAAAGATGTTGACCCTGTTGATTTAGTCAATCTTCGAGAATTAAGCATGGATCGTATCAGGAGCTCTTACTCCCTAAACAACATTAGCAGCTTGAAAAACCTTAGCACTCTCAAATTGATTTGTGGAGAACGTCAATCATTTGCATCCCTTGAATTTGTTAATTGTTGTGAAAAGCTCCAGAAATTGTGGTTACAAGGGAGAATAGAGGAACTGCCTCATCTGTTTTCAAACTCCATCACAATGATGGTTCTGAGTTTCTCAGAACTGACAGAAGATCCGATGCCTATTTTGGGAAGGTTTCCAAACCTAAGGAATCTCAAATTAGATGGAGCTTATGAAGGAAAAGAAATAATGTGCAGTGATAACAGCTTCAGTCAACTAGAGTTCCTTCATCTTCGTGATCTTTGGAAGCTAGAAAGATGGGATTTAGGCACAAGTGCCATGCCTCTGATTAAAGGTCTTGGTATCCATAACTGTCCAAATTTAAAGGAGATTCCTGAGAGAATGAAAGACATGGAGCTGTTGAAGCGGAATTATATGTTGTGAAGCTTTTCTGCCAAGCACATTGGTTATTAATTGAGTGGTTTTAGTGTTGATTTCTTATTATTGTTTTAAGCTTTTTGAGTGTGTAATTGGTTTGAACATTATTGTTTTAATTAATTGGTCTACTGTATGTTCTCATGCTTATCCACATTTAAGACAATGCTTTATATGTTAAAATGAAATTAAAAATACTAGTATATGGTACTCTCTCTTGTCCACAATTTCGTATATTTTTTGTTCCTCTTCATAAAAA

>Rpi-blb2

GATCTAGAATCACCGAACCTCCCCTCGGTACAGCTCCTCCAGTTCTACCATGAATTTCATCCACTGATTCCTCTTCAATCGCCATTGCAGATTCTCTCGATCTATGCTCAAAAAATCCCGAGATAAAACCCTAGATCTGCTTCAAATGCTCTGATACCATGTAATTTCAGTGAATTCTAACTAAACAATGGAGAGAATTAACTATTTTAGAAAGACTGATTGAAGGAGAAGAAGAGAGAAAAATTCTATATTGAACTCATGAACCAAAATGAATGAAAAAAATAATGAGAAGAACTATACTATTACAATCTATATATCTCTATTTATATTCTAATCTGAAGCAGTTAATTTAACTGACTCTAACAACTAGACTGATAGGTGTACATTTTCTGTTAGTGCACTGCAGTGCATTTAACTAACTGCTTAACATAAAGAATGTTGTTCGAACTTCATTCGAATAGCTTCAATGAGAAGCAAACATGTGTACCTGTAAAGACACACAGTAAAAGTGTTAATAATGAATAAATATGAATAAATCAAATAATAAATTAAAAATAAAAACACATCCAATTAACATTGGAGGTCTTGAAAATCGATGGTAATTAACAAAGACCCTTGTGAAATTTAAGTCTGTAATTGAAAATTTGAGTATAGGTTAGGGGACATTTGACTATTTTCTCATTTTCTTTATCTTTTTCCTAATTTGTGGCAGACAAGTGAGGAGGCCCCACTGTAATTGATTCATGCTTTTGCTTTCTTGACTTTTTGGAACAATACTATGCATCATATTTGGTCTTAATTATTCCTCTGTTTATTTCCAGAATTTTGAGCTCTATACATCTAATAACAAAGCAAGCAGAGGATATATAGTTTCATCAACTAAAAAGGTTAGTCAACTCATCTAATATTTGCTACTCTCATCTCTATTGAAGTACAGTTATGGAAAAGTAGAAGTGATGTAAGAAAAATGAAAGAACTTTAGTAGGTTAGTTGGATCTAACAAAGAGAAAGGGAAATAAATTGCAGGAGAAAGAGAGAGGTTAAATACTTACTCACACCACCGATTTACAACAAATCACTTAATTGTGGTTAGTTAATGTATACTTTCACCTCATTAAATTATTACTTACCCATGATAAGTTGTATTAATTTGGTATTAATATCCGGTGCGGGTGAATTCTTACCGGGTGAGAGGGATGGGGTTGGAGAGTGTGGAGTGAACAGAAGCAGATGTTTTAGATTTTTTCTAAGATGACGAAAGATTCCCCTCACTAATGAAAATATATTACTATACGCTATTAGAGATAGAAAGGTTCGGTACCAGTTGGTCTCGTTTCTGGATGAACCCCATTTTTACAAGTCATTTTCTTCAATTCAAATCGCAAGTGTACCTTTATCATCTTCCACTAATTAAGTCCTCTTAAGTTCGCGTGAAAATAGTGAAATTATTGATTATTCTTATCATTTCATCTTCTTTCTCCTGATAAAGTTTTATGTACTTTTTATGCATCAGGTCTTGAGAACTTGGAAAGGAAAAGTAGAATCATGGAAAAACGAAAAGATAATGAAGAAGCAAACAACTCATTGGTATGTTATTTGATAGAGTGAACTGTAAAGTATTGAATTGTAGATATCATGTGGCTTTAAAAATTTGATATGTGTTATTTTGGCAGGAGTCATTTTCTGCTCTTCGCAAGGATGCTGCCAATGTTCTGGATTTCCTAGAGAGATTAAAGAATGAAGAAGATCAAAAGGCTGTTGATGTGGATCTGATTGAAAGCCTGAAATTGAAGCTGACATTTATTTGTACATATGTCCAGCTTTCTTATTCCGATTTGGAGAAGTTTGAAGATATAATGACTAGAAAAAGACAAGAGGTTGAGAATCTGCTTCAACCAATTTTGGATGATGATGGCAAAGACGTCGGGTGTAAATATGTCCTTACTAGCCTCGCCGGTAATATGGATGACTGTATAAGCTTGTATCATCGTTCTAAATCAGATGCCACCATGATGGATGAGCAATTGGGCTTCCTCCTCTTGAATCTCTCTCATCTATCCAAGCATCGTGCTGAAAAGATGTTTCCTGGAGTGACTCAATATGAGGTTCTTCAGAATGTATGTGGCAACATAAGAGATTTCCATGGATTGATAGTGAATTGTTGCATTAAGCATGAGATGGTTGAGAATGTCTTATCTCTGTTTCAACTGATGGCTGAGAGAGTAGGACGCTTCCTTTGGGAGGATCAGGCTGATGAAGACTCTCAACTCTCCGAGCTAGATGAGGATGATCAGAATGATAAAGACCCTCAACTCTTCAAGCTAGCACATCTACTCTTGAAGATTGTTCCAACTGAATTGGAGGTTATGCACATATGTTATAAAACTTTGAAAGCTTCAACTTCAACAGAAATTGGACGCTTCATTAAGAAGCTCCTGGAAACCTCTCCGGACATTCTCAGAGAATATCTGATTCATCTACAAGAGCATATGATAACTGTTATTACCCCTAACACTTCAGGGGCTCGAAACATTCATGTCATGATGGAATTCCTATTGATTATTCTTTCTGATATGCCGCCCAAGGACTTTATTCATCATGACAAACTTTTTGATCTCTTGGCTCGTGTTGTAGCACTTACCAGGGAGGTATCAACTCTTGTACGCGACTTGGAAGAGAAATTAAGGATTAAAGAGAGTACTGACGAAACAAATTGTGCAACCCTAAAGTTTCTGGAAAATATTGAACTCCTTAAGGAAGATCTCAAACATGTTTATCTGAAAGTCCCGGATTCATCTCAATATTGCTTCCCCATGAGTGATGGACCTCTCTTCATGCATCTGCTACAGAGACACTTAGATGATTTGCTGGATTCCAATGCTTATTCAATTGCTTTGATAAAGGAACAAATTGGGCTGGTGAAAGAAGACTTGGAATTCATAAGATCTTTTTTCGCGAATATTGAGCAAGGATTGTATAAAGATCTCTGGGAACGTGTTCTAGATGTGGCATATGAGGCAAAAGATGTCATAGATTCAATTATTGTTCGAGATAATGGTCTCTTACATCTTATTTTCTCACTTCCCATTACCAGAAAGAAGATGATGCTTATCAAAGAAGAGGTCTCTGATTTACATGAGAACATTTCCAAGAACAGAGGTCTCATCGTTGTGAACTCTCCCAAGAAACCAGTTGAGAGCAAGTCATTGACAACTGATAAAATAATTGTAGGTTTTGGTGAGGAGACAAACTTGATACTTAGAAAGCTCACCAGTGGACCGGCAGATCTAGATGTCATTTCGATCATTGGTATGCCGGGTTTAGGTAAAACTACTTTGGCGTACAAAGTATACAATGATAAATCAGTTTCTAGCCATTTCGACCTTCGTGCATGGTGCACGGTCGACCAAGTATATGACGAGAAGAAGTTGTTGGATAAAATTTTCAATCAAGTTAGTGACTCAAATTCAAAATTGAGTGAGAATATTGATGTTGCTGATAAACTACGGAAACAATTGTTTGGAAAGAGGTATCTTATTGTCTTAGATGACGTGTGGGATACTAATACATGGGATGAGCTAACAAGACCTTTTCCTGATGGTATGAAAGGAAGTAGAATTATTTTGACAACTCGAGAAAAGAAAGTTGCTTTGCATGGAAAGCTCTACACTGATCCTCTTAACCTTCGATTGCTAAGATCAGAAGAAAGTTGGGAGTTATTAGAGAAAAGGGCATTTGGAAACGAGAGTTGCCCTGATGAACTATTGGATGTTGGTAAAGAAATAGCCGAAAATTGTAAAGGGCTTCCTTTGGTGGTGGATCTGATTGCTGGAATCATTGCTGGGAGGGAAAAGAAAAAGAGTGTGTGGCTTGAAGTTGTAAATAATTTGCATTCCTTTATTTTGAAGAATGAAGTGGAAGTGATGAAAGTTATAGAAATAAGTTATGACCACTTACCTGATCACCTGAAGCCATGCTTGCTGTACTTTGCAAGTGCGCCGAAGGACTGGGTAACGACAATCCATGAGTTGAAACTTATTTGGGGTTTTGAAGGATTTGTGGAAAAGACAGATATGAAGAGTCTGGAAGAAGTGGTGAAAATTTATTTGGATGATTTAATTTCCAGTAGCTTGGTAATTTGTTTCAATGAGATAGGTGATTACCCTACTTGCCAACTTCATGATCTTGTGCATGACTTTTGTTTGATAAAAGCAAGAAAGGAAAAGTTGTGTGATCGGATAAGTTCAAGTGCTCCATCAGATTTGTTGCCACGTCAAATTAGCATTGATTATGATGATGATGAAGAGCACTTTGGGCTTAATTTTGTCCTGTTCGGTTCAAATAAGAAAAGGCATTCCGGTAAACACCTCTATTCTTTGACCATAAATGGAGATGAGCTGGACGACCATCTTTCTGATACATTTCATCTAAGACACTTGAGGCTTCTTAGAACCTTGCACCTGGAATCCTCTTTTATCATGGTTAAAGATTCTTTGCTGAATGAAATATGCATGTTGAATCATTTGAGGTACTTAAGCATTGGGACAGAAGTTAAATCTCTGCCTTTGTCTTTCTCAAACCTCTGGAATCTAGAAATCTTGTTTGTGGATAACAAAGAATCAACCTTGATACTATTACCGAGAATTTGGGATCTTGTAAAGTTGCAAGTGCTGTTCACGACTGCTTGTTCTTTCTTTGATATGGATGCAGATGAATCAATACTGATAGCAGAGGACACAAAGTTAGAGAACTTGACAGCATTAGGGGAACTCGTGCTTTCCTATTGGAAAGATACAGAGGATATTTTCAAAAGGCTTCCCAATCTTCAAGTGCTTCATTTCAAACTCAAGGAGTCATGGGATTATTCAACAGAGCAATATTGGTTCCCGAAATTGGATTTCCTAACTGAACTAGAAAAACTCACTGTAGATTTTGAAAGATCAAACACAAATGACAGTGGGTCCTCTGCAGCCATAAATCGGCCATGGGATTTTCACTTTCCTTCGAGTTTGAAAAGATTGCAATTGCATGAATTTCCTCTGACATCCGATTCACTATCAACAATAGCGAGACTGCTGAACCTTGAAGAGTTGTACCTTTATCGTACAATCATCCATGGGGAAGAATGGAACATGGGAGAAGAAGACACCTTTGAGAATCTCAAATGTTTGATGTTGAGTCAAGTGATTCTTTCCAAGTGGGAGGTTGGAGAGGAATCTTTTCCCACGCTTGAGAAATTAGAACTGTCGGACTGTCATAATCTTGAGGAGATTCCGTCTAGTTTTGGGGATATTTATTCCTTGAAAATTATCGAACTTGTAAGGAGCCCTCAACTTGAAAATTCCGCTCTCAAGATTAAGGAATATGCTGAAGATATGAGGGGAGGGGACGAGCTTCAGATCCTTGGCCAGAAGGATATCCCGTTATTTAAGTAGTTTTTGAGCATTATGGTTGAAAAGTAGATTGCACTTTGCTGGGTAGATTGTATATGGTTAAGAAAATTCTGTTACAGTTGTTATGAAACATTTTTATTTGACTTTTCTGAGTTTCTTTTAGAAAACTCAGAAGTTTTTAACAAAAATTATAGTTTTTATAAATACAATGTGGATTTGCCTTTGGCTGTCCAACTTGGTCTGAAGTCTCATATGCTCAGAGCACTATCGTTCAACCTCAATCAAGGTACTGATTTAAAATGACATCTATACTACTTTATCACAAACCCAACGAACTTTCATCTCAAAAGCTAGGCCAGGAAGTGAAGAGGTTGTAGAGAGCTTATAAGCACTCATGACTTCCTTTTCTCGAACATTCAACCAACGTAGGCTGAAATCCCACTCTGAACGAAAATAAGTGTTTGTTTATCAAATTAACTCTCGTAGTAGAACACTGAAATACCTTCTTCTAAACGTTCAACAAATGGGATTTCCAGCACTCAAAGTGAATGAAAGGTTCACATTAATCTTCAAAAAGAATTACGACAATTCATGACCACAAGTACATTGACAGCACCATTTCAACAGAAGAACAAGTCAATGCTGCATCTTCATCAATAATCCGAGTGTCGAACCTCCTTCCTGACACTGTCCTGTATATGTAAAGTTTCTCAACAGGGCAACTTTCTGGTCTCGTATCTGGATGACCCCTCTCGTCTATAACTTCAACATTAAGCCCTGGCAACTTCTGGACCAACAGCTTACATGCTTCAAAACTTACTGAACAATTAGACATCCAAAGGGATCGCATTGTCTCCAGCTTTGCAGCATTAGCCAACAGAGCCTCATCGCCAAAGGGGCAGTCTCTAATCTCGAATTTGAAAAAATTGTTGTTGTATGACTTTCCTCTGACATCCGATGCACTATCAACAATAGCAAGACTGGAGGTTGGAGAGGAATCCTTTATTATACAATCATTCAGGGAGAAGAATGGAACATGGGGGAGGAAGACACTTTTGAGAATCTGAAATGTGTTAGAGCCACAAGCTACAGAAGTATTGAATTTGTCATGAATATCAACATTCTTCATCCTAGTTAATTCTTTTTCAATTTTTAATAGACTCTCATTTTAATCACTAATATTCTTCTATTTGTGACTTCTTTTCTGCAGGTGGCAACTTTAAATTCATAAAGTATAGGATTGATGACAAACTCGAAAAATATCTTAATGAGGTGAAGTTTGAGCAGTCAGCAGATGGTGGTTCCAACTCTAAGTTGACAAGCACATACTATCCCGGAGGGCGATTTCAAGCCTGATGCATATGGTTAGTGTGGCTAGAGCAGACAGGATGTATTACCTGGATATCTACCAAGACGAATCCACAATCAGTTTTATGTCAAGCAATACATGAAGTAACTCCCGATAGAACAGTAAAAGCAAGATGTGTAGGTGTATCTCGACTCTAAGAGATTGTACATTCCTCTTTGAGATTTTTACTGCTAATACAAATTTACACCTCAGAAGCGAATCTAGAATTTCTAGAGCATGAATGCACCACTAATGAAAGGAGAAAAAAGGAAGTATGAAGTGGGAATTTGATCCTTGTTTCTAGGTATATAAAATTTATCATTCAACTATACTTCATTTAGCAAACAACTCTCTTTGCCATTATTTCTCAAACAAGGGCTTCTAATATTGCTAAACTAAAGACTGTCAAAAGGTAAGTTCATCTTCAAACTCTCTTGTTTACTTTATCTAAAGGGGAACTATGAAAAACAAGAAACATCAGGAATGTCCCGTAAACAAAGCAGCCTCATGCACAAAACATCCAACGTTGGTAGGATTAATGGAGGGATCGCATCCCAGGAGGATACTGTAGAAAAATTAGTGGCTTCTTTCACCGCTCAAACCCATGATCTATAGGTTACATGGAGACAACTTTATGGTTGCTCGTAGGCTCCCGTCAATTCTCATAAACCACAACACCAAAGTTGCATCAGACATCATCTTCATTCACAAGCTGACAATCTCCACAAGTCTTAGTCAACTTGTAATATGAATATTAGCCAGGTAGACGTACATATTTACAAAATTGAGTTTCCTATATAATATGGTTTGAAGGAATGAAACATGATGGGGAGGGTAGATAAAATAATATATGAGGCATAAAAATAGGAAAGATATTTGTAGTGAGAGGTTTTGACTTTTTATGCTGCTTTTGATCTTCAGTTTCTTGTATTCTTTTTCTACTGCTTTCCTCTTCTTTCTCCTGAGTAAAGTTTTATGTAGGTACTTTTTATACGTCCGATCGTGAGAACTTGAAAGAAAGCTCTCTATAGCTATGTTAGGTGCCCACATAAAAAAATGAAATATTACAAAAACCCTGATAATAAAATACACTAATCTAAGATATTCACTGCAACATACATGCAAAATATATATATATAAATTTTCATGAAAATTATAACAAATAATAGATGTGAACATATAACTTTAAAAATAATATTACATCCATAAAGCTTAAATTCTAGATC

>Rpi-blb1

GATCTTTTAAATATTTTGAATTAGCAATTATTGTGACTATAATACTTTTTACATAATTTGCAAATATATAAAATTTATTTTTTGAAAAAAAGAAGATTTCATGCGCAAATTCCAGGTCAAACTTAAATTATTAGACTCTCGAAAAATGAAAAGTGTCACATAAATTGACACAAAGGGAGTACTTGTTAATGTTGTAATTATTGGCGAACAATAATGTTGTTGATTATCACTTTCTGAATAAATGTTGTGTCACTTGGAAAAAACACCAAATAGAACTATTCATGTTTTTTCTTTAGTATATATAAATATGATCTTTAACTTAATTGCAGCAGACAGGCATGATCTTTAACTTTAAATGTGCACAAGTAGATTGACAGGCTTGCTAATTGAGTGTCTGTTATAATCAGTATTAATTACTCTCAAGGTAATAGTATATTCCAGACAAATTTTGTGTTACCAAATTAAATATATTTCTAAAACTCTCCTCAAAGTAGTTAATATACTTTTGAGTGTTGTATCATGTTTTTAATATAAAATGTTAAAATTTAGATGAAATTTACTTTCTAGTTAAATTGGTCAAAGTTGAAAGAATTTCAAGTGAAAAAGTTTTTAATAATTTGACTTTTATGCTATATTTTTTTAAAGTTGAACGACTTTTTAATAAAAAAGAATAATAAAATTATATGATAATTTTTATAATACAATGGCCTTTATATGATGAAAAAAAAAGAAAGAAATTAGATGACAACAATGTCCAAAAATAATCTTAAAGAATTACGATTTATATATAATAAAATTAAATTTAAAATTTGATGAAAAAATAGAGAAAAGAGGAAGATGATGAAGTGAAATGACGTGGTGGTGGGTCCATGTGACATAAAAAAAAATTCTCTTAAATAATCCTTTCATACTAATGATAAATTTTTTTTTTTTTTTTTTTTTTTACTAATTGCGTATAGAGAAAAGGAAAATGGGGCGGTAATTACAAAGTAGGGAATCGAACTTTATCAACAAGTTGAGAGTTCAAGTAATCAACCAACTAAACTACTAAAATTTTTCTAATTAATGATAATTGTAATTCATTTAGCATAAAAAATTTCATTGCACTTACTTTTAGAGTTTTGAAAACAGTACTTCATCTATTCTATATTAATTAAATTTTCTATATTAATTAAATTTGTGAGGTAATACAAACTTATTAAGAAAAATATTTAAGGACATAATTTAACTCATATTTTTCACTATTGTTTTTTGTGAAATCATAAATATAACTTTGTAAATAGTGCAATTTATCTCCTAGAAGCAAATTTCACCAAAGAAAAGGGCAAAGATGGAAAAGAAACTAAATATTCATCTTAAACTTTGAACAATTCAATTATTTTGAACAATGAAAAAAATCTCAAAAATTCAATTAATATGAAATGGAGAGAGTAACTTTATTTTAGAGGCAAAAAATTAGTACTCCATCCGTTCACTTTGATTTGTCATGTTGCACTTTTCGAAAGTCAATTTGACTAATTTTTAAAGCTAAATTAGATTACACTAATTCAATATTTTAAACAGAAAAATTAGATATTCAAAAACTATACAAAAAATATTATACATTGCAATTTTTTGCATATCAATATGATAAAAAAATATATCGTAAAATATTAGTCAAAATTTTTATAATTTGACTCAAATCATGAAAAGTATAATAATTAATAGTGGACGGAGGAAGTATTGTCTTTCCAGATTTGTGGCCATTTTTGGTCCAAGGGCCATTAGCAGTTCTCTTCATTTTCTACTTCTGTCTCATATTAGATGGGCATCTTACTAAAAATATTTGTCTCATATTACTTGATTATTTATTAAATCAAAAAGAATTAATTAATTTTTTCTCATTTTACCCCTACAATTAATATAGTTTTAAAAGTTTTAAACAAATTTTGAAGAATCAAAATTTCTTTTGCAAGAGACTTATTAATATAAACAAAGGATAAAATAATAAAAGCTGTCAATTTATTGACCATCACTTAATAATATATAAAATACAAACTGCTGATCTAATATGAGACGGACAAAATATATTCTAAAATATTTTCGGACAGATATGTGATATTCTAACCATTCACTACACTATATTATGCATTTTATCCGCCAATGACTTATTTCAGCTTTAATTAATTAGGAAAGAGGAAACTGCCAATGAGGAAGAGTAGGGGCGTAGTTGCTGTCGACGAAAAAAAGATAATACTCACTCTTTTCGATTTTTATTTTTATTTATCACTTTTAACCTATCATGTAAAAAGATAATTATTTTTTTCATGCTTTATCCTTAGTATTAAACAATTTAATAGGGATTATTTTGTAAAATATTTATATGAATAATTGTTTTCGTAATGAATTTGTCCGGTCAAACAATGATAAATAAAAATGAATGAAGAGAGTAGAAAACAAAACAAAAGAACAAGTTGACAACTTGAGAGATTAAAAGGGTCCAAAACGCCTTGGATTTTGAGATTCCATATGTGAAATTTCCATGAAATAATTGAATTTGTATTATTACAAGTCAAACTTTCCATTTCATTCCAACTAGCCATCTTGGTTTCAAAATTACACATTCATTCATTCACAGATCTAATATTCTTAATAGTGATTTCCACATATGGCTGAAGCTTTCATTCAAGTTCTGCTAGACAATCTCACTTCTTTCCTCAAAGGGGAACTTGTATTGCTTTTCGGTTTTCAAGATGAGTTCCAAAGGCTTTCAAGCATGTTTTCTACAATTCAAGCCGTCCTTGAAGATGCTCAGGAGAAGCAACTCAACAACAAGCCTCTAGAAAATTGGTTGCAAAAACTCAATGCTGCTACATATGAAGTCGATGACATCTTGGATGAATATAAAACCAAGGCCACAAGATTCTCCCAGTCTGAATATGGCCGTTATCATCCAAAGGTTATCCCTTTCCGTCACAAGGTCGGGAAAAGGATGGACCAAGTGATGAAAAAACTAAAGGCAATTGCTGAGGAAAGAAAGAATTTTCATTTGCACGAAAAAATTGTAGAGAGACAAGCTGTTAGACGGGAAACAGGTACTCATCTTAAATTAGTATTACAACAACTAAGTTTATATTCATTTTTTTGGCAATTATCAAATTCAGAAAAGGGTTAAATATACTCATGTCCTATCGTAAATAGTGTATATATACCTCTCGTTGTACTTTCGATCTGAATATACTTGTCAAATCTGGCAAGCTCAGAATCAAATTATCCACCCCAACTTTTAAATACTCGATATCTTTAGAAATCCACCTGTCTAACTCATCCACTACCCATTCCCTTTGCTTTGAATTCTTTTCTTTACCTATAAACTTGGAACACTCGATCCGTTTTGCTTTTCTTAACAAAGCAGCTCAGAGAAAAGAGGTTTTCTTCTATTCTGTTTCTCTGTGTGCTGCACTTGGGTCCTTAATCCCATTAAAAACAGGGCATGTTAATCCCAACGACGGTAGCCTTTCCTGACAGCTGACTGTAAATTTTGTCTAACAAAGAAAAAAAAAGATTAGACATGTTTTTCCTTGTCATTGATTAGGCTGGATTTCTTTCAGAGTGGAACATAGGGGATATATTGGACCAAAAGTAGAATGGGTATATATTTAAAGTATTTCTGATAGAACAGGAGTATATTGTGCGAAAATATCCTCTATTTTCTGTTGTCTCCTAATGAGTTTGAATGTAATAATATTCTCATGTGGACATTGCTTGCACCAGGTTCTGTATTAACCGAACCGCAGGTTTATGGAAGAGACAAAGAGAAAGATGAGATAGTGAAAATCCTAATAAACAATGTTAGTGATGCCCAACACCTTTCAGTCCTCCCAATACTTGGTATGGGGGGATTAGGAAAAACGACTCTTGCCCAAATGGTCTTCAATGACCAGAGAGTTACTGAGCATTTCCATTCCAAAATATGGATTTGTGTCTCGGAAGATTTTGATGAGAAGAGGTTAATAAAGGCAATTGTAGAATCTATTGAAGGAAGGCCACTACTTGGTGAGATGGACTTGGCTCCACTTCAAAAGAAGCTTCAGGAGTTGCTGAATGGAAAAAGATACTTGCTTGTCTTAGATGATGTTTGGAATGAAGATCAACAGAAGTGGGCTAATTTAAGAGCAGTCTTGAAGGTTGGAGCAAGTGGTGCTTCTGTTCTAACCACTACTCGTCTTGAAAAGGTTGGATCAATTATGGGAACATTGCAACCATATGAACTGTCAAATCTGTCTCAAGAAGATTGTTGGTTGTTGTTCATGCAACGTGCATTTGGACACCAAGAAGAAATAAATCCAAACCTTGTGGCAATCGGAAAGGAGATTGTGAAAAAAAGTGGTGGTGTGCCTCTAGCAGCCAAAACTCTTGGAGGTATTTTGTGCTTCAAGAGAGAAGAAAGAGCATGGGAACATGTGAGAGACAGTCCGATTTGGAATTTGCCTCAAGATGAAAGTTCTATTCTGCCTGCCCTGAGGCTTAGTTACCATCAACTTCCACTTGATTTGAAACAATGCTTTGCGTATTGTGCGGTGTTCCCAAAGGATGCCAAAATGGAAAAAGAAAAGCTAATCTCTCTCTGGATGGCGCATGGTTTTCTTTTATCAAAAGGAAACATGGAGCTAGAGGATGTGGGCGATGAAGTATGGAAAGAATTATACTTGAGGTCTTTTTTCCAAGAGATTGAAGTTAAAGATGGTAAAACTTATTTCAAGATGCATGATCTCATCCATGATTTGGCAACATCTCTGTTTTCAGCAAACACATCAAGCAGCAATATCCGTGAAATAAATAAACACAGTTACACACATATGATGTCCATTGGTTTCGCCGAAGTGGTGTTTTTTTACACTCTTCCCCCCTTGGAAAAGTTTATCTCGTTAAGAGTGCTTAATCTAGGTGATTCGACATTTAATAAGTTACCATCTTCCATTGGAGATCTAGTACATTTAAGATACTTGAACCTGTATGGCAGTGGCATGCGTAGTCTTCCAAAGCAGTTATGCAAGCTTCAAAATCTGCAAACTCTTGATCTACAATATTGCACCAAGCTTTGTTGTTTGCCAAAAGAAACAAGTAAACTTGGTAGTCTCCGAAATCTTTTACTTGATGGTAGCCAGTCATTGACTTGTATGCCACCAAGGATAGGATCATTGACATGCCTTAAGACTCTAGGTCAATTTGTTGTTGGAAGGAAGAAAGGTTATCAACTTGGTGAACTAGGAAACCTAAATCTCTATGGCTCAATTAAAATCTCGCATCTTGAGAGAGTGAAGAATGATAAGGACGCAAAAGAAGCCAATTTATCTGCAAAAGGGAATCTGCATTCTTTAAGCATGAGTTGGAATAACTTTGGACCACATATATATGAATCAGAAGAAGTTAAAGTGCTTGAAGCCCTCAAACCACACTCCAATCTGACTTCTTTAAAAATCTATGGCTTCAGAGGAATCCATCTCCCAGAGTGGATGAATCACTCAGTATTGAAAAATATTGTCTCTATTCTAATTAGCAACTTCAGAAACTGCTCATGCTTACCACCCTTTGGTGATCTGCCTTGTCTAGAAAGTCTAGAGTTACACTGGGGGTCTGCGGATGTGGAGTATGTTGAAGAAGTGGATATTGATGTTCATTCTGGATTCCCCACAAGAATAAGGTTTCCATCCTTGAGGAAACTTGATATATGGGACTTTGGTAGTCTGAAAGGATTGCTGAAAAAGGAAGGAGAAGAGCAATTCCCTGTGCTTGAAGAGATGATAATTCACGAGTGCCCTTTTCTGACCCTTTCTTCTAATCTTAGGGCTCTTACTTCCCTCAGAATTTGCTATAATAAAGTAGCTACTTCATTCCCAGAAGAGATGTTCAAAAACCTTGCAAATCTCAAATACTTGACAATCTCTCGGTGCAATAATCTCAAAGAGCTGCCTACCAGCTTGGCTAGTCTGAATGCTTTGAAAAGTCTAAAAATTCAATTGTGTTGCGCACTAGAGAGTCTCCCTGAGGAAGGGCTGGAAGGTTTATCTTCACTCACAGAGTTATTTGTTGAACACTGTAACATGCTAAAATGTTTACCAGAGGGATTGCAGCACCTAACAACCCTCACAAGTTTAAAAATTCGGGGATGTCCACAACTGATCAAGCGGTGTGAGAAGGGAATAGGAGAAGACTGGCACAAAATTTCTCACATTCCTAATGTGAATATATATATTTAAGTTATTTGCTATTGTTTCTTTGTTTGTGAGTCTTTTTGGTTCCTGCCATTGTGATTGCATGTAATTTTTTTCTAGGGTTGTTTGTTTGTTGAGTCTCTCTCTCATTGGATGTAATTCTCTTTTGGTAACAAATTAACAATCTATTTGTATTATACGCTTTCAGAATCTATTACTTATTTGTAATTGTTTCTTTGTTTGTAAATTGTGAGTATCTTATTGTATGGAATTTTCTGATTTTATTTTGAAAACAAATCAATAAGATCCATCTGTATTATACTCCCTTCGTCTCATTTTATGTGACACTTTTTGGATTTCGAGATTCTTTGATCTTAAATTTTTCATAGATCTTTTAAACATTTTGAATTATCAATTATTGAGATTTTAGTATTTTTTATGTAGTTTACAAATATATAAAATTAATTTTTTAAAAAAAAGAAGATTTCATGCGCATATTCCCGATCAAACTTAAATTACTAGACTCTCGAAAAATGAAAAGTGTCACATAAATTGAGACAGAGGGAGTACTTGTTAATGTTGTAATTATTGGCGAACAATAATGTTGGTGATTATCACTTTCTGAATAAATGTTGTGTCACGTGGAAAAAACACCAAATAGAAGTATTCATGCTTTTTTAGTATATATAAACATGATTTTTAACTTGGTTTCAGCGGATAGTCATGACCTTTAACTCTGAATGTGCACAAGTAGATACTTGTATAAAATTAAACAAATTTTATAAAATTATACAATATGACACTGAGAGTAATTGATACCAATTGCAGTCGTTGCTGCTTTTCGATTCTCTGTCATTCTCTAGGTAATTGATTTTACAGAAAAGGGCCAAAAATATCCCTGAAGTACCAGAAAAGGTCTCAAAATACCAACCATCCACATTTTGGTCTAAAAATATCCTTCTACTCATCCTTTTTTGTCTAAAATTACCCTTTCATCCACATTTTTGCTCACTTATACCCTTATAACAACTCTCTCCTTTTTTAAAAAAAAATATTTATTATGTGTCATTTTCTTATTGAATGAAATAAAAATCCACCTCTATTAATTTTTTCCCATAATTTATCCAAATCAAAACAATATATTTTTTCAAGATC

>Rpi-ber

ATAGGAGAAACATAGAGAAGGTGATGTGACACCTCTCTATGACCTCCATTCATATATTTTTTTTTTCCTTTTTTTCTTATTTAAATCAATTATTTTATTGTAAAAGTAAATTGATTAATTTTTTGTAAATAAATATTTTATTAATAGAGGAACAATTACAACAAAAACTCTTCATATGCCAATTAACAAAGAGTAACTATCTATAACTCACACCTCTCTAATTTTCAATTTTAATAAATTTATTTTTATTTGTATCTCTTTTTTTACCTTCTTCTAATCGTAAAATGACAACTTTTTTTTAATATCTTCTCTCTTTATTATTTAATTTCTTTACAATCTATCTATATCGATGTATTTTAAGTATTCACGTTTTCAATGCTTATTATGTTTGATGCTTTGGATTGACTTGAAAGAAGATGAAGGCTATTGAATGATGGCTATGGCTTGTGAAAATGGATATCAATAAAAGCTCAAAAAATTGTATATTAATTTCATTTTTAAAAATATTTTAATAATTAAGAACATAAAAATAATACAATTATTTATTATTTTAATTCTTTCATGATTTCTCTTTATCAAACATCAAAAGTTCATTTTATAAATGGATTGCACCTAGAGTTGCATTCCCCATCATAAATATGATTATTTTTAATCATTTATATGTGTAAGAGCCGTTTTTAAAATTCTGTATTGCGATTCTAATATTAAAAAAAATAATTTATAATGTTTTACTGATTTTTATAGTAACACTGTACTAATGATTTGTTTTTCCTATTCCTGACTATATTAATTACTTGCATATTCTCAAATTAAATGGGATAATTAATGGGTAATAATGTTTATACTTCTTCATATTTTATAACATGTTTTTATGCGTGATTTCTGTAATAACTTTTCTTATATTTTATGTTGAGACTTTGAAAATATATTTTGGTTATTTATCTTTATTATTGTGAAGTTAGAAGATGAAAAGGGAATATGATTGGACAATTTTCAAAAGATAATGAGTAATTATGTCGGAAAGTATTTGAGGAGATTAAGTTAAAAGATTAAAAATAAATATTCATGTTTTAAAATTTAGTAATACATATACTTAAAGAAAGAATTTGATTTTTCTTGAACTTCTATCCTTTCTTTACATTCTTTTAACAAATATAAAAATATCATTTCACTTATATAGATTATACATTATACAAGTTTTATAGAGAGGCTTTGAATTTTCAAGTGCTGAAAAATCATAGAAAATGTACTAATAATATAATTGATTAGAATATTATACAAGTTTTATGGAAAGGCTCTGAATTTTTAAGTGCTAAAAAATCATAGAAACTTTACTAATAATATAATTGATTAAAATTTAAAAGATTTAAGAAGAAATCTAATATCAAAAATTCATACGCGCGAAACGCAGATAAGTTCTCTAATAAAAATGTAAAATAGAACTACTTGTACAAATAAAACAAAAAACTACTTAAATAAATTAATATAAAGGGAAAAAGAGACTCAAACTTGGTTTCATCCACATGTATTTTTTTTTTTATTACTGTTTGAATGATTGTATATGAATTATTGTCTTCCTTTGAGTACTCTACAAACAACTACCAAACGAAGACTTACTTTGAGAAGACTTTGTTGCAAACAGCTCAGGAAGAAGACTTTGGTGCAGATTGCAGAGGAAGAGGAAGCAAACACAACTATGGCCGATGCTGTAATTGGTGCTACTGTTCAAGTTTTGCTTGAAAAGTTGATTTCTCTCACTATCGAGGAGGTCAACAGCTCAAGGGATTTCAACAAAGATCTCGAAATGTTGACACAAAATGTATCTTTAATCCAAGCTTTCATTCATGATGTTGAAACACCACAAGAGAAACAACAGTCTGTGGAACAATGGCTCAACAGGCTTGAGAGAGTTGCTGAAGATGCTGAAAATGTGTTTGATCGATTCAGATATGAATCTCTCAAAACAAAAGTGGTGAGGAGCCCATTGAAAAAGGTCAGTGGTTTCTTTTCTCATACTGCTTTTAAGAGAAAAATGTCTCAAAAAATCAACAACATAAATAAAGAGTTGACGGCTATCAATAAGGTAGCCAAAGACCTCGGTCTACAATCACTCATGGTACCTTCTTGGAAAATACTACCAATTCGAGAAACAGATTCCTTCGTAGGTGCTTCTGATGTTGTTGGTAGAGATTTGGATGTTGCTGAGATAAAGGAGAAGATTTTGAACATGAGAGAGGAGGATGTTGTTCTGTCCACCATTCCCATAGTAGGTATGGGAGGTTTAGGGAAAACAACTGTGGCTAAGAGGATTTACAATGATGAACACATCAAGCAAATCTTTAAAGAGAGAATTTGGTTGTGTATACCTGAAATGTCTGAAACGAAGAGCTTTCTTCAACAAATCCTCGAATCGTTGATAGAGAGGAAAATTGAGGTTGAAAGGAGAGATATAATAGTCAAGAAGCTACAAGATGAATTGGGAGGAAAAAAATATTTGCTAGTCCTGGATGATTTGTGGCGTGTTGACTCTACATCGTGGCATGAGTTCGTCGACACCCTGAGAGGAATAAATACATCCAGAGGAAACTGCATTCTTGTGACTACTCGTAGGAAGCAGGTGGCATCCACAGTAGCAACAGATCTTCATATCTTGGGGAAGTTAACAGAAGATCATTGTTGGTCTATTTTCAAACAAAAAGCATTTGTTGATGGCAGGGTTCCAGAGGAATTAGCGAGCATGGGCAACAAGATTGTTAAAATGTGCCAAGGTCTACCGTTGGCTGCAAGTGTGTTGGGAGGGCTCTTACACAACAAAGAAAAACATGAATGGCAAGCAATTCTTGATGGCAACCTCCTTGTTGCAGGTGAAGATGATAATGGAGAAAATAGCATAAAGAAAATCCTAAAACTCAGCTATGATTATCTACCATCTCCACATCTGAAAAAATGCTTTGCTTACTTTGCAATGTTTCCAAAAGATTATATGTTTGAAAAGGACCAACTAATCCAACTCTGGATGGCAGAAGGGTTTCTTCGTCCAAGTCAAGAGATCCCTGTGATGGAAGACGTTGGGCACAGGTTTTTTCAAATCTTGTTGCAGAATTCCTTGCTACAAGATGTTCTGTTAGATGAACACAACAATATAACACACTGCAAGATGCACGATCTTGTGCATGATTTGGCTGGAGATATCTTAAAATCTAGACTATTTGATCCGAAGGGCAACAATGGAGAAAAACTTTCTCAAGTTCGATACTTTGGATGTGAGTCACCAACGGATCAAATAGATAAGATATATGAGCCAGAACGTTTGTGCACACTGTTTTGGAGAAGCAATTATACATCTAAAGATATGCTGTTGAACTTTAAGTTCTTGAGAGTTTTAGATTTGTCCAGTTCAGGAATCAAGGAGTTGTCAGCCAAAATCGGGAAGCTGATATACTTGAGATATCTTGATCTCTCGAACACTGAGATCACAGCCTTGCCCAACTCCATTTGCAAGCTCTATAATTTGCAAACATTTAGAGTCATCAACTGCTTTTCACTCCAGGAACTTCCATATGAGATGAGAAATATGATAAGTTTGAGACACATATATTACACTTCTGTTGACGAAACAAGTGGGCATTGGGGAGGATGGTGTCTTCACAATGAACATTTTCAGATTCCACTTAATATGGGGCAATTGACTAGTCTTCAAACCCTCAAGTTTTTCAAGGTAGGTTTAGAGAAAGGTCGTCAAATAGAAGAATTAGGTCATTTGAAAAACCTAAGAGGTGAATTGACGATCAATGGTCTCCAATTGGTCTGTGATAAAGAAGAGGCTCAAACAGCATATTTACACGATAAACCAAACATCTGCAAGCTGGCATATTTATGGTCCCATGATGAATCAGAAGGCTGTGAGATCAATGATGAGCATGTGTTGGATGGTCTTCAACCGCATCCTAACTTGAAAACCTTAGCAGTAGTGGACTATTTAGGGACTAAATTTCCTTCATGGTTCAGTGAAGAGTCGCTACCAAATTTGGTCGAGTTGAAATTAAGTGGTAGCAAAAGGTGCAAAGAAATTCCATCCCTTGGCCAACTGAAATTCCTTCGGCATCTTGAGCTGGTAGGATTCCATGAGTTGGAATGCATTGGACCTGCTTTTTATGGTGTTGAGATGAGAAATATTGGATCAAACAGCATTATCCAAGTGTTCCCGTCATTGAAAAAACTAGTATTGAAGGATATGCGTAGCCTTATTGAGTGGAAGGGAGATGAAGTTGGAGTAAGAATGTCTCCCGGTCTTGAGAAGTTGCGGATTACAGACTGTCCATTGTTAAAAAGTATTCCGAATCAATTTGAAATCCTCCGTCAATTAAAAATTACAGGAGTTGACAGTGAAATGCCATTGTTGAACTTGTGCAGCAACTTGACATCTCTCGTAAAGCTTAGAGTCTATGATATGAAAGAGCTCACTTGTCTTCCAGATGAGATGCTACGTAACAACGTTTCTCTTCAACAGATAATAATTTTCAACTGCGGAGAGTTTCGTGAATTGCCACAAAGCTTGTACAATCTCCATTCTCTTAGGAGATTAGACATTTACAACTGCACCAATTTCAGTTCTCTTCCTGTTCCCAATGGAGGCAACTATTTGACTTCCCTCGAATTCTTTTGCTTATATAATTGTAATGGATTGATCAGTATACCAATTGGAATGCTAGATCAATGCCGGCTAGTGTTTTTGAATGTCAGCTGCTGTAACAACTTGGTTTCATTCCCTGTACATGTGTGGGAAATGCCTTCACTTTCATATTTGGTTATATCAGAATGTCCCAAATTGATTAGTGTACCCAAAGTGGGCCTTCACCATCTCACCGGGTTAGTGAGATTGGGAATTGGTCCTTTCTCAGAGATGGTGGATTTTGATGCATTCCAATTGATTTTTAATGGCATTCAGCAGTTGTTGTCCCTTCGTGATCTGGAGGTGTACGGACGTGGGCACTGGGATTCTCTGCCCTATCAGCTTATGCAACTCTCTGACCTAAGAGAGATCACAATAGCTGATTTCGGAATTGAGGCTCTTCCTCCTACTCTTGACAACCTTACTTCTCTTGAAAGTTTGACGCTAGTGAGGTGCAAACAGCTACAACATCTGAACTTCTCAGATGCCATGCCCAAATTACGGCTCCTGTGGATACGTGATTGTCCATTGTTAGAAGCTCTGTCGGATGGGCTCGGCAACCTTGTTTCTTTGGAAGAATTATATTTACATGACTGCGAAAAACTAGAGCATCTACCGTCCCGAGATGCCATGCGACGCCTCACTAAATTATGGAACATGAGAATTAAAGGATGCCCAAAGTTAGAAGAAAGTTTCACCAACTACTCCCAGTGGTCCAAAATTTCCCATATTTCAAATATTGAATTAGGTGGGTGGAGAAGGACAGCCATAAGTCTCGGTTTCTCTTTCACTTTCTGAGTCGCTTGCATTTTTAATTAGAATATTATTTTCCACGAGTTCCAAAAAAGAAGTTTTGTAAGTAATGTAATAATTTTTCTTGAAATTATTTTGACATAAAAAATAAAGCTAGATTATCAATCAATTGCATCTGTTGCTATGCTTTCAGGATTCTGCTTGTCCACTTGAACTGAATTGCTGTTATTCTAAAAATTATGTTTCTTTTCCTTATAGATAGCCATTATAGACGTCTCTGTGGTAAATCAAGCCAAATCATATGAGCAGCAGGCAATTACGTAAGGTAAATTTTCATTTATAATTTATAGGCACTTTGTAATCTACTTGTTTCTATCTTTCTTTTTGTTGGAAACCAATTTTTGGAGGTCTATGCTGATGAATACAACATTACCTTCCTTAAAAAAAAGTTTAATCATGAAAATTTATGATTTCTGCTCATAGGTTACTAGGTACAAGGACATGGTAGAACGTCGTTGTGGTATATCTAGTCAAAATCCATGGGCAGAATCATGTTAGGCATTTTTATTTAGTTGTTAGAGGAATGCTATAACAAGTTTGTAAAGTTTCTCTTTTTTTACTTTCCTTTTCTCTCATGAAATAATTTTAATGTTTAACGGGACTTGTCGTGTTTCACAGAGGAACCGCCACTGTTATTTTGTATGAAGAACAATCTTCAACTGTAGACTCATTATGGGACACTTTCATGAGTATTTTCGGGGAACTACAGTCTACATATGAAACATCAAAAACTCAAAAGCGTATAGGGTGCTCAATGAGAAGCTACACGGGGTCTATTCTCCATGTAAGTATTGATAACTCTCAAGGAACAATACCAAGTCTCATATATGGTAGAAATCTATATAAGTTTCTTAATAGACTCATAAAAGAATAATCTTTATGTCTTCTGCAGTTCTGGTTTTATTTTCATGCTTAAGACAGTACAACTTTTATGAATATATGACACACTTCTCGTTACTTTCACCTCACAGGACAATTTCTCTGTTCATTAAAACTTCCGCGAATGAAGGACTTCAACAAAGATAGTTGTGATGAATATTTTTGCAAAGGGTGACCCATTGGCTCTTAAAATGTCCTTAGCAGTATACGTTCATCTTA

# Appendix 2: Coordinates of the reference NLR CDS (start – stop)

| **Gene** | **Start position** | **Stop position** |
| --- | --- | --- |
| *Nem-Gpa2* | 4875 | 7851 |
| *Nem-Gpa2^ΔC2922^* | 4875 | 7850 |
| *Rpi-abpt* | 250 | 2788 |
| *Rpi-abpt^T86^* | 250 | 2788 |
| *Virus-Rx* | 11848 | 14893 |
| *Rpi-vnt1.3* | 256 | 2974 |
| *Rpi-vnt1.1* | 709 | 3385 |
| *Rpi-vnt1.1^A2056^* | 709 | 3385 |
| *Rpi-sto1* | 2249 | 5842 |
| *Rpi-sto1^T3144^* | 2249 | 5842 |
| *Rpi-R9a* | 3313 | 5905 |
| *Rpi-R8* | 1679 | 5417 |
| *Rpi-R3b* | 250 | 4102 |
| *Rpi-R3b^G3111^* | 250 | 4102 |
| *Rpi-R3b^G1696/G3111^* | 250 | 4102 |
| *Rpi-R3a* | 3324 | 7173 |
| *Rpi-R2-like* | 250 | 2794 |
| *Rpi-R1* | 1061 | 5163 |
| *Rpi-R1^ΔT4109^* | 1061 | 5163 |
| *Rpi-pta1* | 2267 | 5858 |
| *Rpi-pta1^A2012^* | 2267 | 5858 |
| *Rpi-mcq1.1* | 250 | 2839 |
| *Rpi-blb3* | 250 | 2794 |
| *Rpi-blb2* | 1545 | 5435 |
| *Rpi-blb1* | 2647 | 6239 |
| *Rpi-ber* | 1561 | 5470 |
